# The Histone Chaperone Network Is Highly Conserved in *Physarum polycephalum*

**DOI:** 10.3390/ijms24021051

**Published:** 2023-01-05

**Authors:** Axel Poulet, Ellyn Rousselot, Stéphane Téletchéa, Céline Noirot, Yannick Jacob, Josien van Wolfswinkel, Christophe Thiriet, Céline Duc

**Affiliations:** 1 Department of Molecular, Cellular and Developmental Biology, Faculty of Arts and Sciences, Yale University, New Haven, CT 06511, USA; 2 Faculté des Sciences et Techniques, Nantes Université, CNRS, US2B, UMR 6286, 44000 Nantes, France; 3 INRAE, UR 875 Unité de Mathématique et Informatique Appliquées, Genotoul Bioinfo Auzeville, 31326 Castanet-Tolosan, France; 4 Université Rennes 1, CNRS, IGDR (Institut de Génétique et Développement de Rennes)—UMR 6290, 35043 Rennes, France

**Keywords:** histone chaperones, protein domains, phylogeny, cell cycle, *Physarum*

## Abstract

The nucleosome is composed of histones and DNA. Prior to their deposition on chromatin, histones are shielded by specialized and diverse proteins known as histone chaperones. They escort histones during their entire cellular life and ensure their proper incorporation in chromatin. *Physarum polycephalum* is a Mycetozoan, a clade located at the crown of the eukaryotic tree. We previously found that histones, which are highly conserved between plants and animals, are also highly conserved in *Physarum*. However, histone chaperones differ significantly between animal and plant kingdoms, and this thus probed us to further study the conservation of histone chaperones in *Physarum* and their evolution relative to animal and plants. Most of the known histone chaperones and their functional domains are conserved as well as key residues required for histone and chaperone interactions. *Physarum* is divergent from yeast, plants and animals, but PpHIRA, PpCABIN1 and PpSPT6 are similar in structure to plant orthologues. PpFACT is closely related to the yeast complex, and the *Physarum* genome encodes the animal-specific APFL chaperone. Furthermore, we performed RNA sequencing to monitor chaperone expression during the cell cycle and uncovered two distinct patterns during S-phase. In summary, our study demonstrates the conserved role of histone chaperones in handling histones in an early-branching eukaryote.

## 1. Introduction

In eukaryotes, DNA is wrapped around histone octamers to form the chromatin. The basic subunit of chromatin is the nucleosome and consists of 147 bp of DNA wrapped around a tetramer of histones H3 and H4, flanked by two dimers of histones: H2A and H2B [1]. Besides these four histones, there is a fifth histone, H1, also known as the linker histone as it binds to the linker DNA between nucleosomes. Histones are among the most conserved proteins in eukaryotes. Each family of histones (H1, H2A, H2B, H3, H4) is represented by several isoforms. These histone isoforms are classified based on their timing of expression during the cell cycle [2,3]: (i) canonical histones are synthetized during S-phase and used for chromatin replication, while (ii) replication-independent histones are produced throughout the cell cycle and are required for specialized functions at chromatin and named histone variants. Beyond its role in DNA compaction, chromatin carries the epigenetic information, and is a highly dynamic compartment since all mechanisms operating on DNA (e.g., replication, transcription and DNA repair) require the eviction, storage and deposition of histones in chromatin.

**Figure 1:**
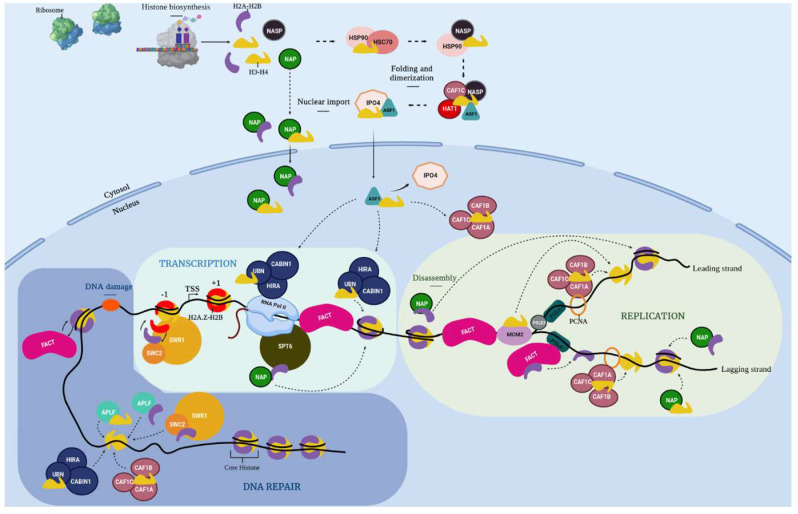
Schema of the histone chaperone network in *Physarum polycephalum.* Histone proteins are synthetized in the cytoplasm by the ribosome. H3 and H4 are taken in charge by the heat shock proteins **HSC70** (Heat Shock Cognate 70) and **HSP90** (90-KDa Heat Shock Protein) that assist their folding before H4 get diacetylated at lysines 5 and 12 by **HAT1** (Histone Acetyl Transferase 1) in the cytoplasm. Then, the HAT1-**NASP** (Nuclear Autoantigenic Sperm Protein)-**CAF1C** (Chromatin Assembly Factor 1C)-**ASF1** (Anti-Silencing Factor 1)-**IPO4** (ImPortin 4) complex ensures the H3/H4 nuclear import. The **NAP** (Nucleosome Assembly Protein) family contributes to the nuclear import of H2A/H2B. Once H3/H4 proteins reach the nucleus, ASF1 is the main histone donor that transfers them to the histone deposition complexes **CAF-1** (Chromatin Assembly Factor-1) and HIR (HIstone Regulator). The CAF-1 complex deposits H3.1/H4 during replication, while the HIR complex deposits H3.3/H4 during the whole cell cycle in chromatin. The NAP proteins ensure deposition of H2A/H2B in chromatin. The **SWR-C** (SWR1 Remodeling-Complex, SWi2/snf2-Related 1) complex is involved in the histone exchange reaction to deposit H2A.Z/H2AB in chromatin. It is composed of **SWR1** (SWi2/snf2-Related 1) and **SWC2** (SWr Complex 2). During **replication**, MCM2 participates in the unwinding of the dsDNA which seems to disrupt nucleosomes. The H2A/H2B dimers are evicted and escorted by the FACT (FAcilitates Chromatin Transcription) complex. Before eviction of H3/H4 tetramers, ASF1 is recruited to form the MCM2-H3/H4-ASF1 co-chaperone complex. After the passage of the replication fork, parental histones are recycled and deposited along with newly synthetized histones thanks to the CAF-1 and FACT complexes and NAP proteins. Moreover, PolE3 also participate in the deposition of parental and newly synthesized H3/H4 in chromatin on the leading strand of DNA. During **transcription**, nucleosomes are disassembled to enable the passage of the RNA polymerase II. The SPT6 (SuPpressor of Ty 6) and ASF1 chaperones, the NAP proteins as well as the HIR, FACT and SWR-C participate in the restoration of the chromatin landscape after the RNA polymerase II passage by mediating histone recycling or deposition in the wake of the polymerase. During **DNA repair** after DNA damage (labelled by an orange cloud), γH2A.X histones are deposited at double strand DNA breaks and then, when repair is complete, they are removed by the SWR-C complex to be replaced by H2A/H2B or unmodified H2AX/H2B by the FACT complex. The HIR and CAF-1 complexes, as well as the APLF chaperone, participate in the re-establishment of histones in chromatin. Since the sub-functionalization of **PpNAP1L1** and **PpSET** remains to be investigated, they were indicated as NAP.

After translation, histone proteins have to be translocated from the cytoplasm to the nucleus and incorporated at the right place and time in chromatin. In the context in which various cellular machineries need access to DNA, histones oftentimes need to be transiently evicted from chromatin and stored. Since histones are highly basic and charged proteins, their presence as “free” proteins in the cell can have deleterious effects. Hence, when histones are not incorporated in chromatin, they are always escorted by specialized proteins known as histone chaperones to form the so-called soluble histone pool. Histone chaperones ensure histone incorporation in chromatin, and also participate in transporting and storing histones and recycling them when they are evicted from chromatin. Because of all these roles, histone chaperones are crucial players that regulate the histone cellular supply. Categorization of histone chaperones is typically based on the specific histone isoform they interact with, or the cellular mechanism (e.g., replication) in which they are involved [4]. We can summarize the histone chaperone network, from histone biogenesis to chromatin incorporation, as shown in Figure 1, which represents the chaperone network from proteins identified in *Physarum*. The heat shock proteins HSC70 and HSP90 (70- and 90-kDa Heat Shock Proteins) are early cytoplasmic chaperones that assist with H3 and H4 folding [5,6]. HAT1 participates with the NASP-p46^RbAp46^-ASF1-IPO4 complex for H3/H4 nuclear import [5,7] and is conserved in *Physarum* [8]. The ASF1 chaperone is the main histone donor that shuttles H3/H4 from the cytoplasm to the nucleus, and transfers H3/H4 to the histone deposition complexes CAF-1 and HIR [5,9]. The CAF-1 complex mediates canonical H3.1/H4 nucleosomal assembly during replication [10]. The variant H3.3 is either incorporated in chromatin by a gap filling mechanism or histone replacement (i.e., substitution of canonical histones by their variants) by several complexes such as the HIR complex [11]. MCM2 is part of the replicative helicase that co-operates with several chaperones to handle histones during replication [12], while the accessory subunit of the Polε polymerase PolE3 participates in the deposition of parental and newly synthesized H3/H4 in chromatin.

Similar to H3/H4, H2A/H2B necessitate several histone chaperones, but they are less well characterized so far. The NAP family contributes to the nuclear import of H2A/H2B and H2A.Z/H2B and deposition of H2A/H2B in chromatin [13]. During processes such as developmental transitions, a global transcription reprogramming is achieved via chromatin remodeling and through substitution of canonical histones by histone variants, a process called histone replacement. The SWR-C complex is involved in H2A.Z/H2AB deposition in chromatin [14,15,16,17,18]. The FACT complex enables the displacement and turnover of H2A/H2B dimers [19]. Furthermore, NAP and FACT proteins are involved in the recycling of parental H3/H4 and H2A/H2B histones and deposition of newly synthetized histones during replication.

Besides replication, transcription also depends on restoring the chromatin landscape after passage of RNA polymerase II, and several histone chaperones such as SPT6, FACT, ASF1, HIRA or NAP proteins participate in this mechanism. Thus, histone chaperones strongly cooperate in a network to fulfill histone cellular supply. We previously reported a complete description of histones in the Mycetozoan *Physarum polycephalum* which led us to conclude that this organism is at the crown of the eukaryotic tree, based on histone phylogenetic analyses, and its histones are evolutionary closer to animal histones than plant proteins [20]. Since histones are highly conserved in *Physarum*, it raises the questions regarding (i) the extent of conservation of the histone chaperones (i.e., the histone-binding partners) in *Physarum* and (ii) to which kingdom (from plants and animals) they are most evolutionarily related.

*Physarum polycephalum* belongs to the Mycetozoans. This organism presents a multiphase life cycle, comprising a vegetative stage named plasmodium which consists of a syncytium. The syncytium in *Physarum* is a cytoplasm containing millions of nuclei originating from nuclear division without cytodiesis. This intriguing structure enables this slime mold to exist as a giant cell of a size varying from the micrometer to the centimeter scale. Due to its natural synchrony, *Physarum* constitutes a unique model to study epigenetic mechanisms occurring during the cell cycle at the single cell level. In a previous study [20], our phylogenetic and protein sequence analyses focused on histones from several animals, plants and unicellular organisms (including *Physarum*) that led us to position *Physarum* in the tree of life, and to identify the various histone isoforms for each of the five histone families. Here, we performed an in-depth study that identified histone chaperones in *Physarum*. We carried out a comparative analysis of histone chaperone sequences that showed a deep conservation of characteristic domains and key residues. In addition, we carried out transcript quantitative analyses by RNA-seq throughout the cell cycle, which showed two main expression patterns of histone chaperones during the cell cycle. Our comprehensive analyses suggest that most components of the sophisticated chaperone network that escort histones are highly conserved in *Physarum*, and that this slime mold is located at the crown of the eukaryotic tree.

## 2. Results

### 2.1. Genome-Wide Identification of Histone Chaperones in Physarum

**Figure 2:**
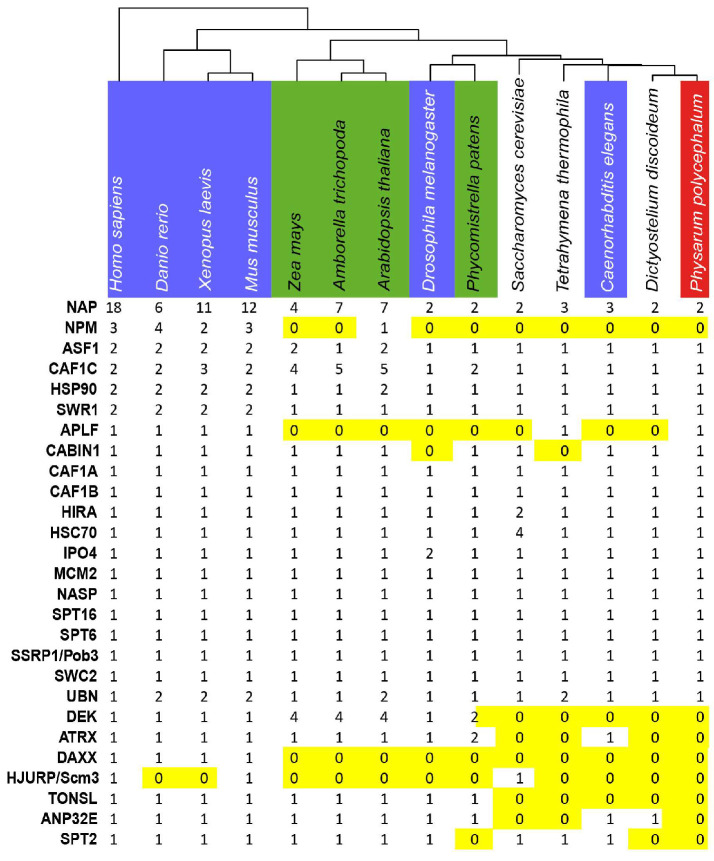
Distribution of histone chaperones in eukaryotes. Selected species used in this study from left to right: animals (blue), plants (green), unicellular and *Physarum* (red). The distribution of the various histone chaperones (rows) in the 14 species (columns) is displayed. Absence (0) of a given protein is highlighted in yellow. *Atr: Amborella trichopoda; At: Arabidopsis thaliana; Ce: Caenorhabditis elegans; Dd: Dictyostellium discoideum; Dr: Danio rerio; Dm: Drosophila melanogaster; Hs: Homo sapiens; Mm: Mus musculus; Pp: Physarum polycephalum; Ppa: Physcomitrella patens, Sc: Saccharomyces cerevisiae; Tt: Tetrahymena thermophila; Zm: Zea mays*.

We used the *Physarum* reference transcriptome [21] to identify the *Physarum* orthologues of the main human histone chaperones. We identified 21 genes and their corresponding transcripts encoding chaperone orthologues in *Physarum* (Figure 2, Table S1, Figure S1). Only a single orthologue was found in *Physarum* for the human histone chaperones Hs-p46^RbAp46^ and Hs-p48^RbAp48^ (Table S1). The NAP family is very large and displays a complicated phylogenetic history, and the *Physarum* genome only encodes two proteins from this family: PpSET (named after the closest human homolog HsSET) and PpNAP1L1 (naming based on its sequence similarity with HsNAP1L1) (Table S1). We did not find orthologues of the vertebrate CENP-A chaperone HJURP, the mammalian H2A.Z chaperone ANP32E and the yeast Chz1 and Rtt106 proteins. Finally, no orthologues were found in *Physarum* for several chaperones present in vertebrates and Arabidopsis (i.e., the H3/H4 chaperones DEK, TSK/TONSL, SPT2 and ATRX/DAXX; the NPM proteins involved in various processes) despite the presence of their histones.

### 2.2. Phylogenetic Study of the Putative Histone Chaperones in Physarum

In order to analyze conservation among members of each histone chaperone family, we generated phylogenetic trees of the *Physarum* histone chaperones and their orthologues from several eukaryotic model organisms. In the majority of trees, the animal orthologues cluster together as one branch, the plant orthologues form a second branch and the *Physarum* proteins cluster together with the orthologues from *D. discoideum* (Figure S2). This organization was found for HSP90 and HSC70 (Figure S2A,B), IPO4 and ASF1 and CAF1A (Figure S2D–F), HIRA (Figure S2I), CABIN1 (Figure S2K), SSRP1/Pob3 (Figure S2M), MCM2 and PolE3 and SPT6 and SWR1 and SWC2 (Figure S2O–S). For the other chaperones, the tree structure is similar, but with *Physarum*, *D. discoideum* and *T. thermophila* belonging to the same clade (NASP, Figure S2C; CAF1B and CAF1C, Figure S2G,H; SPT16, Figure S2N), and with yeast for the UBN orthologues (Figure S2J). Regarding APLF, only animals, *T. thermophila* and *Physarum* encode orthologues of this protein, although they are pretty divergent in these lineages (Figure S2P). To conclude, *Physarum* chaperones are related to their *D. discoideum* counterparts, as expected, and diverge from the other studied organisms.

### 2.3. Protein Sequence and Structure Conservation of the Physarum Histone Chaperones

We then investigated if the 21 histone chaperones that we identified in *Physarum* exhibit conserved features (i.e., protein domains and key residues known to be involved for histone-chaperone and/or chaperone-chaperone interactions) with those of three model species (*H. sapiens, S. cerevisiae* and *A. thaliana*). These three species were chosen based on the data availability for the studied chaperones. Our study analyzed each of the 21 chaperones identified in *Physarum* ordered according to their role in the cellular life of histones as defined in Figure 1, focusing first on H3/H4 chaperones.

#### 2.3.1. The H3/H4 Chaperones Involved before Chromatin Incorporation

**Figure 3:**
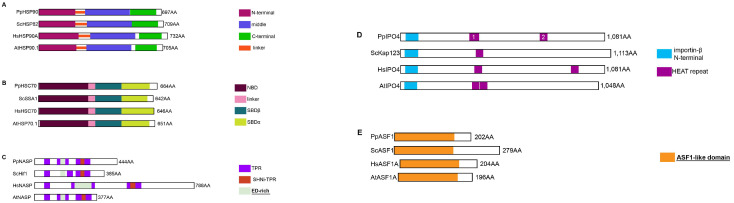
Functional Domains of histone chaperones involved in the cytoplasm. Each diagram displays a scaled representation of the domain structure for HSP90 (**A**), HSC70 (**B**), NASP (**C**), IPO4 (**D**), ASF1 (**E**) histone chaperones from *Physarum*, yeast, human and Arabidopsis. Each domain is depicted at its position by a different color, and the code is indicated at the right. Names of domains involved in histone binding are in bold and underlined. NBD, N-terminal Nucleotide Binding domain; SBD, Substrate-Binding Domain; TPR, tetratricopeptide-like bi-helical repeats; SHNi-TPR, Sim3-Hif1-NASP interrupted TPR; ED-rich, domain rich in glutamic acid and aspartic acid; HEAT, Huntingtin/Elongation factor 3/protein phosphatase 2A/TOR1.

The heat-shock proteins HSP90 and HSC70 promote histones H3 and H4 folding and heterodimerization in the cytoplasm [5]. **HSP90** proteins have several functional domains that are conserved in *Physarum* (Figure 3A and Figure S3). Like other HSC70s, **PpHSC70** contains the three conserved structural domains associated with this protein (Figure 3B and Figure S3). The histone chaperone **NASP** is a non-specific chaperone interacting with H1, H3 and H3/H4 as well as a CenH3 in Arabidopsis [22,23] and in S. pombe [24]. The PpNASP protein presents the characteristic four TPR involved in H1 and H3/H4 binding [25,26] and one SHNi-TPR (Sim3-Hif1-NASP interrupted TPR) characteristic of NASP proteins as well as a “E/D-rich” region (Figure 3C and Figure S4). The nuclear translocation protein **IPO4** is involved in the nuclear import of H3/H4 dimers [7]. PpIPO4 presents a conserved architecture (Figure 3D), as well as most of the residues involved in the interaction with H3 and H4 (Figure S5D,E). The **ASF1** chaperone is considered the histone donor for the replication-dependent and -independent pathways of histone H3/H4 incorporation into chromatin [27]. While the ASF1 N-termini are highly conserved (50–60% of identity for the first 155 amino acids, Figure S6), the ASF1 C-termini are rather divergent in size (Figure 3E) and sequence (Figure S6). Moreover, most residues involved in H3/H4 binding [27,28] are conserved in PpASF1 (Figure S6, in green) as well as those involved in chaperone-chaperone binding [9,29] (Figure S6 in blue). These various findings suggested a conserved H3/H4 network in *Physarum* for histone folding, maturation and nuclear import.

#### 2.3.2. The H3/H4 Chaperones Involved in Chromatin Incorporation

**Figure 4:**
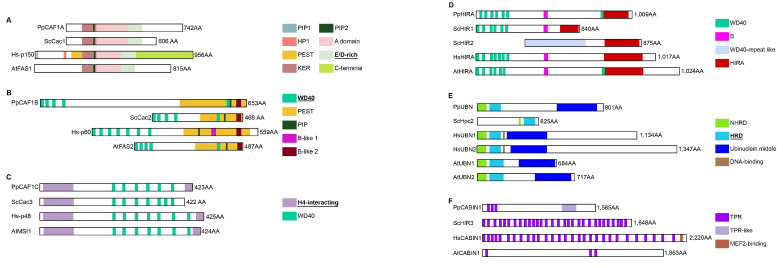
Functional Domains of histone chaperones involved in the H3/H4 incorporation in chromatin. Each diagram displays a scaled representation of the domain structure for CAF1A (**A**), CAF1B (**B**), CAF1C (**C**) HIRA (**D**), UBN (**E**), CABIN1 (**F**) histone chaperones from *Physarum*, yeast, human and Arabidopsis. Each domain is depicted at its position by a different color and the code is indicated at the right. Names of domains involved in histone binding are in bold and underlined. PIP, PCNA-Interacting Peptide; PEST, domain rich in proline (P), glutamic acid (E), serine (S) and threonine (T); ED-rich, domain rich in glutamic acid (E) and aspartic acid (D); KER, domain rich in lysine (K), glutamic acid (E) and arginine (R); NHRD, N-terminal to the HRD region domain; HRD, Hpc2-related domain; TPR, tetratricopeptide-like bi-helical repeats.

The **replication-associated** assembly complex **CAF-1**, ASF1 and the DNA replication machinery coordinately deposit H3/H4 in chromatin during replication. The *Physarum* CAF-1 heterotrimeric complex consists of PpCAF1A, PpCAF1B and PpCAF1C, which correspond to the large, middle and small subunits of the complex, respectively. Aside from the A domain flanked by the KER and “E/D-rich” domains, **CAF1A** structures differ drastically between human and other species, notably in their C-termini (Figure 4A). For instance, animal CAF1A proteins display two PCNA-interacting protein (PIP) motifs, whereas *Physarum* and other organisms only contains the second motif (PIP2) (Figure 4A [30]. **PpCAF1B** harbors domains that are characteristic of the middle CAF-1 subunit. However, while Hs-p60 contains clustered WD40 repeats in its N-terminal and two B-like domains, PpCAF1B and its yeast and Arabidopsis orthologues display one WD40 repeat in their C-termini and only one B-like domain (Figure 4B). **CAF1C** proteins also have several WD40 repeats located in the internal region (Figure 4C), and all residues involved in H4 interaction [31] are strictly conserved in PpCAF1C (Figure S7I,J). Thus, the CAF-1 complex is overall conserved in *P. polycephalum*, but is more similar in domain structure to the Arabidopsis orthologue. The **replication-independent** assembly complex **HIR** preferentially deposits H3.3/H4 in chromatin in a replication-independent manner [32]. The *Physarum* complex consists of PpHIRA, PpUBN and PpCABIN1. **PpHIRA** presents a similar domain organization to AtHIRA, with several WD40 repeats located in the N-terminal and an additional one just before the HIRA domain (Figure 4D). The GRRRIxPLxI motif (with x being any amino acid) involved in ASF1 interaction [9] (Figure S8B) and the HIRA domain involved in CABIN1 binding ([33]; Figure S8C) are strongly conserved in *Physarum*. **PpUBN** presents a domain organization most similar to Arabidopsis UBNs, with a C-terminal middle domain and comparable protein length (Figure 4E). The HRD domain of HsUBN1 provides a H3.3-binding specificity to the HIR complex [32]. Six out of seven key residues identified for their role in histone binding [32] are strictly conserved (Figure S8E, in green); for the non-conserved residue, there is a N/D substitution in plants, yeast, *T. thermophila, C. elegans* and myxomycetes. **PpCABIN1** presents a similar domain organization to AtCABIN1 with grouped in TPR islands, while TPRs are distributed on the whole yeast and human proteins (Figure 4F). Thus, the HIR complex is largely conserved in *P. polycephalum*, with a domain structure closer to plants. Therefore, we can conclude that the H3/H4 chaperones involved in chromatin incorporation are conserved in *Physarum* with a protein domain structure closer to Arabidopsis.

#### 2.3.3. The H2A/H2B Chaperones

**Figure 5:**
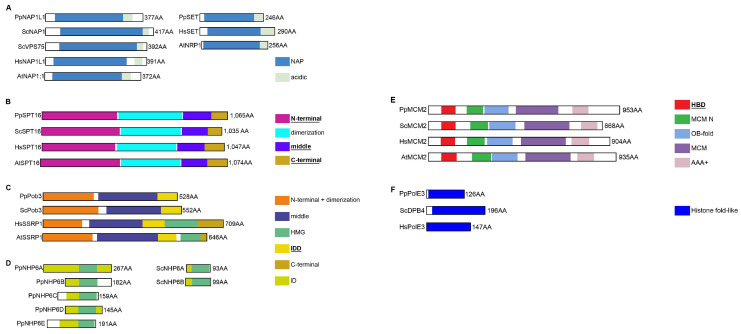
Functional Domains of H2A/H2B histone chaperones and replication-associated chaperones. Each diagram displays a scaled representation of the domain structure for NAP proteins SET and NAP1L1 (**A**), SPT16 (**B**), SSRP1/Pob3 (**C**), NHP6 (**D**), MCM2 (**E**), PolE3 (**F**) from *Physarum*, yeast, human and Arabidopsis. Each domain is depicted at its position by a different color and the code is indicated at the right. Names of domains involved in histone binding are in bold and underlined. HMG, High-Mobility Group; IDD and ID, Intrinsically DisorDered; HBD, histone binding domain; OB-fold, oligonucleotide/oligosaccharide-fold; AAA+, ATPase activity.

The identification of **NAP** proteins relies on the presence of the NAP central domain, which enabled us to identify PpSET and PpNAP1L1 (Figure 5A). NAP proteins are acidic proteins (~29% and 24% of D/E residues for PpSET and PpNAP1L1, respectively), the acidic domain being terminal (Figure 5A) for NAP proteins of groups C and D (Figure S2L). Two motifs are involved in histone binding (NAP1L and Cap-Claw-Anchor). Both were retrieved in PpNAP1L1, but PpSET only harbors the Cap-Claw-Anchor motif since the NAP1L motif is absent from SET proteins (Figure S9C,D). Histone-binding regions and key residues reported in yeast, *C. elegans* and Arabidopsis [34,35] are species-specific and, thus, only few residues are conserved in *Physarum*. Thus, we could speculate that PpNAP1L1 and PpSET are bona fide histone chaperones. The **FACT** complex is composed of two subunits SPT16 and SSRP1. **PpSPT16** presents well conserved domains (Figure 5B). Of the residues important for H3/H4 interaction [36], K692-ScSPT16 is only conserved in yeast and animals while R693-ScSPT16 is conserved in yeast, animals and slime molds (Figure S10C, in green). The SPT16 C-terminal domain is an intrinsically disordered acidic region containing (~47% of acid residues in ScSPT16 and ~33% in PpSPT16). Most **SSRP1** proteins display 5 structural domains but ScPob3 and PpPob3 (Figure 5C) and TtSSRP1 and DdSSRP1 [37] present only three: the HMG domain is absent from these four proteins. ScPob3 forms a complex with ScSPT16 and ScNHP6 which provides the HMG-box function [38]. We identified 5 putative NHP6 orthologues in *Physarum* (PpNHP6A-E, Figure 5D) which might provide the HMG box function. Besides, the H2B-binding motif D/Exxϑ (where ϑ is F or Y, and x is any residue; [39]) is strongly conserved in PpSPT16 (Figure S11C, in green). Hence, our data strongly suggests that the *Physarum* FACT complex is related to the yeast one.

#### 2.3.4. The DNA Replication-Associated Histone Chaperones

**MCM2** can associate with H3/H4-ASF1 [12], as well as with histone-FACT during replication [40] and with all H3 isoforms (H3.1, H3.3 and CENP-A) [41]. Indeed, PpMCM2 presents a HBD (Histone Binding domain; Figure 5E) [12,40]. Key residues for H3/H4 binding (Figure S12A, in green) and association with the histone-FACT complex [40] (Figure S12A, purple asterisk) are conserved in all analyzed species including *Physarum*, except M117-HsMCM2 which is restricted to the animal kingdom (Figure S12A, in orange). The DNA polymerase **PolE** is composed of four subunits in mammals, and is responsible for DNA synthesis on the leading strand during replication. PolE3 and PolE4 are the small subunits of this complex [42] and are H3/H4 chaperones. We found a *bona fide Physarum* orthologue only for PolE3 (Figure 5F) but none for PolE4, suggesting that PolE might comprise only three components in *Physarum*. Functional domains of PolE3 are conserved in PpPolE3, but the αC helix characteristic of the H2B family is much shorter in PpPolE3 (Figure S13B). Thus, functions of *Physarum* MCM2 and PolE3 histone chaperones might be similar to those of animals, plants and yeast.

#### 2.3.5. The Chaperones Associated with Histone Recycling and Exchange

**Figure 6:**
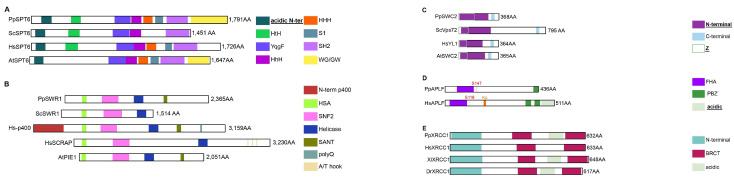
Functional Domains of histone chaperones involved in transcription, replacement and DNA repair. Each diagram displays a scaled representation of the domain structure for SPT6 (**A**), SWR1 (**B**), SWC2 (**C**), APLF (**D**), histone chaperones from *Physarum*, yeast, human and Arabidopsis. Diagrams for XRCC1 (**E**) display a scaled representation of this protein from *Physarum*, yeast, human, Xenopus, *D. rerio* and Arabidopsis. Human possesses two SWR1 orthologues named Hs-p400 and HsSCRAP. Each domain is depicted at its position by a different color and the code is indicated at the right. Names of domains involved in histone binding are in bold and underlined. HtH, Helix-turn-Helix; HhH, Helix-hairpin-Helix; HHH, HHH domain 9; S1, S1 RNA-binding domain; SH2, Src-homology 2 domain; WG/GW, domain containing Glycin (G) and Tryptophan (W) repeats; HSA, Helicase/SANT-associated; SANT, Swi3/Ada2/N-Cor/TFIIIB; polyQ, poly-glutamine domain; FHA, ForkHead-Associated; PBZ, PolyADP-ribose-Binding Zinc-finger; BRCT, BRCA1 C-terminal.

**SPT6** is associated with the recycling of modified histones during transcription [43]. While PpSPT6 exhibits a conserved structure for the core region (Figure 6A), it also contains the plant specific WG/GW domain (Figure 6A and Figure S14A). Moreover, the F249-ScSPT6 residue involved in nucleosome binding [44] is strongly conserved in PpSPT6 (Figure S14B). Hence, based on the presence of the WG/GW domain, PpSTP6 is more similar to plant proteins than to their animal homologs. During chromatin remodeling, the H2A.Z/H2B variant exchange is performed by the **SWR-C** complex [14,15,16,17,18], a multicomponent complex with two proteins that have histone chaperone activities: the SWR1 catalytic subunit and the SWC2 (aka Vps72 or YL1) accessory subunit. *Physarum* encodes one **SWR1** orthologue, while some organisms such as human, mouse, fish and Xenopus have two (Figure 6B); human orthologues are Hs-p400 and HsSCRAP. The ScSWR1 N-terminal region is responsible for H2A.Z binding [45] but key residues [46] are not conserved across studied species (Figure S15D). Besides, some SWR1 orthologues have a SANT domain, such as PpSWR1, Hs-p400 and AtPIE1 (Figure 6B and Figure S15D). **PpSWC2** is the *Physarum* orthologue of ScSWC2 and animal YL1 proteins. SWC2 proteins harbor a widely conserved Z domain (Figure 6C) responsible for H2A.Z binding and selectivity. The DmYL1 key residues [47] are widely conserved (Figure S15E; in blue) as well as the RxxR motif (x for any residue, Figure S15E; in pink) that anchors ScSWC2 to the nucleosome surface [48]. Hence, the SWR-C complex is similar to those of animals, plants and yeast. **APLF** is a DNA-damage response protein that chaperones histones at DNA damage sites. While the plant kingdom and several studied organisms such as yeast do not encode an APLF orthologue, we retrieved one in *P. polycephalum*. However, mammalian APLF proteins exhibit tandem PBZ domains while the other APLFs, including PpAPLF, display a single PBZ located at the very end of the protein (Figure 6D). Hence, only mammalian proteins have an acidic tail (Figure 6D), responsible for interaction with core histones [49]. For slime mold, it was reported that the acidic tail is integrated in XRCC1 [49]. Indeed, we found in PpXRCC1 an acidic domain similarly to XlXRCC1 and DrXRCC1 (Figure 6E). Moreover, these acidic domains display the NAP1L and the H2A-H2B binding cap-anchor motifs as well as the KR-motif (KR, lysine/arginine) [49,50]. (Figure S16B). Since the acidic domain required for histone binding is integrated in PpXRRC1, *Physarum* may display a mechanism in which APLF and XRRC1 cooperate to chaperone histones at DNA damage sites and trigger repair similar to Xenopus and zebrafish.

Based on the above detailed analyses, we could state that most chaperones involved in histone cellular life are present in *Physarum*, with some proteins closer to yeast (PpPob3), animals (PpAPLF) or plants (PpNASP, PpCAF-1, PpHIR, PpUBN, PpSTP6). Therefore, players of the histone chaperone network are conserved in *Physarum*, suggesting that these proteins were present in the last common unicellular eukaryotes, after which some underwent duplication and gave rise to multiple protein families, such as NAP.

### 2.4. Analysis of Gene Expression for Histone Chaperones during the Cell Cycle Reveals Two Main Expression Patterns

**Figure 7:**
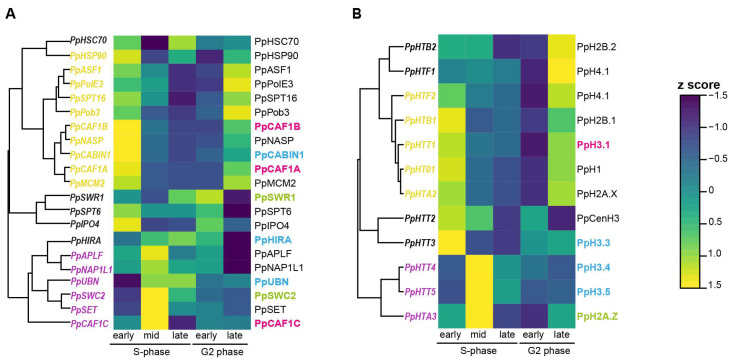
Expression profiles of *Physarum* histones and chaperones during the cell cycle. The heat map displays the RNA-Seq expression of histone chaperones (**A**) and histones (**B**) at five stages of the *Physarum* cell cycle indicated at the bottom of the map. Each row corresponds to a transcript listed on the right and each column to a cell cycle stage. The color bar at the right depicts the scale for z-score, with blue representing the lowest expression and yellow representing the highest. In Figure 7A, chaperones from group 1, i.e., with elevated mRNA levels in early S-phase and late G2 phase are displayed in yellow on the left of the heat map, while chaperones from group 2, i.e., with an elevated mRNA level in mid S-phase are displayed in magenta. Transcript and protein names are indicated on the left and the right of the heat map, respectively. The CAF-1 complex is associated with H3.1/H4 incorporation during DNA replication, and its subunits are displayed in pink (**A**) as well as the *PpHTT1* transcript coding the *Physarum* H3.1 protein (**B**). The HIR complex is associated with H3 variant incorporation throughout the cell cycle and its subunits are displayed in blue (**A**), as well as the *PpHTT3/4/5* transcript coding the *Physarum* H3 variants (**B**). The SWR-C complex is involved in H2A.Z/H2B replacement and its subunits are displayed in green (**A**), as well as the PpHTA3 transcript coding the Physarum H2A.Z variant (**B**).

While histone gene expression and regulation during the cell cycle has been extensively studied in various organisms [51,52], little is known about their chaperones. We thus monitored the expression of the 21 chaperone-coding and the 12 expressed histone-coding genes in *Physarum* synchronous plasmodia at specific time points during the cell cycle by RNA-Seq. The nuclei of *Physarum* plasmodia are naturally synchronized, providing us with the opportunity to quantify chaperone transcript levels at specific stages of the cell cycle. *Physarum* cell cycle consists of a 0.5 h mitosis, a 3 h S-phase and a 6 h G2-phase with no G1-phase [53]. We observed two main expression patterns for chaperones: (i) group 1 (*PpHSP90, PpNASP, PpASF1, PpCAF1A, PpCAF1B, PpCABIN1, PpMCM2, PpPolE3, PpSPT16, PpPob3*: in yellow on the left) with elevated mRNA levels in early S-phase and late G2 phase, (ii) group 2 (*PpCAF1C, PpUBN, PpSWC2, PpSET, PpNAP1L1, PpAPLF*: in magenta on the left) with elevated mRNA levels in mid S-phase (Figure 7A). In group 1, we found chaperones known to be involved in H3/H4 nuclear import (HSP90, NASP, ASF1), in the H3/H4 replication-dependent handling (CAF1A, CAF1B, MCM2, PolE3) and the FACT complex notably implicated in histone handling during replication. In group 2, we found chaperones involved in more various processes and that can handle canonical histones and variants. For the CAF-1 complex that incorporates H3.1/H4 during replication in various organisms, we observed a correlation between *PpCAF1A, PpCAF1B* and *PpHTT1* (*PpHTT1* coding the canonical histone PpH3.1) mRNA levels (Figure 7A,B), CAF1C being involved in various processes and complexes. On the contrary, *PpHIRA* and *PpUBN* mRNA levels are higher during the mid and late S-phase than in early S-phase or late G2 phase (CABIN1 being involved in various processes) as well as those coding the PpH3.4 and PpH3.5 variants, suggesting that *Physarum* HIR complex may incorporate PpH3.4 and PpH3.5. Therefore, chaperones of group 1 may handle the canonical histone pool highly abundant in early S-phase and late G2 phase, while chaperones of group 2 may handle the less abundant histone pool during the rest of the S-phase. We also observed a good correlation between the *PpHTA3* (coding PpH2A.Z) and *PpSWC2* mRNA levels (Figure 7A,B), PpSWC2 being part of the complex responsible for H2A.Z replacement. Finally, we noticed that three chaperones display highly abundant mRNAs compared to the other ones: the two heat shock chaperones PpHSP90 and PpHSC70, and the PpNAP1L1 chaperone (Table S7). This might be due to the involvement of PpHSP90 and PpHSC70 in folding of various other client proteins, and of PpNA1L1 in the incorporation of both H2A/H2B and H3/H4. To conclude, the abundance of histone transcripts and their associated chaperones correlates in *Physarum*, suggesting that histone and chaperone abundance are under the same control.

## 3. Discussion

We performed a comprehensive analysis of the histone chaperones in *Physarum* and found that most histone chaperones are conserved in this slime mold. Based on comparative analyses, we propose that histone folding, transport to the nucleus, supply, turnover and incorporation in chromatin are cellular functions that are expected to be executed by the histone chaperone network of *Physarum*. While histone chaperones in *Physarum* display the common feature of histone binding, they do not share extensive sequence similarity or structural domains [54] compared to orthologues from other eukaryotic clades. However, since histones are highly basic proteins, many histone chaperones contain acidic stretches such as E/D-rich regions to shield the histone charge and avoid aggregation and spurious interactions. IDD regions are flexible and highly dynamic; they may be critical for chromatin assembly and were reported in human histone chaperones [54]. We used RAPID (Regression-based Accurate Prediction of Protein Intrinsic Disorder content) [55] to quantify the disorder content of the 21 uncovered *Physarum* histone chaperones, and found that 11 chaperones present a disorder content above 30% (Table S1). Therefore, histone chaperones in *Physarum* contain acidic stretches and IDD regions that are shared features between and with chaperones from well-studied organisms.

Our study highlights the conservation of the histone chaperone domains and key residues (for histone or chaperone binding) in *Physarum* compared to common model organisms. Most histone chaperones of *Physarum* present conserved domains and key residues (for histone or chaperone binding) compared to studied organisms. However, we observed that *Physarum* chaperones are phylogenetically divergent from plant and animal orthologues. Nevertheless, it is interesting to note that the domain structure of (i) the CAF-1 proteins (one PIP in PpCAF1A, one C-terminal WD40 repeat and one B-like domain in PpCAF1B) and (ii) the HIR proteins (the extra WD40 just before the HIRA domain of PpHIRA, a C-terminal ubinuclein middle domain in PpUBN and the three TPR in PpCABIN1) are closer to the Arabidopsis orthologues. Similarly, the *Physarum* orthologue of SPT6 contains a WG/GW domain, as in plants. In contrast, the structure of the FACT complex (the HMG domain being absent from PpPob3) is more closely related to the yeast complex, and the animal-specific chaperone APFL is present in *Physarum*. Interestingly, unlike *Physarum*, APLF is absent from plants, yeast, some animals such as *C. elegans* and Drosophila and also another Mycetozoan (*D. discoideum*). This suggests that the APLF chaperone might have been present in the last common eukaryotic ancestor, and was subsequently lost in certain lineages or species. Moreover, while the mammalian APLF protein presents an acidic tail, this domain is translocated to XRCC1 in other species [49]. As this tail is responsible for the interaction of APLF with histones, PpAPLF and PpXRCC1 might cooperate to ensure histone incorporation at DNA damage sites and to restore the epigenomic landscape. In SPT6, we found five WG/GW repeats in PpSPT6, whereas PpaSPT6, AtrSPT6, AtSPT6L, ZmSPT6 have 8, 20, 12 and 8 WG/GW repeats, respectively (Figure S14A). These WG/GW repeats were not originally identified in *P. patens* [56], most likely due to the incomplete sequences of SPT6 orthologues at that time (the XP_00175668 and XP _00296188 accession numbers used for P. patens in that study are not available anymore). Interestingly, the WG/GW domain is also called the Argonaute hook, since it is required for Argonaute interaction with other proteins to mediate small RNA-mediated gene silencing in Arabidopsis [56]. Thus, it will be interesting to assess if PpSTP6 can also interact with the *Physarum* Argonaute proteins. Finally, *Physarum* does not have homologues for the ATRX/DAXX complex. While mammals, zebrafish and Drosophila express an ATRX/DAXX complex, Arabidopsis and *C. elegans* only have ATRX but no identifiable DAXX homolog [57,58]. This suggests that: (i) Arabidopsis and worm ATRX might interact with a DAXX functional analog or with other yet unknown partners to trigger H3.3 deposition, while (ii) *Physarum* might perform H3.3 deposition only through the HIRA complex. Yeasts are widely used models to study the maintenance of the epigenetic landscape during replication [59]. Indeed, they can be synchronized and have a short generation time and only few histone-coding genes compared to animals [60,61]. However, they display neither H3 variants nor DNA methylation, while *Physarum* does [20,62,63]. Moreover, *Physarum* has a short generation time, naturally synchronous nuclei, histones coded by one single gene which enable easy mutagenic analyses, and an ability to incorporate exogenous tagged histones [64]. With its unique features and shared similarities with yeast, plants and animals, *Physarum* represents a pertinent model for epigenetic studies.

In this study, we took advantage of the natural synchrony of *Physarum* plasmodia to quantify transcript abundance for the 21 chaperones as well as for the histones. Most chaperones showed elevated transcript levels during early S-phase and late G2-phase. Our previous study in *Physarum* [20] demonstrated a unique pattern of histone gene expression, with elevated histone mRNA abundance in late G2 phase and at the beginning of the S-phase. Thus, it is not surprising that most *Physarum* histone chaperones also present higher transcript levels in late G2 phase to match this abundant pool of synthesized histones. Moreover, *PpCAF1A* and *PpCAF1B* presented elevated transcript levels in early S-phase, which is consistent with known roles of CAF-1 in H3.1/H4 incorporation at replication forks. In contrast, *PpHIRA* and *PpUBN* had higher mRNA levels during the mid and late S-phase, consistent with known roles of HIR in variant incorporation throughout the cell cycle. Finally, PpHSP90, PpHSC70 and PpNAP1L1 chaperones had elevated transcript levels compared to the other chaperones. The two heat shock proteins (PpHSP90 and PpHSC70) have various binding partners besides the histones. Thus, they should be present at sufficient levels to mediate widespread protein folding in *Physarum*. Regarding PpNAP1L1, we can speculate that since NAP proteins are responsible for the incorporation of both H2A/H2B and H3/H4, and contribute to histone shuttling and chromatin assembly during replication and transcription, they are required to be present at higher levels compared to other chaperones. Furthermore, we found a clear dichotomy in expression profile between chaperones associated with replication (notably PpMCM2, PpPolE3, PpCAF1A and PpCAF1B) and those that are replication-independent (notably PpHIRA and PpUBN) (Figure 7A). We investigated whether this pattern could be observed in synchronized cells of other organisms, but we did not observe such changes in the three human cell lines investigated (Figure S17C–E) or in yeast cells (Figure S17G). This result is consistent with the fact that several chaperones are known to control histone gene expression in yeast during the cell cycle [65]. However, cells of *Nicotiana tabacum* presented a pattern similar to *Physarum*, though with the SWR-C clustering with replication-associated chaperones (Figure S17F). Therefore, it is tempting to speculate that in *Physarum*, the abundance of histones during the cell cycle controls the expression of their chaperones (and subsequently nucleosome assembly), resulting in a different expression pattern during replication-dependent and -independent pathways. This feature may be specific to slime molds.

In summary, our work on the histone chaperones of *Physarum* contributes to a better understanding of the conservation of these proteins through evolution. Because of the strong conservation of histones in eukaryotes, many organisms present similar and conserved mechanisms for histone handling using chaperones. The characterization of *Physarum* histones and their chaperones ([20] and this study) and the use of *Physarum* giant cells containing millions of synchronous nuclei open new avenues for the analysis of the epigenetic landscape and its maintenance during the cell cycle in this organism that shares features with both animals and plants.

## 4. Materials and Methods

### 4.1. Identification of Physarum Genes, Transcripts and Proteins for Chaperones

Public genomic and transcriptomic data from *P. polycephalum* were obtained from www.physarum-blast.ovgu.de and former published data [21,66]. Protein sequences from *H. sapiens, M. musculus, D. rerio, D. melanogaster, X. laevis, C. elegans, A. trichopoda, A. thaliana, Z. mays*, *P. patens, T. thermophila, D. discoideum* and *S. cerevisiae* were obtained from UniProt [67] and/or NCBI [68]. In the absence of proteins in both databases, a BLASTp with the human protein against the proteome of the organism for which no protein was recorded was performed. For APLF orthologues, one APLF protein was recorded in UniProt (NP_001097801.1) for *D. melanogaster* but it does not contain the characteristic FHA (ForkHead-Associated) domain. Besides, in *C. elegans*, the CeLig3 protein is only an APLF functional analog [49]. Thus, both proteins were excluded from our study. For the DrUBN1 protein, only a fragment of the protein is available in the databases. For UBN and CABIN1 in *C. elegans*, no orthologues were retrieved in UniProt or by BLASTp. CePICD-1 (pry-1 interacting CABIN1 domain containing) was found in the literature as an orthologue of CABIN1 [69]. We did not identify CePQN-80 based on UBN protein sequence homology, but it was added in the phylogenetic analysis based on a recent study [70]. However, our analysis showed that CePQN-80 is highly divergent from the other UBNs (Figure S2I). For the NAP family, proteins retrieved for a specific organism were cleaned from proteins identical at 92-99% on the whole protein. The Arabidopsis orthologue of PolE3 has not been faithfully identified yet [71] as well as orthologues for *P. patens, T. thermophila, Z. mays*, so these species were not included in the phylogenetic analysis. A local BLASTn (blast 2.6.0) was performed to identify chaperone homologs in the public *P. polycephalum* transcriptomes [21,66]. Identified transcripts were then aligned on the *Physarum* reference genome [21] to identify the corresponding genes. A tBLASTn search was also performed on the *Physarum* reference genome to investigate if the chaperones with no homologs in the *Physarum* transcriptomes corresponded to not expressed genes. No such case was found. All used protein sequences were reported in Table S2 and are available at https://clipperton.ufip.univ-nantes.fr/physabase/, accessed on 22 September 2022. Transcripts found to code histone chaperones were reported in Supplementary Material. More specifically, we did not find homologues either for HJURP (Holliday Junction Recognition Protein) which is a vertebrate chaperone required for CENP-A centromeric deposition [24] or for its yeast counterpart Scm3 (Suppressor of chromosome mis-segregation 3). No homologues were retrieved for the ATRX/DAXX [72] complex and for the DEK chaperone [73], both being involved in H3.3 deposition. Indeed, although Phypoly_transcript_06998 was found to encode a putative DEK protein, the encoded protein does not harbor the DEK characteristic domain, which led us to conclude that *Physarum* does not have a DEK orthologue. The H3/H4 chaperone TONSL (TONSuku Like) firstly identified in Arabidopsis (TONSuku, TSK; [74]) and later in various vertebrates, the mammalian ANP32E (Acidic Nuclear Phosphoprotein 32 kilodalton E; [75]) and the yeast Chz1 [76] both being H2A.Z chaperones, the yeast Rtt106 (Regulator of Ty 1 transposition 106) protein involved in replication-coupled H3/H4 incorporation [77], and the SPT2 protein that chaperones H3/H4 during transcription [78] did not present a homologue in P. polycephalum. Finally, the NPM proteins (NPM1, NucleoPhosMin; NPM2 and NPM3, NucleoPlasMin 2 and 3) involved in various processes such as chromatin remodeling or ribosome biogenesis were found in vertebrates but not in yeast [79] and *Physarum*.

### 4.2. Phylogenetic Analyses and Protein Sequence Alignments

The chaperone protein sequences were aligned with the Clustal Omega program [80]. Phylogenetic trees were constructed with Mega [81] and the ITOL (Interactive Tree Of Life) tool [82]. Plant, animal and *Physarum* proteins were depicted in green, blue and red in trees, respectively. Proteins from yeast, *D. discoideum* and *T. thermophila* were displayed in black. Using the number of proteins per chaperones families, we clustered the species using hierarchical clustering (R core team, www.R-project.org, accessed on 23 May 2022). The Phyre2 [83] and AlphaFold [84,85] web portals were used to create the 3D protein modelling and the Chimera software [86] for superimposition. Pairwise comparison of protein sequence identity from *Physarum* and the other organisms was performed by performing a NeedleMan-Wunsch global alignment, a needle command in the EMBOSS suite [87], of each *Physarum* protein sequence to all its orthologues in the studied organisms (Table S3). The chaperone protein domains in *S. cerevisiae, A. thaliana, H. sapiens* and *P. polycephalum* were determined from InterProScan (5.55–88.0, [88]) in standalone.

### 4.3. Identification of Nuclear Export and Localization Signals

The following tools were respectively used to predict the presence of NES (Nuclear Export Signal) and NLS (Nuclear Localization Signal): http://ehubio.ehu.eus/wregex/home.xhtml and https://www.novoprolabs.com/tools/nls-signal-prediction, accessed on 6 May 2022 and presence/absence of NES and NLS are listed in Table S5.

### 4.4. Physarum Material

*P. polycephalum* strain TU291 was used for this study. Mitosis was monitored on mitotically synchronous plasmodia by phase contrast microscopy observations [89] to further harvest synchronous plasmodium fragments at the chosen cell cycle stages.

### 4.5. Experimental RNA Analysis Procedures

Three mitotically synchronous plasmodia were prepared [89] and harvested, as described in [20]. Fragments of mitotically synchronous plasmodia were harvested ~10 min before mitosis 2 (late G2 phase), 2 min after mitosis (beginning of S-phase), 1 h after mitosis (mid S-phase), 2.5 h after mitosis (late S-phase) and 5.5 h after mitosis (beginning of G2 phase). Polyadenylated-enriched RNA samples and cDNAs were prepared as described in [20]. Quantitative PCR was performed with the SyberGreen qPCR master mix kit (Thermo Fisher Scientific, Scoresby, VIC, Australia) on a Biorad Cycler (Bio-Rad, Hercules, CA, USA). Relative transcript levels for each chaperone were calculated as follows: 106 × E^−Ct[chaperone gene]^/E^−Ct[19S]^. RT-qPCR histograms presented in Figure S17A,B show means of transcript levels ± SE obtained for two independent PCR amplifications of three biological replicates. Primers used in this study are designed with https://www.ncbi.nlm.nih.gov/tools/primer-blast/, accessed on 12 April 2022 and listed in Table S4.

### 4.6. RNA-Seq Library Construction and Sequencing

At 3 stages during S-phase (2 min after mitosis, early S-phase; 1 h after mitosis, mid S-phase; 2.5 h after mitosis, late S-phase) and 2 stages during G2 phase (5.5 h after mitosis, early G2-phase; ∼10 min before mitosis, late G2-phase), RNAs were isolated in triplicates from 3 mitotically synchronous plasmodia, as described in [20] and then treated with DNase I (NEB, New England Biolabs, Inc., Ipswich, MA, USA) and purified with phenol-chloroform extraction. Each replicate from the 5 cell cycle stages was subjected to RNA-seq library preparation using the NEBNext^®^ Ultra™ II Directional RNA Library preparation kit (NEB) with NEBNext^®^ Multiplex Oligos for Illumina^®^ (Dual Index Primers Set 1, NEB) following the manufacturer’s protocol. All 15 libraries were pooled together and run on one single lane of an Illumina NovaSeq6000 for paired-end sequencing (GEO accession number PRJNA894126), using a paired-end read length of 2 × 150 bp. List of files were recapitulated in Table S8. Besides the analysis of gene expression during the cell cycle, assembled transcripts were used to complete transcript sequences of PpHSP90, PpIPO4, PpCAF1B and PpNASP.

### 4.7. Assembly, Quantification and Analysis of RNA-Seq Data

After sequencing of *Physarum* samples, read quality was evaluated by checking the number of expected sequences, the GC percentage, the presence of adaptors and the overexpressed sequences using FastQC [90]. Contamination was checked by aligning reads against E. coli, Yeast, and PhiX Illumina control genomes. Assembly was performed with Trinity [91] and quantification with Salmon [92] to generate expression estimation of the read count (Transcripts Per Million transcripts, TPM). From means generated from normalized read count (in CPM, count per million), heat maps were generated with RStudio (version 2022.02.1) with the package “pheatmap” (Version 1.0.12).

### 4.8. Processing and Analysis of Public RNA-Seq Data

Data from human (HUVEC cells, Human Umbilical Vein Endothelial Cells, GSE211658; MCF-7 cells, a breast cancer cell line, GSE94479; U2OS cells, a cell line with epithelial morphology derived from a tibia sarcoma, GSE143275), tobacco TN-90 cells (GSE121032) and yeast (GSE168699) were downloaded from NCBI. Reads were filtered and trimmed using fastp (version 0.21.0 with length_required 20 and average_qual 20) [93]. Data sets were aligned against the human (Hg38) or tobacco TN-90 (Nitab-v4.5) or yeast (S288C R64) genomes using STAR (version 2.7.2a with “out Filter Mismatch Nmax” = 2, “align Intron Max” = 15,000, “align Mates Gap Max” = 15,000, “out Filter Multimap Nmax” = 100, “win Anchor Multimap Nmax” = 100) [94]. FeatureCounts (version 1.6.4, parameters: -M -C -O) [95] was used to count the reads/fragments over gene annotation. Then TPM was computed for each sample, followed by calculation of z-score for the genes of interest (Table S6).

## Data Availability

All data are included in this article.

## Supplementary Materials

The following supporting information can be downloaded at: https://www.mdpi.com/article/10.3390/ijms24021051/s1. References [56,96,97,98,99,100,101,102,103,104,105,106,107,108,109,110,111,112,113,114,115,116,117,118,119,120,121,122,123,124,125,126,127,128,129,130,131,132,133,134,135,136,137,138,139,140,141] are cited only in the Supplementary Material.

## Abbreviations

APLF, Aprataxin-PNK-Like Factor (PNK, polynucleotide kinase); ASF1, Anti-Silencing Factor 1; ATRX, Alpha Thalassemia-mental Retardation X-linked syndrome; CAF-1, Chromatin Assembly Factor-1; DAXX, Death-domain Associated protein; FACT, FAcilitates Chromatin Transcription; HAT1, Histone Acetyl Transferase 1; HIR, HIstone Regulator; HSC70, Heat Shock Cognate 70; HSP90, 90-KDa Heat Shock Protein; IPO4, ImPortin 4; MCM2, Mini-Chromosome Maintenance; NAP, Nucleosome Assembly Protein; NASP, Nuclear Autoantigenic Sperm Protein; p46RbAp46, RetinoBlastoma Associated Protein 46; PCNA, Proliferating Cell Nuclear Antigen; Pob3, Pol1-binding protein 3; SPT6, SuPpressor of Ty 6; SPT16, SuPpressor of Ty 16; SSRP1, Structure-Specific Recognition Protein 1; SWR1, SWi2/snf2-Related 1; SWR-C, SWR1 Remodeling-Complex; TSK, TonSuKu; UBN, UBinucleiN.
