# Supplementary material for: The Histone Chaperone Network Is Highly Conserved in Physarum polycephalum
Source: Int J Mol Sci. 2023 Jan 5;24(2):1051. doi: 10.3390/ijms24021051 (PMC9864664; doi:10.3390/ijms24021051)
Supplement: Supplementary file 1 [file ijms-24-01051-s001.zip › ijms-2132472-supplementary.pdf]

Supplementary Material

The Histone Chaperone Network is highly conserved in *Physarum polycephalum*

Axel Poulet[1*], Ellyn Rousselot[2*], Stéphane Téletchéa[2], Céline Noirot[3], Yannick Jacob[1], Josien van Wolfswinkel[1], Christophe Thiriet[4] and Céline Duc[2#]

[1]Department of Molecular, Cellular and Developmental Biology, Faculty of Arts and Sciences, Yale University, New Haven, CT, 06511, USA

[2]Nantes Université, Faculté des Sciences et Techniques, CNRS, US2B, UMR 6286, F-44000 Nantes, France

[3]INRAE, UR 875 Unité de Mathématique et Informatique Appliquées, Genotoul Bioinfo Auzeville, 31326 Castanet-Tolosan, France.

[4]Université Rennes 1, CNRS, IGDR (Institut de génétique et développement de Rennes) - UMR 6290, 35043 Rennes, France

[*]Contributed equally.

[#] Corresponding author : celine.duc@univ-nantes.fr

1. Analysis of chaperone transcripts

For each chaperone transcript, we provided below the sequence. The transcript name corresponds to the transcript ID followed by the protein ID. The coding sequence is displayed in upper case in bold italics while 5′UTR and 3′UTR are in lower case.

> Phypoly_transcript_17984_PpASF1
tgtcgtgttcagtgagg***ATGGGTTGTGTCAACGTCACTAACATCCAGATACTGGATAATCCAGCCCCTTTCATGA***
ATCCCTTGCAATTTGAAATCACGTACGAGTGCATCACAGACTTACAAGAAGATCTTGAGTGGAAGGTTGTGTATG
TTGGAAGCGCAGAAAGTGAAGCACATGACCAAGTGTTGGATTCTATTCTTTTGGGACCTGTACGTGTAGGAGTGT
CAAATTTTGTTTTACAAGTTGACCCTCCAGATTCTTCTAGAATTCCAAAACAAGACTTACTGGGGGTTACTGCGA
TATTACTAATGGGACTGTACAAATCACGGGACTTTATACGGGTGGGCTATTTTGTGAACAACGAGTATGCGGAAG
AGGAGCTAAGACTCAATCCTCCAGAGGGAGAGGTAGACTTAACAAAAGTGGTACGAAATATACTCGCAGAAAAGC
CCCGCGTAACCCGTTTCCCCATCCCATGGGACCAAGAAACCATCATTCCCCCTGCAAACACAGAAAATTCCTCCT
CCCTCCCCGCGATCACAACCGAAACCAGCCTTGATGCCGCGTCAAACCACAGCATGACGGAAGAGATGATGCCTA
TGGAAGTCATGTCTACTTCAACTTAAgcggtcaaaattgtaaaataccataaggttgaagcacaagagctgggta
gaacttgttaaaattaccaaaatacgaaaatacgcgaaaaaattataggcggatttggcctagaaaactggataa
actagttatccgtgcatttctaggttttgacgaaa

>Phypoly_transcript_01999_PpMCM2
ttgcgcacgcaaagaattggcgccaaacctacacccacaagacctgccatacaaaacatcccccctacttccctac
caacacaaaccaccttcccatcctactcagtc***ATGAGTGATACCAACAGCAACCACAACTCTGACGAGGAGGGCG***
AGGAGGAGTATGACCCCTCCATCCCGAAGGACAAGGAGGCAGAGGAGGATGTGGAGTCCGAGACCCTGTCGGATG
GCAACCAGGAGGCTATGGATGATATTTTGGATGGTGAGGAGAATGTAGTTTATGACCCTGAAGAATTGGAGGCTA
TCAACCAGGGCGAGGATTTGTACAATGGTGATTTCGAACAGGATTACGAGTATGACCCCAACTTGGATCGATATG
ATGAAAAAGATTTGGATGAAAACGAATATGCTGAAATGGGTCCAGAGGAACGAGCAGCAGCAGAAGAAGCTATTA
AGAAACGGAATCGTAAAGTGCAAAAAGCAAAAACTGGAAGACTTCCTAGTGCTCTACTCTATGATACCGACGAAG
AAGAAGATGCCAAGCCCGAATACAAACCGAGGAGGAAAAGGCCACGAGTCAACGACCCTAATGTTGGAAATGTGT
TTGTTGAAGAAGAGGATACAAGAGATTTGGGTGATTTGGATGTTAATTTGGAAGATGTAAAAGGTCCCTTGCGCG
AATGGATTAGCTTAGATCCCACAAAGAGGACAATTATGCGTCGATTCAGGAAATTCTTAAAAACATATGGTCCTG
CCAAAGCCCCTTTTTATCAACAGGCCATTCAAACAATGTGTGCGGCAAATCAAGAAAGTTTGTTGGTGAACCACA
ATCACCTGACACAGACGGAGCCCTTGATAGCCATTTGGACGGCCGACGCGCCTACTGAAATGATCCAAATTTTTG
ATGAGGTTGCCATGAAACTCGTTTTGGAAATGTTTCCCAATTACGAAAACATATGTCCAGAAGTACACGTTCGTA
TAACCAACTTGCCGGTGAGTGATTCGCTGCGCGAGTTGCGTCAAATTCATTTAAATTGTTTGGTAAAGGTTTCTG
GTGTGGTCACTCGCAGAACATCCGTTTTCCCTCAACTTAAATATGTAAAATTCGACTGCTTGAAATGTTCAACGA
TCCTTGGGCCATATTACCAGGACGGACAAGCGGAGATCAAGGTCGGAATATGCCCCAATTGTCAAAGTAAGGGCC
CGTTCGCCATCAATGCGGAGCAAACTGTGTATCAATGCAAAAAAAAAAAAAAAAAAAAAAAAAAAAAAAAAAAAA
AAAAAAAAAAAAAAAAAAAAAAGATCAAAAGAGGTGATACTTTTGTGGACCTAATCGACAGCGCGCGTCCTGGAG
AGGAAGTAGAAGTAACAGGCATTTACCGCCACAACTTCGACGAGCACTTGAACAAAACACACGGATTTCCGGTTT
TCGCGACTATCATCGAGGCAAATTATATATCCAAAAAAGACGACTTGCTAGCGTCGTTCCGCCTTACTGAGGCCG
ATGAGAGAGAGATTAGGCGACTCGCCAGAGACGAGCGTATAGGAGAGAAAATTGTACAAAGTATTGCTCCTTCTA
TTTATGGGCATGATGACATAAAATTAGCACTCGCGTTATCCCTATTCGGAGGACAATCAAAAAACATTGGTGACA
AACATAGGATTCGTGGGGACATCAACATCCTCTTGCTGGGCGATCCTGGGGTCGCCAAAAGTCAGTTTTTGAAGT
ACATTGAGAAAACTGCCCACAGGGCTGTGTACACTACCGGTCAGGGTGCCAGTGCGGTAGGTTTGACAGCTGCCG
TGAGGATTGATCCCGTGACTCGTGAGTGGGTTTTAGAGGGGGGTGCGCTTGTGCTTGCCGATAGAGGAGTGTGCC
TTATCGACGAGTTCGATAAAATGAATGATAAGGATCGTACTTCCATCCACGAAGCTATGGAACAGCAATCCATTT
CTATATCAAAAGCAGGAATCGTAACGACACTAATGGCGCGCTGCGCAGTGATCGCAGCAGCCAATCCCATAAAAG
GACGATACGACATCAGCATTCCCTTCTCGCAAAACGTAGAGCTCACTGACCCCATCCTTTCCCGTTTTGATATTA
TGTGTGTTGTTAGGGATATTGTGGATCCAGTAAAAGACCAGCTCCTTTCGCGATTCGTCGTGCAGAGCCACATCC
GCTCCCACCCGATCGTGAAAGCGCAGGCGTCTTTAGCGAACGCCCAAAAAACCTTTTCCGCCCCTCCCCCCACCT
CTTCCGCCTCTTTCGATGCACCCTCAGGACCCATGGAGGATGGATCACAATCCCAGTCGCAATCTCAATCGCAAG
ACCTCCCCTCCCAACCAGATCCCACCTATACCGCCAACCACATGCCCGAAAAGACCAACAAAAACATTTTATCTC
AAGAACTCTTACGGAAATACATTCTATACAGCAAACAAAAAGTGTTCCCAAGGTCTTCAGGAATTGATAAGGATA
AAATCAGCAGCCTGTACGCAGACTTGCGTCGCGAGAGCAGCGCTCCTGGGTCTGTCCCGATGACCGTGCGTCATC

TTGAAAGCATCATTCGAATGGCTGAAGCGCATGCAAGAATGCACTTGAGAGAACAAGTGAGAGAGGATGACATAA
ATGTCGCGATCCGCGTGCTGCTAGAAACGTTCATTTCTGCGCAAAAGCTTAATGTGGCTAAAACGATGCGTAAAT
CCTTCCAGAAATACATTACTTACAAGAAGGACTACAACCAGTTGCTTTTCCACATACTGACAATGGAAGTGCGGG
AACAAATGAACTACCTCTCCGTGAAGCACCAAATCGACAACCCGGAGCGCGTGGAAGTGATGTGCCAAGACTTTG
AAACAAAAGCGCGCGAACTTAATATTACAGATTTTTATCCGTTCTACAACTCAGATATGTTCACCAGTAATTTCA
TTTTAGATAGAAGGGAAGAGAAAATCATCAAAACATTTTCATAAacatcaatacgtttcagtacgtaattgattc
cttgtaaaatattattaaatttttgtaat

>Phypoly_transcript_03991_PpHSP90
aaactacctgaaaaacttgcacatcactccacaaaaacaggcacgcaggacctgcgagtcttcgtcgttcttctc
aaacacattgcatcgtctgaagtagcttcgatctaccaaaccaacttatacacacatacacaacc***ATGTCTGAAG***
TCGAAACATACACGTTCCAGGCTGAGATCAACCAGCTTATGAGCTTGATCATCAACACTTTCTACTCCAACAAAG
AGATTTTCCTTCGTGAACTCATTTCCAATGCATCTGATGCTTTGGACAAGATCCGCTATGAGTCCCTTACTGACA
AGAGCAAATTGGAGGGTGCTCCCGAGCTCTTCATCCACATTGTCCCCGACAAGGAAAACAAGACACTCACCCTCA
TCGATACCGGTATCGGTATGACCAAGGCAGACCTCATCAACAACCTTGGTACAATTGCACGCTCTGGAACCAAGA
ACTTCATGGAGCACTTGCAATCCGGATCAGCTGATATTAGCATGATTGGACAATTCGGTGTGGGTTTCTACTCTG
CATACTTGGTCGCCGACAAGGTTACCGTGACCAGCAAACACAACGATGACGAACAATACATTTGGGAGTCCAGCG
CCGGTGGTGAGTTCTTCATCCGCCGCGACACCGGAGAACAGCTCGGCCGTGGTACCAAGATCATCCTCCACCTCA
AGGACGACCAACTCGAGTACCTCGAGGAGAAGAGAATCAAGGACTTAGTCAAGAAGCACTCCGAGTTCATCCAGT
ACCCCATCTCTCTCTGGGAGACCAAGGAGACTGAGAAGGAGGTCTCTGACGATGAGGAAGAGGTCAAGGAGGGAG
AGAAGCCCACCATCGAGGAAGTTGATGAGGACAAAGAGGCCGACAAGAAGAAAAAGAAGAAGGTCAAGGAAGTCA
CACACGAGTGGGTCCTCCTCAACAAGCAAAAGCCCATCTGGACACGCAACCCCGCCGACATCACCAAGGAGGAGT
ACGCCGCTTTCTACAAGAGCATCTCCAACGACTGGGAGGAGCACCTCGCTGTCAAGCACTTCTCCGTTGAGGGTC
AACTCGAGTTCCGCGCCATCCTCTTCGTGCCCAAGCGCGCCCCCTTCGACATGTTCGAGTCCCGCAAGAAGCTCA
ACAACATCAAGCTCTACGTGCGTCGCGTGTTCATCATGGACGACTGCAAGGACTTGATCCCCGAGTGGCTCAACT
TCATCAAGGGTATCGTCGACTCTGAGGATCTCCCCCTCAACATTTCCCGTGAGACCCTCCAACAGAACAAGATCC
TCAAGGTCATCAAGAAGAACTTGGTCAAGAAGTGCATCGAGCTCTTCTTCGAGATCGCCGAGAACGCTGAGGACT
TCAAGAAGTTCTACGAGGCCTTCGGCAAGAACCTCAAGCTCGGCATCCACGAGGACAGCCAGAACCGCGCCAAGC
TCGCCGAGCTCCTCCGCTACAACTCCACCAAGTCCGGAGACGAGCCCACCAGCCTCAAGGACTACATCACCCGCA
TGCCCGAGTCCCAGAAGGACATCTACTACATCACCGGTGAGAGCAAGAAGGCCGTCGAGAACTCCCCCTTCCTCG
AGGCGCTCAAGAAGAAGGGATACGAGGTGCTCTTCCTCGTGGACCCCATCGACGAGTACTGCGTGCAGCAACTCA
AGGAGTTCGAGGGCAAGAAGCTCGTGTGCATCACCAAGGAGGGCTTGAAGCTCGATGAGACCGAGGAGGAGAAGA
AGAAGGCCGAGGAAGAGAAGGCCAAGAACGAGCACCTCCTGAAGCTCGTCAAGGACGTCCTCGGCGACAAGGTCG
AGAAGGTCGTGTTGTCCAACCGCATCGTCGACTCCCCATGCGTCTTGGTCACTGGAGAGTACGGATGGTCCGCTA
ACATGGAGAGAATCATGAAGGCACAAGCCCTCCGCGACTCCAGCATGAGCACTTACATGCTCTCCAAGAAGACCC
TCGAGCTCAACCCCAACCACCCCATCGTCATCGAGCTCCGCAAGCGCACTGACGCCGACAAATCCGATAAGACCG
TGAAGGACCTCGTCTGGCTGCTTTTCGAGACCGCCCTCCTTTCCTCCGGTTTCTCCCTGGAAGAGCCCAACTCTT
TCGCCAACCGCATTCACCGCATGATCAAACTCGGACTCAACGTTGATGACACAACATCCGAAGAGCCCACCACCG
AAGACCTCCCACCCCTTGAAGGAGAGGCCGAGGCTTCCAAGATGGAGGAAGTGGACTAAaaaaacgcgatcctct
ttgtaacgatagcaaaaaaattataaaaaatgatgaaggaagag

>Phypoly_transcript_04582_PpHSC70
ggggtggtacggttaccctggtcttacgcaaagacttgcagatcaatcagttcgtacacaca***ATGAGCAAGACAG***
CACCCGCTATTGGAATCGACTTGGGAACCACATACTCCTGTGTGGGTGTGTGGCAAAATGATAGAGTGGAAATCA
TTGCCAACGACCAGGGTAACCGTACCACCCCCTCCTATGTTGCCTTCACTGACCAAGAGCGTCTTATTGGTGATG
CCGCAAAGAACCAAGTGGCCCTCAACCCCGAGAACACAGTGTTCGATGCTAAGCGTCTCATTGGTCGCAGGTTCT
CCGACCCCGTCTGCCAAGACGACATGAAGCACTGGCCCTTCAAGGTCATTGCCAAGGAGGGAGACAAGCCCCACA
TCAGAGTCACATTCAAGGGAGAGCAGAAGGACTTCTCCCCCGAAGAAATCAGTGCGATGGTGTTGATTAAGATGA
AGGAGACCGCCGAGGCTTTCCTCGGAGCCACAGTCACCAACGCTGTGATCACAGTGCCCGCCTATTTCAATGACA
GCCAGCGCCAGGCCACCAAGGATGCTGGAACCATTTCTGGTTTGAATGTGCTCCGTATCATTAACGAGCCCACTG
CTGCCGCTATTGCCTATGGTTTGGACAAGACTGCCGAGGCCGAGAAGAACGTGCTCATCTTCGATTTGGGAGGTG
GTACATTTGATGTGTCCCTCCTTACCATCGAGAGCGGTGTGTTTGAGGTCAAGTCCACTGGTGGAAACACCCATC
TTGGAGGTGAGGACTTCGATAACCGCCTCGTGAACCACTTCATGGAGGAGTTCAAGCGCAAGTTCAAGAAGGACA
TCACCGGAAACCAGAGAGCCATCCGCCGTCTCAGAACCGCCTGCGAGCGTGCGAAGAGAACCTTGTCTTCCAGCA
CCCAGGCCTCCATCGAAATCGACTCCCTCCACGAGGGTGTTGACTTCTATACCAACATCACCAGAGCCAGGTTCG
AGGAGCTCTGCATTGACTTGTTCCGTGGCTGCTTGGAGCCCGTGGAGAAGGTGCTCCGCGACGCCAAGCTCAGCA
AGAGCCAAGTCCACGAGATTGTGTTGGTCGGTGGATCTACCCGTATCCCCAAGGTCCAGCAACTCTTGCAAGACT

TCTTCAATGGAAAGGAGCTCAACAAGTCCATCAACCCCGATGAGGCCGTCGCTTACGGAGCTGCCGTGCAAGCTG
CCATTCTCACCGGAGAGGGAGGCAAGAAGGTCCAGGATCTCTTGCTTCTCGATGTGACCCCCCTCACCCTTGGTA
TCGAGACTGCCGGAGGAGTGATGACTTCCCTCATCTCCCGTAACTCCACCATTCCTTGCAAGAAGGCCCAGGTGT
TCTCCACCTACTCCGACAACCAACCTGGTGTGCTCATCCAAGTATATGAGGGTGAGCGTTCCATGACCCGCGACA
ACAACTTGCTCGGAAAGTTCGAGCTCTCCGGAATCCCCCCTGCGCCCCGTGGTGTTCCTCAAATCGAAGTGACCT
TCGATATCGATGCCAACGGTATCCTCAACGTGTCTGCTGAGGACAAGTCCACCGGAAAGAAGGACAAGATCACCA
TCACCAACGAGAAGGGACGTCTCAGCAAGGACCAGATCGAGAAGATGGTCAAGGAGGCCGAGAACTACAAGAAGC
AAGACGAGGAGGCCAAGGACAGAGTTGAGTCCAAGAACAAGCTCGAGAACTACGCCTACTCCGTGCGCAACACCA
CCAAAGAGGACAACCTCAAGGACAAGCTCAGCGCCGAGGACAAGGCTGCCATCGACAAGGCGGTTGATGAGACCA
TCAAGTGGCTCGACGCCAACCAGAGCGCCGCCAAGGAGGAGTTCGACCACAAGACGAGCGAGCTCGAGAAGGTCG
TGAACCCCATCATGACCAAGCTCTACCAATCCGCCGGCGGAGCTCCCGGAGGAGCGCCCGGAGGTTTCCCCGGAG
GTGCTGGCGGATTCCCCGGAGGTGCCGGAGGTTTCCCAGGCGGAGGTGCCCCAGGAGGCGGTGCTTCCAGCGCTG
GTGCCGGCCCAACCGTTGAGGAAGTCGACTAAttcgagactcgaaattttgaaaaaaataaacccttgtaaaata
gcaaaacaaaaaagagaatagtcaacaaaaaaataaacccttgtaaaat

>Phypoly_transcript_01375_PpIPO4
cgcacacacgagcaccgctttcgtttgcctctgtgctgccATGGGGGTTACTGTGGAGCAATTGACACACTTATG
TGAGCAGGCCCTCCATCCCAACACAGAGATTGTTCGTGCTGCCGTCACCCAGCTCAATGCCGCGCTCACCGACCC
TCAATCAACCATGCTTTTCCTTGCGCTATTGCAACAATCCCCCCATACACACGTAAAGCAATTTGCAGCAGTGGT
GATGAGGAAGCGAGTGGTGGGCCACTACTACAAGCTGCCTCAAAATGAGAGAGGGGTGTTAAAGGCCTCCCTCCT
TTTGATGCTGCATTCTGACACAAAAGTCGAGGTTATTGTAAGGAGGGCAGTGGCAGACGTTGTGGTGACACTTGG
TAAAGCTATTGTGCCAAGAGGTGACTGGAATGTTCTGTTACCCACTATCCTCAATGCTGCTTCTAGTGCTGTTTT
GATTGAGGACAGAGAGATAGCAATGTATTTGTTAGCAGCAGTAATAGACTCCCTTGGGGACCAGATGAGAGAACA
CTATGCATCATTGCACACCCTCCTGGCTGCCTGCTTACGAGACAGCAACCCTTCTATCCGTTCATTGGCTCTCCA
CACCACAAGTGCTTTAATTCCATGGCTGGTAACCAAAGATGATATAGCACTATATGGATCTCTAATACCATTGGT
GGTTGATGTTGTAAAATATTTTGTACAAAATGAAATGGATGATGATGCTATTGAGTCTTTCTCAATTTTAGAGGA
TCTTGTAGAGACACCCATACCTGTGATTACAAAATTCATACCAGCTTTGGTGAACCTCATGCTTGAAATATGTGC
TAACCATCAAGTTGATATTAGTGTCCGACAAAGTGCTCTGTCATTTGTCCAACTTGTTGTGCGGGTGAAACCTAA
GATGGTTGTGAAGAACAAATTGGTCACTCCAACACTCGCAGCAGCATTTGCCATGTGTGCAGAGGGAGAGGGTAA
CGATGAGGCTCAACAGGATCTCTATGAACAAGAGGAGACACTGTCCCCATACATCTTTGGCTCACAAGTGATTGA
TACTGTGGCACTCTCACTACCTGTGAACCAGGTGATAGGCCCTTCGCTCGCACTAGTCGCCCAATATGGCAGGCC
TGAGATGCACTTCCGCCAGCGTCGTGCTGCCATTACTGCAATTACTGTGTTGACCGAAGGCTGTGCGGAGTCAAT
CAAGGACACTCTGGCTAAGGTCGTACAGTACGTGGTGGGGGCACTAGTAGATCCCTCGTTTCTGGTGCGCCAGGC
AGCATGCTTGGCCATAGGGCAATGTGCAGATAACTTACAACCTGAAATCATTGACTACCATAATGTAGTGGTTCC
TGCTTTATTGCAAGGATTACGAGATCCCATGGAATCGGTTGTGCTCCGCTGCTGCTACGCCCTCGAGGCGTTCTG
CGAGTCCATGGGCGACAAAATTGTGCCATATCTAGCCACGTTCATGGAATACATGGGCGAGGTCATGCGCACGAA
GGGCATAGAGACGCAGGAGGTGGCCGTCAGTGCGATTTCGTCCGCGACTGTCGCCGCGGGTCCTCTGTTCGTACC
CTATTTCGGTGCAGTTTACGAGCTGATGCGAACTATGATGATGGCTACCGGCTTAGGTGCTACGGAGCCAGAAAA
GATCATAGTGGTGCGAGCCAGGGCGACGGAGTGCATAGGGTTGTGCGCTCTGGCAGTGGGAGAGCCGGCGAAAGG
GATAGTGCATGACGCGATGTCTCTTGCATTGGCAGGTCTGGAACTTGGTTTTTCGGAATTGAATGAGTTTACCTT
TGGATTTTTTTGCAACTTTTGCGAATTGCTAGGCGCCGATTTTGTGAACTACTTACCAGTTGTGATGCCGCACAT
ATGGAAAGCCTGCGAATCTTCGGATGGAATATTCCCTACAGCAAAAACCCAACTAGAAGTAGAGGGCGATGACGA
AGAGCCTCTTGATGTTAGCGTGCGCACTTCCTTCCTTTACGAAAAAAGTGCTGCTTGTCAGGCTATCGGGTCTTT
TGCAATACATGTCAAGGTTGGCTTTATACCATTTGTAGACCAGTCAATAGCAGTATTGAATCCTTTGCTTAATTA
TTTCCACGAGACTGTTAGAGCTGCTGCCATAGTTTCTATCCAACAGATCGCGGTTCCTATTCACACAGCTTTTCC
TCCACCTACGCCTTTTGTACCTGGCGATTTGACCATCCCTCTGTCGACGCAGAGCAAGGAGTACAGCACACGAGT
GATGCCCGTATTAATCTCGGCTATGCGTGATAAATCGTCTGATGTAGTTGTGCGCGCCAGTGACTCGATTGGTGA
AATTGCTCAGCTGTTAGGTCCTGTTGCTGTCGCCGAATTCATGCCAGCAATTGTAGAAGAATTTTCATCCATTTT
GCAACAAAAGTCTCCTTGCCAAATCCATTTTACTGAAGGTGATGAACTGGATGATGAAGGCAATTCAGAGGATGA
AGAGGGAGGTCCGGCAGAAGAAGAAGAAGAGGATGACGATGCACGCTTCCTTACACTACTCGATAATGTGACCGA
GTGTATGGTGGATCTTGCACATGCTCTTGGGTCACAGTTCACCACAGCCATGCAAGCATTCTTCCCACAACTCAT
GAAATACGCACGCAACAGTGCACATCACTCACTGCGTGCTACTTCCATCGGTGCAATGGCTGAGACCATCAAAGG
CGCACATACCCTCCCTCCCCCAGACCTCACCTCTCAATTGTTCCAGCTTGGGCTAAATGGTATGACCAACCGGCA
AGCTGACCCAGATATTCAGCGCACGGCTGCGTTTTTGTGTGGTGTTGTGATTGAGATGGGCGGAAACAACATGGA
AACGCAGATTACAAATGCACTCCAAGCCCTGCACCCCCTGTTTGGCAGTCCAGATCCGACTGTGATTGATAATGC
ATGTGGTGCAGTAGCCCGAATTATACTCACATTCCCCGATTCACCACATGTGCCCATTGAATTCATCCTTCCCAC
CCTTCTCCCTGCACTCCCCCTCAGAAAAGACCACCAAGAAGATGAACCTGTGTGGAAATGCGTTGCGGAGCTAAC

ACGGTCTGCAAAAGTCAATGCACAGGGGGAAACGACAGTTGCCCTCCTAGAGACAATACTCCGTGGATTGGAAAA
TCCCCCACTACAACCCCAACTCAAAGATTTACTGAAGCATGCACTTGGTGGATGGTTCCCCACATTGCAAGCACT
CTCCAGTTCTACCCCTTCCTTAGTCCCTCTGTTGCACACTGTCACCAATAAAAACCTGTAAattacaacccatct
cctaatatcccaata

>Phypoly_transcript_00177_PpSPT6
ctcctgcgaagcacacataaaagctgttgtttgtgatttcaccagcattcagttgctgagcatcgctggtggaca
caagacATGTCTAGCAATGAATATGTCGAAGAGGAGCAAGACACCCTTGTAGTGCCAGAAGGTGAGGCCGCCGCG
CCAGAGGGAGCGGAAGAAGGATATCAGGAGGGACAAGCGGAAGGTCAAGAGGAAACACAAGAGGGGGAGGTGGCa
gagcaggaggaaggcgcagaagaggaaggcgaaggggaatcaagagaaaagaagaagaagaaaaagaagaaaaag
aGGAGCGCTTTTATAGAAGATGAGGCGATACTCAGCGGAAGTGAGGTCGACAGCAGCGAAGAAGAGGACGGAGAC
TCGCAGAACGAGTACGAGTACGACGACTTCACGGTGCCCACCGGGATggaggaggaggggagagagggaggggga
ggaggagagaggaggaggaggagaaagaagaagaggaggcatgaggagacactggaggaggaTGACTATGATTTA
GTCAGAGAGAACACAGGACAAAACATAGCCAGGAGGAAACAGGATTCTGCAGGGGCAGGAGGGGGGAACAAAAAC
TCAAAGAAACTAAAGAGGCTGAAGCAGATGGGAAACAGGACAGACTTGTACAGTAATATCCAGAGCAGGCTTTTT
GGTGATGATGATGACGAAGCGGGGCCATCAGAAGCCATCACCCACGAGGAACCGCGATCGGATGACGAACTAGAT
GACGAGGACGAGGACGATTTCATCGTGGGCGCGGACGGGAAGCCCATCAAGAGGTCGAAACAGAGGAGGTACAGG
GGAGGAGAGGACAGGGGAGCCCTGCAGGACGCAGAAGACATTTTTGGCAGTGTCGATTTTTTCAAACAAGCATCA
GGCCGGCGTTCTCGTCCTCGGGGCGATGAGTTCGACGACGATGGTGACTATGTCGACGGCACGCAGGGCCTGAGC
GAGGATGAGCTGGACGCAGAGATAGAGGAGGGGGACGAGAAGCAAAAGGACCCCCTCGCGGACATCTACGAGCAC
TACGAGCCGAGCCTGCTCGAGCAGAAGCACTTCACCTCGGAGGACGAGAAAATTCGCCAGTTGGACGTTCCCGAG
CGATTGCAGATGCGAAAAGGCACAGCCTACGACGAGAAGCTCGTGCCCGAAGAGGCCGAGTGGATCTTCACGCGC
GCCTTCGCGCGCACGCACGCGACCGATAAGGCAATCATCATCCAGCGCATCATCCACGTCTTGCGCTTCCTGCAA
GAACAGTTGCTGGAGGTCCCCTTTATTAGCACATACAGAAAAGACTATTACCAACCCTTAAAAATATCTGAACTT
TGGGACATTTATGACTGGGACGAGAAATGGGCTCATTTACAAGCGCGAAAGAATAACATTATGCAAATGTGCAGG
ATGCTCGAGGGCAACCCCAACCACGGCGACGAGCTGCGCGAGTACATGGAGCTCCTGCGCGACGGGCAGACGGAG
GAGGACCTGAGCGACCTGCAGGACTACTTCCAGATGTTCTACGCGGACGAGGTGCGCGAGAGCGCCGCGCACGCC
GAATCGGGAAAGGTCAAGCGCGCGCTGAGGAGGGACTTGTACACTACGGCGAAGCGCAACGGGTTGGGCGACTTT
GCAAAGAAATTCGGAATAACCCCCAAAGAATTCGGAGACAATCTTATGGACAACTACGCAACACACACACCTATA
GACAACGAAGAAGACCCCGAGGCACTTGCGCAAAACTACTTGTGCAAACCATTCCCCGACACAGAGTCCGTGCTG
AAAGCGGCGCGAGTCATGTGTGCGCAGGAAATCGCATTCGACATCCACGTGCGCCAGAGTGTGCGCTCAGTATAC
GTGAAACGCGCCATTCTTTCCACCGTGCCAACGCTAAAAGGCAAAAAGGACATTGATGCATTTCATCCATATATG
ATCATAAAACAAATGGACAAGCCGGCCATGCAGATGGAGGACTTCCAAATGTTACTCGTACTCAAAGCAGAGAAG
GAAGGTTTTATCACAACAAAAATACACACGGACGAGAAAGACGTGCTCCAGGAAATGGACACCTTATATACAAGC
GACGGAACGAGTGCTACCGCAGAAGCGTGGAACAACCACCGCCGCGAAATCCTCGCCCTCACGCTGCGGAACTAC
CTCTTCCCTCTCTTCGCGCGCGAGCTACGCGCGAAACTCACGCAGGAAGCGCAGAACTTCGTCGCGTACGACGTG
TCGCGCCGTCTTGAGCAGCAGCTTCTCGTCGCGCCGTGGGCTCCTGAGGAGGACGACTCGACCGACGCAGAGGAA
GAGGATGACACGCCGCGCAGGATGCGCATCATCGCAACGTGTCGCGGGGGGCTCGGGGAACCCACGATGGCTGTG
GTTTTGGATGACGAGGGAGAGGTTGTAGAGTACCTTAAGCTCAGCTTTTTGAACGAAAGGCGTACAAATGCGGAC
GGACGAGATAGCACCACAGACCTAAAAAACCTCGAAAAATTTGTACAGGACCACCAACCTCGAGCTATCATTGTC
GCTGCAGAGAACCTCGAGTGCAGGCGATACTACGAAGATATTATGGCAATTGTCCAAAAACTGAGGGGGTCTCGC
GAATTGCGCAAGCAGTGCAAGGTGATGTATGGTGACGCGGAGCTTGCGCGTGCATTTAAGCAATCCCATCGTGGA
ATTTCAGAATTCCCTGAGTATCCTCCACTTTTGCGCCAAGCTATTTCGTTAGGACGCAAATTGTTGGATCCAGTA
ACAGAATTTGCTAATTCTTGCGATGACGCTAACGGTCACTTTGTGTTACGCATGCACGATTTGCAAGACATGGTG
CCCCAAGACAGGCTCGCGAAGTCAATCCACCGCGTGTTCATCAACGTCGTGAACGCAGTGGGAGCCGACGTGAAC
CGCATGCTCACGCACAAGTACGCGAACGCATCGCTCCAGTACGTTTCCGGTCTGGGCGTGCGCAAGGCCGAGTAC
CTATTGCAAGCTATTTTCAGAAAGGGTCGATCGCTCGCGTCCAGAGACGAGCTCACAAGTTTCGTGGACAAGTGC
GTTTTCATGAACTGCGCTGGATTCATTAGGATTCGCGACAAGCATTTCTACAACGAAAACGTAGAACTTGAGCCA
CTCGACGATACACGTATACACCCGGAGGACTACAGGCTCGCGCGCAAGATGGCTGCCGATGCGCTGGATCGCGAG
GGCGAGGATGCAGTTTTACAAGAGTGCATTTACGCGATTATGGAAAAGCCGAAGAAGTTGGATGATATTGATTTG
GATGCTTTTGCTACAATGTTGGAAAGCAGTGGAGAGATCAAAAAGATGACACTGTACGACATCAAGAAAGAGCTC
ACACATCAATTCGCCGACATCCGCACTAAGTACACTGACCCTACTAACGTTGATATCTTCCACTGGCTCACGGGA
GAGACCGACAACTCGCTGGCGGCGGGAATGATCATCGCCGCTCGCGCTTTCCGCCAATCGGAACGTTCCGTTTTG
TGCCGGCTAGAGAGTGGGTTGATCGGATTCATCAATTCCGAAAATGTGAGCGATGACAGAGAGGCCGCGTCCAGT
TCTACCATTGCTGTGGGGCAAACCGTTCACGCGCGTGTTCTCAGCATTGACAAGGACAAGTTTTCTCTAAGGCTC
TCGTGTCGCGGCTCGGATCTGGACAACCCGAAGTGGGAGTACGACAAGTACGAAGAATTGAAAAAATTGAACAAA

TTCTTGGTTATCGAGTTGGAGCCCAACAAGGACGAGCCCAAGAAAGAGGCCGTGCGCAGGACCACGTGGAACGTG
CACCACCCGCTCTTCCAGGACACGGATTTCATCGGGGCCGAGAAGTTTTTGGCCGACAAACCGATCGGGGAAGTT
GTAATCCGGCCCTCGTCCAAGGCCCCCGACCACCTCACCTTTTCTTGGAAGTTTTTTGACGGCGTGTACATTCAC
ACTGATGTCCAAGTGAGGGACAAGAAAAGTGCGAGGAGTTTGGGATCACTTTTCATTGGAGAAAATAAATATGAT
GACTTGGACGAAATTGTGGCGAGATACATGGACCCGATAAACTTTTTTGTACTCGAGATGATTAACTACCGAAAG
TTCAAGCGATCTTCCCAGCAGGAAGTTGATCAATTGGTCACGTTCGAGAAAGGAGCAAGCCCTACATCGATCCCC
TACTACATCACAGCCCCTAAGGAGCACCCTGGACGCTTCGAAATCACTTACATGCCCTCAACGCGCCTTAAGCAC
GAGTTCATCACGGTGAACCCAGAAGGCTTCTCGTTCAGGAAACAGCCCTTCAAGAGCGTGGAGCAGCTGATCAAG
TGGTTCAAGTTACACTACAGGGATCCTATTCTCCCACCAAAGAAAGCCGACGAGAAACCCCCCACGCCAGCCCCA
ACACCCGCGCAACTGCAGCAGCTGCAGCAAATGCAAACACAGCAGCGCATGTCGACGCCGCAGATCAACGCAACA
CCCGCGCACCCTGGCGCAGCGACGCCCGCGCACCCCGGCAACGCAACACCCGCGCACCCGGGCAACGCGACGCCC
GCGCACCAGGGCAGCGGATGGGCGAACGCCGCGCAGGGCGGGAACAACTTCCCGGGGATGCAGGCCATGCAGCAA
AACCTACCTGCCACGTCATCACTTTCCTTATGGGGCGGGTGGGACCAAAGCGGAACGAGCACGCCGGGGCACTCG
CAGGGAGGGGCAGGAGGGTGGAATAACAATAACACAAACAATTACGGAAATAACAATAACGCGTTCGCTAACCCA
ACGAATGACGTCACGATGGGATGGGAGGACAACTACAACAACAATAACAACAATTCCAACAATTACGGAAATAGC
AATTCCAATTACGGACAGAACGACAGTTACGGGAATAACCGGGACGATCGCTCGCGAAGGGACAATAATTCCAAT
AATTACGGAAATAACAGAGATAATTATGGGAATAATCGGGGGAATAGTTACAGAGATAACAATAACTACAACAGT
AACAGAGATAGCAGTAGGTACGGCGGGGGAGGGTAtggggggaggaggaagagacaactacaggagtggaggagga
ggagggggaggatacggaggaggaagcggagggggatacggaggagATAGAAACAGAGACAGCAGGGGAGGAAGA
GATAACTACAACAATAACAGGAGAGACGACAGGGATTACAAcaggaggagcgggggcggagggggagggggatac
ggaggagacaggaacagggaTAGTCGAGATAatagagggagagacggagggagagggtgggacgatagagataag
gggagtggaggaggaggaggagggggaggaagaagaagcagagggggagagGGTTTCGAAATATCGGCGCAGCAG
GTCTTCCACGGACTCCTCAGCTTTCCCCTCGTACCGTTCTGGAAAAGTCTTTTGTAAaaaagttgggagctgaag
ttggcctatgtaggtgtcatttggcaaggagctgagcacttcagaggtttcttcgtcataaaactgatgttgaaa
gaaagcattttttgtgagttgttgggaccgaagaaaagccctcaaacactaaaatgggttccaagaaatttcctag
aggtacctcttcgaggatatctgtgcgaaacaactcgtgtttggaatatttttttcctcattttaatactgcggtc
cttagaacagcgataaaactggcaaatagcaaccaaacgctcttccgggttcagttcctccgtatcctctacaat
gagcaatattttataatagaagggatacatggaaacattgagatcaatatttttttgcagttgcaaagtgtcctac
tgaaaagtggacgagtgttcctgccgcagaagtgagtgcgaaggaatcgtagagattgcaattcttaatgttaaa
ggtgtggatttgatctcctagtacggtcggctccggtgggggctcctcaccttcgactacgttatcaaatattg
gttcgatatgccttcatttatttcttggctttttgcttgaatgtttggtagcaaaggagctacgtccaccatatt
ggtgtagatctagagaatgaacgtgagaaaagtactgggttaaaagaaccaggacgttaatgatcgtgaatgtgc
aaacaaatgtggtattaaggtctattttaagtatagtgcagaaaaatgtggcgctaaacatatttgtcatgtgtg
gaaagaagaaat

>Phypoly_transcript_03499_PpAPLF
ataatccggtacaaagaacgctctggatggcaaacaactcctacacagtagattatgcacccacagcaaaagctg
gatgcaaattttcacaatgcaaaacaaaaatagaaaagggatctttgcgcattggaaaatcatatccctctgata
ggtttactgatggagggtctggaaatgaatattatcatcccaagtgctggtttgaggctcaaaagaagacaaaga
aaactacaaagaaagtagagagcgaagatgatttacagggattcgacgttatttcgtcgaccgatcaagaactta
ttctggatcttattaaacataggaatgatcccccgctacactctccaaagaaaaaagcaaaaaaagacgcaggag
gcggagggggagggggaacgcccaaaaagaacaccaaacagacctacatcgacagcgacgaggaaaaaagcacgc
ccaaaaaaggaaattctaataatagcagcaccagtaacggaaataacaagaataaggacaacggcaaaaacaaag
ggaagcaaatttcggggtcggatggggaggaatcggaggacctggaaaaggacgcgtgggctgctgtggcgtcaa
acgggagcagggtggccagagtcctaaatagcacagatatcagacaagtttcagttgtgatactcgcctcagcct
cgagcgaggccataaagcgtgctgccgcgcaagatgatcgcgcttttgaggatgaatttggaggagacccggatg
catatctacaatggttggcaggaactggagtgctatggatcagcacggattttgatgatgcaggctatacatctc
caggcttgtggaccgatacgctatcgcagatcgtgcgccagattttcatgcaaaaaccaaagaagggccttgtgg
tggctatgtggggacctgaaacaaagcgtttccgaaatgagataaagaagcactaccaagacaccatggggacag
tcaagtaccgagcgctcgagggtcccacgcccctaaactcagccttcaactcgaaagacgagctctcgaaaatcg
acgacgcacgcaccttattgagc***ATGCACGCGCTGCACTGGTGGCCGTTCGAGGGCGAGGTCGCGCAGAACCAGG***
AAAAGAAGGAGAAAGAGAAAGAGAAGAAGAAGGGGGGTAGTGTGGGGGAGGGAGGGGCGAGGTGGGTGCTGGAGT
GCAGCGAGGAGGGGGACATCAAGGTGGAACTGATGGGCGGTCCTACGGAACTAGGACGGGGGTTAGTGAACAACA
AGAAGATCAGTCGCAAGCAGTGTGTCGTGGAGACGGTGGATGACGGCGTTGAACTCACTCCCCTTGGTGTGAATC
CCATGACAATGTCGCGCGCAGGAAGCGAAGACGTGGAGATCCTCAGCCGAAACGAGAAGTATACATTGCACGAGG
GCGATTCGTTCACCTTATGCGGCGAGCAGTACCGATTCGTGCTCCAGCGGCCAACGGTCACCCAGACTACAACGA
GCCAAACTACAAGTCAAAAAGATGGCGAAAAATCGGCGAAACCCGCCTCGCGCCAAGCTTCGAAAATCGAGGGCG
CGGAAGGCGAGGGCGAGAAAACGAAGAGGGACGGGGAGGCAGATGAGATAAATGAGGGAGATAAAGGGGAGGAGG

AAGGAAGGGAGAAGCAACGAGGGGAGAAGGGAGAGGAAGGGGAGAAGGGAGAGGAGGGATCACCCGAAAAGAAAG
GAGTGAAACGCAAGGCGAGCGATATCGCCACGACTACTACCACAACCTCCTCGGCCAAAAAAACCCCGCAAGGAT
CCGCAGAAAGCGATTCGGCTACCGTTCCGTTTAGCGCGGGCTACGATTCCCCAGAGGAGAGCAAGGCAAAAAGCA
AGAAGAAGCCCGACGTCAAAACGGAGGAGGCTGATGACGTAATCGATGACGAAGAGCTCGCGCGCAAACTACAAG
AGGAGCTGAACAAGGAAGACGAGGCGCCCAAACCGAAAACCCCGAAATCGAAGAACAGCACCCCCGAAAAAGACG
CAGAGATCGCGAGGAAGTTACAGGAGAAGTACAACAAGGAGAGCGGGGGAGGAGGGAAGAAGAAGAGGAAGCAGG
AGGACGACGAGGACGATGGCGAGGTGAACTGGGCCGCGAAAGTGAAAAAGCAAAAGAAAGGGAAGAAGGGGGGGA
GCGACGACGAGGATGAGGACTGGAACGACGTGGGAGGAGGAGGGGACGAGGACAGCGACGAGGACTACCAAGAGG
AGGAGAGCGACAGCGAAAAGAACAAGAAAAATAAGAAAAAGGGGGCGAGTCCCAAGAAACCACCATGCAAGTACG
GCAAGTCGTGCTACCGCACCAACCCAGACCACCTCCGGGATTTCTCACACCCCAAATAAGttatgaacgtatccg
tttatgtaattgtacttaatttttatagttatatgcaccctc

>Phypoly_transcript_27931_PpPolE3
Aaattcgaaaaatacgagagctcgaaaatttaaaatttcaaaaaaggcgcgttcttgcgcgggaaacgcaggaaa
gagacaagg***ATGAGCGGAGACAAGACAGAACCAAAAGAAGAGAAAGAGAAATCAGGAGAGGACCTGGAATTACCT***
CGAGCAGTAGTGCAAAGACTAATTAAATATTCATTACCAGACAATGTACATGTACAACAAGAGGCTAAACTAGCT
ATTGCACACGCGGGGAAAGTTTTTATCAATTATTTAACAGCTTGTGCGAATGATTACTGCATACAAGGAACGAGA
TCTACCATTTCAGCTAAAGATGTATTAAATGCATGTGACGAGCTGGAAATGCCTGAAATCACTAAAAAATTAAAA
GAAGTTTTGGAAGCATACAAAAAAGAGCAATCAGAGAAAAAAGAAAAAGGGAAAGACAAAGAAAAAGACGAGGGT
AAGTCCGAGGATTAAactttgttatactatgtacaatataaaatcatttattcaaaaaaaaaaaaaaaagagcaa
tcagagaaaaa

>Phypoly_transcript_00085_PpSWR1
ctgaattacgcgagggagcaacatccatagtgttcacgcatctctatcaacatgatgagcatcttctgagccttt
ggcatggaatggagatgcccacgtg***ATGGTGCAGAAGGGAAGGGAAGCAGCAGAGGTGGAGAGCAGGGGGAATAA***
AAGACAGAAGGGGGTTGCAGAGCCCCCCATTCATGATACACGCAATGCCAACACTAAGAGACCATCTCGCACTTC
TGAGCAAGCCTCCCCAATCCCCAAGAAGAGAGTGCGTGGGTCTGGTGTGCCTGAATCACCATCAGTAGAAGCAAT
CGATGTGAGCCAAGAAAGTGAAGTTGCACCTAAGGCTGGAGTAGAGACAGACATTAACATTCCAGCCATTCGGGA
GTATGAAGTAGAGAAAAGGCTAGAATTGATAAAACAACTCACAGAACAGAAAGAAAATGAACTCAAAGAACTTGC
ATATCTGGAAGCGGGGAACAACTTGATGGACTATGAAGCCGGCTTCACAAAGGGTAAGATAATGACCGCAAAGGA
GGAGCCAGGGGAGAATGTGGCAGTAGGTGCAGAAGTCCCCAACAAGAAGGGTCCTGGACGTCCTGCCAAGGATCC
CAACTCTCAATCAAGGAGGAGATCAACAGTTGGCCAGTCTCCTGTTCAAGGTGAATCACTACAAGCCCAATCACC
AATTCCACAAACTCCCTCAGCGCCACAAACTCCAGTACAACCCAGTCAACCTACTCCACAAACACCCACCCAACC
TACAGAGCAGATCACAGCACAAGAAGCTAAGCCAGCGGTAGTATTTCACCAGCCGCTCCAAGTGCCACTTCCATC
CATCTTTACCGTCCCTCCACCTACTTCGGAACAAGACCGTGAGCGCGCACTTAAAGACGCAGCGATCATACGCCG
CGTAGGCGAGCTTCAGCGCACGGGCATGTGGTCCCTCAAGCGTATTCCTAAGGCTGTAGAGCCGCCGCGCGCCAA
GGTCCACTGGGATCACGTGCTCGCGGAGATGGGGTGGCTGGCGAACGATTTCAAGCAGGAGCGCAAGTGGAAGAT
GGCTCTTGCGAAGAAAGTGTCTAAACAAGTCATGAAGTACCACCAGATGCAGGAGACGCGCGAGCAGCGCAAGCG
CAAGGAAGAGGAACACCAGATCCGCAAGATCGCGAGTGGCATTGCTCGTGAAGTGAAGAAATTTTGGCAGAAAAT
CGAGAAGGTGGTCATGTTCAAGGAACAGACCAAATTGGACGAGAAGAAGAAAGAAGTGCTAGACAAGCACCTGGA
TTTCCTCGTGGGACAAACAGAGAAGTACTTCGAGATGATCGCCAACGACCTCAAGCAACCCGAAGGAGGAGCTCA
GAAGGACGCACCAACGACTGAGCAATCCACAGAAGAGAAGGCGGACGGAGAACCAGCCGACGAGAAGCCACTCGT
CCGAAGCCACACGCAAGAGCTGCTGATGGGGGCTGAGGAAACGGGAGGAGAAGAGGGAGCTGAAGATGACGCCGA
ATTCGTTCCACCTGAGGAAACTGTAGATGAGGTCGACGACGAGGAGACGATGGAGGAGGAGGAGACCAAGGAGGA
CTCCGCTGACCTCCCCAAGGGTGAGGAGGAGCTCTCCCAGCTCCAGCGCGAGAGCGAGATGCCACTAGAGGAGAT
CATCAAACAATTCAAGGAACAGGGCGGCGGGGAGGGTGAGCAAGATGACGCCGAGGATGCAGACGCGGATGAAGA
CGACGAAGGGGAGGACGACGATGGGAAAACGGGTCAAGAGCGTATCAGCGAAGCAGCAGCCGCAGCCAACGCAGC
TCAGCCTACGGGATTTACACTGTCCACCACCAAGGTCAAGACGAAAGTCCCCTTTTTACTCCACGGAACCTTGCG
CGAGTATCAACACATTGGTCTCGACTGGATCGTAACCATGTATGAGAAGCGCTTGAACGGAATCTTGGCTGATGA
AATGGGTTTGGGCAAGACCATCATGACAATCGCCATGCTTGCCCATCTCGCCTGCGAGAAAGGAATCTGGGGTCC
TCATCTAATCGTGGTTCCCACAAGCGTGATGCTGAACTGGGAAATGGAATTCAAAAAGTGGTGCCCCGCGTTCAA
GATCCTCACCTACTTCGGAACGCAAAAGGAGCGCAAGCTCAAGCGCCAGGGCTGGTCGAAGACGAACTCCTTCCA
TGTTTGCATCACCTCGTACAAGGTGGTTGTGCAGGATCAACAGGCCTTCCGCCGCAAGAAATGGAAGTATTTCAT
CCTGGACGAGGCGCACAACATCAAGAACTTCCGGTCCCAGCGCTGGCAAGTCCTTCTCAACTTCAACTCCAAGCG
CCGCCTGCTTCTTACAGGAACCCCACTCCAGAACAGTCTGATGGAGCTCTGGTCCCTCATGCATTTCCTCATGCC
ACATATGTTCCAGTCCCACAAGGAATTCAAAGACTGGTTCTCCAATCCCGTCACCAATATGATAGAAGGGACTGA
GACCCTCAACGAAGACCTCATCACGAGACTCCACGCCGTTCTTCGTCCCTTCCTTCTACGTCGCATGAAGAAAGA
CGTAGAGAAGCAGTTGCCTAACAAGTACCACCACGTCGTTACCTGCCAGCTCTCGAAACGACAACGATTTCTGTA

CGAGGAGTTCATGTCTAGCGCCGTTACGCAAGACACCCTCAAATCAGGCAACTTTTTGGGGATCATCAACGTTTT
GATGCAACTCCGAAAGGTGTGCAATCACCCCGACCTCTTTGAGGGTCGGCCCATCATCTCTCCCTTTGATATGGA
CCCGATTGAGTTCTACACCAGCTCAGCAGTGTGCAGAGCCCTGGAAGAGGACCCATTCAAGTCCGTCAACCTCCC
TTTCCTCAACCTCTCTCTTGCCTCCACCGAACTCGAAGTTGGCCACATACAGGCACTGCGCACGTCACAGTTACG
CACCCCGTCACCCAAAATCGTCGAGCTTTCTTCCACTCCCTCTTCTAAATCCACCCCTCTTACAACATTCGCCCC
GAGCACCGTCGCTCCTTCCATGTTAGACGAGCTGCGTTCCGCACGTGAAGCGCGCATGCACCAAGAACGCCTGGA
CAACCTCAAGCATGCTGCGTATATCAACGAATTTAGGTGCGCTCGCAAGCCGCTCTATGGATGGGAGCTACATCG
CTCTGTGCGCATCCACCGCCCAGTCCATGACGTTTATACCTACGCCCAAGACCCCAAGCACTTCCTAGACTACCC
TTCCACCCTAACTGACTTAGTGCGCCTTCCTGAAGCTCGCGCCGCGGAACTCAACCCTACCCTCATCGCCCCATT
TGTATGCATCATCCCAAAAGCCCGTTCCTCACCCCCACGACTCGCCTGCCCACATCCCAACCCCTCCACCGTCCT
CGAAACCAAGCGATCAGAACTCGAGTTGGTTCAGAAACTATCGCCTACAGTGGATATTTTCCGTCCCGCGTTCGT
GCGTTCCCAGGTCTACTTCCCAGACAAGAGCCTCGTGCAGTACGACTGCGGTAAGCTGCAAGAGCTAGCTGTCCT
CCTGCGCACGCTGAAGAGCGGCGGACACCGCGCGCTCATTTTCACGCAGATGACGCGCATGCTCGATATCCTCGA
GGCCTTCCTGAATCTCCACGGGCACACGTACCTGCGCCTGGATGGCTCCACGAAGGTCGAGAAGAGACAACAGCT
GACGGAGCGTTTCAATACGGATCCCAAAGTGTTTGTTTTCATCTTGTCCACGAGGAGCGGTGGTGTCGGAATTAA
CTTGACTGGTGCTGACTCAGTTATCTTCTACGATAGCGATTGGAATCCTGCTATGGATGCACAAGCGCAAGACCG
CTGTCACCGCATAGGACAAACGCGAGACGTGCACATCTACCGCCTTGTTACAGAATTCACCATCGAGGAAAACAT
TTTGAAGAAGGCAAACCAAAAGCGGCATTTGGACGACATGGTAATCAAAGGTGGTGGCTTTACTACCGATTTCTT
CAAAAATGTAGACGTCAGAGATTTGTTTGAGTCAACCAAGGACCAAATTGACCGAGAGAGCGCACCAATTCAGCT
GAGAGAAAGCAAGGCAATTTCGCAGCAGGACTGGGAGCGTGCAGCGGCTAGTGCTGAGGATGAAACCGATGCAAT
AGCCCTACGGCAAGCACAAGCCGAACAAGATTTTGATAACGAAGAGTTTACCGAAGAGGCTGGGCAACCTAGCGA
ATCTCAGCCCAACGGAACGGACGCCCCAACGGCAGCCGACCAAGACATGTCATTCTTGCAGCAAGAAGACGAATA
CGAGCTCCAGGCCACGAATGGCAAGTTGGCTAGCAGTGCCAGTGGATTCGAGTTCGAAGATAGCCTACTCCCCAT
CCAACGTTATGCGGTACATTTCCTTGAGGCCGTGAACGCAACAACGAGCGGAGAGCCAACCGAAACTGTTACTGA
GGAAGTGAGCACCCGAGAGCAAGAATGGGAGCTGGAGAACATCAAGCGCATGAAGGAAGAGGAAGAAGAGCGCAT
GTCAGAAGAAGAAGATCAGCTTTTTTACGAGGTTTCTGGAAATGCGGCCGAGTTCCGGGCTAAGCTGCTCGCCGA
AATGGAAGTGTACTGCCCACCCAACCCTGATCTCGAGCACCTGTACATCCCGCCCTACGATGGGGACTACCTCTC
CCAATTCCCCCCGTTCCCACCGCCGTTCGAGGACGACGATACTCTCCCACAAAACGAACCCCCAGAGAAGCGATC
CCCGCCCCCTAGCCCTCCGGGAGGAGAAGTGGGGGATCAGGAGGCATACACCACTCGCCGCGAAAAGAGAGAAAG
GAAGAAGCATGCCCAGACTGATCATGTTCCTCAGTCTCCTCCCTCCCCTCCCATGCCAACTAAAGGAGGAAGGGG
CAGGAAAGCTAATGGCGGTCTTTTCCCAAGTAGGATTGTACCGAAAACGGCCAGAGACGGGAAGAAAATCGCACC
GTCAGCCCACAAGGACCTCTACCCCTGGTCCCCCGAGGAGGACGAGGCACTGACCAACGCCACCCGGACCTACGG
GCTCAACTGGGATCTGGCCTGCGAGATGCTCCTCTCCAACCCCGCCCTACCCTCGCGGAAGTTCATGCGCACGGC
CACTGCGTGCGCGGAACGGTATCGCACGGTTCTGCAGCCGCGCGCAGCGGGCGAGGCGGATAAGGACCGCAAGTC
TCCTCCCTCGTCTCCCCTCATGTCGCCATCCCAAGCGCAGCCACCGCACTTTGGTATCGTGGATGTCGTTCGCAG
TATGGTTAACCAGAAGAAGGAGATCCCTGGCATGATAGGGAGTGTTGCGCAGACGGACCAATCTGTGCCACCACA
TCCTTCCCATATGCAGGCTGCCATGCACGCTGGAATCACGTCAGCAAAGCCACTTACCCCCCGCGATGTTCTCGC
TCTCATTTCACAGCGCGTCGTGTTGACAATGCCACAAAAGGAAGTCCCGATCCTAACAAAACTGGCTACCAAGCT
TGACCCCAAAATATGCGGCTTCGCGCAGGGGCTCGGCTCGAGCATGGCGCTCGCGATCGCAGTGGCGAATCACGC
CGCGGCATCTGCGGCAGCAGCTCAACCGGGAATCCAGCCAAATTCCATGGTCGCAGGGCAAGGACAACCCGGCGT
GCCTGCCAACACCGCCGCGCCAGCCGCGACGTTTACGGTGCCTGGACCCGGGGTCGTCGGGGGCGTTCCGGCCGC
CAACAGCATCATCCTCAACCCCAACGCGAGCAGCAACGCGCAGACCTCGCGCAAGCTCGCGCAGATCAACCAAAG
CATGCTCCCCACCGCCCCGAATGTCACCATCCCCCGCGCCAACCTCCCCGGGGGTGTCCCCACGGTTAACGCGGC
GCCTGCGGTAGTTGTAGCAATCCCGAACAACCCAGCTAGTGTCGCGGTCCCCCGCTCTAATCCGAATAATACCAT
TAACCCGCAGGGCAACCCCATCGCACCTACTGTAAATATGACTGCTCCCACACCCGTTCTTCCTTCAACCACCCC
ACAACCACCCGCAGCAGGTTCCAAACCCTCACCGTCCAAGAGATCGAAGAAAAACGCAGAAAATGCCGCAAATGG
GGCTGCAGGTGGCAACAAACGCCAACGATCGAACAAAAACGCCGCTCCGCCCGTCGCCGAACAACCACCCGCACA
ACCCACACAATCAGTCCAACCACAACCCACACAATCAGCCACGCTACAGCAAAATCAGATGCACCAACTACAACA
AATACAACACCAGTTACAACAGCAACACATGCAACACCAGCAACAACCCCACTTAGCGGGCCAGATTCCACAAGT
GACACAGCCACATTTGCTCACACAACCACAACCCACCCATGTGCACCAATACTCCACCCAACCACAACCCACACA
ATCCACAGTTCCTGGTCAACCAAATCCTTCAGGCACGCAGTACACCACTACACAACCATACACAACTACTCCCAC
GTATACAACAGGGCCTACGTACGGTACACCAACCAACTACACCACTCCCACAACAGGGATTACGTACGCTACAGC
TTACACCACCCCCACTTATGCCAACCCTACCTCCACACCTAGCACCTACACAACAGCCACCCCCGGCACTACCTA
CCCAGCAACTACCACCTTCAACAACACGACGACACCCGGAACGTATACTACCGCGTACCCCGGCACCACGCCCGC
GGCAACGTACGCAACGATGCCAGCAACTACTCCAGCTGGTGCCACGTACACGACCGCTGCCACGTCAGCAACGTA
CGCGGCACCCACAGCCGCGCGACCATTCACCACAGCACCAGCGGGCTCCACCTTCACGGGCTCTCCTGCGACGTA
CATCTCCGCAGGAAATCCTGCGGGCACGTTTGCCACACCTGCCTCGACCCCGTACACGAACTCGGCTGTGGGATA
TTCGCCGCAGGGTATGGTTATCGGAGTTCCGCAACAGCCAACAATGATAGGAACAATACCTCCAAACGCTCTCAA
ACCCCGCGCAGGGCAACCCAACACGGGCGCATCTCGCCCCGGGGCACCTGCGCAGTCTTCCCCGGGCACTGCGCA
AAACGCGGAGCTGCTCGCAATGATGTGCACGTCGCAGTTCCCTGCGTCCACGAACGCAGTGCAGGGCGTGCTAAT

GCGCAAAGACTTGACTGAGGAGCAGCGCCTGGAAGCCGTGCGCAAGATTATGAGCGAGCACACTCCCATGTGAtc
ttgcttctcgtgttattgtatgtaaagtagtgtacactttgtgaa

>Phypoly_transcript_09236_PpSWC2
attgagatttaaacatgtctaccagaagtttatatatacttacatttgaatattaaggaggggaatattacacca
gaaattgaaaaatcatcaaacagcttagttcttcagaaaatattgagatttacaa***ATGTCTACCAGAAGTTTACC***
ATCCCGCTCCACAAGGGGCAAAAGGATGAACGCGCTCGTTGGGGATGATGCGCTTGTAGATGAAGAGTTTTGGAA
AATGGATATGTTTAAGGAAGATGAAGAGGATGATGAATATAAATCTGAAGCAGAGGAGGAAGATATCATCGATGC
CGATTTCGACAATCCAGAGGAGGCCGAGTcagaagaagaagttgaagttgccaaagagaaaaaagaaaagaaaag
gatggcctatgtagatccaaaagctaaaaaagggccaaaaCGACGGAAGATCCAAGATAAAGCGAAAGCACTCGA
CGCAGAACTACTAAAGGGGCCCGAATCGGACGTGGAAGTGATCATGGACGAAGAACCAAATGAGGAAAAAGGCGA
GGCGCCAAAAGCCGAGCCCAAAAAAGGAAGGGGAGGCAGACGAAAAAAGACGCCCAAAGCCCTAGAAATCCCGGA
GGGCGAGGAAGGGGCTGATGCTGCGTCACCCCCTAGCACCCCCGGCTCAGTCCCTAACACACCTAAATCCACGCG
CAAGTCCACGCGCGCAGTAGCTATTCAAAGGGCCGAGCAGCGAGAAAAGGAACGAGAAGAAACAGAGAAAAAGAG
GTCACAACGGTCAAGGAAAAGATCTCCTGTGGTCGTCCGGCGCATTACACAGGAAGAACTACTAGAAGAAGCAAA
AATGACCGAGATTTATAATACAGAGTCTCTAAAATATATTCTGTCTATGGAAGAAGAAAAGAAGAAGATAAAGGA
AGCACCCACAATCGTAGGTCCAGCAATAAAATATTACTCAAAAGACGGCAAAACGTGCGTGTGTTTTGACGAGTT
GCCCGACGTTTTGGCTTCAGCCCCGCCCACACCCCCATCCCGCGAAATTTGCCCAATTACTGGCCTTCCAGCGAA
GTACAAGGATCCCAAAACAGGAATTTGTTACGCCAATTTGGACGCGTTCAAAACACTTCGCACCAAGCTACTACA
ACAAGAAGAAGCAGAAACCAATTTGAAAATTGCCCAGTTGACCGCCTTGTTGCAAGAATCTAAACGAAAAAAAGA
TCAATTACAAAACACGCCCACGGGAGTTTTGGTGTGAggaaactttttattagatttggtgcatggcaggtaggt
taggctcggctaagctaggctagtctagtctaaattttgcagagggacaaagataaagacggaagagagtaaggc
atcttggcaaaggcttggtggatttggaaatgtaccaaggtctttgtattgtattttatgtaaaatagttcacac
tt

>Phypoly_transcript_00559_PpSPT16
acagcgcaaggaaacctgttaccaagacc***ATGGCTGACAAGTCTGCTGAAGCTTCTGCAGGTTCTAAAATAACTA***
TTAAGCTAGACAGTAAGTCTTTTTGGAGAAGAGTAAAAGCCTTATATGATTCTTGGCAGAAAAAAGATGATTATT
TATGGAAGGGACCGGATGCATTGGTTATAGCACAAGGAACACCAGATGATGATAATCCGTATAAAAAAACAAATG
CTTTGCAATATTGGCTTTTGGGATATGAGTTTGCGGATACCATCATTGTATTTTGCGAGTCAAATATATATGTAC
TCACTTCTCCGAAAAAAATTGGATTGCTTGAAGTATTGAAGGAAGAAAATGCGCCATACGTGTTGTGTTTTTTGC
AGCTTTCCAAAGCTGAAAACAACCAAAACTTGAATACTTTATTCGAGGCTGTCCACAAGAGCAAAAGTGGGAAAA
GCATTGGAGTGTTGCCAAAAGAGAAAATGCAAGGAGCTTTGGGGTCACTTTGGGAGGAAGCGCTTGCGAAATCGG
GGCTGACACTAGTGGATGTGGCACCAGGATACTCTGAAATGTTGTCTGTAAAGGATACTGTTGAAATGAAACTCT
TAAAACAAGCTGCAGAAACTACTTCCAAAGTGATGTCAAACTTCTTAATTCCAGCTGTTGAAACTGTGATTGATG
AAGGAACACAGAAATCACACACTGCCCTAGCAGAAGAAATGGAAGAAGTATTTGCTCAACCTGAAAAAATTAATT
CTAAGTTGGTAGGTGAGCACGTCGAATCATGCTACATTCCAATCATTCAAAGCGGGGGCAAATATGACCTGAAAT
TAAGTGCAACTAACGACAACGAGAATTTACACTTTGGCACTATCGTGTGTGCTCTTGGTGCGAGATATAAAGGAT
ATTGTTCCAATGCAGCGCGCACTTTTTTTATAGATCCGAAAAAGGAAGTAGAGATCAATTATAGGTTTCTTCTTG
AAGTACACAATATAGTAGTGAAATCCATCAAACCAGGTGTAAAAATATCGGTGATAATGGACAAAGCGACCAAAT
ATATTGCCGACAAGCGCCCTGAGTTATCCAGCTCTTTCTTAAAGTGCTGCGGTTATGGAATAGGAATTGAGTTCC
AAGAAACGTACCTGATGCTCAGCAGCAAGAACACACGCACCATCAAACCTGGCATGGCTTTTAACATCATGGTAG
GGTTTCAAAATCTCGAACTTAAGGAAGAGCCTAAAGACCCCAAGAATAAAGTGTATTCTATGTTGTTGGCAGACA
CAGTGATTGTTAGTGAAACCGGAGAGGCGGAGGTTTTAACTAATTCTGTAAGCAAAAGATTCGGTGATGTCTCTT
ATTTCATTGAGGAGCTAGACAACGAAGAAAAGGCCTTGCCGACTACTACCAAGAAATCCTCTGCCACTGTGAAGA
GCGAGAAAGCCGAGAAGCTTGATGACGCTTTCAAGAGTGCCACGTTTGACGGACCACGCTCTCGAGACCAGAAGG
GCAAATCAAAAGGCTCGGAGGATAAGATAAAGAAACATCAAGAAGAACTAGCAGAGAAGTTGGCACGGGATCGAC
GGAAAGGTGCAGACGAAGATAGTACCTCGGCTGGCCCTTCCACCGTCAAACCCTCCTCAAAAGATATTGTGGCTT
ACCCTGACACATCTGCATACCCTCACGAAGCTCACAGAAACAAGATTTTGGTTGACCACAAGGCCGAAGCTATTC
TTTTGCCCATCTATGGGTATCTAGTCCCCTTCCACATTTCGATCGTGAAAAATGCAAGCAAAACAGACGACTATC
TCAGAATCAACTTCGTAACACCCACCCAGGTCATTGCTCAATCACAGGGCCAAGGAAAGGAATTGCTATATATTA
GGGAGGTGACATACAGGATTCAAGACCAAAGAAACCTTACAAATTCCTTGCTGCTCATCAAGCAACTGAGGAAGC
AAGTGAGCGAGCGCGAAGCAGAGCAGCGCAATGTCACTGGCCTGGTCTCCCAGGACAAACTTATTGTTAGTAAAA
ATCCCAGGAATCCCAAGCTCATGGGCCTCTTTATTCGTCCTAATATCAGTGGCCACAGGACAGGTGGGACACTTG
AAGCACATACAAACGGCTTCCGTTTTTCTACTAGCAAGGGTACTACTGTTGATATTTTATTCAAGAATATCAAAC
ACGCATTTTTCCAGCCATGTGACCATGAACAAATTGTTTTAGTGCACTTCCATCTTCACAACTATATCATGATCG
GCAAAAAGAAGGCTAAGGATGTTCAGTTCTGCACAGAAGTGATGGAGGGGTCATCACACTTAGATGGAACATCAC
GTTCATCATCACGTGATGAGATTGAGGATGAACAAGCTGAGGCTGAAATGCGAAATAAACTCAACAGGGATTTCC
AGACTTTTGTCAAGCGTGTTGAAGAGGTTTCTGCCCAATCTGGCACTGAAATAGAGTTTGATATTCCGTTCCGTG

AGCTAGGATTCTTTGGAGTGCCCTCCAGGAGTAATGTGTTCCTTCAACCCTCTGTGCATTGCTTGGTGAGTCTGA
TTGAGCCCCCTTTCTTTGTACTGACACTCAAAGAAATTGAAATTTGCAACTTTGAGCGTGTATCGTTTGGGTTGA
GGAACTTTGATATGGTGTTTGTGTTCAAGGACTACGAACGACCAGTAACCAAAGTTAACAATATCCCCGTCGATT
CCCTAGAGGCCATCAAGGAATGGCTTGATGAATGTAACATAAAATTCTACGAGGGCCCCACAAATCTGAACTGGA
CCCACATCATGGGCACTGTAAGGCAGGACGTGGATCAATTCTTTGAAGATGGTGGCTGGAGCTTCTTGGAGGACA
AGGACTCCGATGCAGGCGATTCGTCATCTGAAGATGGTTCCGAGTTTGAACCTGAAAGCAGCGGCAGTGGTGAAG
AAGAAGAGGCCAGTGAGGATGATTACTCATCAGATTCGGATGCCTCAGGATCGGATTACAGCTCTGCTTCAGATG
AGGAATCAGAAGAAGAGGGATTGGATTGGGAAGAGCTTGAGCGCAAAGCTGCTGCTGAGGATAGAGCAAAGAAAT
ACGATGAAGATGAGCCTCCTAAGCGAAAGAGAGCTGGAGGAGCGTACTCGGACGACGAGGACGATCACCCCAAAT
CCAAAAAACCAGCAGTATCGAAGGGAGATAGTTTGTCCAGAAATGGAAGCAGTGAGTGGTGCCAGAGACATCACT
AAgtacccaggactggatattgttcttaacattcacattttttatttctaggccaccccccccaggggggttggtgtg
gaggggtgggccccccactgaccagcacaaatttgtacatttgcaaccaggcaaatggataatttcttggagctg
aatcatttggaagtgctgtgtgggctttattcataagcagtttgggggggcatttgatagtaccactgtattcctt
gtttgattatgatttcaagtgtcttggtgttgtagagggttgaatttgctttttgtgtagcagttttttttatcat
caagcttatgtgaatctgtgctgggcatgtgtgtactttttatcagcaacttttgctaaagcattagctggtgctg
gcaccaaaaatccatgcactgcactatattcacattcttgggacaataggccaggcttgacatgcttaatgactg
aagcacacatataaattggcatcaaaggggaagtgcttaaaaacacatcaaacaatcgtgccacacaagtaacat
catctaagttgtgtgcaaaccaagttaagacccaactaagcgcaaaaaaagggttgcacatgtgattcaacaatga
aatgggaaatttttggctcaaatattgataataatggcacaaggtgatccaagtatgctataatataatccaagt
taggtcgtaaggcatctctaatgtgaaaaagggacagtttttctaacattgcaaatgccaatttctcatcagtta
ccattaagaaaacactagcaatgtcatgatatccctgaaagtatttcaaggtggggtgctcagaaagaacagcca
aaataatatttgataactctttgcgcttctgagcaagtagcattggatcaccctctgtgaacttccacagtgatc
tctccacatctctctccacttgatgccgctctgggtgttcttcttgtattaattcagtgaatgcacgaccatcag
caggaatgcactcacactgctggatgccaagtaagatgggccaggcccttgctcgtattgcattgttcaccagcc
caccctctgttagtgcatattcaatcagtgctgcccgtgacacaggggggtgatctaaacaataaacaatgcatt
gggctttggaatctttggaacccttcatctattgcgcggtgtattgcggcaagaagaagaggagaatgtagttgg
tggggcaagaaagtttgacctgcgtgtccttcgttgataataaaaaaaaaaaagtgctggtgtgttt

>Phypoly_transcript_05191_PpSSRP1
gtctttcttgtatcttgctttcacacaccaacatacatatgattcattttaattttgtttttttaaaatattact
aaatgatatacatgttgtattatttttttttcaaagtgcatgcacatatttttgtgaataaaagtatgtttgtgtt
gttttgtgtgaaatttgatgtgaatgtgaggcatgaacatgatggaaatgagaggtgcatgctgtacaagagcga
aaagaaaatgacgcaagaaatccattgcgtgggcagctttttattcgcaggtaggtctttaaaaatttagcgcaa
caggttcccgcgcaaagttgttcttccaa***ATGGCCACAACAGCACATCAATTCAACAACATTACCCTTGGGGGTA***
GGGTTAGTAGTACAAAAGGTGTTCTTAAGGTGAACAGCACCATGTTCTTGTGGCGGACAGAAGCTGGAAGAGTGA
TATCGGTACTTCCAACTGACCTATCACAAGCTTTTTGGACACGTATTTACGGAAGAGTATTTCAGCTCAAATTGC
AGTTAAACATTGGAAATTCTATCAAATTTGATGGATTTAGAGAGTCCGATTACGATGTTTTGAAGACATTTTTGT
CTGAAAATTTTCATGTTGAACTTACTACTGTACAATTAGCTACAAAAGGACTGAATTGGGGGGAATACGAATTAG
CTGGACCTGTGATCACTCTCGCAGTTGACGGAAAGCACGCTTTTGAAATTCCTGTTTCTGAAATTTCTGGTGCTG
TAATGCAACCTACTCAAAAAAATGAAGTTGCAATTGAATTTCATCAAGATTCCACATTGGAAGATGAGGACGAAT
CCCTTGTTGATATTCGTTTCTTTATCCCCAATCGATCCACAGAAGAAGGAAGCAAGACATCAGCACAAATATTAC
ACAAAGGACTGTTAGACGTAATTGGTGGTGGCATTCAAGGAAAAGATCGCATTGCAACTCTTAACAAAGTACCCA
TACTTACTCCAAGAGGTCGTTATGATATTGAACTTTATCCCACTTTCATGAAATTCCGTGGCAAAACACATGATT
ACAAAGTCACTTATGAATCCGTATCTCGTCTATTCCAGCTTCCAAAGCCTGACCAACATCATATATTCTTTATTG
TTACACTAGATCCCCCAATTCGACAAGGAACAATGCGGTATCCTCACCTTGTCATGCACTTCAAAATGGACGAGA
AGTCTCCTCCAGTACCTATCACACTGCCTGCAGATCTCAAAGAAAAAGAACAATTTAAGAGCTTGCCAGAGGAAT
TCAAGGATGCAGATGCTTATGAAGTGGTTAACAAGTTATTCCGTGCTTTTACACAGCGCAAAATTACCACTCCAG
GCTCCTTCAAGTCTCATTCTGGCGCTTCTGCTGTCAAATGCGCCTTCAAGGCTAATGATGGATTCCTTTTCCTTC
TCGAACGAAGCTTTTTCTTTGTACACAAACCTCCTCTGCATATGCGATTTGAAGAAGTCTCCAGTGTGGAGTTTG
CACGTGTGGCATCAAGCACTAATGCACAGGCCAACCGTACGTTCGATCTTGTGATTAGGCTAAGTGACTCAACTG
TGCACCAATTCACTGGCATTCAAAGAAATGAATACGGGTCTCTCTTCAATTTTATTCAATCAAAGAAGCTTCGCA
TAGAAAATCCTGATGAAGCAACTGAGGAATTGGCAGGGTTGGCAGGAGTGATCGGAGCAGATGATGATGAAGAAT
CTGAGGAGGATGAGGACTTTGTTGATGAGGGTGAGGAAGAAGATGAAGAGTATGACTCTAATGAATCAGAAAGCG
GTGAGGAGGCAGAAGAAAAGCCTAAGAAGGACAAGCACGAAGGTAAGCGCAAAAAATCAGACGGAGAAGAGAAAC
CCAAGAAGAAAAAGAAGGCAGCGGAAGAAGAGGAAGACTAGggtgtgatcattttgttttaatactttaactgta
tgtctattttccactaagctgtaaaatagtacttttacggcgaataaattctcgttataatg

>Phypoly_transcript_13763_PpSET

tttttttttttttttttttttttttttttttttttttttttgttcgctgtgggtttgcagctatcaaacctgctc
ctcttctcttttcttttgtccctgggccaccccaaaagcaatttgtttgtttttcaacaaagcatccttccattg
cactagac***ATGGCTGCTCCCAAAACTAAGAGATCCAAGACCGAGCCTGAGGACACTGTGGACGACGAAGACATCC***
AGGTGTTGGAGGGCATTCAAGATCAAATTGAGGAGCTTAATGACAAGGCAACTGAGGAGATCTTGGAGGTTGAGA
GAAAGTACAACAGACTCCGCAAGCCAATGTATGATAAAAGAGCTGAGGCTATTCGCAAGTTGCCTGAGTTCTGGT
TTAAGGTGTTCACAAGCCAAGACATGCTTGCCGCATTATTGGTTGGTGAAGACTTAGCTGCATTCAAATATATGA
AGGAATTGAATGTAGAGGATCACGACGACGTCAAGTCTGGCTTTAAAATATCACTTAAATTTGACCCGAACCCTT
TCTTCAAGAACGAACTACTTTCAAAGGAGTTCCGCTATGATAAAGAGGGTAACTTAACTGTTATCCCAACAAAAA
TTGAGTGGAAAGATGGAAAAGACCTCACAAAGAAGTCCCAATCGAAGGACACTAAGGGAAAGAGGTCAAGGGATG
AGGAGTCAGAAAGCTTTTTCACGTGGTTCAATCCAGATGACCAGGATCTCGAGTTAGCTGAGATTATCAAAGAGG
AACTGTGGCCCAATCCCGCCAAATATTACTTAGGATTGGTGGAGGATGAGGCCGAGGAAGAAGGTGACGATGACG
ACGAAGGTGAGGATGATGGCGAAGACGGTGAAGGTGAGGGAGAAGGAGAAGGTGAAGCTGAAGAGGACCAGTAAa
cgacaccccacgcgccgctgttagatatttattttgttgacgccacgctacacattaaaatctgtaaagtaagct
tcttcatcactacccctccacaaccggcaatttttgttttattaataaacaaaaatttcaaaatt

>Phypoly_transcript_11436_ PpNAP1L1
ccagctttcagtgtgaggttcttgttcgtctttgttcgcatctatctctcgtc***ATGGGCAAAGGTTCTACTTTCG***
GAGGTCCCCCTTCTGCCAACGCGGCTGCTGAGCACGACGAAGAAGAGCTCGACCTCGCAGAGCTCCAAGATGCGT
TGGGCAGCCCCCTCCACGAATTGCCCCCTGTTGTTCGTCGCAGAGTTGATGCTCTCTCAAACTTGCATAAACAAT
ACTTGACCCTTCAAAAACAATACCACGCTGAGGTCAGAGAGCTCGAGCGCAAGTACCAGAAGTTATACGATCCTG
TATTCGATAAACGCGCCAACATTGTATCAGGCAGCTATGAGCCAACTGATGAGGAGGCTAAAGTAGAGAAGGCTG
AAGAAGAGGAGGGCGTGAAGAAACGTGAAGCCCAACCCGGGGATGCTGACATCAAGGGAGTCCCTGAATTCTGGT
TGACTGTGTTACAAAACAATGGCTTTGTGGCCCAATCTATCTTTGAGGAAGATGAGGAGGCTCTGTCGTTCCTTC
GTGATATTCGCTATTCTGAATTGGAGGGCGAGAATGAAGGATTTACACTTACCTTCCACTTCGCTGAGAACCCCT
TCTTCTCAAACACCACCTTATCCAAGACATATCACATGGAACAAGATGAGCTTATGGGTGAGCTCCAATATGACC
ACGCCACTGGAACCAAAATCGAGTGGAAGGCTGGCAAGAATTTGACCGTTAAGCAAACCAAGAAGAAGACCAAGG
CCAAGGGAAAGAAGCCTGCTCGTGTTGTCACTGTTGAAGAACCCTGCGACTCCTTCTTCTCTTTCTTCAGCCCCG
TCCAGCTGCCCGCCAGTGAGGAGGAGGAACTATCTGAGGAAGATGCACAAAACATGGAAATGGACTTTGAAATTG
GTTGCATTATCAAGGACAAATTAATCCCCTTCTCCGTCTTGTGGTTCACAGGTGAGGCTGCCGAATATGAAGACG
ATGATGACGATATGTTTGGCGATGAAGAGGGTGATGATGATGAGGAAGATGATGAGGAAGATGACGATGAGGAAG
AGGAGGAAGAGGCACCCAAGAGAGGAGGCAAGCGACAAGGAGGAGGACCAGGACACAGTTTCGCACCCCCACCAG
GCGCTAAGCCCGGAGCTGGCGCCCCTGGCCAACCCCAACAACCCGAATGCAAGCAGCAATAAgcttgtgctcttt
ttttaatttgtcccccc

>Phypoly_transcript_03998_PpCAF1A
*tt****ATGGATGCTTGGCTCAGTATCTATTTGTGCTTGGTTTGGGCTGTCTTTATGGATGCTTGGCTCAAAACTAAGC***
GTGTAAATACTCCACAAAATGATGTAAATAATGCACCTGCACCCATAACAGATAACATGGATATAGACACGACAC
CCAAACAAGAAGATACAAAAATAAAATCACCATCAAAAACAAAATCACCATCAAAACCATTACCTCCAGACCTCC
AAAATTGCTTTGTTCTATTGAGCCCGTGTCCAACGTTCCCACCCACACAAAACACCCCACCACCTCACCCAAATA
TCCCCAATGGCAACACATTGCAAGACAAACCGATTCTTGTTGATCCCTCTGTAAATCAATCGCATGCACCTAATC
CTGCCGTGGATGCTTTAGCAACCGATCAACCTGCTAAACCATCTCCTAAAAAGCGGAAGCTCAGTCCTGAGGAGA
AACAAGCCCGAGAGGACGCCAAGAAAGCGAAGGAGGAAGAACGTAAATTACAAGCAAAACAAAGAGAAGACGCCA
AGAATAAGAAGGAGGAAGAGAAGAGATTATTGGCTCTTGCTAGAGAAGAAGAGAAGAAGAAGAGAGACGAAGAAA
AGCGCGTGAAAGACCAAGCAAAAGAAGAGGCCCGTTTGAAAAAAGAAGCTGAAAAGAAGGCACGCGATGACGCGA
AACTACAGAAGGACCTCGAGAAAAAAGCCAAAGAAGAGGCGAGACTACAAAAGCTTCAAGAGAAGCAGAAGCAAG
AAGAAGAGGAGAAGCGCAAGAAGGAACGCCAGCAAGCGGTGTTAGCGAACTTTATCAAAGTTGCCCCTGTTGAAG
CACCGCGAAAGCCACAAAAGGTAGACCCGGATTTGTTGATTCAACCTTTTGTTTGTGGCTCAGAATTCGTCTTCC
CTCCCTCCTATCTCCGTACAAGGCAGCCCTCACCTGGTTTTGATGACGCAATTGCACACAAAGATGGACAGATAA
CCATCGAGAAATTACAAGTGGAATTTAGGGAGCACATGCACGGGAAAAGGAACAGGAAGAGAGATAAAAAGAGAA
GGGCGGAGAAGCTCCAGGGCCTACCCCCCGCGCTCAGCAACATGAAGCTCCTGCGCTTCCACGACAACGTGCGAC
CCGCCTATTACGGCACGTTCTCGAAGCGCAGCGCGGTCCTAACCGGACGCAGGCCGCTTAGGACCGATGACGCGG
TTTTTGACTACGAGGTGGATTCGGACGCGGAGTGGGAGGACGAGGGGGAGGGGGAGGAGCTCTCGGAGAGCGAGG
GGGAGAAGGACAAGGAAGAAGAGGAGTCAGAAGAAGAGGAAGAGGATAAATGGGTGGTCCCAGATGGGTACCTTT
CGGAGGACGAAGGAATGGACAGCGATGACGAGAAGAGTAAAAGGGCGATGAAACTGAAAGCTGAGGAAGGGGGAG
AGGGTGGGGATGCAGATACTGCGTCGAGCAGTAGTGGTAAACGACCAGCAGAGCATATGGAAAGAGACAAGACAA

AGCGAGAAAAGCGCGAGAAAAAACCATGTATAATAAGCACGACATTCGACCCGGATTCTTTGGACAGTTCTGCGA
AGGCGATCTTGACAAATCTTTCCGTTCAGCCTCTCGTTGATTGCCCGATTGATTTAGATTGGAAGCCTCCCGCAG
ATCCAGAAGAACAAAGTGCCGATTCGCGAAGTTCAAAACAAGTAGTTCCAGATAGTTTAATGCCTGCTTTGATTT
TGTACATTCACACCCATCCAATGTCTCTACACAAAATTATTGAAGGTTTCCACTCTGAAAATACGCAAGTTTCCA
AGCGCCAACTAGAGCTAAAAATACGAGAAATTGCCGTGAAAGTCAAAAAATCTTCGTGGCAGGTCCGAGAGGAAG
TCGTCAAATCCTATGACGTCACACTACCCGAAATAATCGAGAGCAAACCCGTTCCGCCCAAAACCCCCCAGAAAA
ACATGACAGATTTCTTTGCCAAAGCCGCCGGCGCCAAAAATCAACCACCAAATTCAAACTCAAACTCAAATTCAA
ATTCAAACCCCAATTCAAATTCCAGCACACCCACCAAAAAAACCCCCACCAAAAACACCCCCACCAAAAACCTCC
CCACACCCTCTTCCCACACCCTCCCTTCCGAAAACAGCACAAAAAACGCACCTACCGAAAACGGATGTCACACGA
CCCCAAAAAGGAATATTATGGATATGTTTCAGAAACATCAAAAGACCCAGGGTTAGgttccactgcgtcttttg
tttgtgtaattattttgtaatatttcaaaaaataaaatatcttgcgaaagt

>Phypoly_transcript_09859_PpCAF1B
attaatttgaattttagcgggaagcaccagctacaatcttcttgtgcctgttgttgtagtttatgctcccttagt
tgcctttctttatgtcctagagttgtcttcgtgcaccataaaATGAAGGCGGACACTCTTATGGTCCTATGGCAC
AGCAAGGAACCTGTCTTCTCTGTGGACTTTCACTCTTCTGGTAGACTTGCTTCAAGTGGTGCAGATAATGATATA
AAGATATGGAAAATTGGAAAACGTGCGGATGGAAGTGTTTCACTTGATTTCTTGTCTAGCCTCACGAGACACACC
AAAGCAGTGAATGTGGTTAGATTTTCACCTGATGGAAACTTCCTTGCCACAGGATCAGATGATGGATACATATAT
ATTTGGAAGTTAAATACATCTGTGCCAGTATTACCCGATTCTATTGACAAAGAAGTGTGGTCCGTGGCTTATACC
TTGAGAGGAAAAGCACAAGATGTGTATGATTTGGCTTGGTCCCCAGACAGCTCGCAGATCATTGTAGGTAGCACC
GACAATAGTGCCTCTGTTTGGAGTGTGAAAGGAGCGCAATGCAAACACCACTTTGAACACCACAACCATTACGTA
CAAGGAGTAGCATGGGATCCTTTGGGAATATACCTTTTGACGTTCAGCAGCGATCGCACGCTCCGCGCCTACACG
CGAAAGCTCCCAAAGAACAAGAAGTCCCACAAAGCCCTTCCTCCCCCTACCCCTTCCTCTTCCGCCTCCGCCTCT
ACCTCTTCCTCCTCCTCCGACCCCCTTCTCCCTGTCCCGGACCCCCTTTCCTCCCCCTCCTCCCCCGTCCCCGCC
CCAGCGCAAAACCCGCCCGCCGCCACGCCCAGCAAAAAACGGGTGAAAGAAAAGTGGATCGTCGCGCAGCTCGTC
AACAAGCGCGAGTTCCCGGGGACACAAGGGCTGCTCGAAAAAGAGAAAGAAAAGGAAAAAGAGGAATCCGGTAAA
GGAAAAGAGAAAGAAAAAGAAAAGGACTGTCTACAACACAAGCTATTTTTGGACGAAAGTGTAAATACATTTTTC
AGGAGGCTCACATGGTCCCCTGAGGGCTCCCTTCTGTTTCTTCCTACTGGAATTCACAAAGCCTCCCCTGCATCT
CCTCCTACCAATGTATGTTATGCATTTTCTAGGAATAATCTTAACAGGCCGTTATTTTACATACCAAGCGGGAAA
CCGGTTATAGCCGTGCGATGTAGCCCAATCCTGTACAAACTGTGGAACGGCAAGCACCCTGCGCAAACCAAACCC
CTCTCTACCTCTCCCACCCCCTCCACACCCTCCTCCGCACCCTCCCCCACCTCCTCTTCTACCTCCCCCACTCCC
GCCTCCTCCACCCCTGTTTCTACCGATGCACCCTCCGCCCCTACCTCCACACCCCCTGCCACTATCCCAGATACC
CCGATGAGTGACTCCCCCACCAACACCGCCCCTACCCCCACCCCCACCCCTGCTGCCCCTCTCCCCATCTCCCCT
CCATCTCCCTCCTCCCCTCCCTCGTTCGTGAAGCTCGATTACCGAATTGTGTTTGCGATCGCGACGGTGGATTCG
GTGGTGGTTTACGACACGCAACACTTACACCCCCTCGCGTTTATGGGAGACATGCATTACGCGAATATGACTGAT
GTAACTTGGTCGGCTGACGGTTCGTCCCTGATCATGTCCTCGACGGATGGCTATTGCTCGTATGCCACCTTCGCT
CCTGGCGAGCTTGGCACTCCCCTGGAAGAAAATGAGATACCAAGTTGTATGAAAGAGGCCATTTTACAAAGACAG
CAGAAAGCAGAGGAAGAGGAAGAGGAGCCCAGCCATCATGCCTCCGCTTCTCACGAACCCAATGGAGAGCCATCC
GCGTCCGCGTCCGCGTCTGCAGTCGCATCTGCGCCTGCCGCCACGTCCGAAGATACGGCTGATGCAGGCTCGCTT
TTTTTTGGGAAGAGAAAAGAGATTCCCCCAAATACTACCCAAGACCCCTCGCTCGCCAGCGCAAAACCTGCCGAA
AAGAAGCCTCGCAGATTGGCTCCTACGTTTATTTCCGCTCTTCCCAATAAGTAAgtaaaatgttaaatgcgtttg
ttttaaggtaataaatgcacttaatactaaaaaaaaaaaaaaaaaaaaaaaaaaaaaaaaaaaaaaaaaaaaaaa
aaaaaaaaaaaaaataaaaaagggtgatggggagggggtgggggtgggaaagttt

>Phypoly_transcript_09477_PpCAF1C
cccacaacaagcaataaacgaggcgtgcgcagctttcccttcccgcgcatttgttgcaagATGGCCAAAGCTACT
GAGGAGGAGATACCAGAGGCAGAGGAAAAAATTATAAATGAGGAGTACAAAATATGGAAAAAGAACGCACCATTC
TTGTACGATTTGGTGATGACACACGCTCTTGAATGGCCAAGTTTAACTGTTCATTGGTTGCCAGACAAGAAAATA
CAATCCAACAAAGAGTTTTCTGTGCAACGACTCGTTTTGGGGACACATACATCTGATGCAGAACAGAATTACCTC
ATGATAGCCAAAGTTCACCTCCCATTGGATGACGCAAATATTGACCCACGTAAATATGATGACCAGAAAGGAGAG
GTCGGTGGATTTGGTGCAGTGAGCGAGAAGATAGAGATTGAACAACGTATCAACCACAGTGGTGAGGTGAATAGA
GCTCGTGCTATGCCTCAAAACCCTACTATCATTGCTACTAAGACGGTTACTTCTGATGTTTTTCTCTTTGATTAC
ACTAGACACCCCTCAAAGCCATCAGCAGATGGAAAGTGCAACCCCAATGCCATTTTGAAGGGACACACAAAAGAA
GGGTATGGTATTGACTGGAATCCTTCTCGCGAGGGCCATCTCCTTTCGGCTTCTGATGATAAAACTATTTGTTTG
TGGGACATACACGATGCCAAAGCAAATACCACTATAGAGGCAAAGCAAATCTTTAGGGGCCACAAAGAAGTTGTT

GAGGATGTTGCCTGGCATTGCAGCCATGCAGACTATTTTGCTTCTGCTGGCGACGACAAAAAATTGATGATATGG
GATGTCAGGACAAATGCAGATCCACTGCAAGTAGAAGCACATGCTGGAGAAGTAAACTGCGTGGCTTTTAACCCG
TTTTGTGAGTTTCTTTTAGCTACTGGTTCTGCTGACAAGACTGTCGCCCTGTGGGATATGCGCAACCTCGATGCC
AAACTGCATTCCTTTGTCTCACATACTGATGAGGTATTCCAAGTGCAATGGTCACCACACCACGAAACAGTACTG
GCCAGCTGTGGCTCGGATAGGCGAGTGAATGTGTGGGATCTATCTCGCATTGGTGATGAACAAACAGCAGAGGAC
GCAGAGGATGGTCCCCCTGAGTTGCTCTTCATCCATGGAGGGCACACCTCAAAGATCTCTGACTTCTCTTGGAAC
TACAATGAGCCATGGGTGCTTGCATCTGTGGCAGAGGACAACATCATTCAAGTGTGGCAAATGGCAGAAAATATT
TACAATGATGATGACACAGATGAAGCCCAGCCCTCCAATGAGGGGgatttggagtgatgtgtgggttaatcaccc
tactttccttttctctctttgtacacatagattaaattatatgttctttctgt

>Phypoly_transcript_01462_HIRA
ggttagagttaatgtatttttttaaatttgtagcacaggaatataaaaatgataagcagtttacatctctatttga
agtagcaaaagaaacagctgcaatcttgtacacaatttgtgtgcaagtagataacactatccaaccatcctggat
tcttgaatttcttcatttgcttgttatgttgtatgaatgcttggacacagtgtgtagaaaagagctttcactggc
agaacgagcaatggacggtaacttgagacaacccagattgtgtaacacttgggtgtggtgATGCCCGCACAAGGA
ATAAAATGCAATCCTTCACAGCCACGTGCTTGCGCGAAATTTGAAGATCAGCAGAACACAAGTTATAATGAACAA
ATGAGGATTTTAAAGCCTGATTGGGTCACCCACACAGGATACCCAATATTCTCGGTAGACATTCACCCAGATGGT
AGTCGATTTGCTACAGGAGGCGGAGACAACAAAGTAATCATATGGGCTTCTGCACCTGTACAATATGAGGAACAA
GAGGCCAACAATTCATGCCCACGAAAGCTTGCAGAGCTCACCTACCACATGTCTCCTATTAGCTGTGTTAGGTGG
TCACACAACGGGCAATTTTTAGCATCCGGCTCAGATGATGCACTAGTGATGCTGTGGAAACTCTCTTCAGGTCCT
GCGTCAAAAAGTTTTGGTTCAGATGAGCTCACTCAAGAAAACTGGCGCTGCACCGCTACTCTTCGTGGACATTCG
AAGGACATTACTGATGTTGCATGGTCTCCTGATGACAGTCAGCTCGCTTCGTGTAGTGTGGATAACGAAATTTTG
GTCTGGGATACTGCTAAAAATTCTGTAGTAGCAAAATTGAGTGGGCACACTGGATTTGTAAAAGGAGTGGCATGG
GACCCCATGGGGCGATACATTGCTTCTCAGTCAGAGGATAAGACAATGATAGTATGGCGGTGCTCCGACTGGAAA
ATCGAAACAAAAATCGAAGAACCATTTGTCCGGTCTGCTGCCACTACTTTTTACAGAAGACCAAGCTGGTCCCCA
GATGGGCAATTCATAAGCTCTGCACACGCGTTCAGCGGTTCAAACCATGTAGCCATGATCACAGCACGTGACACG
TGGCGGTCTGTGTGTGACTTTGTAGGCCACAAATTACCTATTGTGGTAACTCGGTTTAATCCTGTTATATTTGCA
GACACAGCAGGAAATAATTATGGAGTGTGCCTAATAGGAGGGCAAGACAATACCATTTCTGTGTGGACAACCATA
TCATCTCGTCCTCTTCTTGCTCTTAATTCTTGTTTTAAACAGAGTGTGCTTGATGCATCATGGGCAAAGGATGGA
TTCAGCTTTATGTGTTGCTCTACAGATGGGACAGTAGTATGGGTAGAATTTGCAAAAGGAGAATTAGGAACTGCT
CTTTCACCAAAAGAAATGGATAAAGTGCTCAGGAGCATCTATGGTGACCATATCCTCTCTCGCCCCAAGAGCACA
GTAACAGTGGCAGAGGATCCCTCTCAGCTCTACCTCCCAAAGACCACTGCACCAATGAAACCAGTAGCAGCACAG
CCACCTCCTGTGCCTACACAGCAAGTAGAAACCAGATTAGCCAATGGCAAAAGACGCATACAACCCCAATTTCTT
GGCAACATTGCTTCTACCACTGCAACCACTCCCTTCATCCCCCCCCCTACCCCCTTCTCCTCCTCTTCCTCCTCC
TCTACCCCTTTCCCCTCTTCTTCTTCGTCTTCCTCTTTTCCTGCTCCCTCTGCCTTCTCCTCTTCCTCCCTCCCT
ACCCCCCTGCCTACTGCTGCACAAGTGAATGGATTGCAGGTGCCTGCCAACAGCTTGTCATCTAATGGCTTGATT
ACGTCATCATCTGTGAACAGCTCTGTGATGTCATCAGCTACTACAAATCACTCAACCACATCATCAGCTGTACCT
GTGTTCATCCCTCCCACCCCGCTCCCTGGTTCAAAATCAACTTCACCTGTTGCTGTTCGCCACACAATCTCTCTC
CCAGATGTTGGAAAGAGGAAAGAAAGTGATGATGTAGTAGAAGTGCCACCACCAACAAAAAAGCACAAAAAAGAT
GAAGAAAGTGATAAAGAGAAGGGAAAAGAGGCAGAAAAAGGGGACAAAAACAAAGACATAGAGGCAGACCACACA
AAAGAGAAAGAGAGGGAAAAGGAAAGAGATAAGGATAGGGAGAAGGAAAGGAGGAGGCGTGAGAAAGAAAAAGAG
AAGGAAAAGGTAAGAAAAGAGAAAGAAAAGGAGCGGGAAGCAACAAAAGAGAGCACAGGAGGGACAAAAGAGCGA
CACATGTTGTCAAGGCTGCACATTGCACATGCACAACATGCTCCAAAATTATGCAAACTTATTTGGAACCCACA
GGACTACAGTTAGAAACAAAAACAGAAGGAATGGGAACACAGGTGAGCTGCCGCAACACAAAATTCCCACAGCAG
TTGTGGCGCTCATCTATTCCTGGAAAAGCTGTTTTGGTGTGTGGAAATCAATCTTTTTCGGCAGTGGCTATGGAA
GATTCCTTGTTGTATATATTTTCAGCAGTTGGCAGATTGATATTTCCTCCTATTTCTATGAGTGCTCCACTTTCA
TTTTTAGAGGCTAATAAAAACTTCTACTTGGCTGCTATTACTTCTGACTGTCTACTGCATGTGTGGAATATAGCA
CAGCAATGTGCTACTATAACAGCACAACCAGTTAATATTTCTCCTCCTTCCAATCTACACATTTCCCATTTGGTG
CTCAGTGATAATGGCCAACCTGTAATAATTTGCTCTTCACATACATATGCATTTCACACTGAGATGAAAGTGTGG
ATGAAAATCGACTCAGTGTCTTCCCCTTCCACAGGTATAACAGATGGATTTTTATCAAAATTAATGGCTACAACA
GAAAGTGGAAATAGAAATATTGCCACAATGACACAAGAAAGCGATGAAATGCTTGGTGGTTTACTGAGCATGGAA
TCACATCTTGCTGCTAGTTTAGTTGTTGGCTCTAGTGTAGAATACAAATATTGGCTGCTTACATATACCCGCTAT
CTTTCCTCAGAAGCTGATGTCAACCGCCTCACAGAACTCTGCAAATCTCTCTTAGGTCCTCCCACCGGAAAAACT
GGCCCTGCCCCCACCCCTGCACAGGGTAAAAACTGGGAACCTTCCATATTAGGCATGGCAAAACGCGAGCTGCTG
CGTGAGCTGTTGCCTATTATGTCTACGAACCGCAGCCTGCAACGTTTAGTCGCGCAGTTCCGTGAAGGCCTCGAG
AGTATATCTAAATAAtatgaagtaaccacaaatc

>Phypoly_transcript_03291_PpUBN

ggccagtttttggcactagttgtcttcttgtagagcgtacactgctcccaggcttcccccaagatg***ATGGAAGT***
GGACAAGCCAGAAAAACAAGAAAAGAAGAACCCGTGTTATAGGTTCTCGATATCACTTTCAGAAACGAAGGGCGT
AGTCAACTATGCAGACCTAATGAGCCAAGAACGCAAGAAACGCCACCTCCTCTCCAATCCTCCCTCAGATCCTTC
CACCAATCCTACTACCACAACTACAACCAGCGAAGATACTCCAAAGACACCACCATTAAATGGCTCTGTGGATGA
AGCGAGCATAGATACAGCCGGTGATGAGAGTGTGGAGGCAGCTGATGAAAGTGTGGAAGTAGATGAGGGTGGTAA
CGCAAGCGCAAGTAAGGATGTGAAGAAGTCATCCCCCAAGCGCAGTATGCCAGTATTGCCAAAGAAAAAGACCAC
TAACAGACTAGTACAAATCATAGACAAGATAGAGAAGATGTACACCACTGGTGGTGACTCGTTCATGAATGGTTA
CGATACCTCCGATTCATTCATTGATGACTCTGATATGGTGGAAAAGCCAACGGAAGAGGTAGAGAACGAGTACGG
CGGATTCTTTGTGCACTCTGGCGAACTTAAGACAAGGAAGAAAAAGCGCGTCTCCGATTTCCAAGAAGATAGCCC
CGATAAAAATAAGTCAAAACGAACGAAAAAACTGAAGATAGAGGACGAGAAAGGAAGCCCGAAAGAAAAAGACAA
GAAGAACGATGAAAACGGAAAAAAATCGAAGGATAAAAAACCCAAAGACAAGAAAAAGGAAGAGACGCCGAAAGA
CAAAGAAAAGAAAAAGGAGGACTCAACGAAAGACAAAGAGAAGAAAAAGGAAGATCCAGGCAAAGAGAAAGAGAA
GAAAAAAGAAGAGAAAGAGGAGACCAAAGATTCAGAAGAAGAAAAAGCAGCCTTGAGACAAAAGTTATTACAGAC
TGAGGAAAAAATAGAGCAAGCAATCAAAGAGCTGGAGACGGAGGCTAAAAAGAAAATTGTGCCGGGACAGACAAA
GCGAATTCCGGCGGAACTGGCGCCTCAGCTATCAAACCTGTACGAGGCTTTACAACAAACGGCTAACGGGTTTAT
CACGGATCGCCTCCTGGATCGGCTCGTAGAATTTTTGCCTTTTAAGAAAAGTACCATTAAAATGCACCTGAAGAA
ATCAAAGCACAAGGCGGCTGTTGATACCATGGAAGTAGAATTGAAAAGGCTAGAGGACGCATATGATACAGAATA
TAACTTACTAAGAGCGGCAGTTTTCAACACAATGGCCGAAGAACAAAAGAAGCAACAGCAAAATCCTCCTCCCAC
CGATCCCCCTGCTGACCCGAGCAATCCTGCAGAGCCAAAGAAATATGCGTGGACTCTTGCCCAAAAGAACTTGCT
GTACGATACACTTGTTGCGGGTTATCAAATTGTGGACTGGAAAGTCCAAATAGCCCAAGAACGAACAACGGGAGC
CGAAGGAGAGCCCCCAATACCAAGCCAAAAGGCTGAgcgtgctaagattgtccaaaaagtattggagattttccc
ggcaggacaagtaaccacctcagaactcacacaaatatattccgtgcgaaaccaagcccgaaaaaagaaagaaca
gaaacagaaagagctcgaaaaagcgagcaacccaaatccggaaagcgccgcgaacgcgaactccaattccaaccc
ctccccttcccccteccccttctctctctccctctagctcctccgccctcccctcctcttcgtctacgcccgcctc
tccgaacccctcgagcgctgcgtcacaactaatgcctcctccctcctcctcctcctcttcttccgcaccgaccac
ctccccatttttcaacgccgccactacgtcaccgaagatcaaaaaggaaaaagccaaaaatggagaaaagaaggc
cgccggaccgggggcgcaaaaccggcgaaagtgtactcgcgcaagaagacgcagggcaattcgaattcttccgt
cccctccgcgtcctcggtcctagcgagcctatcctccgctcccccgctaggccacatcctcaccccacccgtctc
tcactcccagatcccctccgcatcctcgtccctctccctctcctctcccacgtcctccacccccatccccagttc
ctcgtaccccaaatcccccgcgcagcagcagcaccccaccctcccgtccatccaatcccatttttcgcagtcgct
ctccggcctcaccctccccccctctcgccccgaaaccattccccagcacccccctcttccccgcgcgcaccctatgc
atccacccccctcttcacccctccctctcacctccctggcactcccgccagcccatccacccccctaccaaacccc
ctcctcgcaacacgcaccactacccttcactcccattgctcccgtgaaaacacaagaaggtggagcgttataacc
cctccatcctccctcctcgcaacacgca

>Phypoly_transcript_00351_PpCABIN1
ctttgcgcgcgcaattcaaattgcaacaagcaaatgtacaatacacatcagtggacgcaagggtaactc***ATGATT***
AGATTTGCGGCCATCAACACCCAGCCCTCTCCTGAAGGGAAAAAGCATAAAACACCAACAAAAGGCAAAGATGCT
CTGGAAGCAGAAAATTTTGCGCTTTACGAGAAAGCACTTCGCCATCAGTATGCTGGAGAGTACCAAGAGGCAGAA
AAGCTCTACAAAAGCATCCTTGCATCGCCGATTGTTAACGAATTTGTGGAGGTAAGTAAAAGGGATGACCTGTAC
TACACTCTCCCTGTGGTGACTCTGCGATATCTTGCCTACAAAAACTTCGCGTCACTGTGCGAGACCACTTCACGC
AAAACCGAGGCCATGGAGTATTACGCAAAGGCACTCGACGTCGATGGCAGGGATGTAGTGGTATGGTACCATATG
GCTCTAGTGTGCATGAGCGTGGAGAGGCTACAACTTGCCAGATTTGCCTTGGAACAAGGTTTAAAGGCCTCTCCT
GATCATTGGTTGTCCATAGACAAGCTGGCCGAGGTACTGTTTATTCTTGGGGACTACGGCGCGTGCATGAACGTG
GTTGATCGCATATTGGAATTAGAGCCTGGATTTTCCAAAGCGATTTTACTGAAAACGAAAATGATGTCAGAAAAT
CCAGACAACAAGTTCAAAGTTGAAGCTTTTCTGTGGGAAGCCGGAGACTTAGGAGATTGTAACATTGATGATTTG
GTAAGCGCTAGAAGTAAGCGACAAAAAGTTGCAGATTTTGACCACGACACGGTAGAAGAGCCTCCAAGAGAGTTC
GTGCTAAAAGAGAAAACCTGGAGCAGCTTTGCGGATTTGCTTTACGATATTTATAAGGAAATGACCTCCGAAAAA
GGGAAATCGAAGTTTCTGAATGATCCCATATATATCACCGATCCCGCGCCCCTCAGTCCCCCAGGCTCGCCCGCC
GCGTCCGCGCTCAACGACTCGTTCCCCTCTCTGAACTTCACCACTCCCAGTTCCGGCACCGACCTTTCCAATTCC
GGGAGCGGCACTGCCAGTTCCGGAACTAACCAACCGGGCTTGAACGAGAGCGCGACCAACTTGAACACGAGCGCA
ACTAATTTGAACACGAGCGCGACCAATTTGAACACGAGCACGAGTAATTTGAACACGAGCGCGACGGGTGTGAAC
TCCGGCGAAACGGAGGCGCCAAATGTGGAAAGCGGGCGCAAATCGACGCGTGGAAGAAAACCTGCGGCAGATAGC
GCAACACGTCAATCAGCTAGGGTGAAAGAAATCAAGCACGACAAGATATCAAGTGATCTGGACGAATACCTGAAC
AAATTACTTAACACGCAAGAGGAATCCGATGAAACAACAGCATCCTCTGGAGAGGaaaaaaaaGACACCAACGAG
TCTATAGACGAAAGCTCCAATCCTGTCCCACCTCCTACGCCCACAAAGGACGACGAAAGAAAACAGGTGACTCGG
GCATTGATAGCCTTACAGTCGCACACTCCCGCGCTACGTGGAATTGTAGATTGGATCGTTGCCTTTTTAGGAGAA
ATCTCGACACGTGTAAGCTTTTTTTGGGGACCAGCGCTTTCCGCTAAAGTAATGGGCTTGCAGGCTCTAGCTAAT
CGCCATTCCACCTGGTTCAAAAAGCACTACTTAACAATCGCAGAACTTCATTTCGACGCCCACGTGTGTAGCGTG
ACATCCATTGGCCATCTCCCGGCACCCACTTTCACACCAGCGCTCCATTTGGCCGAATACGCGTACTACAGTGCT
CAAATTTTCGCGCAGCAGAGCCACGACCTAGATCCCGCTTTCCAAATCAGGTCGTTATGGCTTAAGGCCAACAGT

TGCATTCAGGCAGAAGAAATACCGATGGCCATGGAATGCTTGGAAGGATGCAAGGAGCTCTTGGCCGATAGCGGT
ATTCCCATCATTTGGGTCGCACATTGTACGCACAACCGCAATATTACTGCACAGGGAATCGAGCGAAAACTCCAG
GAAATTTCAGGACAGAACACTATAACGACCGCGGCAGACCACTTCATGCGCGAGAACTACGAGCAAGTCGTCGCA
TCGCTCGAGCCTATCGCGCGTGGCTACCCCGAATCGGCGATAGAACCTGACAGCAGGCAAAAGGACATAACAAGC
GCCAAACGAAATAGACTTAAAATCTTCGATTTATTACACCAGGCGTATTTGAAACTGAAGGATTTGAAGAACGCA
TTCTGGTGCTGCGAGCGGATGCTCGCGGAATCACTGCCCCACTACAACATGCCCGAAATGCACGCCAACTTCCAA
TCTCTGCTGGATTGTATTATAGCCACTGAGAAGCAAGCGCAGACATTAGGAGTCTCTCTGTCGACCCTGAACCTC
CAAAAACTAAAGAAAGCTATTATGGTCTTGGTAAAGAAATCAACTGTCCCTATGCTCGTGATAAACGGCGTGATG
GTGTTTTACGCATTGCACTCCGAAAACATGACCCCGACGCAGAAAGTGGTCCTTATGCACCTAACCCACCACGAA
CTTTCACGGAAGAAGGCTTGTTATTTGCAAGATGGCAAATTTCTTCGCCGTTTTATCGCGCTGCTCATCCCGCTG
CACTTCCCGGAGTCGGACGAGCCAATGAGCACGGAAGCTTGGAACCTCCTTGCCGAATACCGAATCGACATAGCA
ACGGAATTGGCTCAATGCATTTGGTGTCTTTACGGCATAAAACTCCACCCAAACATAGACGAGCACGCGCACAAA
ACGCAACAAACACCGCGAAGCGCGAAAGAGCTCATGCTGATTTTCATCCTCGCAAGACGTATTTATCAGGACAAC
AAACAAAAGCTTCCCAAGGAGACGAAGGATGCACTTTCCAAGCTCTACTCCTCCTTCAAAGATCCCCCCGAAAGA
GTCACCCCTTTAGTAAAGGAAATCGAAAGATATCTGATCGATACCGAAGCCCGCCTCTCCTTGCAAAAAATAAAC
GAGCTCAAAGCCGAGCATTCCGCGAAGGCCAACAGTTACCGTGCGGTGCTCGGAAAATGGACCGAAGACTCGGAA
CCGGAGACTGAGCAGTTTGACCTGGCTTCTAATCCCGATTGCGATTTTGTATATAGAGATTTGTATTATATGATG
GCACTTACCTACCCAGACTCGGACTCAGCCTATGAAGAATTGATTAAGAAAGATTTATTCTGCAACCCGTGGCAC
ATTGAATCGTGGGTTGCTCTTGGGGTGATGCACCTACGCAACCTAGTGCTGCTCACCGAAGAGAAAGGCGTTTTC
TTCCCTAATCAGCCTCCGTTGGTGGTTTCCGTGGAAAATTTGAATCGGTTGTGCGATAAATGCCTCTCACAAGCA
CTAGAGCTGGATGGCGAAAGAGAGGACCTGTGGAAGGAACTCGGCCTCCTGCTCTACCAACAACACAAGCACATC
CTCCTCCCGTCTTAcccccttcttcccccctctccctcttttaccccttcttcctcctcgccctcccctccttcc
tctcttctatctctctcggAACCTAACACCTCGCCCAATGACTCAATTACCGCATCGAACGCCGAACCCGAAAAA
GACACGAGCGACGATTTCCAAAAGAGGAAAGAACTCGTCGCGCGCGTTTACGAGCGCGCTGCGTCGTGCTTCAAA
CGCGCGTCAACGCTCAATCCCAAAGAATGGTTATATCCACAGATGCTAGGCAAAATAAGCGAAAAATGCGGGAAA
CCCCTCAAGGAATATTTAACATTATACCGTTCTGGATCGGAAATGCGGAAGAAAGAGGAACCTGAACCACTGTAC
CGCCTGCACGCTAGCAGGCTCAAACTTTTACTACGAACTCCTACACTTAGTGAAGCTAAGATGGAGGAATTAGAA
CAATTTAACTTCGCACCTATTGAAAATCCAAGTGAGCTTTCGCATGAACAGAGGTACAAAAAACTTGTCAGTGAT
TGTCTGGCCGCAATGGAAGAATGCAAAAACGACTACAAATGGTTCCACAAATCCGTTTACATGGTGTCATACACT
TTGAAATATGGGCCAGAACCAGACGTGAAAACGGCGCAGAAAGCTCTCAGCTTCCTTTTCAAGCCCATGAGCCAG
AATTTCTGGTACATTTGGAAAAAGAAAGGATCAGAAATTCCACCGGGGAAAATGGACCAGTACAGAATAAAGTAC
ACAAAGTTATTCGTTGTTCTGCTGGAGGACAATGTAGACAAAAGCGCACTGGAAAACTGCTTGCTAAAAGTGAAG
AGAGACCCCAAGTGCGTGGAAGTGGGCTTGGTTGAGGATACTTTTGCTGCTTTCTGTCGCGTCCAAGCTATTGTA
ATCCAGCAACTATCAGAAACCCTTAAAACAGCAGCGTCCCTCCCCCCTCCCTCACAAGACTCTTTGCCCAACCCC
TCCGAACCCTCTGTCAATCCAACTTCCTCTTCCGAAACCTCACCCACCTCTACTGCTCAAACCTCTACCGCTATT
CCGGCAACCGAAGACTTTACCTCTTCTTCTGTCTCTTCTTCTACCCCAGCTACTTCTACTCCCCAACCTCCTGCT
TCTGCTATCCCTCCTTCTGCCTCCTCTACCTCTTCAACTATCCCTTCTGCATCTCCTTCTATCCCTCCCTCTACG
ACCTCCTCTTCCATCATCTCGCCTCACCTGGAATCGCTCGAAAAAATTTTACTGAACGCGTGGGAGAGCCACACG
GAAGCCAATACAAAAAAATCGCGCGATGCAGGACCTCGCTAAccaagttctagaacaagcctttaaattatatat
ggcatacggccaagaccaaaatatgccaactgacgaaccaactgatatcctacaagcggcttctcagttttgcga
aaagaagtttgccaagggaagaagacgaaaagaaacagactctaaagatgcaaacaagaaagacggagccgcacc
acgaagaagaaggaaagtggaagaaaaagagaaacctacaaatgaaccaccacaaagcgaagaaatggaaattga
ttcctcacaaaatgcgccctcccaggtgtagatacatttttttaaaaaccgtgccggcttattaaaaaaagccata
aaagttcgaatttacatcaccg

> S3S101K5_5354_LULU_Contig588_N11D4_PpNASP

gcgcgcaaacttacaaagaATGAGCACAACAAGAGAAGATGGTCAACCTCTAGAGGGTGGATCAAACACAACCAC
TACCACCACAACAACTAGCACAACTACCTCTCCCCAAGAGACCGCTGTAGAGGCTGGCGCCTCCTCTTCAGAGCC
CAAAGAACTAACCCCAGAAGAGGCAGCGAAGAAATATGAAGAAGCAAAGGCTTTGTTTAATGAAGACAAGTACGC
AGAAGCTTCTGATTTATTTGCGGATGTGTTGACCCACAAGGTTGCTGTCCATGGCGAGCTAGGGCTAGAGTGCGC
TGAGTGCTACTTCTTGTATGGAAAGTGTCTATTCTTGCAGGCAAAAGCAGAAAGCTCCGTGTTCGGTCCCACAGC
ACAACAAAAAATAAATGAGGATGACGAAGACAGCAGCGATGAAGAAGACGTACCACCAGTTGGTGAGGGAGGAGA
GAAAACCGAAGGAAAAGAGCTAGAGGAGAAACCAGAGGATGTAGAGAATACACAAGTCGCGTGGGAGACCCTCGA
AGTAGCCCGACTAATTTACGAGAAGCACCCTGAAAAAGCGGCCGAGTTGTCTGACACCCGATATGCATTGGCAGA
GATCTTTTTGGAAACAGATAATTGCAATGACGCCATCGAAGAGTTCAAAAAATGTGTGTCTTTACGCGAATCGAT
CTTTGTTCCTAATGACAGAAGGATTGCTGAAGCTCACTATCACCTTGGACTCGCGTATTTTTCGACGGACAGACC
AGCAGACGCACACTTCCACTACTCGAAAGCCGCAGACGTGCTCGATTCGAAACTGAAGTCACTTCCTCCTGGCAG
TGCAGAAATCGAGGACATAAAATCCATTTTGGAAGACTTGGCGGCTAAAATGGAAGAAGTAGATCCCAGCCTATT
GGCCAAAGAGAAAGAAGCCACCGAAAATGCAATAAATGGCACGACAAATGGAAAGGGCAAAGCACCCGTCTCCTC
ATCCTCATCCTCCGCTCCCCCCTCCTCATTTCCCAAAGAAGAGACCGGCTTCGCGGCCCCCATCTTCGACAAGAC

ACAACCAGTCACGAGGTTTGGTGTGGTTGGTAGCTCAGTAAAACGTGCTACACCTGTCACCGCCTCCTCTTCCTC
CTCTTCCTCTTCGTCCTCCTCTTCTTCTTCCACTCCCTCAGCCTTCCAAATCGCTTCCGCTTCCTCTTCATCCTC
ATCCTCATCCTCATCCACAACCCCATCTCCTGCACCACGACCGGCCCTATCATCCTCCGGAGAAAAACGAAAACT
GGAGGACATAATGGGAGGAGAAGAGGGATTCGAGAAGCGAACGAAAACGCAGGACACAGTATGTGAAACCAGAAC
ATGAataaacctgcttcgtagctaaaccaaatccggtacataagcgcaagtacttccagga

>Phypoly_transcript_05212_PpXRCC1
gttgcgcgcaggacgcatgggcttttacactaatcatgcaccaaacatgaatcattttctcctttagttg***ATGGC***
AGAAACCTGCTGTGAGTTCAAAGTGATATCAGTCTCCTCCGAAGATGCGGAGCAGAAAGCAAGCAACATTGCAGT
GCAAGATTTGTATGCGACATGGCAAACAGAACCTGGAGAAGAGAAAGCAAGTATATTGTTGGAACTACCAGCTGA
AACAACATTGCATACTATCGAAATTGTGAATGCTGGAAGTACAAATGTAGAGATATACGTGACAAACTCGGAAAA
CCAGCAGGATGAAGAAATCTTATTACCAACTGCTATCATGATGACTCTGGGCGAAGTGAATAGCAATACTAACAC
CAATCGGTTACGATTTTTTGGACCTGACAAGCTCACAAAAGGAATATCGGATAAGGCGTGGAAATACATAAAGAT
CGTATGCATATCATTCGACACTTCTAGGAAGCCCTTTGGCTTAGCTCGCATTCGCCTTTTTTCCTCCGCTCCTTC
TGACGAATACATTGCTTCCTCTATTGCCGACAACCGCTCTTCTATGGTCTCCGTTCCCTCTTCTCAACCTACCAC
CTCAACTTATGTACCTCAAAAGTATGCTTCAAGGAGCAAGTCTTTTGCGGGGCCACCTCCTGATACCACAATAGC
CCACGACACAAACAACAAAAAGACTCCAAACCATACAAACACAGCCAACAAATCCAACAATAATAATCGTACAAA
CGACACAAACAACTCACCGGCCAAGTCAGCAGCAAAGCGCAAGAGGGTCATCATGAATGACGATTATGACGATAC
AGAAGGCGATTCAGCGAAAGACCAAAACCCACGATCCCTCACTTCCTTACACATTTCCCCTCATGTGAAGAAGAG
CGTACCAGTAGCAAATAGGTCGAAATCGCTTGTATTTACCTCATTTAACAAAATATTGAGCGGCGTTTCGTTTGT
AGTTTCCGGCATCGCTAACCCAGAGCGACAAGAGCTGCGAGATAAGGCCATAGAAATGGGAGCCCAGTATTACGC
AGATTGGATCGAATCTGCTACTCATCTTATTTGCCCATTTACCAACACTCCTAAGTTTATTTTGGCCCAAAAGGC
AGGTGGTACCATTGTCACTCCAGCATGGATTTTAGACTGCTATAAAAACAAAGTGCGCCTTCCAGAAACAGAATA
CCTATTAGCGGAGGTGAAGAAGATCGACGACGCGGGGATGAAAAGCACCATGGGCAACATGCGCGATTGGCTCGC
TACATTTAGACCGAAGTTTGACAAACCCGCAAATAATAGTACCAATACTACCCAATCCCAACCTCAACCAAAACT
AACAAAATCCCTAACATTTGTTAACAACGCCCGAATCACCAAGGACTCACAACCAGAAGCTGACGTCGATGATAC
CTCTTCCGTCAGCACTGAACCGATGAGCGACGACGAAAAAACAAATAATGACAAAGAACCCCCAACCAAAATGTA
CAAAAAAGATAGCGTGGTGAGTAGCGCCCTTACAGACACCACGGAGGATATGGGACCAACTCAAGATAACGTTTA
CTTCTCGACAAGCGAAGACGACACACATATCGACAAACAAGGGACAATACGAAAGGACTTCCAACTGAACAAACC
CAAGAAATCCGGCGTTTTTGACATCTCAGCACCTTTGCCGAACTATTTCAAGAACATCAGGGTTGTATTATATGG
CAACTTCGACCCCGCTTTGAAAACACTTCTGTACCGATACGTGACTGCTTATTCTGGAGAAATAGAGGATAAGCC
GACTAGCAAGACAACTCACATTATAACCAACCAATTGTGGGATAAAGAATTGCAACAACTGCGAGACGAATATCC
CTCAGCATGTATAGTAAGACCCAGTTGGGTGTTTGATTCGCACAACGAACAAAGAAAGGTGGAAACCAATTTGCA
CAGTACACAAACGGTTTAAcaaacttgtatcttttatttaatgtaaaatattctaaggc

>Phypoly_transcript_12278_PpNHP6A
tatcttcccgaacacatttctactctgacttgaaagttgacatgtgtaaaagtagtttcgaaacaaagaattgta
gcgaatcttcaaagtatatcaactcgagaattgtagcgaaagggacatacaacgagtattctgcgaaaatgcaaa
tgagtagcgagaaacttctatctttttttcaaaacattgttaaagcctgcagccggtctttcgagtctattgttct
tttctgctttattctgctata***ATGGAAGGTGAATCTCAAGCTCCTGAGGATATGGTTAACGAGGAAGAGGTCAAG***
GACACTGGTAAGAAGAAAGGAGGCAAGAAAGCGGTTAAGCAACTAGTGAAGCCGAAAGCTCCAGCCAAAGAAGAC
AAGAAGAAGGTTGACGCCAAGGAGGATAAGAAAAAGAAGGACGCCAAGGATGACAAGCAGAAGAAAAAAGAAGAG
GAGGCGGAGAGCGAGGAGCAAGAAAGCGACAAGGAGGAGGAACAGGAGGAGGAAGACGGCGCGGAGACCAAGGCT
AAAGACAAGACCACGGCCAAAGGGAAAGTAGGAGCAAAGAAGGCGACTACCAAGGAGAGCAAGCCCAAGAAAGAG
AAGGCCGGGAAAGACAAGAAAGACAAGAAGGACAAGACGGAAAAGAAGGTAAAGGATCCCAACGCCCCCAAGAGG
CCCCAGACAGCGTACCTTATCTTCGCAAATGAGCAACGTGAAACCATCAAGAAGGACAACCCCAGCTTTTCTCCG
CCAGACATACTGAAGGAAGCTGGAGCACGCTGGAAAGCGCTCACCGACAAGGAAAAGAGGAAATACCAGGATAAG
AATGAAATAGAGAAGCACAGATATAAGAAAGAAATGGAGGCCTATAATGCGAATGGTGGCGAGAAAGACACTGAT
AAGAAGGGTGCGAAGAGGAAGAAGGGCGAAGAGGAGGAAGAAGAGGACGAGGAAAACGCCAAGCCGAACAAGAAG
GCAGCCGCAGCAGACAAAGGCAAAGGCAAACCGAAGGGTCTCCTTCTCGCAAAAGCAGGGGCGAAAAAAAAGTAG
agtccacgaccgaagagtgaaggatgtaatttgccaccgacctctgacgaactaatacgcgtacgtgtacatact
atacaataaaaccagacttatattt

>Phypoly_transcript_14313_PpNHP6B
aaacgtcgtcaaagagccggcatgcgagcaaccctgccctgagcagcccgct***ATGGATACTCCCAAAGAGTGCCC***
TGCCAAGGAGGACGCCCCTGCTGAGAAGACCGAGCAGCCTGCGCCGGAGAAGAAGGAGGCCGAGAAGGAGGAAGG
GAAGGAAGGCAAAGACGAGGAGAAGGACGTCAAAGTTAGCGAGAGCGAGGAGGAGACCCTCAAACAAGAACCCAA

GAGAGCTCGCTCCGCGTACGCCATCTTCACTGCTGAGCAAGTGCCTATGGTTAAGAAGGAGCATCCCGATATGTC
TCGCACTGAGATAACGAAGTGTATTGCCGCGAAGTGGAAAGCCATGACCGCCGAGCAGAAGCAACCATTCGAAGA
GCAAGCAGTTAATGAGAAGAAGCGCATCAAGGAAGCCAAGGAAGCGGCAGCCAAGGAGGCCAAGGACAAGGAGGC
AGAGAAACCCGCGGAATCGGAGGTCCGCTATTCGCTGAGGAACTCGACCGTGATCAAGGACGGCGAGGAGCTCGT
GAACACGAAGAAGAGGGCGCTCGAGACCGAGGAGGACAGCGCGAACAAGAAGAAGTGCGTCACTTCGGAGGAGTA
Ggatggtaagcatgcgatattgcgaagcatgacgtcatatggcggcttgtttgaggtgcttagcgaagggagtcg
agatacacatgaggagaccgttggggagatactggtggaggatcgtgaagacttcttcaggcctcgtgaagggggg
agggcatgtcggagaacgcttttatcagcattgttattacattcaaagcgaggaaccggttttcatctggttcca
aaaccattacaaaggctgtcatcacattgcctagttgcgcacgtttccacacaacacaatgctttggatcaaata
gatttccttccagtggtaatctaaatgttccatcagtggccgcgtcaccagatccaccagctccactcgcgccac
ccatcccacccccctccgcctgccatcag

>Phypoly_transcript_15733_PpNHP6C
Cgtgaataccatatttgggttttgttgccgcgttcacctttagtgcataaggtctattgtggagcacttttgaat
tttcaaatttgaagttttgaattttgcgagagaattccgaaattcgtaaatcacgaaaattagatgcaaaagttg
acttttctaagaattttgtggtaccagtatttgaaaagtttatcatacagaagagaactttcccaaatattaatg
ttaatattattgtgatgttaggtttgttatttatagactgaaaatacaaatttgtgaaatcaaaaggaaaagaga
aaataaaaagaaggtgcgcgtatttcaaacaacacctttcctttgttctccttctctttttttgtacttttaccac
cc***ATGACAACCTTACACGAATGTCTGAAGACCCTGCTTGCTGATACCGAAAATGTAACACTCGACACAGAAGCTG***
AAATTCTATTGTACAACTTCGTTTCGTTTATTAGCACAAGAGGAACGAAAGGAAATAAAGAAAACACATACCCCA
CACCTATAAGTAGCGAAACAAGCTCACAAAATGAAGACGACTCGGATGAAGAGCCTACGCGAAAAACGAAAGGAA
CACCCATTAAAAGAAAAAAGGATCCTAATGCACCGAAAAGACCCCTGACAGCTTTTATGTTGTATGCACACGATA
AAAGAGCGGAAATCAAGGCACAAAACCCGGATATGACCTTCGGAGCGCTGGGCAAAAAACTGGGCGAGACGTGGC
TCACATTTGATGCCACACAAAAGAAGGTTTATTCCGATATGGCAGATAAGGCAAAATTAACCTACGAGAAGGAAT
TGGCAGAATACACAAAAAACAAACAAACATAGgagaaatggatgcaccaaatactgtaatattggatattacgga
tttgtttt

>Phypoly_transcript_17941_PpNHP6D
ccaccctagcatgtattaatagcacacctttccaccctccactcccgccatccagtacattctagccgtttgaag
ggggatagcttttccctccctttccccatacatttttttgcgtggcgctctcccacaaaatttttgtacggagcc
ccgtacaaaattcctgtgtggcacgctttcctttgttttgttcttctccttgtagcccctagcagtacctcctgc
tagacccctgctctccccttaagacatccctaacacatcaca**ATGTCATCTGACGAAGAATCCGCATCTTCCGCA**
TCTGAGGTTGAGGAAGTAGAGGAGAAGAAGAAGAGAGGACCGAAAAAGGGCGCTAAGAAGGCAGAGAAAACGAAA
AAAGCCACCAAAAAAGTAGCCAAAAAGAAGAAGGATCCCAATGCTCCTAAGCGCCCTCTCTCAGCCTTCATGTAC
TACTCCCAGAAGCAGAGACCCAATGTCAAGAAGGAGAACCCCTCCGCCACCTTTGCTGATGTGGCCAGGATCCTT
GGTGAGCAATGGAAGGCGCTCGATGCCAGCCTCAAGAAGGAATACGAGAAACAAGCCGCAACAGACAAGGCTCGT
TGGGAGAAAGAAAAGGCTGCCTACGATGGAGGAAGCAGCAAGGGAGGAAAGAAGAAGGCAGCAGGAAGTGACGAC
GAGGAGGAAGAGTCTGGATCCGACGAATAGacgagtcaacaataggaagtttaagtgtaccattacttactgcat
ctcccaaacaactttttttgtgtaataataataacaat

>Phypoly_transcript_18969_PpNHP6E
ta***ATGGTCCGCTTCCCTTCATATTTTGGCCCACAGGATTTTGAATTCCAAAAAGGAAAGCGGGCACGGCCTTATT***
TCAAACGGAAACGTTTACTTAGCCGTTGTCCGCTTTTTGTATTTCAAACTACGCGAACGCACTCGCAACACTCGC
AAGAACACAAAATGGTAGCGAAGAAGGAAAAGAAAGAAGCTGTCGAGGGAGAGCGCAAGTCCTCCCGCACCTCCA
AGCAGGTGGAGCGCCTCAGCGTAAGCCCCCCAACCATCCTCAAGAAGAAGGCCACCAAGAAGTCCACCACGGCTT
CCAAGACCAAGTCGCCCAAGAAGGCTGGAGCCAAGAAGGGTACCAAAAAGGCCAAGGGAGAGAAAGGCCCCAAGC
GCCCAGCGAGTGCTTATCTGCTCTTCGCCAACGAGAACCGTGCTAAGGTCAAGAAAGCCAACCCTGATGCTACCT
TTGGGGAGCTGGGCAAACTCCTGGGTGAGGCATGGAGCAACGCCACTGCTGCAGACAAGAAGAAATTCACCGCTT
TGGCCGACAAGGACAAAGCTAGATATGAGAAAGAAAAGGCTGCTCTCGCATAAatgcgacgacgctaatgcagcg
gtctatggattaccactgtgcgggcttttacaagcgtgtagtattatgtattataaataaattttctttgctgta
aaaaaaaaaaaaaaaaaaaaaaaaaaaaaaaaaaaaaaaaaaaaaaaaaaaaaaaaaaaaaggcggcc

2. Detailed analysis of the chaperone structure

2.1. The H3/H4 chaperones involved before chromatin incorporation

PpHSP90 harbors 4 domains: (i) the N-terminal ATP-binding domain with high conservation, (ii) the glutamic acid-rich (20% in the PpHSP90 linker) and charged linker that is less conserved, the highly conserved (iii) middle and (iv) C-terminal domains (Figure 3A). **HSC70** interacts with H3 histones right after their ribosome exit as well as with H4 and HSP90 [5,6]. As other HSC70s, PpHSC70 presents the 3 general structural domains (Figure 3B): (i) a NBD (N-terminal Nucleotide Binding) domain containing an ATPase domain, (ii) a SBD (Substrate-Binding Domain) domain divided in SBDβ and SBDα, the latter acting as a flexible lid and (iii) a flexible linker connecting NBD and SBD [96]. Overall, the HSC70 is well conserved across species, SBDα being more divergent (Figure S6F). In PpHSC70, we noticed the presence of the EEVD motif (Figure S3F, red box) involved co-chaperone binding [96].

The **NASP** proteins frequently present two isoforms corresponding to splice variants but *P. polycephalum* only present one splice variant and only one NASP orthologue is present in studied organisms excepted in *C. elegans* which contain two paralogues. NASP is a protein that bound H3/H4 constituting a reservoir of soluble H3 and H4. The PpNASP protein presents 4 TPR and one SHNi-TPR (Sim3-Hif1-NASP interrupted TPR) repeats as well as a "E/D-rich" region (Figure 3C). TPRs are involved in H1 and H3/H4 binding [25,26]. Moreover, PpNASPs presents an "E/D-rich" region that interrupts the TPR2 and is involved in H1 binding [25], this region being larger in human (Figure 3C) and mice NASPs. Besides, PpNASP presents a well conserved SHNi-TPR (Sim3-Hif1-NASP interrupted TPR).

The nuclear translocation protein **IPO4** is associated with the cytosolic multiprotein complex involved in H3.1/H4 dimer nuclear import [7]. Another model has been proposed in which H3 and H4 are bound as monomers to IPO4 in the cytosol before being translocated to the nucleus and being addressed in the nucsleus as dimers by the NASP, ASF1, p46^{RbAp46} and HAT1 complex [97]. Excepted *Drosophila* that presents tandem duplicate genes giving rise to Artemis and Apollo proteins [98], *Physarum* and studied organisms present one single IPO4. PpIPO4 contains an importin-β N-terminal domain and two HEAT repeats (Figure 3D). Acetylation of K5 and K14 residues of the histone H4 increased affinity with IPO4 [99] and facilitates histone nuclear import, notably in *P. polycephalum* [8] and in yeast [100].

The **ASF1** is highly conserved from yeast to mammals. Two isoforms exist in mammals and plants but *P. polycephalum* contains only one isoform similarly to yeast or Drosophila. Alignment of ASF1 proteins confirmed that the N-terminal is highly conserved (50-60% of identity regarding the first 155 amino acids) while the C-terminal is rather divergent and smaller than in yeast similarly to other

metazoans (Figure S6). Moreover, the C-terminal contains several acidic amino acids - mainly glutamate - as it was reported in other organisms [101] but too a smaller extent.

2.2. The H3/H4 chaperones involved in chromatin incorporation

The CAF-1 complex comprising 3 subunits, named PpCAF1A, PpCAF1B and PpCAF1C in *Physarum*. **PpCAF1A** harbors 4 domains: (i) the A domain involved in ScCac3 interaction and flanked by (ii) the "E/D-rich" domain enriched in glutamic acid (E) and aspartic acid (D) involved in H3/H4 interaction, (iii) the KER domain rich in lysine (K), glutamic acid (E) and arginine (R) involved in ScCac2 interaction and (iv) a PIP2 domain (Figure 4A). Besides these domains, Hs-p150 also presents PEST, PIP1 and C-terminal domains as well as the HP1 motif. The PEST domain - rich in proline (P), glutamic acid (E), serine (S) and threonine (T) [102]- is absent in *P. polycephalum,* yeast and plant proteins (Figure 4A). The CAF-1 large subunit associates *via* the PIP domain (PCNA-Interacting Peptide) with PCNA (Proliferating Cell Nuclear Antigen) which triggers CAF-1 recruitment to the replicating sites [102]. While animal CAF1A proteins display two PIPs (PIP1 in the N-terminal region and PIP2 in the KER domain, proteins from the other analyzed organisms only present PIP2 (Figure 4A). Besides, Hs-p150 and ScCac1, respectively harbor a p60-binding and a WHD (Winged Helix domain) involved in DNA binding without sequence specificity [103,104] in their C-termini (Figure 4A). **PpCAF1B** harbors several domains: (i) WD40 repeats involved in the interaction between the middle subunit of CAF-1 and the H3/H4 histones and ASF1 [103] and in the interaction with the large subunit of CAF-1 [105], (ii) a PEST domain comprising a B-like similar domain to the HIRA B domain and (iii) a PIP domain. WD40 repeats are clustered at the N-terminal except one in the C-terminal region for PpCAF1B, ScCac2 and AtFAS2 (Figure 4B). Most animal CAF1B proteins contain two B-like domains (B-like 1 and B-like 2) located in the middle and C-terminal regions of the PEST domain for Hs-p60 while *Physarum*, yeast and plants only display B-like 2 (Figure 4B). **PpCAF1C** harbors 2 domains: (i) two H4-interacting regions located at the N- and C-termini and (ii) several WD40 repeats in the internal region (Figure 4C).

The HIR complex comprising 3 subunits, named PpHIRA, PpUBN and PpCABIN in *Physarum*. **PpHIRA** presents three domains: (i) the N-terminal region composed of several WD40 repeats involved in the UBN1 interaction [106], (ii) the B domain involved in ASF1 interaction and (iii) the HIRA domain involved in CABIN1 binding [33] and HIRA homooligomerization [107] (Figure 4D). The B-domain and the HIRA domain are well conserved (Figure S8B-C). One should note that HIRA protein has two orthologues in yeast, ScHIR1 and ScHIR2 respectively homologous to the N- and C-terminal regions of HsHIRA (Figure 4D). Besides, HsHIRA was also reported to bind H2B and H4 [108]. **PpUBN** presents three domains: (i) a N-terminal domain named NHRD (N-terminal to HRD -

Hpc2p-Related Domain), the (ii) HRD and (iii) ubinuclein middle domains (Figure 4E). UBN proteins present similar domain organization across eukaryotes, except for ScHpc2; the human UBNs display longer C-termini (Figure 4E). The NHRD domain mediates HsHIRA interaction [106,109] through 5 keys residues (R46/E53/K74 conserved in most species but E61/F62 only conserved in animals; Figure S8D, in blue). NHRD and HRD domains are located in the N-terminal region of UBN proteins excepted for ScHpc2 in which they localize in the C-terminal (Figure 4E). The HRD domain confers the H3.3/H4 specificity to the HIR complex whereas the ubinuclein middle domain is able to bind both H3.1/H4 and H3.3/4 [32,110]. HsUBN1 also presents a DNA-binding domain which participates in HIR-mediated histone deposition in chromatin [111]. We did not find this lysine-rich domain in PpUBN. As other UBN, PpUBN presents a ubinuclein middle domain which is absent from ScHpc2 and DdUBN (Figure 4E). **PpCABIN1** presents several TPR and TPR-like repeats and HsCABIN1 a MEF2-binding domain (Figure 4F). These TPRs are sufficient for the HsCABIN1-HsHIRA interaction [18]. This MEF2-binding domain was retrieved only in animal CABIN1 (CePICD-1 excepted). The TPRs are sufficient for the HsCABIN1-HsHIRA interaction [33] and HsCABIN1 and HsUBN1 can interact together [109].

2.3. The H2A/H2B chaperones

Proteins of the **NAP** family have various functions beyond their roles in chromatin assembly. ScNAP1 participate to the H2A.Z/H2B variant exchange [15] and AtNRP proteins counteract SWR-C activity in Arabidopsis [111]. More generally, NAP proteins are able to bind H2A/H2B and H3/H4 histones (reviewed in [13]). **PpSET** and **PpNAP1L1** harbor a N-terminal tail of variable length and an acidic C-terminal tail as most NAP proteins. Sequences participating to the NAP hydrophobic core and protein assembly [13] are highly conserved in both *Physarum* NAPs (Figure S9A-B). Finally, the NES and its key residues as well as the NLS [112,35] are conserved only in PpNAP1L1 (Figure S9H-I, Tables S5 and S6). The **FACT** complex is composed of two subunits SPT16 and SSRP1 (or ScPob3) and contributes to chromatin dynamics during transcription, DNA repair and replication. This heterodimer deposits H2A/H2B dimers, H2A.X/H2B dimers and H3/H4 tetramers into chromatin but not H2A.Z/H2B [19]. It also disrupts interactions between core histones as well as DNA/histones interaction, notably during replication. It is thus able to bind either nucleosomes or free histones. **PpSPT16** present four domains: the (i) N-terminal, (ii) dimerization, (iii) middle and (iv) C-terminal domains (Figure 5B), based on the homology with HsSPT16 [113]. N-terminal domains are conserved across species (Figure S10A) and present an aminopeptidase-like "pita bread" fold [114]. Moreover, they are binding modules for H3/H4 in fission yeast [115] and human [116]. The SPT16 dimerization domain enables formation of the FACT complex through an interaction with the N-

terminal/dimerization domain of SSRP1 [117] and presents some similarities across studied species (Figure S10B). The middle domain presents a double pleckstrin homology structure in yeast that is able to bind H3/H4 [39] during nucleosome dynamics operated by FACT and it is conserved across studied species (Figure S10C). Besides, the H2A/H2B minimal binding domain defined in ScSPT16 [39] is poorly conserved across species (Figure S10D, green square) but its key residues are either conserved or substituted by an amino acid of similar properties in animals and *Physarum* (Figure S10D). Finally, the C-terminal domain is an intrinsically disordered region involved in mononucleosome binding and weakening of interactions between H2A/H2B dimers and H3/H4 tetramers [118]. **PpPob3** is the orthologue of ScPob3, the second yeast FACT subunit. SSRP1 proteins display 5 domains: (i) a N-terminal/dimerization domain with a single pleckstrin homology structure [113] involved in dimerization with SPT16 [117], (ii) a middle domain with a double pleckstrin homology structure [113] involved in DNA binding [119], (iii) an HMG (high-mobility group) domain flanked by two intrinsically disordered regions, the (iv) IDD (Intrinsically DisorDered) and (v) C-terminal regions (Figure 5C). However, ScPob3 and PpPob3 presents only three domains (Figure 5C) similarly to *T. thermophila* and *D. discoideum* [37] orthologues. The N-terminal region of the HMG domain is involved in DNA binding in human [120] and yeast [121].

2.4. The DNA replication-associated histone chaperones

MCM2 is a component of the DNA replicative helicase MCM2-7 complex. This complex is loaded on double-stranded DNA during late M and G1 phases and participates to DNA unwinding during replication. Moreover, MCM2 can associate with H3/H4-ASF1 during replication [12] and with the histones-FACT complex during transcription [40]. Besides, MCM2 can bind all H3 isoforms (H3.1, H3.3 and CENP-A) [41]. We analyzed the amino acid composition of PpMCM2. The PpMCM2 protein displays an acidic tail (33% aspartate and glutamate in the first 186 amino acids) as the other eukaryotic MCM2 orthologues (~30% for the human, yeast or Arabidopsis MCM2; [40]). PpMCM2 displays five domains: (i) a HBD (histone binding domain), (ii) MCM N-terminal, (iii) OB(oligonucleotide/oligosaccharide)-fold, (iv) MCM and (v) AAA+ that confers the ATPase activity (Figure 5E). The DNA polymerase ε also named **PolE** is responsible for DNA synthesis on the leading strand during replication. PolE3 and PolE4 are its small subunits. HsPolE3 and HsPolE4 promote H3/H4 dimers and tetramers deposition into chromatin during replication [122]. ScDPB4, the yeast PolE3 orthologue, interacts with the histone chaperones ScHIR1 and ScNAP1 and is involved in heterochromatin silencing [123]. PpPolE3 presents a histone-like fold (Figure 5F) with 4 helices similar to H2A and H2B ones and an acidic C-terminal (Figure S13A).

2.5. The chaperones associated with histone recycling and exchange

SPT6 is present as one isoform in most organisms such as *Physarum* but Arabidopsis presents two isoforms, AtSPT6 and AtSPT6L. The former presents a very low expression level in most tissues while the later displays a wide expression [124] and its chromatin localization was studied [125]. For this reason, we only included AtSPT6L in our study. PpSPT6 presents 8 domains. Interproscan did not retrieve the HtH DNA-binding domain in the Arabidopsis protein (Figure 6A, [125]). The N-terminal region of SPT6 is involved in histone interaction. The core region is composed of: (i) 3 DNA-binding domains HtH (Helix-turn-Helix), HhH (Helix-hairpin-Helix) and HHH domain 9, (ii) the YqgF domain from the ribonuclease family and (iii) the S1 RNA-binding domain (Figure 6A). The C-terminal region of SPT6 is involved in the binding to the Ser2-phosphorylated RNA Polymerase II thanks to the SH2 (Src-homology 2) domain. Moreover, AtSPT6L presents a plant-specific WG/GW domain required for Argonaute interaction and small RNA-mediated gene silencing [126] also present in PpSPT6 (Figure 6A). To perform chromatin remodeling, substitution of nucleosomal canonical histones by histone variants, the so-called "histone replacement", takes place. The H2A.Z/H2B variant exchange is performed by the **SWR-C** complex [15,14,16-18] which destabilize the chromatin to allow the ATP-dependent substitution of H2A/H2B dimers by H2A.Z/H2B dimers. **PpSWR1** is the *Physarum* orthologue of ScSWR1, DmDomino [127] or AtPIE1 (Photoperiod-Independent Early flowering 1, [128]). To note, two orthologues ScSWR1 have been identified in human, HsSRCAP (Snf2-Related CBP Activator Protein; [129]) and Hs-p400 (400KDa protein, [130]) as well as in mouse, fish and xenopus. Several members of the SWI2/SNF2 protein family were first identified from genetic studies conducted in *S. cerevisiae* (SWI refers to yeast mating type *swi*tching, while SNF is an abbreviation for Sucrose Non Fermenting). These proteins behave as chromatin remodelers and display ATPase and Helicase activities. **PpSWR1** presents 4 characteristic domains (Figure 6B): (i) a HSA (Helicase/SANT-associated) domain conserved in studied orthologues; an ATPase domain split in two segments (ii) SNF2 and (iii) Helicase domains, both being highly conserved and responsible for SWC2 binding [131,45]; (iv) a well-conserved SANT (Swi3/Ada2/N-Cor/ TFIIIB) domain in the C terminal. The HSA domain is located at the N-termini of most proteins excepted for p400 ones which harbor a longer N terminal (Figure 6B). The AtPIE1 N-terminal region binds to H2A.Z but not canonical H2A [18] and the N-terminal region of ScSWR1 is responsible for H2A.Z binding [45] but residues identified in yeast for their role in H2A.Z binding [46] are not conserved across studied species. Besides, some SWR1 orthologues present a SANT domain while SCRAP, yeast, *C. elegans, T. thermophila, A. trichopoda* and maize proteins do not (Figure 6B and S15D). Instead, SRAP proteins have A/T-hook motifs and harbor a long spacer between the SNF2 and Helicase domains (Figure 6B; [132]). Moreover, p400, DmDomino

and DdSWR1 proteins present polyglutamines in their C-termini downstream of the SANT domain (Figure 6B) while PpSWR1, AtPIE1 and PpaPIE1 do not. **PpSWC2** is the *Physarum* orthologue of ScSWC2 and of animal YL1 proteins, which are present in both SRCAP and p400 complexes [16]. The ScSWC2 N-terminal region binds H2A.Z [57] and is responsible for DNA recognition [133]. Similar to ScSWC2, PpSWC2 contains ~40% of charged residues. PpSWC2 presents N- and C-terminal regions characteristic of SWC2 proteins as well as the Z domain (Figure 6C). **APLF** belongs to the Aprataxin and PNK (polynucleotide kinase) family which are DNA-break response factors. This family is characterized by the presence of a FHA (ForkHead-Associated) domain [134] which interacts with the DNA-damage repair facilitators XRCC1 (X-Ray Cross Complementing group 1) and XRCC4. PpAPLF presents a single PBZ located at the very end of the protein while HSAPLF has tandem PBZ domains (Figure 6D).

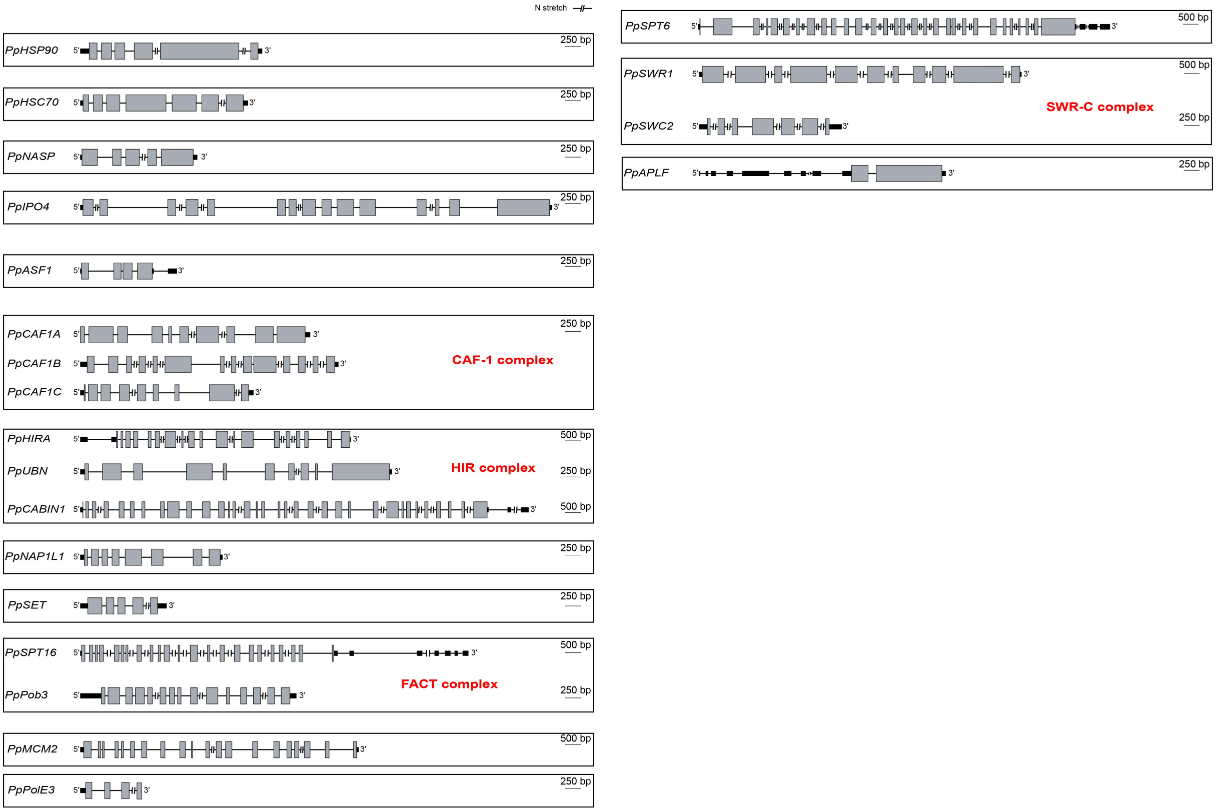

Figure S1. Structure of the various *Physarum* histone chaperone genes. Exons, grey rectangles; untranslated regions, black rectangles. Presence of N stretches are indicated by an interrupted line as indicated on the top right of the figure.

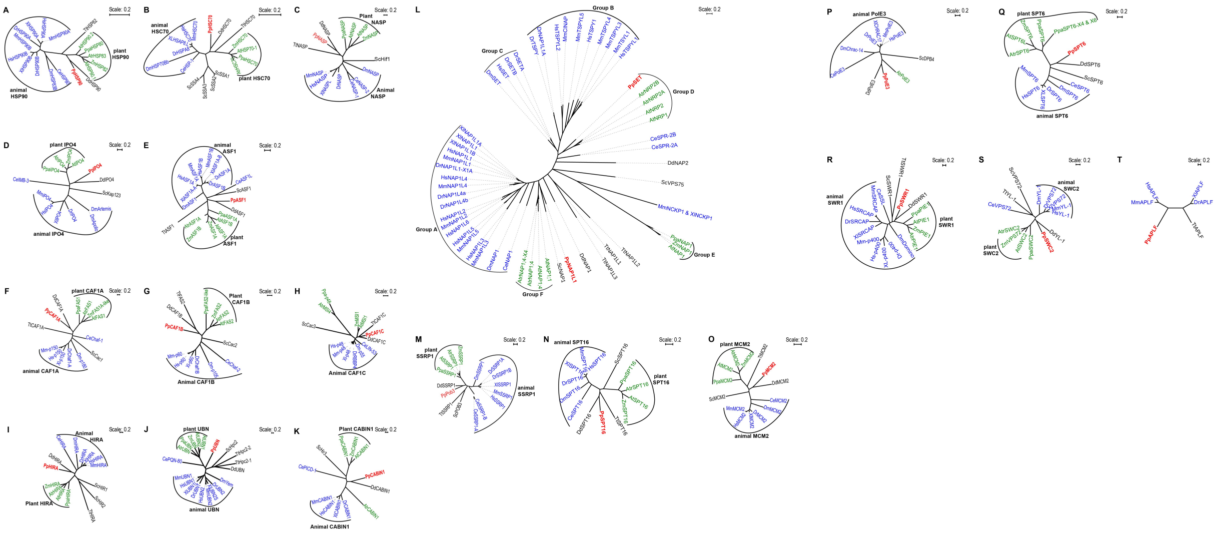

Figure S2. Phylogenetic trees of *Physarum* histone chaperones. Phylogenetic tree of the chaperones HSP90 (**A**), HSC70 (**B**), NASP (**C**), IPO4 (**D**), ASF1 (**E**), CAF1A (**F**), CAF1B (**G**), CAF1C (**H**), HIRA (**I**), UBN (**J**), CABIN1 (**K**), of the NAP family (**L**), SSRP1 (**M**), SPT16 (**N**), MCM2 (**O**), PolE3 (**P**), SPT6 (**Q**), SWR1 (**R**), SWC2 (**S**) and APLF (**T**) from *P. polycephalum* and various eukaryotes. The NAP family (**L**) is very large and displays a complicated phylogenetic history. Given the high number of NAP proteins in the selected organisms, we condensed highly similar copies in each of the species (between 92 to 99% of identity over the whole protein). All the animal NAP protein cluster together into a group that we named group A, the ScVPS75 protein being found in this group. The animal TSPY proteins cluster into a group that we named group B. The SET chaperones constitute a single clade that we name group C, excepted for PpSET which belongs to the same clade than the plant NRP proteins that are closely related to HsSET. The plant kingdom presents three groups of NAP proteins: (i) group D comprising the NRP proteins and PpSET, this *Physarum* protein presenting a longer branch suggesting a lower homology with the plant NRPs; (ii) group E comprising three proteins and arising from the same clade than the mouse and Xenopus NCPK1 but the long branches suggested a lower homology between them; (iii) group F comprising four plant proteins and PpNAP1L1 presenting a longer branch suggesting a lower homology. The three *T. thermophila* NAP proteins group together in a single clade as well as the *C. elegans* SPR proteins. We found two NAP protein in *D. discoideum* and both do not belong to any other clade and are not evolutionary related to each other. Atr: *Amborella trichopoda*; At: *Arabidopsis thaliana*; Ce: *Caenorhabditis elegans*; Dd: *Dictyostellium discoideum*; Dr: *Danio rerio*; Dm: *Drosophila melanogaster*; Hs: *Homo sapiens*; Mm: *Mus musculus*; Pp: *Physarum polycephalum*; Ppa: *Physcomitrella patens*, Sc: *Saccharomyces cerevisiae*; Tt: *Tetrahymena thermophila*; Zm: *Zea mays*.

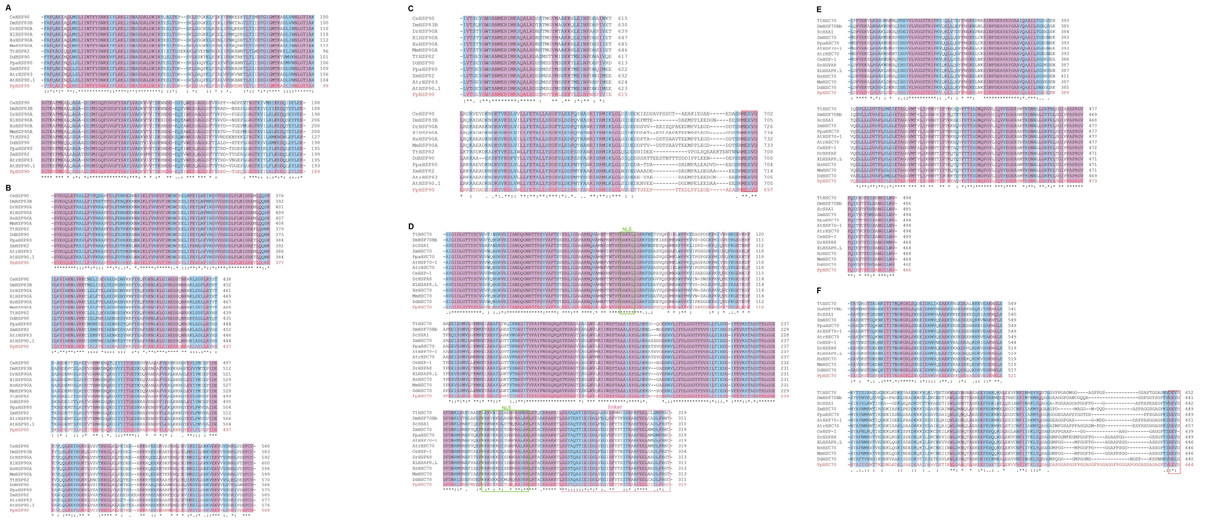

Figure S3. Protein alignments for the *Physarum* HSP90 and HSC70 heat shock proteins. **a to c.** Alignments correspond to the N-terminal (**A**), middle (**B**) and C-terminal (**C**) domains for the HSP90 proteins. The C-terminal region of PpHSP90 contains a strictly conserved MEEVD motif responsible for interaction with TPR-containing proteins [135] (**C**) which is displayed by a red box. Alignments are performed with HSP90 proteins used in the phylogenetic tree reported in Figure S2A, only one HSP90 protein per organism being conserved for an easier reading. **D** to **F.** Alignments correspond to the NBD and linker domains (**D**) and to the SDBβ (**E**) and SDB α (**F**) domains for the HSC70 proteins. Two NLS (Nuclear Localization Signal) were reported in HsHSP70 [136], the first only being strictly conserved while the second one is less: both NLS signals are indicated by a green rectangle and the linker by a pink rectangle (**D**). The HSC70 EEVD motif involved co-chaperone binding is displayed by a red rectangle (**F**). Alignments are performed with HSC70 proteins used in the phylogenetic tree reported in Figure S2B, only ScSSA1 being used among yeast homologues for an easier reading. The *Physarum* proteins appears in red. Positions refer to the mature protein with the initial methionine. Asterisk indicates a fully conserved residue labelled with a pale pink rectangle. Colon and dot indicate conservation between residues of, respectively, strongly and weak similar properties and are labelled with a pale blue rectangle. Atr: *Amborella trichopoda;* At: *Arabidopsis thaliana*; Ce: *Caenorhabditis elegans;* Dd: *Dictyostellium discoideum*; Dr: *Danio rerio;* Dm: *Drosophila melanogaster*; Hs: *Homo sapiens*; Mm: *Mus musculus*; Pp: *Physarum* polycephalum; Ppa: *Physcomitrella patens*, Sc: *Saccharomyces cerevisiae;* Tt: *Tetrahymena thermophila*; Zm: *Zea mays*.

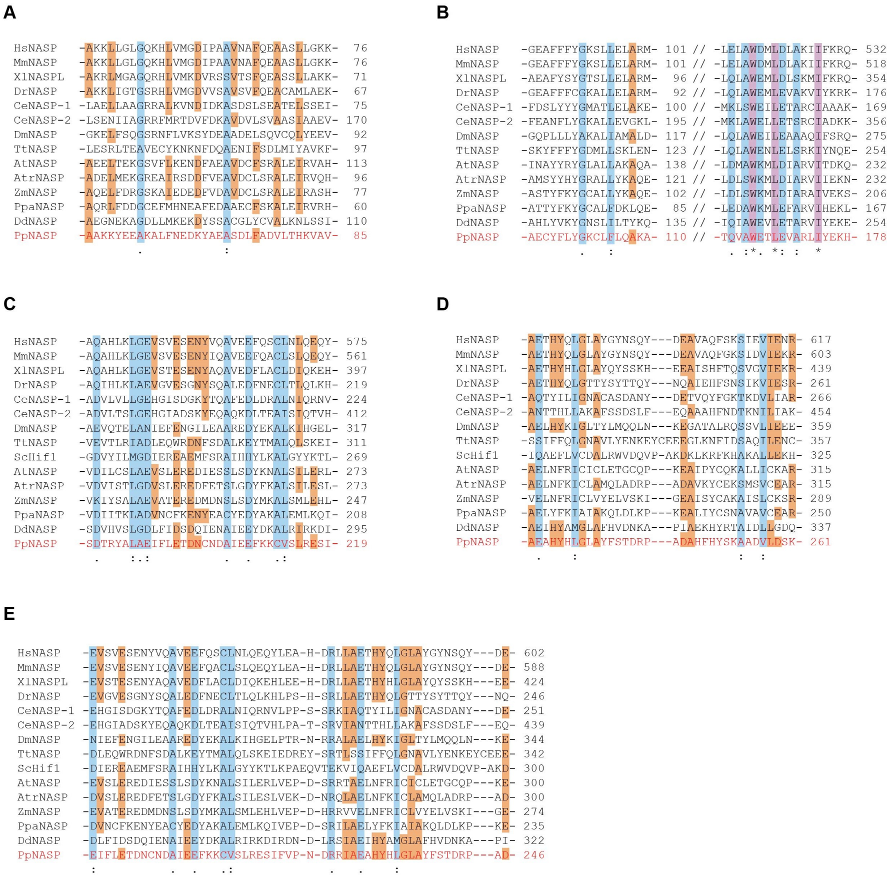

Figure S4. Protein alignments for the *Physarum* NASP protein. Alignments correspond to the TPR1 (**A**), TPR2 (**B**), TPR3 (**C**), TPR4 (**D**) and SHNi-TPR (**E**) repeats for the NASP proteins. Several TPR residues were previously described to be almost unchanged through evolution [137] which we could also observe. Alignments are performed with NASP proteins used in the phylogenetic tree reported in Figure 2C. ScHif1 was removed from TPR1 and TPR2 for an easier reading. For the interrupted TPR2, the TPR2-1 and TPR2-2 alignments are displayed together and separated by a double slash. The *Physarum* protein appears in red. Positions refer to the mature protein with the initial methionine. Asterisk indicates a fully conserved residue labelled with a pale pink rectangle. Colon and dot indicate conservation between residues of, respectively, strongly and weak similar properties and are labelled with a pale blue rectangle. Almost conserved residues are labelled by an orange rectangle. Atr: *Amborella trichopoda;* At: *Arabidopsis thaliana*; Ce: *Caenorhabditis elegans;* Dd: *Dictyostellium discoideum;* Dr: *Danio rerio;* Dm: *Drosophila melanogaster*; Hs: *Homo sapiens*; Mm: *Mus musculus*; Pp: *Physarum* polycephalum; Ppa: *Physcomitrella patens*, Sc: *Saccharomyces cerevisiae;* Tt: *Tetrahymena thermophila*; Zm: *Zea mays*.

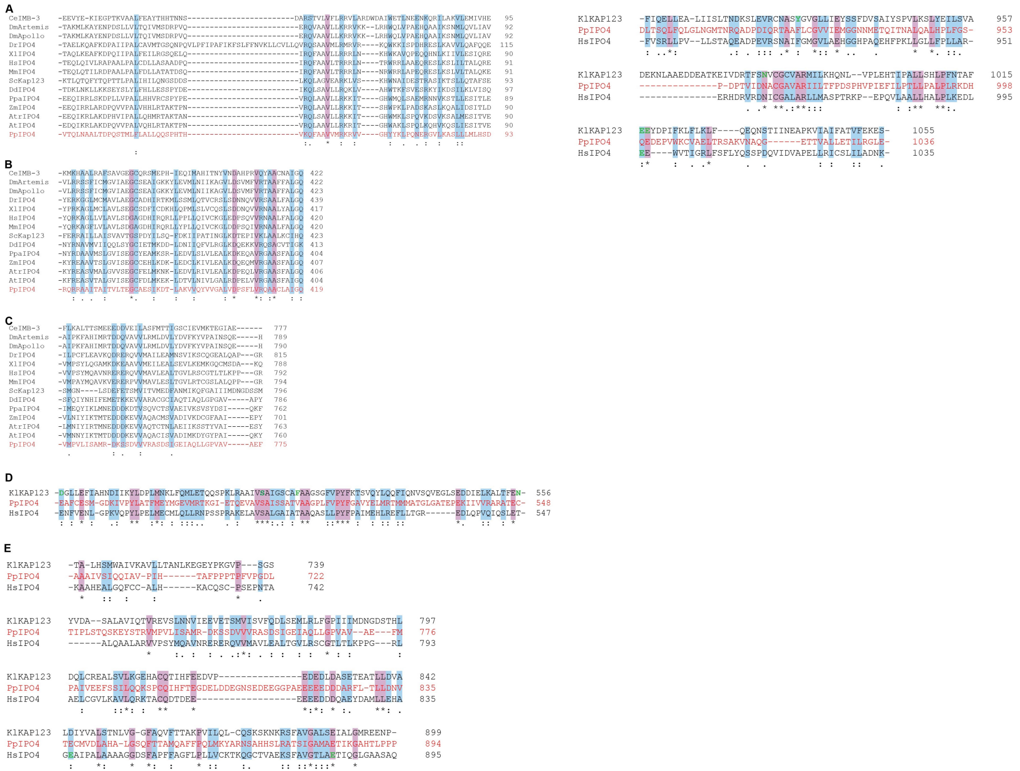

Figure S5. Protein alignments for the *Physarum* IPO4 protein. **A.** Alignment correspond to the importin-β N-terminal domain for the IPO4 proteins. **B-C.** Alignment correspond to the HEAT repeats 1 (**B**) and 2 (**C**) for the IPO4 proteins. **D-E.** Alignment correspond to the two H3 and H4 lysine-binding pockets from IPO4^{Kap123} of *Kluyveromyces lactis* and HsIPO4 that recognize histone nuclear localization sequences [100,138]. Some residues (F512 and Y926, respectively, in KlKap123 first and second pockets) form hydrophobic interactions while others (D465/S505/S509/N556 and N980/E1016/E1017, respectively, in first and second pockets) perform electrostatic/hydrogen interactions [100]. In human, HsIPO4 residues E997 (E1016 in KlKap123), E884 (E988 in KlKap123) and E837 (D844 in KlKap123) are required for IPO4 binding to the histone H3 tail [138]. These key residues are depicted in green (**D-E**). Alignments are performed with IPO4 proteins used in the phylogenetic tree reported in Figure S2D. The *Physarum* proteins appears in red. Positions refer to the mature protein with the initial methionine. Asterisk indicates a fully conserved residue labelled with a pale pink rectangle. Colon and dot indicate conservation between residues of, respectively, strongly and weak similar properties and are labelled with a pale blue rectangle. Atr: *Amborella trichopoda;* At: *Arabidopsis thaliana*; Ce: *Caenorhabditis elegans;* Dd: *Dictyostellium discoideum*; Dr: *Danio rerio;* Dm: *Drosophila melanogaster*; Hs: *Homo sapiens*; Mm: *Mus musculus*; Pp: *Physarum* polycephalum; Ppa: *Physcomitrella patens*, Sc: *Saccharomyces cerevisiae;* Tt: *Tetrahymena thermophila*; Zm: *Zea mays.*

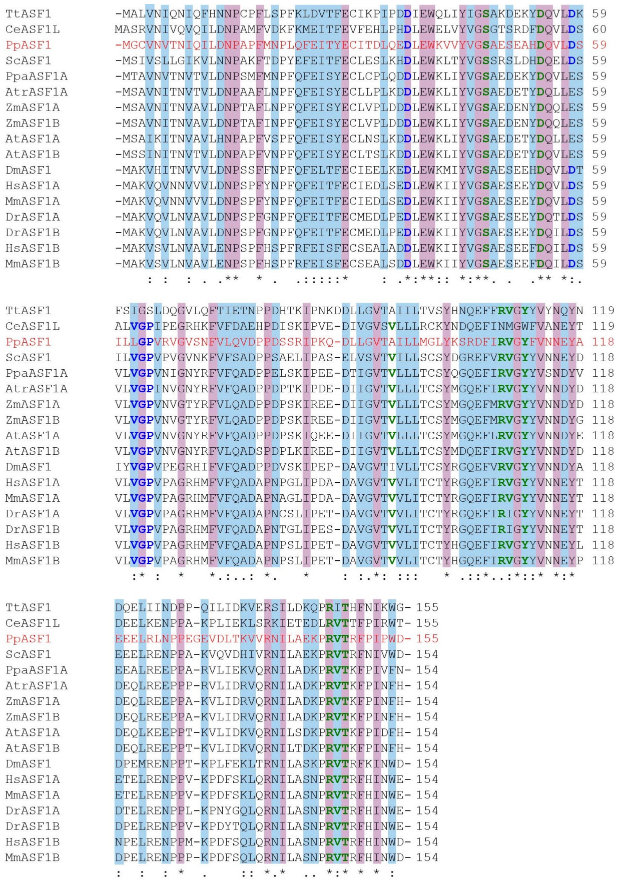

Figure S6. Protein alignment for the *Physarum* ASF1 histone chaperone. Alignment is performed with ASF1 proteins used in the phylogenetic tree reported in Figure S2E. The C-terminal of PpASF1 contains several acidic amino acids - mainly glutamate - as it was reported in yeast [101] but too a smaller extent. Two aspartate residues (D37 and D58) and a VGP triplet are crucial for the HsHIRA-HsASF1 heterodimer formation [9]: they are reported in blue. Both aspartates are conserved in PpASF1 while the VGP triplet (residues 62-64, [29]) is a LGP triplet in PpASF1 as well as in DdASF1. Since valine and leucine are both branched-chain amino acids, the V-L substitution should not disrupt the association of PpASF1 with the other chaperones. Mutations reported to affect the ASF1 binding to H3/H4 [27,28] are reported in green). They are all conserved in PpASF1 excepted V94-ScASF1 which is replaced by an alanine that is also hydrophobic. The *Physarum* protein appears in red. Positions refer to the mature protein with the initial methionine. Asterisk indicates a fully conserved residue labelled with a pale pink rectangle. Colon and dot indicate conservation between residues of, respectively, strongly and weak similar properties and are labelled with a pale blue rectangle. Key residues for ASF1 binding to H3/H4 and HIRA are, respectively, depicted in green and in blue. Atr: *Amborella trichopoda;* At: *Arabidopsis thaliana*; Ce: *Caenorhabditis elegans;* Dd: *Dictyostellium discoideum;* Dr: *Danio rerio;* Dm: *Drosophila melanogaster*; Hs: *Homo sapiens*; Mm: *Mus musculus*; Pp: *Physarum* polycephalum; Ppa: *Physcomitrella patens*, Sc: *Saccharomyces cerevisiae;* Tt: *Tetrahymena thermophila*; Zm: *Zea mays*.

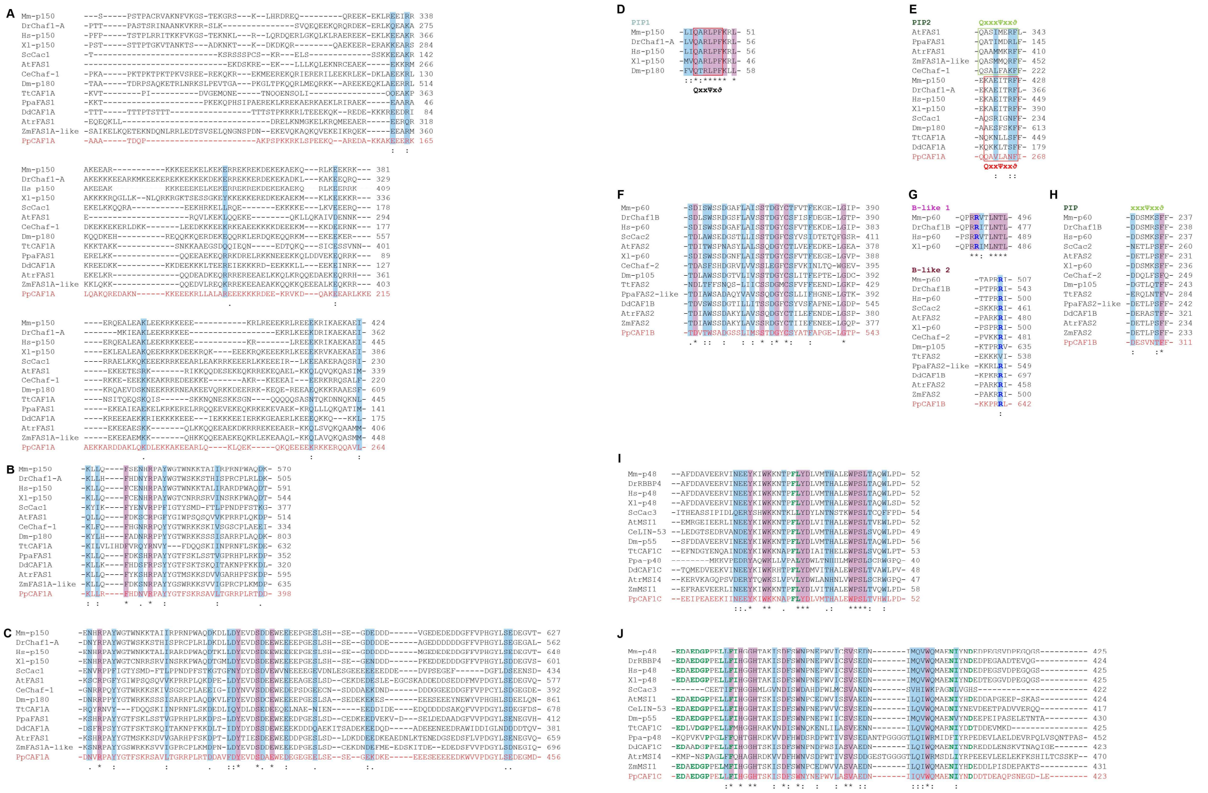

Figure S7. Protein alignments for the *Physarum* CAF-1 complex. **A** to **E.** CAF1A Alignments correspond to the KER domain (**A**) involved in the interaction with ScCac2, the A domain (**B**) involved in the interaction with ScCac3, the E/D rich region (**C**) involved in the interaction with H3/H4 [139], the PIP1 (**D**) and PIP2 (**E**) boxes. Alignments are performed with CAF1A proteins used in the phylogenetic tree reported in Figure S2F. **F** to **H.** CAF1B alignments correspond to the PEST domain (**F**) which is strongly conserved for the studied organisms, the B-like1 (top) and B-like 2 (bottom) domains with the key residues for CAF1B binding to ASF1 depicted in blue (**G**), the PIP box (**H**). Residues R483 and R499 from Hs-p60, respectively located in the B-like 1 and 2 domains, are involved in the interaction between Hs-p60 and ASF1 [9]: these residues are displayed in blue in **g** and both are highly conserved. The PIP domain of PpCAF1B is more degenerated in sequence compared to the ones in CAF1A proteins (**G**). Alignments are performed with CAF1B proteins used in the phylogenetic tree reported in Figure S2G. **I-J.** CAF1C alignments for the CAF1C proteins correspond to the N- (**I**) and C-terminal H4-interacting regions (**J**) with the key residues for CAF1C binding to H4 depicted in green. Based on the crystal structure, several residues were previously identified for their role in the interaction with the histone H4 (F29/L30 and F367/I368 in **i** and I407 of the Hs-p48 hydrophobic patch in **j**; E356/ D357/ E359/ D360/ G361/ P362/ L365/ N406/I407/ D410 in **j** that form hydrogen bonds with specific residues of H4). These residues are displayed in green (**I** and **J**) and they all are strictly conserved in PpCAF1C. Alignments are performed with CAF1C proteins used in the phylogenetic tree reported in Figure S2H. The *Physarum* proteins appear in red. Positions refer to the mature protein

with the initial methionine. Asterisk indicates a fully conserved residue labelled with a pale pink rectangle. Colon and dot indicate conservation between residues of, respectively, strongly and weak similar properties and are labelled with a pale blue rectangle. Atr: *Amborella trichopoda;* At: *Arabidopsis thaliana*; Ce: *Caenorhabditis elegans;* Dd: *Dictyostellium discoideum*; Dr: *Danio rerio;* Dm: *Drosophila melanogaster*; Hs: *Homo sapiens*; Mm: *Mus musculus*; Pp: *Physarum* polycephalum; Ppa: *Physcomitrella patens*, Sc: *Saccharomyces cerevisiae;* Tt: *Tetrahymena thermophila*; Zm: *Zea mays*.

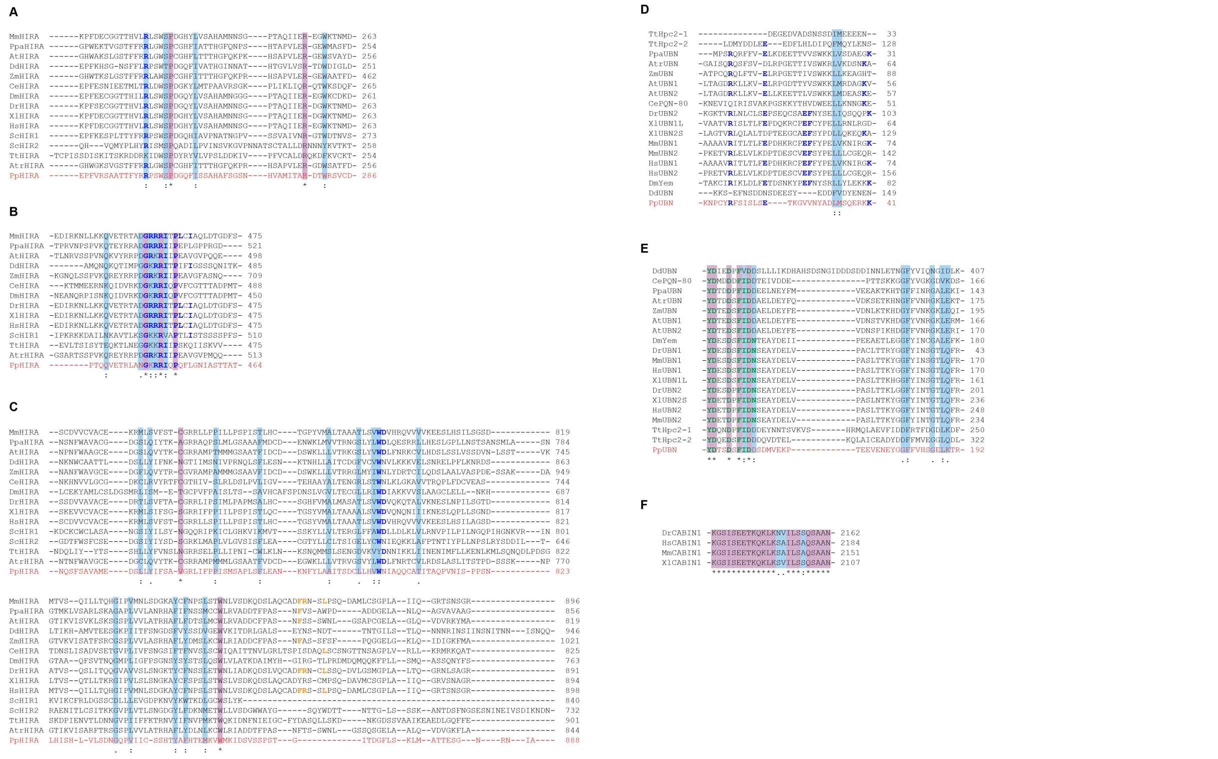

Figure S8. Protein alignments for the *Physarum* HIR complex. **A.** Alignment correspond to the N-terminal domain for the HIRA proteins. The R227-HsHIRA residue is involved in HsHIRA-HsUBN1 interaction [106]. This residue is displayed in blue and highly conserved. **B.** Alignment correspond to the B domain for the HIRA proteins. The GRRRIxPLxI (with x is any amino acid) motif is involved in ASF1 binding [9] and corresponding residues are in blue. This motif is highly conserved except the last isoleucine. **C.** Alignment correspond to the HIRA domain for the HIRA proteins. The W799/D800-HsHIRA residues reported for their putative role in homooligomerization [107] are depicted in blue and those for CABIN1 binding (with the N-terminal region of CABIN1 containing TPR repeats, [33]) in orange (F870/R871/L874-HsHIRA). Only alignment of the beginning of the HIRA domain is displayed since the end is less conserved. Alignments are performed with HIRA proteins used in the phylogenetic tree reported in Figure S2I. **D.** Alignment correspond to the NHRD domain for the UBN proteins. Several residues (R46/E53/E61/F62/K74) were identified in the HsUBN1 NHRD domain for their role in the interaction with HsHIRA [109]. These residues are depicted in blue. **E**. Alignment correspond to the HRD domain for the UBN proteins. The HRD domain confers a specificity for H3.3/H4 binding and key residues were identified (Y132/D133, D136, F138/I139/D140/N141) for their role in histone binding [32]. These 7 residues are displayed in green. Alignments are performed with CAF1B proteins used in the phylogenetic tree reported in Figure S2J. DrUBN1 was removed from the NHRD alignment since the sequence available in databases is truncated and does not have such a domain. ScHpc2 was removed from both alignments since both domains are localized in the C-terminal. **F.** Alignment correspond to the MEF2-binding domain retrieved only in animal CABIN1.

Alignments are performed with animal CABIN1 proteins used in the phylogenetic tree reported in Figure S2K. The *Physarum* proteins appear in red. Positions refer to the mature protein with the initial methionine. Asterisk indicates a fully conserved residue labelled with a pale pink rectangle. Colon and dot indicate conservation between residues of, respectively, strongly and weak similar properties and are labelled with a pale blue rectangle. Atr: *Amborella trichopoda;* At: *Arabidopsis thaliana*; Ce: *Caenorhabditis elegans;* Dd: *Dictyostellium discoideum*; Dr: *Danio rerio;* Dm: *Drosophila melanogaster*; Hs: *Homo sapiens*; Mm: *Mus musculus*; Pp: *Physarum* polycephalum; Ppa: *Physcomitrella patens*, Sc: *Saccharomyces cerevisiae;* Tt: *Tetrahymena thermophila*; Zm: *Zea mays*.

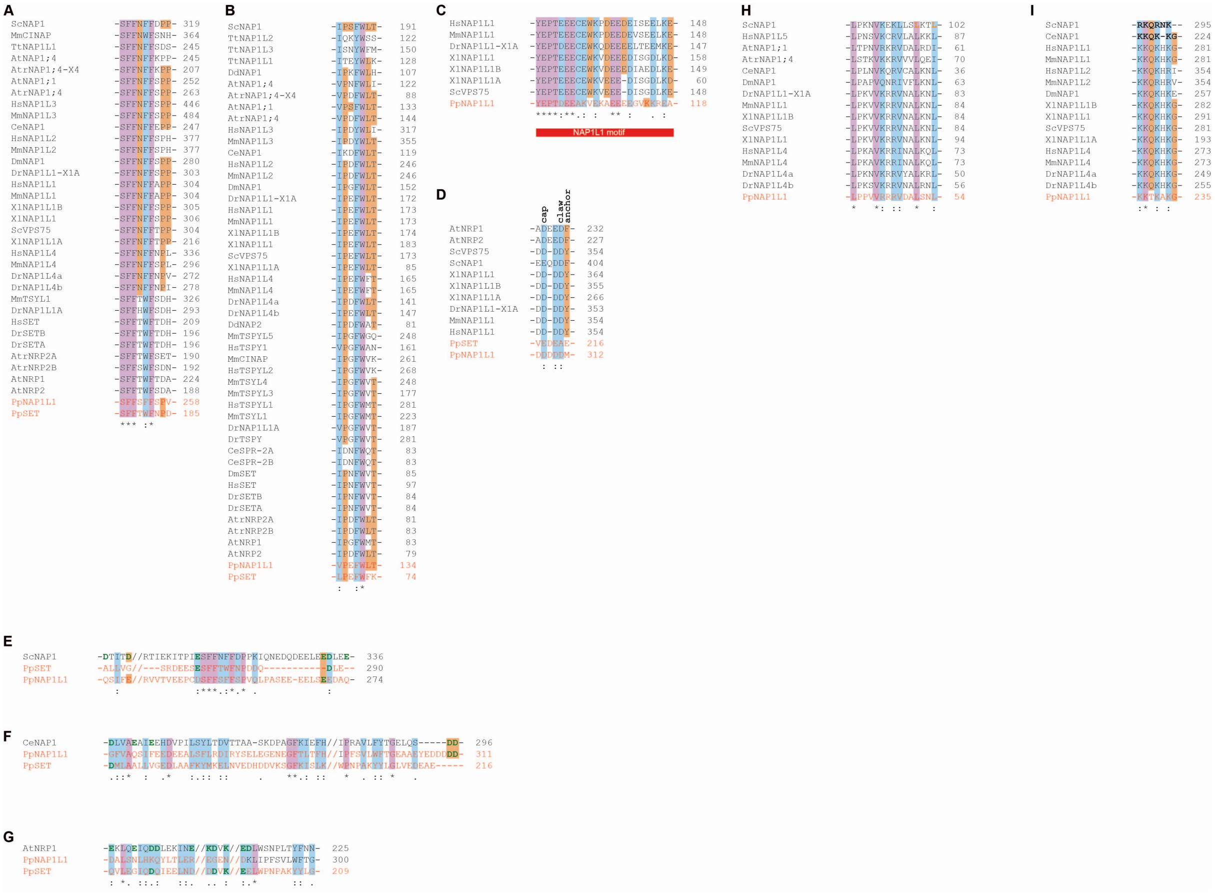

Figure S9. Protein alignments for the *Physarum* proteins of the NAP family. **A-B.** Alignments with the SFFNFFDPP and IPSFWLT sequences of ScNAP1 that participate to the NAP hydrophobic core and protein assembly [13]. Both sequences they are highly conserved among NAP family members, especially in PpSET and PpNAP1L1. **C.** Alignment for the NAP1L histone-binding motif [49]. It was retrieved in PpNAP1L1 but not in SET proteins since this motif is known to be absent from SET proteins. **D.** Alignment for the Cap-Claw-Anchor (cap residue: E/D/S; claw residue: D/E; anchor residue: Y/F; [34]) motif located in the C-terminal acidic tail of some NAP protein. This motif is also important for H2/H2B binding but only the cap and claw residues are conserved in PpNAP1L1 and PpSET. **E** to **G.** Alignments of the PpNAP1L1 and PpSET proteins with the histone binding modules of NAP proteins from yeast (**E**), *C. elegans* (**F**) and *Arabidopsis* (**G**). Mutation of residues D201/ D205/ E310/ E332/ D333/ E336-ScNAP1 (**E**), D125/ E129/ E132/ D295/ D296 of CeNAP1 (**F**) and K115/ D116/ K118/ E213/ D214 of AtNRP1 (**G**) reduce H2A/H2B binding [34,35]. Those residues involved are depicted in green and only few residues are conserved in *Physarum* NAP proteins which suggests PpNAP1L1 and PpSET present distinct surfaces for H2A/H2B binding. **H.** Alignment correspond to the NES signal for NAP proteins that present such signals. NES enables cytoplasmic localization [112]. Mutations of L99/L102-ScNAP1 prevent its cytoplasmic localization [112]: these two residues are depicted in orange and are conserved in PpNAP1L1 and in some other NAP proteins but not in PpSET. Those findings were further confirmed by predictions tools (Tables S5 and S6 for NSL and

NES). **I.** Alignment correspond to the NLS signals for NAP proteins that present such signals. AtNRP1 was removed from the alignment since its NLS signal is too divergent (**I**). In bold, are presented the NLS signals described in [35]. Alignments are performed with NAP proteins used in the phylogenetic tree reported in Figure S2L. The *Physarum* proteins appear in red. Positions refer to the mature protein with the initial methionine. Asterisk indicates a fully conserved residue labelled with a pale pink rectangle. Colon and dot indicate conservation between residues of, respectively, strongly and weak similar properties and are labelled with a pale blue rectangle. Almost conserved residues are labelled by an orange rectangle. Atr: *Amborella trichopoda;* At: *Arabidopsis thaliana*; Ce: *Caenorhabditis elegans;* Dd: *Dictyostellium discoideum*; Dr: *Danio rerio;* Dm: *Drosophila melanogaster*; Hs: *Homo sapiens*; Mm: *Mus musculus*; Pp: *Physarum* polycephalum; Ppa: *Physcomitrella patens*, Sc: *Saccharomyces cerevisiae;* Tt: *Tetrahymena thermophila*; Zm: *Zea mays*.

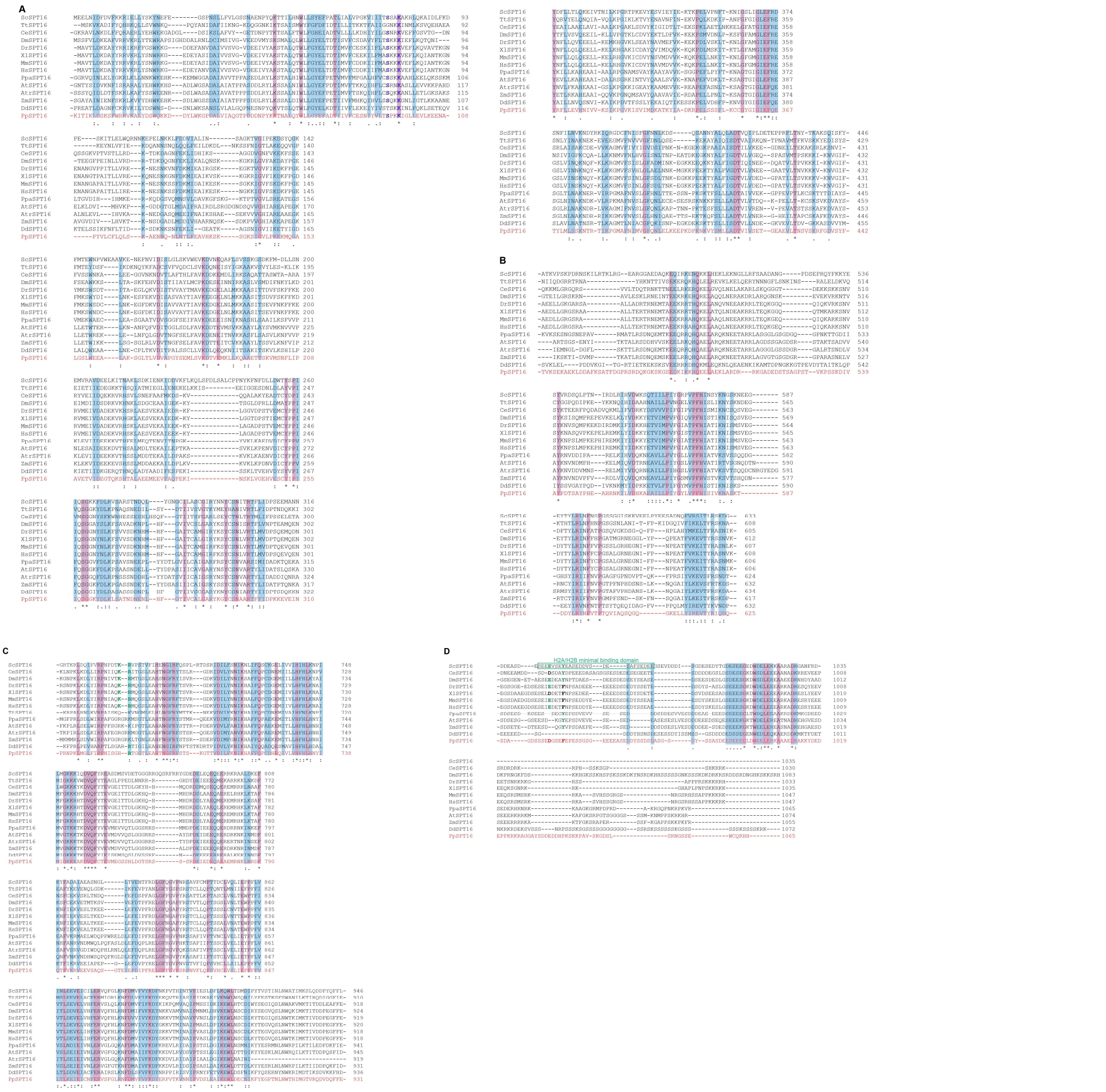

Figure S10. Protein alignments for the *Physarum* SPT16 protein of the FACT complex. **A.** Alignment correspond to the N-terminal region for the SPT16 proteins. Residues S83A/K86A-SpSPT16 are involved in H4 binding [113]. They are conserved across species and are depicted in blue. **B.** Alignment correspond to the dimerization region for the SPT16 proteins. **C.** Alignment correspond to the middle region for the SPT16 proteins. Residues K692/R693-ScSPT16 are important for H3/H4 interaction [36], the former being only conserved in animals while the latter is conserved in animals and slime molds. Both are depicted in green. **D.** Alignment correspond to the C-terminal region for the SPT16 proteins. AtrSPT16 was removed from the alignment since it is a much shorter protein and TtSPT16 was removed for an easier reading. The H2A/H2B minimal binding domain defined in ScSPT16 C-terminal domain [39] is represented by a green square. and key residues for H2A/H2B binding are depicted in bold green or in bold black if substituted. Residues E968/Y972-ScSPT16 are either conserved or substituted by, respectively, another acidic (D) or aromatic (F) residue like in

PpSPT16. They are depicted in green if conserved or in bold black if substituted to an amino acid of similar properties. Alignments are performed with SPT16 proteins used in the phylogenetic tree reported in Figure S2M. The *Physarum* protein appears in red. Positions refer to the mature protein with the initial methionine. Asterisk indicates a fully conserved residue labelled with a pale pink rectangle. Colon and dot indicate conservation between residues of, respectively, strongly and weak similar properties and are labelled with a pale blue rectangle. Atr: *Amborella trichopoda;* At: *Arabidopsis thaliana*; Ce: *Caenorhabditis elegans;* Dd: *Dictyostellium discoideum*; Dr: *Danio rerio;* Dm: *Drosophila melanogaster*; Hs: *Homo sapiens*; Mm: *Mus musculus*; Pp: *Physarum* polycephalum; Ppa: *Physcomitrella patens*, Sc: *Saccharomyces cerevisiae;* Tt: *Tetrahymena thermophila*; Zm: *Zea mays*.

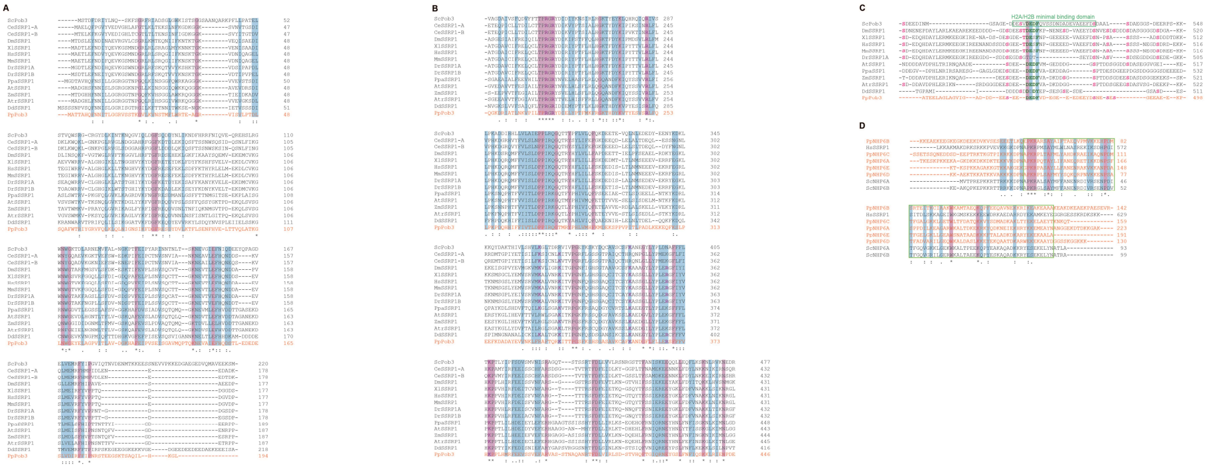

Figure S11. Protein alignments for the *Physarum* Pob3 and NHP6 proteins of the FACT complex. **A.** Alignment correspond to the N-terminal and dimerization regions for the Pob3/SSRP1 proteins. **B.** Alignment correspond to the middle region for the Pob3/SSRP1 proteins. Residues R211/R213/R241/K319/K325/K346/R357/K364-HsSPT16 involved in DNA binding [119] are well conserved and depicted in purple. **C.** Alignment correspond to the IDD region for the Pob3/SSRP1 proteins. The H2A/H2B minimal binding domain defined in ScPob3 IDD region [39] is represented by a green square. It is poorly conserved across species. Key residues of the H2B-binding motif (D/E)xxϑ (where ϑ is F or Y, and x is any residue) [39] are depicted in green and those for nucleosomal DNA binding in pink. The H2B-binding motif is strongly conserved in SPT16 proteins, especially in PpSPT16 and in DdSSRP1 [37]. Serine phosphorylation in DmSSRP1 C-terminal region inhibits nucleosomal DNA binding. These residues are depicted in pink and conserved but to a smaller extent in PpPob3. **D.** Alignment correspond to the HMG region for the Pob3/SSRP1 proteins. The HMG box is depicted by a light green box. The yeast and *Physarum* Pob3 proteins lack the HMG box present in HsSSRP1. The HMG box is located in the NHP6 proteins of yeast and *Physarum*. Alignment is performed with NHP6 proteins reported in Table S2. Alignments are performed with Pob3/SSRP1 proteins used in the phylogenetic tree reported in Figure S2N. TtSSRP1 was removed from the alignment for an easier reading in Figure S11A-C. The alignment in **c** was performed according to [140]. The *Physarum* proteins appear in red. Positions refer to the mature protein with the initial methionine. Asterisk indicates a fully conserved residue labelled with a pale pink rectangle. Colon and dot indicate conservation between residues of, respectively, strongly and weak similar properties and are labelled with a pale blue rectangle. Atr: *Amborella trichopoda;* At: *Arabidopsis thaliana*; Ce: *Caenorhabditis elegans;* Dd: *Dictyostellium discoideum*; Dr: *Danio rerio;* Dm: *Drosophila melanogaster*; Hs: *Homo sapiens*; Mm: *Mus musculus*; Pp: *Physarum* polycephalum; Ppa: *Physcomitrella patens*, Sc: *Saccharomyces cerevisiae;* Tt: *Tetrahymena thermophila*; Zm: *Zea mays*.

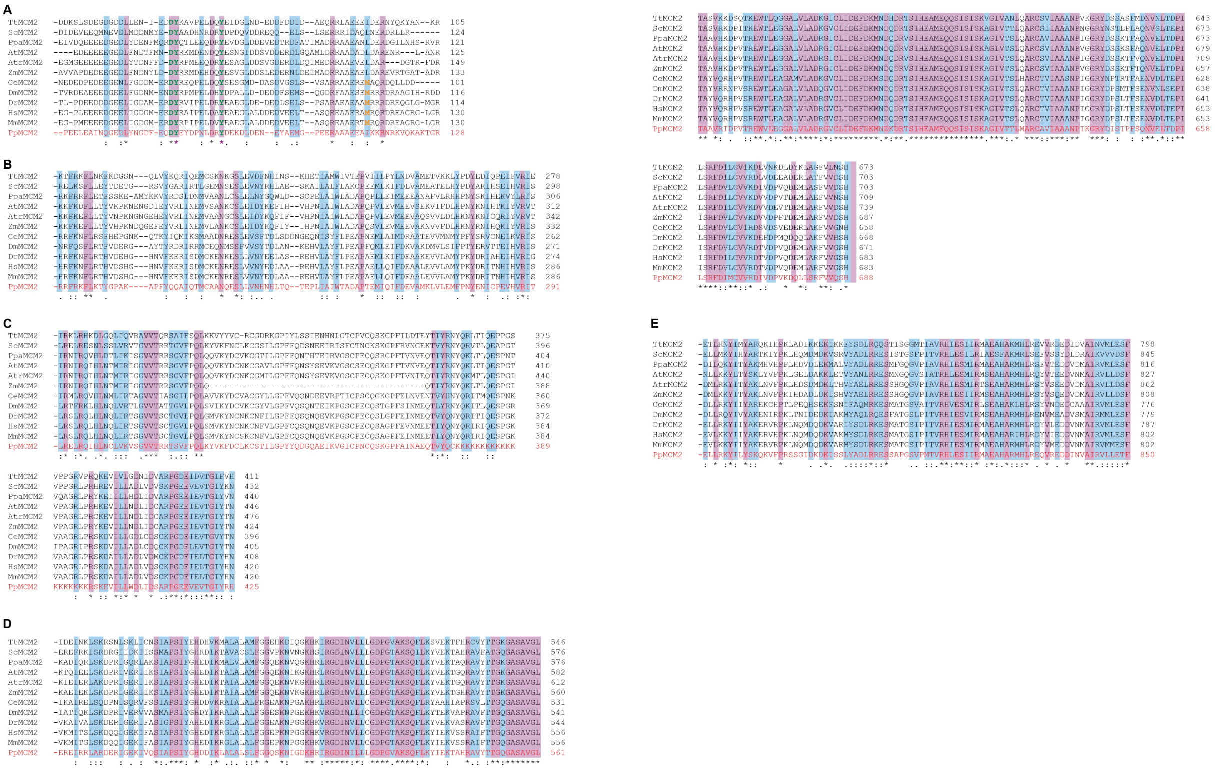

Figure S12. Protein alignments for the *Physarum* MCM2 protein. Alignments correspond to the HBD (**A**), MCM N-terminal (**B**), OB-fold (**C**), MCM (**D**) and AAA-lid (**E**) domains for the MCM2 proteins. The D80/Y81/Y90-HsMCM2 residues are involved in H3/H4 binding: they are conserved in all analyzed species including *Physarum* and are displayed in green (**A**). The Y81/Y90-HsMCM2 residues are also involved in the MCM2 association with the histones-FACT complex [12]. Both are conserved in all analyzed species which is represented by a purple asterisk (**A**). Alignments are performed with MCM2 proteins used in the phylogenetic tree reported in Figure S2O. The *Physarum* protein appears in red. Positions refer to the mature protein with the initial methionine. Asterisk indicates a fully conserved residue labelled with a pale pink rectangle. Colon and dot indicate conservation between residues of, respectively, strongly and weak similar properties and are labelled with a pale blue rectangle. Atr: *Amborella trichopoda;* At: *Arabidopsis thaliana*; Ce: *Caenorhabditis elegans;* Dd: *Dictyostellium discoideum*; Dr: *Danio rerio;* Dm: *Drosophila melanogaster*; Hs: *Homo sapiens*; Mm: *Mus musculus*; Pp: *Physarum* polycephalum; Ppa: *Physcomitrella patens*, Sc: *Saccharomyces cerevisiae;* Tt: *Tetrahymena thermophila*; Zm: *Zea mays*.

A

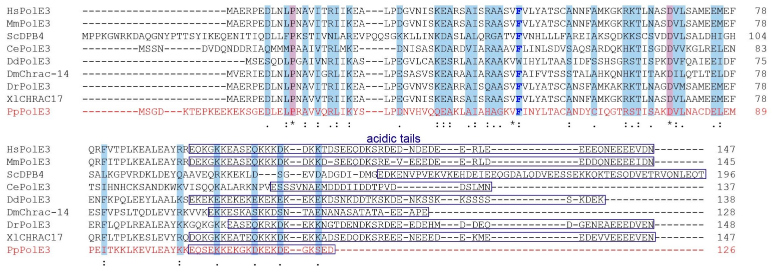

B

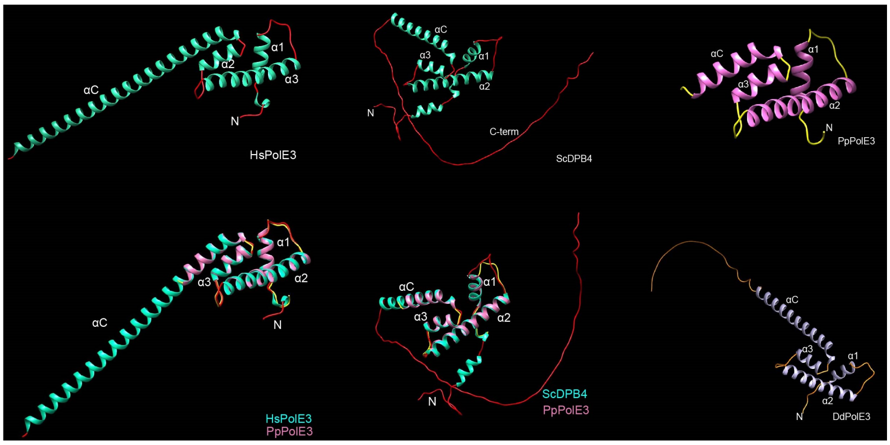

Figure S13. Analysis of the PolE3 chaperone. **A.** Alignment correspond to the PolE3 proteins. The C-terminal region of PolE3 is required for interaction with H3/H4 and the F44 residue involved in the interaction between HsPolE3 and HsPolE4, HsPolE1, HsPolE2 [122] is conserved in PpPolE3 and displayed in blue. Acidic tails are indicated by blue rectangles. Alignment is performed with PolE3 proteins used in the phylogenetic tree reported in Figure S2P. The *Physarum* proteins appear in red. Positions refer to the mature protein with the initial methionine. Asterisk indicates a fully conserved residue labelled with a pale pink rectangle. Colon and dot indicate conservation between residues of, respectively, strongly and weak similar properties and are labelled with a pale blue rectangle. Atr: *Amborella trichopoda;* At: *Arabidopsis thaliana*; Ce: *Caenorhabditis elegans;* Dd: *Dictyostellium discoideum*; Dr: *Danio rerio;* Dm: *Drosophila melanogaster*; Hs: *Homo sapiens*; Mm: *Mus musculus*; Pp: *Physarum* polycephalum; Ppa: *Physcomitrella patens*, Sc: *Saccharomyces cerevisiae;* Tt: *Tetrahymena thermophila*; Zm: *Zea mays*. **B.** Ribbon representation of HsPolE3, ScDPB4, PpPolE3 and DdPolE3 proteins. The αC helix characteristic of H2B family is much shorter in PpPolE3 than in HsPolE3 and that HsPolE3 presents a long αC helix while ScPDB4 presents a long C-terminal tail. Representation of each chaperone is displayed on the upper panel, excepted for DdPolE3 presented in the right lower panel. Superimpositions of HsPolE3 and ScDPB4 with PpPolE3 are presented in the lower panel. The various helices (α1, α2, α3, αC) and the N-terminal tail (N) are indicated. Models were generated with Phyre2 for PpPolE3 and AlphaFold for HsPolE3, ScDPB4 and DdPolE3. Superimpositions were done with the Chimera software.

A

```
PpaSPT6     -YSVGS-YGYSA---------------------------------GSRRPSESGAS-------------   1483
AtrSPT6     -GGSSGDGGWRGGRSSFDRDRSS---TPGSRTGRHGNNARDGHPSGVPRPYG---QG-RGRGGRNDEGN   1529
AtSPT6L     -HGSSGGSGWGSSQSEGGWKGNSDRSGSGRGGEYRNGGGRDGHPSGAPRPYGGRGRG-RGRGRRDDMNS   1502
ZmSPT6      -WGSGG------GGDANGGWRGDGSNDR------DRPFSGRSGGRFDSRNSSGGRGRG-RGRGRGN----   1509
PpSPT6      -MST--------------PQINATPAHPGAATPAHPGNATPAHPGNATPAHQGSGWANAAQGGNNFPGM   1518
               .                                          .

PpaSPT6     GFDSRNSQWGDSR--AGGGGWGSS-----SGWGSR---GNSGWDKAV-----------VR   1522
AtrSPT6     DSGYGSSKWGSGAN-DSGGGWGSGSKDSGGGWGSGSKDTGGGWGSGSKDTGGGWGSGSKE   1588
AtSPT6L     DRQDGNGDWGNNDTGTADGGWGNS--------------GGGGWGSES-------------   1535
ZmSPT6      --------FGDD--DNAGGGWSGG--------------GGGG------------------   1527
PpSPT6      QAM--QQNLPATSSLSLWGGWDQSGTST----PGHSQGGAGGWNNNNTNNYGNNNNAFAN   1572
                              ***. .                .*

PpaSPT6     PSG-----------------------------DN---------RGSTSNWGPEPSPRNNW   1544
AtrSPT6     SGGGAWGSLSGHKSSESPSRDDLQAGGGWSGSAG---------GGRNSDWGGGGGGGGG-   1638
AtSPT6L     ----------AGKK------------------TG---------GGSTGGWGSESGGNKS-   1557
ZmSPT6      ---------------------------------------------NSGRWTDNIGT-GG-   1540
PpSPT6      ----------------P---TNDVTMGWEDNYNNNNNNSNNYGNSNSNYGQNDSYGNNR   1612
                                                          .. :    .   .

PpaSPT6     DKNGGAGRSAWDNSQPGAPSQWDNVT----------------------------------   1570
AtrSPT6     -SGGKGSSSDWVGGGSGRNSEWGSGESGQNSDGVGDAGSGRNNDWGGGGGSGSGRN-NDW   1696
AtSPT6L     -DG--------------------------------------AGSWGSGS-----------   1567
ZmSPT6      -SG--------------------------------------GSSWGGGGGGD--------   1553
PpSPT6      DDR-----SRRDN---------NSNNYGNNRD---NYGNNRGNSYRDNNNYNSNRDSSRY   1655
             .

PpaSPT6     --------------NSEGGAAGDGWAAVPGATPT---------------SGSGWDAVP--   1599
AtrSPT6     GGGGSVAGGKGPNSDWGGGGGGGGGSGGGGKGPNSDWGGGSGGGGKGPNSEWGGGGS-   1755
AtSPT6L     -----------------GGGGSGGWGNDSGGKKSSED---GGFGSGSGGGGSDWGNES--   1605
ZmSPT6      --------------DSNRGGGGGGWGSAGGGAD------------NAGGGNGAWGAAA--   1585
PpSPT6      GGGGYGGGGR-DN-YRSGGGGGGGYGGGSG---------------------GGYGGDRNR   1692
                              *....* .   *                     . :.

PpaSPT6     ----GAT-----P-------------------------TSGSGWMPGGHAGPRGF-----   1620
AtrSPT6     -GSGGGE-----KGPDGDWG--GGGSGGGGKGPDGDWGGGDSGWNSGKKGGSSGAAWPES   1807
AtSPT6L     ---GGKK-----SSADGG------------------WGS------------------ESG   1621
ZmSPT6      ---GGSD-----DPGLGS------------------A--------------------KKV   1599
PpSPT6      DSRGGRDNYNNNRRDDRDYNRRSGGGGGGGYGGDRNRD-------------------SRD   1733
                *

PpaSPT6     ----------------------------------------------------------   1620
AtrSPT6     GGGNSGGGGWNASTTTSATNVGGGGWA-------------------------------   1834
AtSPT6L     GKKSDGEGGWGNEPSSRKSDGGGGGW--------------------------------   1647
ZmSPT6      VPAQDGGTGWGS---------EGGGGW--------------------------------   1617
PpSPT6      NRGRDGGRGWDDRDKGSGGGGGGGGGRRSRGGEGFEISAQQVFHGLLSFPLVPFWKSLL   1791
```

B

```
ScSPT6      -SDKIDEMYDIFGDG-HDYDWALEIEN---EELEN---GNDNN-EAEEEEIDEET--GA---------IKSTKKKISLQDIYDLEDLKKNLMTEG   314
CeSPT6      -EGAEDDARDVFGVEDFNLDE-----------FYDDDDGEDGLED-EEEEIIEDDGEGGEIKIRRKKDTTK---KSTLLESIEPSEIDRGFLLPG   292
DmSPT6      -DASLQEGQDIFGV-DFDYDD-----------FSKYEEDDYEDDS-EGDEYDEDLGVGDDTRVKKKKALKKKVVKKTIFDIYEPSELKRGHFTDM   263
DrSPT6      -DAALQEAQEIFGG-DFDFAE-----------FDTEAYDHAE--------EE-EEDQDDESWDRPKKQTKRRVSRRSIFEIYEPSELESSHMTDQ   280
XLSPT6      -DAALQEAQEIFGV-DFDYDE-----------FEKYDEDDEEM-E-EYD-YE-DDEGDAETRVRPKKTAKKRVSRRSIFEMYEPSELESSHLTDQ   283
HsSPT6      -DAALQEAQEIFGV-DFDYDE-----------FEKYNEYDEELEE-EYE-YE-DDEAEGEIRVRPKKTTKKRVSRRSIFEMYEPSELESSHLTDQ   273
MmSPT6      -DAALQEAQEIFGV-DFDYDE-----------FEKYNEYDEELEE-DYE-YE-DDETEGEIRVRPKKTTKKRVSRRSIFEMYEPSELESSHLTDQ   281
PpaSPT6     -SIALQEAQEIFGDVTSLLEERRRRERDTLAGVDDEDRVDD-------DD---DDGYGR---------SRKPPSQKLLEKQFEPSLLEEKFLTER   364
AtrSPT6     -SSALQEAHEIFGDVDELLRFRKLGHGKAGDGFDGVD----------------DS---------------VDGKPKRLEDEFEPSILEEKYMTEK   286
AtSPT6L     -ITAMRDANEIFGDVDELLTIRKKGLA---------------------------S---------------NQRMERRLEDEFEPTVLSEKYMTGN   286
ZmSPT6      -SSALQEAQDIFGDVDELLALRKQELEREAL----------------------NS---------------GELRGARLEDEFEPFILAEKYMTSK   338
PpSPT6      -RGALQDAEDIFGSVDFFKQASGRRSRPRGDEFDDDGDYVDGTQGLSEDELDAEIEEGD---------EKQKDPLADIYEHYEPSLLEQKHFTSE   275
                  :  ::**                                                                : .  :   :    :
```

Figure S14. Protein alignments for the *Physarum* SPT6 protein. **A.** Alignment correspond to the WG/GW domain for the SPT6 proteins. The WG/GW domain was described as a plant-specific WG/GW domain required for Argonaute interaction and small RNA-mediated gene silencing: this domain is surprisingly also present in PpSPT6 (**f**, WG/GW in purple). **B.** Alignment correspond to the H3-binding region for the SPT6 proteins. The H3-binding region corresponds to the region 239-314AA of ScSPT6 [44] which is located between the acidic N-terminal domain responsible for Iws1 (Interact-With-SPT6) binding and the HtH domain. The F249-ScSPT6 residue involved in nucleosome binding is depicted in green (**B**). Alignments are performed with SPT6 proteins used in the phylogenetic tree

reported in Figure 2B. The *Physarum* proteins appears in red. Positions refer to the mature protein with the initial methionine. Asterisk indicates a fully conserved residue labelled with a pale pink rectangle. Colon and dot indicate conservation between residues of, respectively, strongly and weak similar properties and are labelled with a pale blue rectangle. Atr: *Amborella trichopoda;* At: *Arabidopsis thaliana*; Ce: *Caenorhabditis elegans;* Dd: *Dictyostellium discoideum*; Dr: *Danio rerio;* Dm: *Drosophila melanogaster*; Hs: *Homo sapiens*; Mm: *Mus musculus*; Pp: *Physarum* polycephalum; Ppa: *Physcomitrella patens*, Sc: *Saccharomyces cerevisiae;* Tt: *Tetrahymena thermophila*; Zm: *Zea mays*.

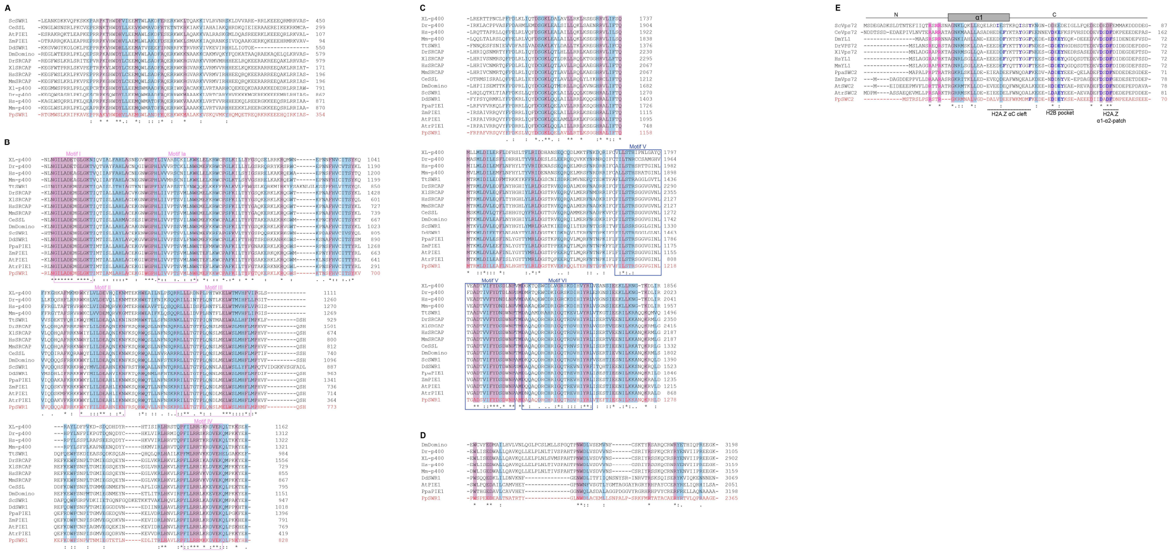

Figure S15. Protein alignments for the *Physarum* SWR-C complex. **A** to **D.** Alignments correspond to the HSA (**A**), SNF2 (**B**), Helicase (**C**) and SANT (**D**) domains for the SWR1 proteins. No HSA domain was found neither in AtrSWR1 nor in TtSWR1 with Interproscan. Five of the seven motifs (I and Ia to IV) that constitute the ATPase domain are located in the SNF2 region (**B**) and two (V and VI) in the Helicase domain (**C**). Alignments are performed with SWR1 proteins used in the phylogenetic tree reported in Figure S2Q. **E**. The helix $\alpha 1$ present in the Z domain is indicated as well as the N-terminal (N) and C-terminal regions. Dashed lines indicate regions which interact with the H2A.Z–H2B dimer (namely H2A.Z αC cleft, H2B pocket and H2A.Z $\alpha 1$–$\alpha 2$-patch). The Z domains (Figure 6C) responsible for H2A.Z binding and selectivity as well as DmYL1 key residues [47] are widely conserved (**E**, in blue). The RxxR motif (x for any residue) anchors ScSWC2 to the nucleosome surface [48] and its arginines are displayed in pink. Alignments are performed with SWC2 proteins used in the phylogenetic tree reported in Figure S2R, TtYL1 and DdYL1 were removed from alignment due to lower conservation. The *Physarum* proteins appear in red. Positions refer to the mature protein with the initial methionine. Asterisk indicates a fully conserved residue labelled with a pale pink rectangle. Colon and dot indicate conservation between residues of, respectively, strongly and weak similar properties and are labelled with a pale blue rectangle. Atr: *Amborella trichopoda;* At: *Arabidopsis thaliana*; Ce: *Caenorhabditis elegans;* Dd: *Dictyostellium discoideum*; Dr: *Danio rerio;* Dm: *Drosophila melanogaster*; Hs: *Homo sapiens*; Mm: *Mus musculus*; Pp: *Physarum* polycephalum; Ppa: *Physcomitrella patens*, Sc: *Saccharomyces cerevisiae;* Tt: *Tetrahymena thermophila*; Zm: *Zea mays*.

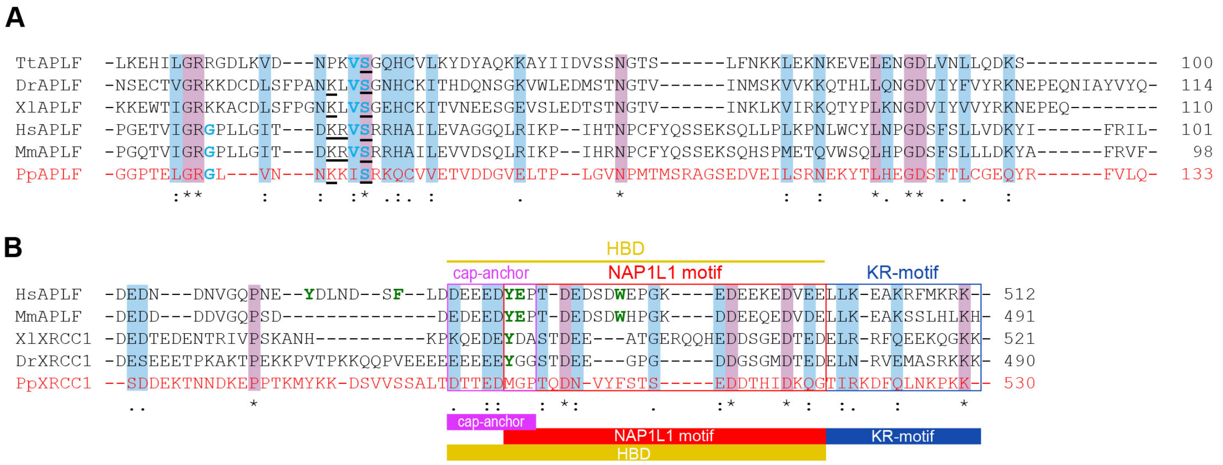

Figure S16. Analysis of the APLF chaperone. **A.** Alignment correspond to the FHA domain for the APLF proteins. In response to DNA damage, APLF proteins can be phosphorylated. The putative phosphate-binding residues are displayed in light blue. Basic residues supposed to confer specificity for binding CK2-phosphorylated motifs are underlined, CK2 being a serine/threonine protein kinase. The HsAPLF S116 which is phosphorylated in response to DNA damage [141] is conserved in PpAPLF and depicted in blue and underlined. **B.** Alignment correspond to the acidic domain for the APLF proteins. The acidic tail of HsAPLF is integrated in XRCC1 for Xenopus, zebrafish and *Physarum*. The acidic domains present the NAP1L motif [49] of the NAP family (red rectangle). They also harbor the H2A-H2B binding cap-anchor motif of HsAnp32E, ScSPT16 and DmYL1 chaperones (purple rectangle) as well as the KR-motif (KR, lysine/arginine, blue rectangle) [50]. The NAP1L motif and the cap-anchor motif constitute the HBD domain of AFLF (orange line). Residues Y462/F468/Y476/E477/W485-HsAPLF are involved in histone binding [49,50,142]. They are depicted in green and are not conserved in most analyzed acidic domains. Alignments are performed with APLF proteins used in the phylogenetic tree reported in Figure S2S. HBD, histone binding domain; KR, lysine/arginine. The *Physarum* proteins appear in red. Positions refer to the mature protein with the initial methionine. Asterisk indicates a fully conserved residue labelled with a pale pink rectangle. Colon and dot indicate conservation between residues of, respectively, strongly and weak similar properties and are labelled with a pale blue rectangle. Atr: *Amborella trichopoda;* At: *Arabidopsis thaliana*; Ce: *Caenorhabditis elegans;* Dd: *Dictyostellium discoideum*; Dr: *Danio rerio;* Dm: *Drosophila melanogaster*; Hs: *Homo sapiens*; Mm: *Mus musculus*; Pp: *Physarum* polycephalum; Ppa: *Physcomitrella patens*, Sc: *Saccharomyces cerevisiae;* Tt: *Tetrahymena thermophila*; Zm: *Zea mays*.

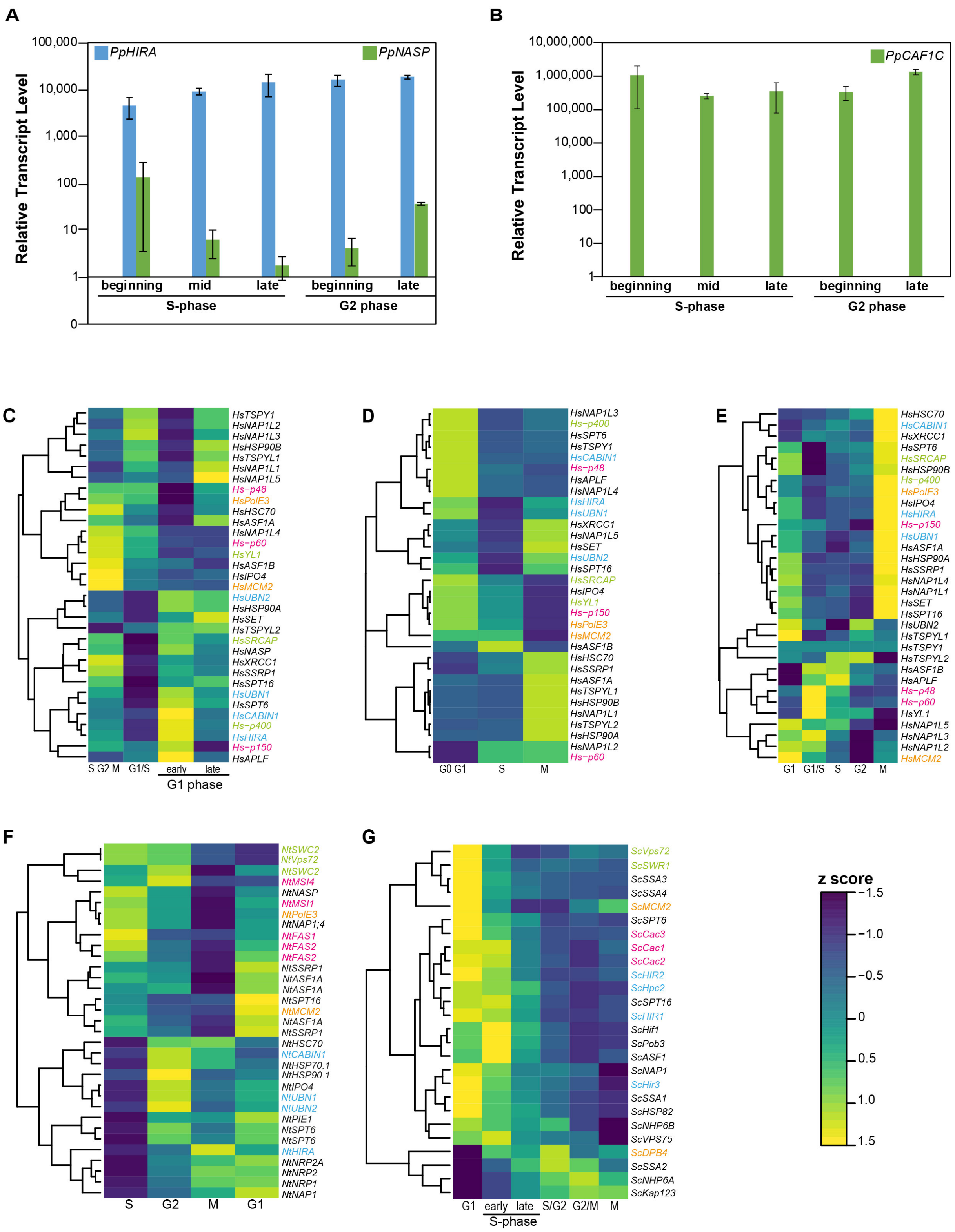

Figure S17. Expression analysis histone chaperone-coding genes. **A-B.** Mean relative transcript level for *Physarum PpHIRA, PpNASP* (**A**) and *PpCAF1C* (**B**) genes measured by qRT-PCR. Means are displayed with a logarithmic scale and obtained from three biological replicates consisting of independent mitotically synchronous plasmodia cultures harvested at different times of the cell cycle. Samples used for RNA-Seq and qRT-PCR are independent. **C-G**. Heat maps displaying the RNA-Seq expression of histone chaperones at various stages of the cell cycle in human (HUVEC, **C**; MCF-7, **D**;

U2OS, **E**), tobacco TN-90 (**F**) and yeast (**G**) cells. Cell cycle stages are indicated at the bottom of the map. Each row corresponds to a transcript listed on the right and each column to a cell cycle time point. The color bar at the bottom right depicts the scale for z-score, with blue representing lowest expression and yellow representing the highest. The CAF-1 complex is associated with H3.1/H4 incorporation during DNA replication and its subunits are displayed in pink. The HIR complex is associated with H3 variant incorporation throughout the cell cycle and its subunits are displayed in blue. The SWR-C complex is involved in H2A.Z/H2B replacement and its subunits are displayed in green. MCM2 and PolE3 are histone chaperones associated with replication and are displayed in orange.

Table S1: List of histone chaperones identified in *Physarum polycephalum.*

Physarum Histone chaperone	Physarum Transcript ID	Scaffold	Number of exons/introns	Predicted Protein Molecular Weight (KDa)	Human homologue(s)	% of Disordered residues (RAPID)
PpASF1	Phypoly_transcript_17984	Scaffold927	4/4	22.56	Anti-silencing function 1A/B (ASF1A/B)	27.23%
PpMCM2	Phypoly_transcript_01999	Scaffold1687	20/19	108.7	Minichromosome maintenance protein 2 (MCM2)	25.29%
PpHSP90	Phypoly_transcript_03991	Scaffold842 & Scaffold258	6/5	80.31	Heat shock protein 90A/B (HSP90A and HSP90B)	21.09%
PpHSC70	Phypoly_transcript_04582	Scaffold831	15/14	91.91	Heat shock cognate 70 (HSC70; HSPA8)	10.99%
PpIPO4	Phypoly_transcript_01375	Scaffold213	7/6	117.65	Importin 4 (IPO4)	11.3%
PpSPT6	Phypoly_transcript_00177	Scaffold740 & Scaffold436	30/32	202.76	Suppressor of Ty 6 (SPT6)	33.22%
PpAPLF	Phypoly_transcript_03499	Scaffold4622 & Scaffold945	2/9	48.09	APLF_aprataxin_and_PNK-like factor	78.67%
PpPOLE3	Phypoly_transcript_27931	Scaffold54	4/3	14.22	PolE3	58.73%
PpSWR1	Phypoly_transcript_00085	Scaffold2256	11/10	261.27	SRCAP, E1A-binding protein p400 (hDomino)	36.0%
PpSWC2	Phypoly_transcript_09236	Scaffold551	7/6	41.56	Protein YL1 (YL1), vacuolar protein sorting 72 homologue (VPS72)	64.67%
PpSPT16	Phypoly_transcript_00559	Scaffold433 & Scaffold1547	23/28	119.68	Suppressor of Ty 16 (SPT16)	25.3%
PpSSRP1	Phypoly_transcript_05191	Scaffold957 & Scaffold3182	15/14	59.88	Structure-specific recognition protein 1 (SSRP1)	28.6%
PpSET	Phypoly_transcript_13763	Scaffold146	5/4	28.52	Patient SE translocation (SET)	45.36%
PpNAP1L1	Phypoly_transcript_11436	Scaffold35	8/7	42.35	Nucleosome assembly protein 1-like (NAP1L1-6)	51.22%
PpCAF1C	Phypoly_transcript_09477	Scaffold1106	ONLY RBAP48	47.76	Retinoblastoma associated protein 46 (RBAP46)	18.44%
PpCAF1A	Phypoly_transcript_03998	Scaffold5423 & Scaffold4498	10/9	83.98	Chromatin assembly factor 1, subunit A (CAF1A or p150)	34.31%
PpCAF1B	Phypoly_transcript_09859	Scaffold6006 & Scaffold8861 & Scaffold10170	14/13	70.28	Chromatin assembly factor 1, subunit B (CAF1B or p60)	61.05%
PpHIRA	Phypoly_transcript_01462	Scaffold428	19/19	110.63	Histone regulation A (HIRA)	25.7%
PpUBN	Phypoly_transcript_03291	Scaffold395	10/9	87.31	Ubinuclein 1 (UBN1) and Ubinuclein 1 (UBN2)	68.04%
PpCABIN1	Phypoly_transcript_00351	Scaffold5	34/35	176.49	Calcineurin-binding protein cabin-1 (CABIN1)	23.1%
PpNASP	S3S101K5_5354_LULU & Contig588_N11D4	Scaffold42	5/4	47.52	Nuclear autoantigenic sperm protein (NASP)	45.72%

<u>Table S2</u>: List of protein sequences used for phylogenetic trees. NA, no homolog identified by a blast search in this organism.

RefSeq ID	Tree ID	Organism	Chaperone	Protein Sequence
NP_176846.1	AtASF1A	A thaliana	ASF1	MSAIKITNVAVLHNP<br>APFVSPFQFEISYECL<br>NSLKDDLEWKLIYVG<br>SAEDETYDQLLESVLV<br>GPVNVGNYRFVFQA<br>DPPDPSKIQEEDIIGV<br>TVLLLTCSYMGQEFLR<br>VGYYVNNDYEDEQLK<br>EEPPTKVLIDKVQRNI<br>LSDKPRVTKFPIDFHP<br>EEEQTAATAAPPEQS<br>DEQQPNVNGEAQVL<br>PDQSVEPKPEES
NP_198627.1	AtASF1B	A thaliana	ASF1	MSSINITNVTVLDNP<br>APFVNPFQFEISYECL<br>TSLKDDLEWKLIYVGS<br>AEDETYDQVLESVLV<br>GPVNVGNYRFVLQA<br>DSPDPLKIREEDIIGVT<br>VLLLTCSYMDQEFIRV<br>GYYVNNDYDDEQLRE<br>EPPTKVLIDKVQRNIL<br>TDKPRVTKFPINFHPE<br>NEQTLGDGPAPTEPF<br>ADSVVNGEAPVFLEQ<br>PQKLQEIEQFDDSDV<br>NGEAIALLDQPQNLQ<br>ET
XP_006843956.1	AtrASF1A	A. trichopoda	ASF1	MSAVNITNVTVLDNP<br>AAFLNPFQFEISYECLL<br>PLKDDLEWKLIYVGS<br>AEDETYDQLLESVLVG<br>PINVGNYRFVFQADP<br>PDPTKIPDEDIIGVTVL<br>LLTCSYMGQEFIRVGY<br>YVNNDYDDEQLREEP<br>PPRVLIDRVQRNILAD<br>KPRVTKFPINFHPESA<br>DSTDAPPPSSPPSSEN<br>PIEEPQTEPSARLDT
NP_492567.3	CeASF1L	C. elegans	ASF1	MASRVNIVQVQILDN<br>PAMFVDKFKMEITFE<br>VFEHLPHDLEWELVY<br>VGSGTSRDFDQVLDS<br>ALVGPIPEGRHKFVF<br>DAEHPDISKIPVEDIV<br>GVSVLLLRCKYNDQE<br>FINMGWFVANEYTD<br>EELKENPPAKPLIEKLS<br>RKIETEDLRVTTFPIR<br>WTDEDPVAEPVDEE<br>ANKVFDEDDLMPLH<br>DDGQDDDEEEEDDD<br>ETGPNTEEVDLNESF<br>NERMANAHDGTEQ<br>KNGEESMEHDGASG<br>DVEMGDKH
XP_638098.1	DdASF1	D. discoideu	ASF1	MSHIRLTQVLIHNNP<br>SSFNSPFIFDISFECVS<br>PIKEDLEWKVIYVGSA<br>DNEKNDQVLDSILLG<br>PVAVGQNQFVFEVD<br>PPDANKIPKDDLLGV<br>TVVFLICAYKGEDFIR<br>VGYYVNNDYFEQELK<br>DNPPETPDLSKIQRN<br>VMDDKPVVTGFPIQ<br>WN
NP_001189131.1	DmASF1	D. melanogas	ASF1	MAKVHITNVVVLDN<br>PSSFFNPFQFELTFECI<br>EELKEDLEWKMIYVG<br>SAESEEHDQVLDTIYV<br>GPVPEGRHIFVFQAD<br>PPDVSKIPEPDAVGV<br>TIVLLTCSYRGQEFVR<br>VGYYVNNDYADPEM<br>RENPPTKPLFEKLTRN<br>ILASKPRVTRFKINWD<br>YGHINGNGNGVENG<br>HQDEMATDGPSTSE<br>AASAVIHPEDDNSLA<br>MPMENGIKALNENS<br>NSLAMEC

NP_996946.1	DrASF1A	D. rerio	ASF1	MAKVQVLNVAVLDNPSPFGNPFQFEITFECMEDLPEDLEWKIIYVGSAESEEYDQTLDSVLVGPVPAGRHMFVFQADAPNCSLIPETDAVGVTVVLITCTYRGQEFIRIGYYVNNEYTDTELRENPPLKPNYGQLQRNILASNPRVTRFHINWEGCAEKMEDSENVDPAPNAMLPPSCTPGKAPLLGLVPDNSMDCL
NP_999922.1	DrASF1B	D. rerio	ASF1	MAKVQVLNVAVLDNPSPFGNPFQFEITFECMEDLPEDLEWKIIYVGSAESEEYDQILDSVLVGPVPAGRHMFVFQADAPNTGLIPESDAVGVTVVLITCTYRGQEFIRIGYYVNNEYTDPELRENPPVKPDYTQLQRNILASNPRVTRFHINWEATMDKMEDSENVDPAPNAMLPPTCMPGKAPPLGLMPDNSMDCL
NP_054753.1	HsASF1A	H. sapiens	ASF1	MAKVQVNNVVVLDNPSPFYNPFQFEITFECIEDLSEDLEWKIIYVGSAESEEYDQVLDSVLVGPVPAGRHMFVFQADAPNPGLIPDADAVGVTVVLITCTYRGQEFIRVGYYVNNEYTETELRENPPVKPDFSKLQRNILASNPRVTRFHINWEDNTEKLEDAESSNPNLQSLLSTDALPSASKGWSTSENSLNVMLESHMDCM
NP_060624.1	HsASF1B	H. sapiens	ASF1	MAKVSVLNVAVLENPSPFHSPFRFEISFECSEALADDLEWKIIYVGSAESEEFDQILDSVLVGPVPAGRHMFVFQADAPNPSLIPETDAVGVTVVLITCTYHGQEFIRVGYYVNNEYLNPELRENPPMKPDFSQLQRNILASNPRVTRFHINWDNNMDRLEAIETQDPSLGCGLPLNCTPIKGLGLPGCIPGLLPENSMDCI
NP_079817.1	MmASF1A	M. musculus	ASF1	MAKVQVNNVVVLDNPSPFYNPFQFEITFECIEDLSEDLEWKIIYVGSAESEEYDQVLDSVLVGPVPAGRHMFVFQADAPNAGLIPDADAVGVTVVLITCTYRGQEFIRVGYYVNNEYTETELRENPPVKPDFSKLQRNILASNPRVTRFHINWEDNTEKLEDAESSNPNLQSLLSTDALPSASKGWSTSENSLNVMLESHMDCM
NP_077146.1	MmASF1B	M. musculus	ASF1	MAKVSVLNVAVLENPSPFHSPFRFEISFECSEALSDDLEWKIIYVGSAESEEFDQILDSVLVGPVPAGRHMFVFQADAPNPSLIPETDAVGVTVVLITCTYHGQEFIRVGYYVNNEYPDPELRENPPPKPDFSQLQRNILASNPRVTRFHINWDNNPDSLEAIENQDPNVDFSLSLSCTPVKSLGLPSCIPGLLPENSMDCI
XP_001768290.1	PpaASF1A	P. patens	ASF1	MTAVNVTNVTVLDNPSMFMNPFQFEISYECLCPLQDDLEWKLIYVGSAEDEKYDQVLESVLVGPVNIGNYRFVFQADPPELSKIPEEDTIGVTVLLLTCSYQGQEFIRVGYYVSNDYVEEALREEPPARVLIEKVQRNILADKPRVTKFPIVFNSPAPVLNHIESERITDSYISEPAAISTLHTYENVSDVHSTPPAAVDIMQTDENIVHAHASLDTPMDLAPADEVRSVDLRTPQTLQEVC

-	PpASF1	P. polycephal	ASF1	MGCVNVTNIQILDNP<br>APFMNPLQFEITYECI<br>TDLQEDLEWKVVYV<br>GSAESEAHDQVLDSIL<br>LGPVRVGVSNFVLQV<br>DPPDSSRIPKQDLLGV<br>TAILLMGLYKSRDFIR<br>VGYFVNNEYAEEELRL<br>NPPEGEVDLTKVVRN<br>ILAEKPRVTRFPIPWD<br>QETIIPPANTENSSSLP<br>AITTETSLDAASNHS<br>MTEEMMPMEVMST<br>ST
NP_012420.1	ScASF1	S. cerevisiae	ASF1	MSIVSLLGIKVLNNPA<br>KFTDPYEFEITFECLES<br>LKHDLEWKLTYVGSS<br>RSLDHDQELDSILVGP<br>VPVGVNKFVFSADPP<br>SAELIPASELVSVTVIL<br>LSCSYDGREFVRVGYY<br>VNNEYDEEELRENPP<br>AKVQVDHIVRNILAE<br>KPRVTRFNIVWDNE<br>NEGDLYPPEQPGVD<br>DEEEEDDEEEDDDED<br>DEDDEDDDQEDGEG<br>EAEEAAEEEEEEEEKT<br>EDNETNLEEEEEDIEN<br>SDGDEEEGEEEVGSV<br>DKNEDGNDKKRRKIE<br>GGSTDIESTPKDAARS<br>TN
XP_001033143.3	TtASF1	T. thermophi	ASF1	MALVNIQNIQFHNN<br>PCPFLSPFKLDVTFECI<br>KPIPDDIEWQLIYIGS<br>AKDEKYDQVLDKFSI<br>GSLDQGVLQFTIETN<br>PPDHTKIPNKDDLLG<br>VTAIILTVSYHNQEFF<br>RVGYYVYNQYNDQE<br>LIINDPPQILIDKVERSI<br>LDKQPRITHFNIKWG<br>TEDENKETDPNTLAIL<br>QQQLAQNGTIPNQL<br>MQEMQQVNSQMQ<br>SFLPDPFSKSSGILQEL<br>TTQGQTNSTNSFMF<br>GQGLDIPQNNAFQP<br>TNVFSSNPY
NP_001086449.1	XlASF1A-A	X. laevis	ASF1	MAKVQVNNVVVLD<br>NPSPFYNPFQFEITFE<br>CIEDLSEDLEWKIIYVG<br>SAESEEYDQVLDSVLV<br>GPVPAGRHMFVFQA<br>DAPNPGLIPDADAVG<br>VTVVLITCTYRGQEFI<br>RVGYYVNNEYTETEL<br>RENPPVKPDFSKLQR<br>NILASNPRVTRFHIN<br>WEENTEKLDDSNPH<br>MHPVLSIEARPSASK<br>GWPMSENSLNVMLE<br>SHMDCM
NP_001080310.1	XlASF1A-B	X. laevis	ASF1	MAKVQILNMVVLDN<br>PCPFHNPFQFEITFEC<br>IEDLPDDLEWKIIYVG<br>SAESEEYDQTLDSVLV<br>GPVPAGRHMFVFQA<br>DAPNCSLIPESDAVG<br>VTVVLITCTYRGQEFI<br>RVGYYVNNEYSDPEL<br>RENPPLKAHFGQLQR<br>NILASNPRVTRFHIN<br>WECTSEAKMEDIENV<br>DPASNTMLPPNCAP<br>SKGLAAALNTLPENS<br>MDCM
NP_001150107.2	ZmASF1A	Z. mays	ASF1	MSAVNITNVAVLDN<br>PTAFLNPFQFEISYECL<br>VPLDDDLEWKLIYVG<br>SAEDENYDQQLESVL<br>VGPVNVGTYRFVLQA<br>DPPDPSKIREEDIIGVT<br>VLLLTCSYMGQEFMR<br>VGYYVNNDYDDEQL<br>REEPPAKVLIDRVQR<br>NILADKPRVTKFPINF<br>HPEPSTGPGQQQQE<br>PQTTSPENHTGNGEA<br>NGSKPEADQ

XP_008656015.1	ZmASF1B	Z. mays	ASF1	MSAVNITNVAVLDN<br>PTAFINPFQFEISYECL<br>VPLDDDLEWKLIYVG<br>SAEDENYDQQLESVL<br>VGPVNVGTYRFVLQA<br>DPPDPSKIREEDIIGVT<br>VLLLTCSYMGQEFMR<br>VGYYVNNDYGDEQL<br>REEPPAKVLIDRVQR<br>NILADKPRVTKFPINF<br>HPEPSTGTGRQQQQ<br>QEPQTASPEKHAGSG<br>EGNGSKPEAD
NP_175112.2	AtMCM2	A thaliana	MCM2	SPSSAGFNTDQLPIST<br>SQNSENFSDEEEAAV<br>DTQVIRDEPDEAEDE<br>EEEEGEDLFNDTFMN<br>DYRKMDENDQYESN<br>GIDDSVDDERDLGQA<br>MLDRRAADADLDAR<br>ENRLANRKLPHLLHD<br>NDSDDWNYRPSKRS<br>RTTVPPRGNGGDPD<br>GNPPSSPGVSQPDIS<br>MTDQTDDYQDEDD<br>NDDEAEFEMYRIQGT<br>LREWVMRDEVRRFIA<br>KKFKDFLLTYVKPKNE<br>NGDIEYVRLINEMVS<br>ANKCSLEIDYKEFIHV<br>HPNIAIWLADAPQPV<br>LEVMEEVSEKVIFDLH<br>PNYKNIHTKIYVRVTN<br>LPVNDQIRNIRQIHLN<br>TMIRIGGVVTRRSGV<br>FPQLQQVKYDCNKC<br>GAVLGPFFQNSYSEV<br>KVGSCSECQSKGPFT<br>VNVEQTIYRNYQKLTI<br>QESPGTVPAGRLPRH<br>KEVILLNDLIDCARPG
XP_020523093.1	AtrMCM2	A. trichopoda	MCM2	GARSPNPSGNGNPR<br>SMDSDDPSTPASPTS<br>AGFSTDRLPPNTSRTS<br>QSLSDEAAVDPAIIRD<br>EEEEGMGEEEEEEGE<br>DLYTDNFFDDYRPME<br>EQDRYESAGLDDSVG<br>DERDLDQIMEDRRA<br>AEVELDARDGTRFDR<br>KLPRLLHDQDTDDDI<br>DYRPKRSRVDYRRPR<br>GGMSYEDVDGATPIS<br>PGGGGSHGGYHGGE<br>PPTTDQTEDEDEDDD<br>AEYGIYRVQGTLREW<br>VTRDEVRRVIAKKFKE<br>FLLTYVNPKNGNGEH<br>EYVRLINEMVAANGC<br>SLEIDYKQFIFVHPNIA<br>IWLADAPQPVLEVM<br>EEVAQSVVLDLHKNY<br>KNICQRIYVRVTNLPI<br>YDQIRNIRQIHLNTMI<br>RIGGVVTRRSGVFPQ<br>LQQVKYDCNKCGMIL<br>GPFFQNSYSEVKVGS<br>CPECQSKGPFTVNIE<br>QTIYRNYQKMTLQES
NP_001022416.1	CeMCM2	C. elegans	MCM2	QPLPIADDADDDVD<br>GIDEMFNNEDEDPE<br>DEEGENLFGDDMER<br>DYREQPELDQYSESG<br>MDDASDVGSLSVSA<br>RRAAEREMAQRDQL<br>LDDDALMYEDGDSEE<br>VDTRRRGRGRRGRG<br>DAADDDSVPMEEEDI<br>PVDILENIRGRTIRDH<br>VSDEAVAKEIERRFKN<br>FLRSFHEPGNKQTKYI<br>QMIKSMAADNRESL<br>EVSFTDLSDDNGEQN<br>ISYFLPEAPNEMLAIM<br>DRAATEVVMNMYPF<br>YSRVCNEIKVRISQLP<br>VEEDIRMLRQVHLN<br>MLIRTAGVVTIASGIL<br>PQLAVVKYDCVACGY<br>LLGPFVQQNDEEVRP<br>TICPSCQGKGPFELNV<br>ENTVYHNYQRITMQ<br>ESPNKVAAGRLPRSK<br>DVILLGDLCDSCKPGD<br>EIEVTGVYTNNFDGSL<br>NYKQGFPVFNTLIHA<br>NHITNKDKMASDQL

XP_637579.1	DdMCM2	D. discoideur	MCM2	NNNNNNNNNKRKT<br>KTPSSVNHNNTKINE<br>DKDLYNFIDKIRDKTD<br>KSHLEDEYDEDDNTK<br>DGKFNKVEDEIKNDI<br>DSEEDDDDIIEDNEHL<br>YDGDEDSENLSSEGE<br>GDDLLDEDLQDQDEE<br>EVLGDEDNHYEMEG<br>LDNETYEDIDKDKKTE<br>VDSILTKRDREEQKTR<br>QATNRHQYRISSFLLD<br>ENDENMVDEDTENN<br>KLLEKRKANLIKKRRE<br>TIRQQNDNIKKDYDG<br>SVPSFKDGFVNSERYF<br>EDGDGDDDDDDDDF<br>DIGDNGQVVNLEEPD<br>KDKCLREYVCMSGPR<br>NEISKQFAKFLLEFKEK<br>DGFNPIYQESIQKMC<br>AANKESLMINFTHLC<br>ASTVFGVWVAEIPTE<br>MIEIFDEVALKVVLRIY<br>PNYRNIVKSIHVRITH<br>LPICESLRDIRQSNLN<br>KLTKVGGVITRRSNVY<br>PQLKHVKYDCVKCKT
NP_477121.1	DmMCM2	D. melanogas	MCM2	AAERRDLRAAMTSPV<br>GDFEPFENEDEILGD<br>QTVRDEAEEEDGEEL<br>FGDNMENDYRPMPE<br>LDHYDPALLDDEDDF<br>SEMSQGDRFAAESE<br>MRRRDRAAGIHRDD<br>RDLGFGQSDDEDDV<br>GPRAKRRAGEKAAV<br>GEVEDTEMVESIENLE<br>DTKGHSTKEWVSML<br>GPRTEIANRFQSFLRT<br>FVDERGAYTYRDRIRR<br>MCEQNMSSFVVSYT<br>DLANKEHVLAYFLPE<br>APFQMLEIFDKVAKD<br>MVLSIFPTYERVTTEI<br>HVRISELPLIEELRTFR<br>KLHLNQLVRTLGVVT<br>ATTGVLPQLSVIKYDC<br>VKCGYVLGPFVQSQN<br>TEIKPGSCPECQSTGP<br>FSINMEQTLYRNYQKI<br>TLQESPGRIPAGRIPR<br>SKDVILLADLCDQCKP<br>GDELEVTGIYTNNYD<br>GSLNTDQGFPVFATV<br>IIANHVVVKDSKQVV
NP_775364.2	DrMCM2	D. rerio	MCM2	TRGSRRGDLTSSPGR<br>DLPPFEDESEGLLGDT<br>LPDEEDDDGEELIGD<br>AMERDYRVIPELDRY<br>EAEGLDEDEDLSELSP<br>SARAEAEAAMRRRD<br>REQGLGMGRIGRGLL<br>YDSEDEDDKRPTKRQ<br>RVLAERAAEGGAME<br>GEDEEMIESIENLED<br>MKGHTVREWVSMA<br>APRLEIYHRFKNFLRT<br>HVDEHGHNVFKERIS<br>DMCKENKESLLVNYE<br>ELASREHVLAYFLPEA<br>PAEMLKIFDEAAKEV<br>VLAMYPKYDRIAHEI<br>HVRIGNLPLVEELRSL<br>RQLHLNQLIRTSGVV<br>TSCTGVLPQLGMVKY<br>NCNKCNFILGPFFQS<br>QNQEVKPGSCPECQ<br>SLGPFEINMEQTVYQ<br>NYQRITIQESPGKVA<br>AGRLPRSKDAILLADL<br>VDMCKPGDEIELTGIY<br>HNNYDGSLNMANGF<br>PVFATVILANHIARKD

NP_004517.2	HsMCM2	H. sapiens	MCM2	QRRRGNDPLTSSPGR<br>SSRRTDALTSSPGRDL<br>PPFEDESEGLLGTEGP<br>LEEEEDGEELIGDGM<br>ERDYRAIPELDAYEAE<br>GLALDDEDVEELTAS<br>QREAAERAMRQRDR<br>EAGRGLGRMRRGLLY<br>DSDEEDEERPARKRR<br>QVERATEDGEEDEE<br>MIESIENLEDLKGHSV<br>REWVSMAGPRLEIH<br>HRFKNFLRTHVDSHG<br>HNVFKERISDMCKEN<br>RESLVVNYEDLAARE<br>HVLAYFLPEAPAELLQ<br>IFDEAALEVVLAMYP<br>KYDRITNHIHVRISHL<br>PLVEELRSLRQLHLNQ<br>LIRTSGVVTSCTGVLP<br>QLSMVKYNCNKCNF<br>VLGPFCQSQNQEVKP<br>GSCPECQSAGPFEVN<br>MEETIYQNYQRIRIQE<br>SPGKVAAGRLPRSKD<br>AILLADLVDSCKPGDE<br>IELTGIYHNNYDGSLN<br>TANGFPVFATVILAN
NP_032590.2	MmMCM2	M. musculus	MCM2	QRRRISDPLTSSPGRS<br>SRRADALTSSPGRDL<br>PPFEDESEGLLGTEGP<br>MEEEEDGEELIGDGM<br>ERDYRPIPELDVYEAE<br>GLALDDEDVEELTAS<br>QREAAERTMRQRDR<br>EAGRGLGRMRRGLLY<br>DSSEEDEERPARKRR<br>HVERATEDGEEDEE<br>MIESIENLEDLKGHSV<br>REWVSMAGPRLEIH<br>HRFKNFLRTHVDSHG<br>HNVFKERISDMCKEN<br>RESLVVNYEDLAARE<br>HVLAYFLPEAPAELLQ<br>IFDEAALEVVLAMYP<br>KYDRITNHIHVRISHL<br>PLVEELRSLRQLHLNQ<br>LIRTSGVVTSCTGVLP<br>QLSMVKYNCSKCNFV<br>LGPFCQSQNQEVKP<br>GSCPECQSAGPFEIN<br>MEETIYQNYQRIRIQE<br>SPGKVAAGRLPRSKD<br>AILLADLVDSCKPGDE<br>IELTGIYHNNYDGSLN<br>TANGFPVFATIILANH
XP_024380390.1	PpaMCM2	P. patens	MCM2	GDFDTDRLPLSSTGG<br>GRRGSMGGSEFDSE<br>NEAVEDRNLFIDEEIV<br>DQEEEEDGEDLYDEN<br>FMQRDYQTLEEQDR<br>YEVDGLDDEVEDTRD<br>FATIMADRRAAEANL<br>DERDGILNHSRVRKL<br>PTMLQEYDDDENEEY<br>QPARRRMRLHEPVA<br>SPVMSMSEHDETGD<br>NEEEEDDEEDDGGTD<br>FLNVQGNSIAEWVA<br>RDDVRRFVQRKFRRF<br>LETFSSKEAMYKKVYR<br>DSLDNMVAANLCSLE<br>LNYGQWLDSCPELAI<br>WLADAPQPLLEIMEE<br>EANAFVLRHHPNYSK<br>IHEKVYLRISNLPLEDK<br>IRNIRQVHLDTLIKISG<br>VVTRRSGVFPQLQQV<br>KYDCVKCGTILGPFFQ<br>NTHTEIRVGSCPECQ<br>SRGPFTVNVEQTIYR<br>NYQKLTLQESPNTVQ<br>AGRLPRYKEIILLHDLI<br>DVARPGEEIEVTGIYV

-	PpMCM2	P. polycephal	MCM2	EEEYDPSIPKDKEAEE<br>DVESETLSDGNQEA<br>MDDILDGEENVVYDP<br>EELEAINQGEDLYNG<br>DFEQDYEYDPNLDRY<br>DEKDLDENEYAEMG<br>PEERAAAEEAIKKRN<br>RKVQKAKTGRLPSAL<br>LYDTDEEEDAKPEYKP<br>RRKRPRVNDPNVGN<br>VFVEEEDTRDLGDLD<br>VNLEDVKGPLREWIS<br>LDPTKRTIMRRFRKFL<br>KTYGPAKAPFYQQAI<br>QTMCAANQESLLVN<br>HNHLTQTEPLIAIWT<br>ADAPTEMIQIFDEVA<br>MKLVLEMFPNYENIC<br>PEVHVRITNLPVSDSL<br>RELRQIHLNCLVKVSG<br>VVTRRTSVFPQLKYVK<br>FDCLKCSTILGPYYQD<br>GQAEIKVGICPNCQS<br>KGPFAINAEQTVYQC<br>KKKKKKKKKKKKKKK<br>KKKRSKEVILLWDLID<br>SARPGEEVEVTGIYRH<br>NFDEHLNKTHGFPVF
NP_009530.1	ScMCM2	S. cerevisiae	MCM2	DSENELPPSSPQQHF<br>RGGMNPVSSPIGSPD<br>MINPEGDDNEVDDV<br>PDIDEVEEQMNEVDL<br>MDDNMYEDYAADH<br>NRDRYDPDQVDDRE<br>QQELSLSERRRIDAQL<br>NERDRLLRNVAYIDD<br>EDEEQEGAAQLDEM<br>GLPVQRRRRRRQYED<br>LENSDDDLLSDMDID<br>PLREELTLESLSNVKA<br>NSYSEWITQPNVSRTI<br>ARELKSFLLEYTDETG<br>RSVYGARIRTLGEMN<br>SESLEVNYRHLAESKA<br>ILALFLAKCPEEMLKIF<br>DLVAMEATELHYPDY<br>ARIHSEIHVRISDFPTI<br>YSLRELRESNLSSLVRV<br>TGVVTRRTGVFPQLK<br>YVKFNCLKCGSILGPF<br>FQDSNEEIRISFCTNC<br>KSKGPFRVNGEKTVY<br>RNYQRVTLQEAPGTV<br>PPGRLPRHREVILLAD<br>LVDVSKPGEEVEVTGI<br>YKNNYDGNLNAKNG
XP_001009217.2	TtMCM2	T. thermophi	MCM2	ADNDSASVSQEEAIE<br>ENQIVEEDDKSLSDE<br>GDGDDLLENIEDDYK<br>AVPELDQYEIDGLND<br>EDDFDDIDAEQRRLA<br>EEEIDERNYQKYANK<br>RRIPTALREFDLGEED<br>EEDLNVQLQRRRQFY<br>EAHNLYESEDDEQME<br>EEEENKDEREYDKYLD<br>REQARGKLAVWIKEE<br>STVRYIRKTFRKFLNKF<br>KDGSNQLVYKQRIQE<br>MCSKNKGSLEVDFN<br>HINSKHETIAMWIVT<br>EPVIILPYLNDVAMET<br>VKKLYPDYEDIQPEIF<br>VRIENYTILDHIRKLRH<br>KDLGQLIQVRAVVTQ<br>RSAIFSQLKKVYYVCR<br>CGDRKGPIYLSSIENH<br>NLGTCPVCQSKGPFIL<br>DTEYTIYRNYQRLTIQ<br>EPPGSVPPGRVPRQK<br>EVIVLGDNIDVARPG<br>DEIDVTGIFVHRYDYA<br>LNVKHGFPVFSTMIE<br>ANYIKRVREGDQSSL

NP_001080759.1	XlMCM2	X. laevis	MCM2	AGSRRDALTSSPGRD<br>LPPFEDESEGMFGDG<br>VVPEEEEDGEELIGDA<br>MERDYRPISELDRYEV<br>EGLDDEEDVEDLTAS<br>QREAAEQSMRMRD<br>REMGRELGRMRRGL<br>LYDSDEEEEDRPARKR<br>RMAERAAEGAPEED<br>EEMIESIENLEDMKG<br>HTVREWVSMAATRL<br>EIYHRFKNFLRTHVDE<br>HGHNVFKEKISDMCK<br>ENKESLPVNYEDLAA<br>REHVLAYFLPEAPAE<br>MLKIFDEAAKEVVLV<br>MYPKYDRIAREIHVRI<br>SHLPLVEELRSLRQLH<br>LNQLIRTSGVVTCCTG<br>VLPQLSMVKYNCNK<br>CNFILGPFFQSQNQE<br>VRPGSCPECQSFGPF<br>EINMEETVYQNYQRI<br>TIQESPGKVAAGRLP<br>RSKDAILLADLVDSCK<br>PGDEIELTGIYHNNYD<br>GSLNTANGFPVFATV<br>ILANHITKKDDKVAV
PWZ26529	ZmMCM2	Z. mays	MCM2	PGFSTDRLPPNTTTSR<br>GATDPSSYSDDDGEA<br>EVDPHVLPDDDDPA<br>AVVAPDEDEEGEDLF<br>NDNYLDDYRRMDEH<br>DQYESVGLDDSLEDE<br>RNLDEIMADRRAAEA<br>ELDAREVRTGATADR<br>KLPRMLHDQDTDDD<br>TNFRRPKRHRASFRQ<br>PSGPRTPRSDDDGDA<br>ATPSSPGRSHRGMSS<br>GGDVPMTDQTDDD<br>PYEDEFDEEDEMNM<br>YRVQGTLREWVTRD<br>EVRRFIAKKFKEFLLTY<br>VHPKNDQGEFEYVRL<br>INEMVLANKCSLEIDY<br>KQFIYIHPNIAIWLAD<br>APQSVLEVMEEVAK<br>NVVFDLHKNYRNIHQ<br>KIYVRISNLPVYDQIR<br>NIRQIHLNTMIRIGGV<br>VTRRSGVFPQLQQTI<br>YRNYQKLTLQESPGIV<br>PAGRLPRYKEVILLND<br>LIDCARPGEEIEVTGIY<br>TNNFDLSLNTKNGFP
NP_194494.2	AtIPO4	A thaliana	IPO4	DNDARRQAEDQIKRL<br>AKDPQVVPALVQHL<br>RTAKTPNVRQLAAVL<br>LRKRITGHWAKLSPQ<br>LKQHVKQSLIESITVE<br>NSPPVRRASANVVSV<br>VAKYAVPAGEWPDL<br>LTFLFQCSQSAQEDH<br>REVALILFSSLTETIGN<br>TFRPYFADLQALLLKC<br>MQDESSSRVRVAALK<br>AVGSFLEFTNDGDEV<br>VKFRDFIPSILDVSRKC<br>IASGEEDVAILAFEIFD<br>ELIESPAPLLGDSVKAI<br>VQFSLEVSCNQNLES<br>STRHQAIQIVSWLAK<br>YKYNSLKKHKLVIPILQ<br>VMCPLLAESSDQEDD<br>DDLAPDRASAEVIDTL<br>AMNLPKHVFLPVLEF<br>ASVHCQSTNLKFREA<br>SVTALGVISEGCFDL<br>MKEKLDTVLNIVLGA<br>LRDPELVVRGAASFAI<br>GQFAEHLQPEILSHY<br>QSVLPCLLIAIEDTSEE<br>VKEKSHYALAAFCEN

XP_006838378.1	AtrIPO4	A. trichopoda	IPO4	DNDARRQAEEQIKRL<br>AKDPHVIPALLQHMR<br>TAKTANVRQLSAVLL<br>RKKITGHWMKLSPQ<br>VKDSVKSALMESITVE<br>HSPPVRRASANVVSV<br>VAKYAVPAGEWPELL<br>PFLFQCSQSPQEDHR<br>EVALILFSSLTETIGEM<br>FRPHLATLQSLFLKSL<br>QDETSTRVRIAALKAV<br>GSFVEFVHDRDEVV<br>MVRELIPSILNVSRQC<br>LANGDEEVAVIAFEIF<br>DELVESPAPLLGSFVV<br>SIVQFSLEVSSSENLET<br>NTRHQAIQIISWLAK<br>YKPKSLVKHKLVVPIL<br>QVMCPLLAEASHGD<br>DDDDDLSADRAAAE<br>VIDTMAVNLPRKLVF<br>PPVLEFASIGYQNPN<br>PKYREASVMALGVVS<br>EGCFELMKNKLEDVL<br>RIVLEALKDPEQLVRG<br>AASFALGQFAEHLQP<br>EIVTYYESVLPCILNSI<br>GDASEEVQEKSYYAL
NP_490715.1	CeIMB-3	C. elegans	IPO4	ADNDIRKQAEEVYEKI<br>EGPTKVAALFEAYTH<br>HTNNSDARSTVLVFL<br>RRVLARDWDAIWET<br>LNEENKQRILAKVLE<br>MIVHETELSIKKKIADL<br>ISEIASNLIDDSGDMT<br>WGGVLELMDHCLKS<br>EDLTGNYIALLILRGCP<br>IIFGNRLAHFLPTLKV<br>VLEKCMATPDLQIKA<br>TAVRAVIAFAVDNDE<br>EKDVVRLMTSLVPNV<br>LQVCNETSDEDDSDG<br>PLGEFAELASSLPKCL<br>NTHMSQVLQVTLAIA<br>GNKEKNEMVRQNAI<br>EVICSYMESAPKGLKK<br>YAPGALGPILETLLSC<br>MTEMDDDVLNEWL<br>NEIEEEEDYEDIPIIAES<br>AIDRVACCINGKVML<br>PVFLPLVEKLLTSEDW<br>KMKHAALRAFSAVG<br>EGCQRSMEPHIEQIM<br>AHITNYVNDAHPRV<br>QYAACNAIGQMSSD<br>FAPTLQKKCHAAVIP
XP_646718.1	DdIPO4	D. discoideur	IPO4	RALLQPNNDIIKAAT<br>DKLNKLLKKSEYSLYLF<br>HLLESSPYDEIKQLSA<br>VLLRQKLVAHWTKFS<br>VESRKYIKDSILKLVIS<br>QPSQLVRRSISEVIIIIA<br>RLEVATGTWGDLFPF<br>LLQLSSSPDTIVRQIQI<br>HILDSLISNVDVFLKYF<br>PQLPTVLSQAVIDPQL<br>SVRALAVKAIGSSIYA<br>VQTDSKLKPFIDLIPTT<br>LQVIKQCIENEMEDD<br>VISAFEIFNDLVESPYS<br>SIKAHIPLIVNFSIEIVK<br>QPEIDNSIKTIALEFLE<br>TCIKYQPKILKNSNLL<br>NPILEILFKILTFESDSSI<br>DENDYEYNILQSASV<br>AIKECGKSYSSKLIYYPI<br>LPTLKQFLESENVNYR<br>NAVMVIIQQLSYGCIE<br>TMKDDLDNIIQFVLR<br>GLKDQEKKVRQSACV<br>TIGKLSQTLTPEIYKYT<br>NQVFPLLFQQLSDPD<br>DQFILRCCFALENFLL<br>NLDSQELIPILPNVMD

NP_730184.1	DmArtemis	D. melanogas	IPO4	ERIRESTAKMLKAYEN<br>PDSLLVLTQIVMSDRP<br>VQERQVAAVLLKRRV<br>KKLRHWQLVPAEHQ<br>AAIKSNMLQVLIAVK<br>EKTVKGTVAFIIGSLV<br>RHEEDKQNSWREEIL<br>KFIYERCSSPDPIESER<br>GSSIFSSLMDAAPDQ<br>FSNHTDTIFPLLAGILV<br>TAEANGNMATPTVH<br>NMLAGTCVLLPFISG<br>HSDAEQIMVKAVPLI<br>LKALAAFAEKGNSNE<br>FMGAFDIIDSMAEYV<br>PHLLTGNVKLLLEFCL<br>MIARNKQFDASIRVQ<br>VLTFVGSLVRLKKKII<br>MKQKLLQPTLSVLFE<br>VICQDDLKEDDDYFSS<br>ESLNSPSNAAAQTLD<br>LMALHMVPDKFIPPL<br>LDLLEPALQSPEPVLR<br>RSSFICMGVIAEGCSE<br>AIGKKYLEVMLNIIKA<br>GVLDSVMFVRTAAFF<br>ALGQFSEFLQPTICKF<br>APQILPVLFDYLNQLV
NP_730182.1	DmApollo	D. melanogas	IPO4	ERIRESTAKMLKAYEN<br>PDSLLVLTQIVMSDRP<br>VQERQVAAVLLKRRV<br>KKLRHWQLVPAEHQ<br>AAIKSNMLQVLIAVK<br>EKTVKGTVAFIIGSLV<br>RHEEGEQNSWREEIL<br>KFIYERCSSPDPIESER<br>GSSIFSSLMDAAPDQ<br>FSDHTDTMFPLLAGIL<br>VTAEANGNMATPTV<br>HNMLTGSCFLLPFVS<br>GHSNAEQIVVKAVPL<br>ILKALAAFVEKGYSIEF<br>MGAFDIIDSMAEHVP<br>HLLTGNVKLLLEFCLM<br>IARNKQFDASIRVQV<br>LTFVGSLVRLKKKIIM<br>KQKLLQPTLSVLFEVI<br>CQDDLEEGDDDYFSS<br>ESLSSPSNAAAQTLDL<br>MALHMVPDKFIPPLL<br>DLLEPALQSPEPVLRR<br>SSFICMGVIAEGCSEA<br>IGKKYLEVMLNIIKAG<br>VLDSVMFVRTAAFFA<br>LGQFSEFLQPTICKFA<br>PQILPVLFDYLNQLVL
F1QIP3	DrIPO4	D. rerio	IPO4	NAVIQQATAELKQAF<br>KDPAIIPALCAVMTG<br>SQNPQVLPFIPAFIKF<br>SLFFNVKLLCVLLQVR<br>QSAAVMLRMRVRK<br>QWKKISPDHRESLKA<br>VVLQAFQQETEHTVR<br>HSLSQLSAVLVKHETP<br>DRWPALLELLNQSTK<br>SNNPQDRQVGLLLLS<br>KVVGSNPEPFKPHYK<br>QLLQLFGTVLQDLNN<br>PTALYYCILTLTAITAY<br>TGTEEMNLMRSLIPK<br>LLVALKHLIQADQDQ<br>ASEAMEVFDELMESE<br>VSIVVPHIAEIVRFCLE<br>ISADASLSDSLRVKAL<br>SCIAVLIRLKNKAVLK<br>HKLLQPILQVVFPILSA<br>APPPGEEDPEDEEND<br>TGGDSENPKHFAVQ<br>VIDTMALHMPPEKLF<br>NQLLPFTQACLSSEN<br>PYERKGGLMCMAVL<br>AEGCADHIRTKMLSS<br>MLQTVCRSLSDNNQ<br>VVRSAALFALGQFSE

NP_078934.3	HsIPO4	H. sapiens	IPO4	DTERIRRATEQLQIVL<br>RAPAALPALCDLLAS<br>AADPQIRQFAAVLTR<br>RRLNTRWRRLAAEQ<br>RESLKSLILTALQRETE<br>HCVSLSLAQLSATIFR<br>KEGLEAWPQLLQLLQ<br>HSTHSPHSPEREMGL<br>LLLSVVVTSRPEAFQP<br>HHRELLRLLNETLGEV<br>GSPGLLFYSLRTLTTM<br>APYLSTEDVPLARML<br>VPKLIMAMQTLIPIDE<br>AKACEALEALDELLES<br>EVPVITPYLSEVLTFCL<br>EVARNVALGNAIRIRI<br>LCCLTFLVKVKSKALLK<br>NRLLPPLLHTLFPIVA<br>AEPPPGQLDPEDQDS<br>EEEELEIELMGETPKH<br>FAVQVVDMLALHLP<br>PEKLCPQLMPMLEEA<br>LRSESPYQRKAGLLVL<br>AVLSDGAGDHIRQRL<br>LPPLLQIVCKGLEDPS<br>QVVRNAALFALGQFS<br>ENLQPHISSYSREVM<br>PLLLAYLKSVPLGHTH
NP_077229.4	MmIPO4	M. musculus	IPO4	DTERIRRATEQLQTIL<br>RDPAALPALFDLLATA<br>TDSQIRQFAAVLTRR<br>RLNNRWRRLAPEQR<br>ESLKSLVLTALQKETV<br>HSVSVSLAQLSATIFR<br>KEGLQGWPQFMNLL<br>QHSTHSSHSPEKEVG<br>LLLLSVVVSSQPEAFH<br>AHQHELLQLLNETLS<br>DVSFPGVLFYSLRTLT<br>AIARYVRPDDVSLAR<br>MLVPKVVTALRTLIPL<br>DEVKACEALEALDEM<br>LETELPIINPHLSEVLT<br>FCLEVAKNVALGEPL<br>RVRVLCCLTFLVKVKS<br>KALLKNRLVPPLLHAL<br>FPLMAAEPPMGQLD<br>PEDQDSDDDDLEIGL<br>MGETPKHFAVQVVD<br>MLALHLPPEKLCPHV<br>MPMLEEALRSEDPYQ<br>RKAGFLVLAVLSDGA<br>GDHIRQRLLYPLLQIV<br>CKGLDDPSQIVRNAA<br>LFALGQFSENLQPHIS<br>SYSEEVMPLLLSYLKS
XP_024374755.1	PpaIPO4	P. patens	IPO4	DNASRKQAEEQIRRL<br>SKDPLLVPALLHHVRC<br>SPYPEVRQLAAVLLRK<br>KITGHWMQLSAEMR<br>NNVKSTLLESITLENSP<br>PVRRGSADVVSVVAK<br>HAVPAGEWPELLPFL<br>HQCSQSAQEDHREV<br>ALILFSSLTETIGDLLRP<br>HFATLQSVFITGLND<br>QQSNRVRVAALKAV<br>GALVGYIQSEQEVM<br>MFRELIPPILNVSRLCL<br>ANGDEDVAILAFEIFD<br>ELIESAAPVLGPTIPVI<br>VQFALEVCSNKHLEA<br>NTRHQAIQIIFWLAK<br>YKPKTLVKHKMVTPIL<br>SVICPILAEPESRTHED<br>DIACERAAAEVLDTM<br>ATSLPKKHVFPPVLHF<br>ATSNFHNPDPNYRD<br>AAVMSLGVISEGCYE<br>AMKSRLEDVLSLVLEA<br>LKDKEQAVRGAASFA<br>LGQFAEHLQPEISEHY<br>ERVLPCIFAVLSDAVP<br>DVQEKAFYALAAFCE

-	PpIPO4	P. polycephal	IPO4	ALHPNTEIVRAAVTQ<br>LNAALTDPQSTMLFL<br>ALLQQSPHTHVKQFA<br>AVVMRKRVVGHYYK<br>LPQNERGVLKASLLL<br>MLHSDTKVEVIVRRA<br>VADVVVTLGKAIVPR<br>GDWNVLLPTILNAAS<br>SAVLIEDREIAMYLLA<br>AVIDSLGDQMREHY<br>ASLHTLLAACLRDSNP<br>SIRSLALHTTSALIPWL<br>VTKDDIALYGSLIPLVV<br>DVVKYFVQNEMDDD<br>AIESFSILEDLVETPIPV<br>ITKFIPALVNLMLEICA<br>NHQVDISVRQSALSF<br>VQLVVRVKPKMVVK<br>NKLVTPTLAAAFAMC<br>AEGEGNDEAQQDLY<br>EQEETLSPYIFGSQVI<br>DTVALSLPVNQVIGP<br>SLALVAQYGRPEMHF<br>RQRRAAITAITVLTEG<br>CAESIKDTLAKVVQYV<br>VGALVDPSFLVRQAA<br>CLAIGQCADNLQPEII<br>DYHNVVVPALLQGLR
NP_011035.1	ScKap123	S. cerevisiae	IPO4	AITSGVGLKEATKTLQ<br>TQFYTQPTTLPALIHIL<br>QNGSDDSLKQLAGV<br>EARKLVSKHWNAIDE<br>STRASIKTSLLQTAFSE<br>PKENVRHSNARVIASI<br>GTEELDGNKWPDLV<br>PNLIQTASGEDVQTR<br>QTAIFILFSLLEDFTSSL<br>SGHIDDFLALFSQTIN<br>DPSSLEIRSLSAQALN<br>HVSALIEEQETINPVQ<br>AQKFAASIPSVVNVL<br>DAVIKADDTMNAKLI<br>FNCLNDFLLLDSQLTG<br>NFIVDLIKLSLQIAVNS<br>EIDEDVRVFALQFIISS<br>LSYRKSKVSQSKLGPE<br>ITVAALKVACEEIDVD<br>DELNNEDETGENEEN<br>TPSSSAIRLLAFASSEL<br>PPSQVASVIVEHIPA<br>MLQSANVFERRAILL<br>AISVAVTGSPDYILSQ<br>FDKIIPATINGLKDTEP<br>IVKLAALKCIHQLTTD<br>LQDEVAKFHEEYLPLII<br>DIIDSAKNIVIYNYATV
NA	NA	T. thermophi	IPO4	
XP_018114829.1	XlIPO4	X. laevis	IPO4	NAVIQQATAQLKEAF<br>KDPQIIPALFEILRGSQ<br>ELQIRQFAAVLLRRRL<br>NKHWKAVQPEQQH<br>NLKIIVLESIQREPEHK<br>VRYALAQLIAVILKNE<br>RLEHWPEFIKFVLQLS<br>HSDVPDQKQVGILVL<br>WCSLHLKASLFQPHV<br>HDLLGLFKQTLSDLN<br>NGPLIYYTVQSLTCILP<br>YIVGNETNLLRPFIPKI<br>LAAIRQLILVNQVQA<br>CEAMEFFDVLMEDE<br>VPVIVHYIADTVQFCL<br>EMAVNTSLSDELRVK<br>ALSCIMCLIKLKSKSIIK<br>QKLLPQILNSLFPIMC<br>AAPPAGEMDKEDQE<br>EEDDDIEDSVETPKEY<br>AMQVIDMLALHLPP<br>EKLFKELSPLMEPCLLS<br>SNPYPRKAGLMCLAV<br>LSEGCSDFICDKHLQP<br>MLSLVCQSLSDDNQV<br>VRNAALYALGQFSEH<br>LQPDITNYSDTVLPLL<br>LEYFSRVDPSNTAHLT

ONL96235.1	ZmIPO4	Z. mays	IPO4	DNDARRQAEEQIRRL<br>ARDPQVVPALVHHL<br>RTAKTPNVRQLAAVL<br>LRKKITSHWPKLPADS<br>KASLKQALIDSITLDHS<br>HPVRRASANVVSIIAK<br>YAIPAGEWPELLPFLF<br>QCSQSPQEDHREVAL<br>ILFSSLTETIGATFQSH<br>LNNLQPILLKCLQDET<br>SSRVRIAALKAVGSFI<br>EYVNDGGDIVKMFR<br>DFVPSILNISRQCLAN<br>GEEDVASIAFEIFDELI<br>ESPAPLLGDSVRSIVQ<br>FSLEVSANQDLEINIR<br>QQAIQIISWLVKFKAS<br>FLKKHKLVVPILQVM<br>CPLLTETANEDEDSDL<br>AADRSAAEVIDTMAI<br>NLPRHVLAPVLEFASL<br>SFHHINPKYREAAVTS<br>LGVISEGCCEHLKDKL<br>EDCLKIVLEALKDQEQ<br>MVRGAASFALGQFA<br>EHLQPEILSHYASVLP<br>CILNALEDPSDEVKEK<br>SYYALAAFCEDMGED
NP_001329034.1	AtSPT16	A thaliana	SPT16	GVPPKAGNTYSIDVK<br>NFISRARALYEHWKK<br>HSADLWGSADALAIA<br>TPPASDDLRYLKSSAL<br>NIWLLGYEFPDTIMV<br>FTKKQIHFLCSRNKAS<br>LLEVVKKPAHDELKLD<br>VIMHVKPKGDDGTG<br>LMDAIFRAIRDLSRG<br>DGNDSQVVGHIARE<br>APEGKLLETWTERLK<br>NANFQFVDITGGLSD<br>LFAVKDDTEVMSVKK<br>AAYLAYSVMKNVVV<br>PNLESAIDEEKDVTHS<br>ALMDLTEKAILEPTKA<br>SVKLKPENVDICYPPIF<br>QSGGKFDLKPSAASN<br>DELLTYDPASIIICAVG<br>ARYNSYCSNVARTYLI<br>DATSLQSKAYEVLLKA<br>HEAAIDALRSGRKINT<br>VYQAALSVVEKNAPE<br>FVDKLTKSAGTGIGLE<br>FRESGLNINAKNDKV<br>LRPKMAFNVSLGFQ<br>NLECESESRSKNKKFS<br>LLLADTVLVTDQKPEL
W1NNZ7	AtrSPT16	A. trichopoda	SPT16	NASKSSSYQIDLDTFS<br>KRLKAFYSSWEENRD<br>SLWASADAIAVATPP<br>PSDDLRYLKSSALNIW<br>LLGYEFPETIMVFTSK<br>QIHFLCSQKKANLLET<br>LGKSAKSALNLEVLIH<br>VKAKNEDGASQMEEI<br>FNAIKSHAESEKVVV<br>GHIAREAAEGKLLET<br>WREKLELNFQLGDVT<br>NGFSELFAVKDENEIT<br>NVKKASFLSASVLKNF<br>VVPKLEVIIDEEKKVT<br>HSSLMDDTEKAILDP<br>AKSKVKLKAENVDICY<br>PPIFQSGGQFDLRPN<br>SSSNDDHLYYDATSVI<br>ICAIGSRYNSYCSNVA<br>RTYLIDADDIQNRAY<br>NVLFKAHEAAIGSLKP<br>GKKIGEAYKAAMEVV<br>EREAPEFASNLTKSAG<br>TGIGLEFQESGLRLNA<br>KNDRLVKAGMIFNVS<br>LGFQNLKAPTNNPKT<br>ESFSLLLADTVIVREN<br>VPEVATSTSSKAFKDV

NP_492821.1	CeSPT16	C. elegans	SPT16	RAERLYEHWEKGAD<br>GLDSIKSLAFVYGETD<br>NPYTKTSALFTWLFG<br>HEIADTVLLLLKDHIYI<br>LGSNRKVEFFGSVTG<br>DNQSSGKVPTVSTLL<br>RDKTDKDAGNFEKLI<br>DHIKSAGGDVGNFVK<br>EKFSSEFVSSWNKALE<br>EGGVNKNDVTLAFTH<br>LFAVKDDKEMDLIRK<br>SAQATTASWTAARA<br>RYVEIIDQEKRVRHSV<br>LSNEFAAFMKDSKVQ<br>QALAKYEADTCYDPI<br>VMSGGNYSFKWNHE<br>SSESHLHSQFGTIITSF<br>GARLSEYCTNLTRTM<br>LIFPSSELETAYEAILA<br>AELAVIAALKPGAKLS<br>DVYKIGIDTLTEKSPKL<br>AETLNKKELGFATGIE<br>FRESRLAISAKCDEVV<br>KAGMVFIVYIGVDSIP<br>NKNKGEKGKPAAIAI<br>SDTILVKEEGDNEILTE<br>KAKSRLKSNVIKFKEE<br>QENREAEKDNDQKK
XP_640196.1	DdSPT16	D. discoideur	SPT16	AVPTGPREATLDAGN<br>FCKRVKILYDSWNSD<br>SNLWKSANSLVLALG<br>QPNESNPYQKVTSLQ<br>TWLFGYELKDTIIVFLE<br>KEIYIVSTSKKINLFQK<br>LSETEQVKTELSSIKFN<br>FLTIDKSDKNKSNFEK<br>LIGEATKAGSNIGVIIK<br>ETYIGDLALQWEAAL<br>NECPLTKVDITPALSS<br>CLLVKDLQEQKNIITS<br>AKITSKVLKSHILPKIET<br>IIDKGERQTHNQLAD<br>YAADIFESPEKISSKLT<br>VEHVDYSYVPIIQSGG<br>IYDLRASASSDDNPLH<br>FGTIIVSCGARYKNYC<br>SNIARTYIIDPTSDQK<br>KNYAILLNVQSNVIKA<br>IKPDVTFSSLYEKAIQT<br>IKESSKPELVDHFPKN<br>VGYGIGIEFQESLAVL<br>NATNSRTLKAGMTL<br>NIACGFQKISNPEGK<br>DEKSKTYSLLISDTVLL<br>NDEGKVEVLTDVGKK<br>ASDVVYMLGGEDDD
NP_476610.2	DmSPT16	D. melanogas	SPT16	KRLYTEWRAPSIGHD<br>DALRNLDCIMSIVGV<br>EEDVMYSKSMALQL<br>WLLGYELTDTISVFCS<br>DAVYFLTSKKKIEFLK<br>QTQNITEEGFPEINLL<br>VRDRTDKDQGNFEKL<br>IKALQNSKKGKRLGV<br>FAKDAYPGEFSEAWK<br>KSLTASKFEHVDISTII<br>AYLMCPKDESEINNIR<br>KASLVSMDIFNKYLK<br>DEIMDIIDSDRKVKH<br>NKLSDGCEAAIGEKK<br>YTSGLDPRLLDMAYP<br>PIIQSGGAYSLKFSAV<br>ADKNPLHFGVIVCSL<br>GARYKSYCSNISRTFL<br>VNPTEAMQENYTFLV<br>SVQEEILKLLVPGTKLC<br>DVYEKTLDFVKKEKPS<br>MVDNLPKSFGFAMG<br>LEFRENSIVIGPKCQA<br>LLKKNMVFNLHVGIS<br>NLTNPEATDKEGKNY<br>ALFIGDTVLVGEQSPA<br>SVMTPSKKKIKNVGIF<br>IKDDSDEEDVDDKKT

NP_001091053.1	DrSPT16	*D. rerio*	SPT16	RLYGNWKKGEDEFG<br>KVDAIVVSVGVDEEIV<br>YAKSTALQTWLFGYE<br>LTDTIMVFCESKIIFLA<br>SKKKVEFLKQVAVTK<br>GNENANGVPPITLLV<br>REKNESNKVNFDKMI<br>EAIRGSKEGKTVGVFI<br>KDKFPGEYMKSWSD<br>TITAEGLQKVDISTVV<br>AYTMAVKEDGELAL<br>MKKAASITTDVFSKFF<br>KERVMEIVDADEKVK<br>HSRLAESVEKAIEDRK<br>FLGGVDPSTVEMCYP<br>PIIQSGGNYSLKFSVV<br>SDKNHMHFGAITCA<br>MGIRYKSYCSNLVRTL<br>MVDPSQEMQDNYN<br>FLLQVEEELLKEMKH<br>GVKLSEAYNAVMEFV<br>KKEKPDLVSKLTKNLG<br>FAMGIEFREGSLVIN<br>QKNQFKLKRGMVFSI<br>SLGFADMINKEGKKE<br>EQKKYALFIGDTIQIN<br>EEDQATVLTPVKKKIK<br>NVGIFLKNDDEDEDE
NP_009123.1	HsSPT16	*H. sapiens*	SPT16	RLYSNWRKGEDEYA<br>NVDAIVVSVGVDEEI<br>VYAKSTALQTWLFGY<br>ELTDTIMVFCDDKIIF<br>MASKKKVEFLKQIAN<br>TKGNENANGAPAITL<br>LIREKNESNKSSFDKM<br>IEAIKESKNGKKIGVFS<br>KDKFPGEFMKSWND<br>CLNKEGFDKIDISAVV<br>AYTIAVKEDGELNLM<br>KKAASITSEVFNKFFK<br>ERVMEIVDADEKVRH<br>SKLAESVEKAIEEKKYL<br>AGADPSTVEMCYPPII<br>QSGGNYNLKFSVVSD<br>KNHMHFGAITCAMG<br>IRFKSYCSNLVRTLMV<br>DPSQEVQENYNFLLQ<br>LQEELLKELRHGVKIC<br>DVYNAVMDVVKKQK<br>PELLNKITKNLGFGM<br>GIEFREGSLVINSKNQ<br>YKLKKGMVFSINLGFS<br>DLTNKEGKKPEEKTY<br>ALFIGDTVLVDEDGP<br>ATVLTSVKKKVKNVGI<br>FLKNEDEEEEEEEKDE
NP_291096.2	MmSPT16	*M. musculus*	SPT16	RLYSNWRKGEDEYAS<br>IDAIVVSVGVDEEIVY<br>AKSTALQTWLFGYEL<br>TDTIMVFCDDKIIFM<br>ASKKKVEFLKQIANTK<br>GNENANGAPAITLLV<br>REKNESNKSSFDKMI<br>DAIKESKSGKKIGVFS<br>KDKFPGEFMKSWSD<br>CLNKEGFDKVDISAV<br>VAYTIAVKEDGELNL<br>MKKAASITSEVFNKFF<br>KERVMEIVDADEKVR<br>HSKLAESVEKAIEEKK<br>YLAGADPSTVEMCYP<br>PIIQSGGNYNLKFSVV<br>SDKNHMHFGAITCA<br>MGIRFKSYCSNLVRTL<br>MVDPTQEVQENYNF<br>LLQLQEELLKELRHGV<br>KICDVYNSVMDVVKK<br>QKPELLNKITKNLGFG<br>MGIEFREGSLVINSKN<br>QYKLKKGMVFSINLG<br>FSDLTNKEGKKPEEKT<br>YALFIGDTVLVDEDGP<br>ATILTSVKKKVKNVGI<br>FLKNEDDEEEEEEKDE

XP_001783705.1	PpaSPT16	P. patens	SPT16	QINLELYGKRLKLLNN<br>KWKEHKKEMWGGA<br>DAIAVVTPPASEDLRY<br>LKSTALHIWLLGYEFP<br>ETVMVFMPGALHFV<br>CSSKKAAHLEELQKSS<br>KMLTGVDIHIHMKER<br>KQDGSVQMNSVLDA<br>VKGFSKGKTPTVGVL<br>SREATEGSVMEKWA<br>ECLEASGAATVDVSG<br>GFSEIFAVKDEEEISNI<br>KNASHLSAAILKSFVV<br>PKLEVIIDEEKKVTHFE<br>LMEQTENVITNPGKY<br>VKLKAEDVDICYPPVF<br>QSGGVFDLKPSAVSN<br>EEPLYYDTLGVILCAIG<br>ARFRSYCSNVARSIMI<br>DADKTQEKAYKILLKA<br>HEAAIAALRPGNAM<br>SVAYKAAYAVVESGG<br>PEFLPYFTKNAGTGIG<br>IEFRESGLTLNAKNER<br>VIRPGMAFNVSLGFH<br>NLTTESSNPKSKTFSLL<br>LADTAIVVEKGPPEVP<br>TLKCSKTYTDIAYSFKD
-	PpSPT16	P. polycephal	SPT16	KLDSKSFWRRVKALY<br>DSWQKKDDYLWKGP<br>DALVIAQGTPDDDNP<br>YKKTNALQYWLLGYE<br>FADTIIVFCESNIYVLT<br>SPKKIGLLEVLKEENA<br>PYVLCFLQLSKAENN<br>QNLNTLFEAVHKSKS<br>GKSIGVLPKEKMQGA<br>LGSLWEEALAKSGLTL<br>VDVAPGYSEMLSVKD<br>TVEMKLLKQAAETTS<br>KVMSNFLIPAVETVID<br>EGTQKSHTALAEEME<br>EVFAQPEKINSKLVGE<br>HVESCYIPIIQSGGKY<br>DLKLSATNDNENLHF<br>GTIVCALGARYKGYCS<br>NAARTFFIDPKKEVEI<br>NYRFLLEVHNIVVKSI<br>KPGVKISVIMDKATKY<br>IADKRPELSSSFLKCC<br>GYGIGIEFQETYLMLS<br>SKNTRTIKPGMAFNI<br>MVGFQNLELKEEPKD<br>PKNKVYSMLLADTVI<br>VSETGEAEVLTNSVSK<br>RFGDVSYFIEELDNEE
NP_011308.1	ScSPT16	S. cerevisiae	SPT16	LLYSKYNEFEGSPNSL<br>LFVLGSSNAENPYQK<br>TTILHNWLLSYEFPAT<br>LIALVPGKVIIITSSAKA<br>KHLQKAIDLFKDPESK<br>ITLELWQRNNKEPEL<br>NKKLFDDVIALINSAG<br>KTVGIPEKDSYQGKF<br>MTEWNPVWEAAVK<br>ENEFNVIDISLGLSKV<br>WEVKDVNEQAFLSV<br>SSKGSDKFMDLLSNE<br>MVRAVDEELKITNAK<br>LSDKIENKIDDVKFLK<br>QLSPDLSALCPPNYKF<br>NFDLLDWTYSPIIQSG<br>KKFDLRVSARSTNDQ<br>LYGNGCILASCGIRYN<br>NYCSNITRTFLIDPSEE<br>MANNYDFLLTLQKEI<br>VTNILKPGRTPKEVYE<br>SVIEYIEKTKPELVPNF<br>TKNIGSLIGLEFRDSN<br>FILNVKNDYRKIQRG<br>DCFNISFGFNNLKDS<br>QSANNYALQLADTV<br>QIPLDETEPPRFLTNY<br>TKAKSQISFYFNNEEE

XP_001018189.2	TtSPT16	T. thermophi	SPT16	QLLSVWNKQPQYAN<br>IDAFIIKNGKDQGGN<br>KIKTSAISMWYFGFD<br>FIDTILLITKKTFAIIGG<br>NKKINMLKSVQEHAE<br>AKEYNLVFIEKDQAN<br>NSNQLQQLFEILDKD<br>LNKSSFNIGTLAKEQQ<br>VGPFMTEYDSFIKDK<br>NQYKFADCSVFVQD<br>CLSVKDQNEISYIGKA<br>AKVSVYLESKLIKEIETI<br>IEDEGKKTHSQIATMI<br>EGLIENEKELKKISEEI<br>GGESDNLDLAYVPIV<br>QSGGKYDLKPNAQS<br>NEDILSYDTIIVSVGTK<br>YMEYHANIVRTLFIDP<br>TNDQKKIYQRVYELQ<br>NQIAVQLKPGIKLKTV<br>YENAVNFINEKVPQL<br>KDKIPANFGFGIGLEF<br>RESNLYINAKNEKEVE<br>EGMVFNVVVGFDNL<br>QSEKEKAYAIQISDTV<br>AIRKQNTPNAVMTFK<br>VSKKYEDISYSIQDEG<br>QDEEQEEEEDDLEKE
NP_001084166.1	XlSPT16	X. laevis	SPT16	RFFGSWKKGDDEFA<br>NVDAIVVSVGVDEEI<br>VYAKSTALQTWLFGY<br>ELTDTIMVFCEEKILF<br>MASKKKVEFLKQIAN<br>TKGNENANGTPAITL<br>LVREKQNESNKGNFD<br>KMIEAIKVSKKGKRIG<br>VFIKDKFPGDFMKSW<br>YDILNKESFEKVDISAS<br>VAYTIAVKEEGELNL<br>MKKAASITSDVFSKFF<br>KDRVMEIVDADEKVR<br>HGKLAESVEKAIEDKK<br>YLGGTDPSTIEMCYPP<br>IIQSGGNYNLKFSVVS<br>DKNHMHFGAITCAL<br>GIRYKSYCSNLVRTLM<br>VDPTQEMQENYNFL<br>LQLQEELLKELKHGAK<br>ICDAYQVIMDQVKK<br>QKPDLMSKITKTLGF<br>AMGIEFREGSLVINN<br>KNQYKLKKGMVFSV<br>HLGLAELNNKMGKK<br>PEEKTYALFVGDTVLV<br>NEEGAATVLTNVKKK<br>VKNVGIFLKKEDEEEE
NP_001105557.1	ZmSPT16	Z. mays	SPT16	YAINIENFSKRLKVFY<br>DHWKEHKSDLWGSS<br>DAIAIATPPPSDDLRY<br>LKSSALDIWLLGYEFP<br>ETIIVFMHKQIHVLSS<br>QKKGNLIGTLKKAAN<br>EAVGVDIVLHVKTKN<br>SDGADLMDDIVHAA<br>RNQSKSDKPVVGHIA<br>KEAPEGKLLETWIKKL<br>SGSGLRLVDVTNGFS<br>ELFAVKDTTEITCVKK<br>AAYLTSSVLKNFVIPKL<br>EKVIDEEKEVSHSSLM<br>DDAEKAILDPLKVKVK<br>LKPDNVDICYPPVFQ<br>SGGKFDLKPGASSND<br>EYLYYDSASIIICAIGSK<br>YSSYCSNVARTYLIDA<br>TPTQNKAYETLRKAH<br>EAAIQQVKPGNQMS<br>AVYQAAVAVIERDAP<br>ELLPNLTKSAGTGIGL<br>EFRESGLNLNAKNDR<br>RIKKGMVFNVSLGLH<br>NIQAETTSEKTKQFSL<br>LLADTVLVNERGHEIL<br>TAPCSKAFKDVAYSF

NP_189515.1	AtSSRP1	A thaliana	SSRP1	GGKNPGLLKINSGGI<br>QWKKQGGGKAVEV<br>DRSDIVSVSWTKVTK<br>SNQLGVKTKDGLYYK<br>FVGFRDQDVPSLSSFF<br>QSSYGKTPDEKQLSV<br>SGRNWGEVDLHGNT<br>LTFLVGSKQAFEVSLA<br>DVSQTQLQGKNDVT<br>LEFHVDDTAGANEKD<br>SLMEISFHIPNSNTQF<br>VGDENRPPSQVFND<br>TIVAMADVSPGVEDA<br>VVTFESIAILTPRGRY<br>NVELHLSFLRLQGQA<br>NDFKIQYSSVVRLFLL<br>PKSNQPHTFVVISLDP<br>PIRKGQTMYPHIVM<br>QFETDTVVESELSISD<br>ELMNTKFKDKLERSY<br>KGLIHEVFTTVLRWLS<br>GAKITKPGKFRSSQD<br>GFAVKSSLKAEDGVL<br>YPLEKGFFFLPKPPTLI<br>LHDEIDYVEFERHAA<br>GGANMHYFDLLIRLK<br>TDHEHLFRNIQRNEY<br>HNLYTFISSKGLKIMN
XP_006842123.1	AtrSSRP1	A. trichopoda	SSRP1	RGGTNPGQLRIHSGG<br>IVWRKQGGGKVVEV<br>GKSDLVGVSWMKVP<br>KSYQLGVRIKAGLVYK<br>FIGFREQDVNNLNSFI<br>SNTLGITPQEKQLSVS<br>GRNFGEIELNGNMLT<br>FLVGSKQAFEVSLAD<br>VSQTQLQGKNDVSLE<br>FHVDDTTGANEKDSL<br>VELAFHIPNSNTTFVG<br>DETRPPAQVFRDKIM<br>SMADVGPSGEEAVV<br>TFDGIAVLTPRGRYTV<br>ELHISFFRLLGQANDF<br>KIQYSSVVRLFVLPKF<br>NQPHTFVIITLDPPIR<br>KGQTLYPHIVLQFETE<br>YVVESTLSISEDLLSTK<br>YKDRLEASYKGLLYDV<br>FTAILRGLSGAKLTRP<br>GKFRSCQDGYAVKSS<br>LKAEDGILYPLEKSFFF<br>LPKPPTLILHDEIECLE<br>FERHGAGGSSISSHYF<br>DLLVRLKNEQEHLFR<br>NIQRNEYHNLFEFINS<br>KGLKITNLGETQATG
NP_498633.1	CeSSRP1-A	C. elegans	SSRP1	GHLAFGTLKLTEKSLN<br>FKGDKGGKSVNVTGS<br>DIDKLKWQKLGNKP<br>GLRVGLNDGGAHRF<br>GGFKDTDLEKIQSFTS<br>SNWSQSIDQSNLFIK<br>GWNYGQAEVKGKTV<br>EFSWEDKPIFEIPCTN<br>VSNVIANKNEAVLEF<br>HQNDDSKVQLMEM<br>RFHMPIDLENEEDAD<br>KVEEFKKAVLAYAGLE<br>AETEQPICLLTDILCTT<br>PRGRYDIKVYPTSIAL<br>HGKTYDYKIPIKSINRL<br>FLVPHKDGRHVYFVL<br>SLNPPIRQGQTRYSYL<br>IFEFGKDEEQDLELAL<br>TDEQLESSNGNLRRD<br>MTGPIYETISILFKSIC<br>NLKITVPGRFLGSSGT<br>PAIQCTHRQNPGLLY<br>PMEKGFLFIHKPAMY<br>IRFEEISSCHFARSDSG<br>TVTRTFDFEIDLKYGG<br>PLTFNAMEKEENNKL<br>FDYLNKKNIKIRNSQR<br>VENTVADSSDDEIDP

NP_491688.1	CeSSRP1-B	C. elegans	SSRP1	HLTCGTLTLTENSINFI<br>GDKGGKSVYITGTDV<br>DKLKWQKLGNKPGL<br>RVGLSDGGAHRFGG<br>FLDDDLQKIQSFTSSN<br>WSKSINQSNLFINGW<br>NYGQADVKGKNIEFS<br>WENEPIFEIPCTNVSN<br>VIANKNEAILEFHQNE<br>QSKVQLMEMRFHM<br>PVDLENEEDTDKVEE<br>FKKAVLAYAGLEAETE<br>QPICLLTDILCTTPRGR<br>YDIKVYPTSIALHGKT<br>YDYKIPVKTINRLFLVP<br>HKDGRQVYFVLSLNP<br>PIRQGQTHYSYLIFEF<br>GKDEEEDLELSLTDEQ<br>LDYFNGNLQREMTG<br>PIYETISILFKSICNLKV<br>TVPGRFLGSSGTPAIQ<br>CTHRQNLGLLYPMEK<br>GFLFIQKPVMYIRFEEI<br>SSCHFARSDSGTVTR<br>TFDFEIDLKTGSSLTFS<br>AMDKEENNKLFDYL<br>NKKEIKIRNSHRIDNK<br>SAGYGSSDEDDIDPY
XP_635805.1	DdSSRP1	D. discoideur	SSRP1	LGGRISGTRGILKFTT<br>NNITWKSENGKIETV<br>SSSDIKRANWARVTP<br>RIFQLILSIKGGATVKF<br>DGFKEQDYEVVRKYL<br>SDQYNVSPLEIIELSSK<br>GCNWGEVKVNGPM<br>IQFTTDHGKVGFEFPI<br>SEVSQSVIGANNKNE<br>LTLEFHHDKAMDDD<br>DETMVEMRFFTPIRP<br>SKEGEEGGKEKKVGE<br>DGEEDEEDEEDAEKE<br>EEISALEQFQQTIMN<br>KSDMVSNVGKSLVVF<br>SAIQFLTPRGRIDIEM<br>YPTFLKLHGKTHDYKV<br>PYESISRLFQFFRPDQ<br>KHIFFIISLDPPIRQGQ<br>TKYAHLVIQFQAEENI<br>HLELNLTDELQQKFK<br>DQLSPIMNGNANALI<br>CKILKALTGKKITIPGN<br>FQSDSGANSIKCSLKA<br>NEGYLYPLERCFFFVH<br>KPPTYIKFEDISNIEFA<br>RYGAPSVRGGSNRTF<br>DLSINLKNSTSIQFVNI
NP_523830.2	DmSSRP1	D. melanogas	SSRP1	GVLCSGRLKMTEQNI<br>IFKNTKTGKVEQISAE<br>DIDLINSQKFVGTWG<br>LRVFTKGGVLHRFTG<br>FRDSEHEKLGKFIKAA<br>YSQEMVEKEMCVKG<br>WNWGTARFMGSVL<br>SFDKESKTIFEVPLSHV<br>SQCVTGKNEVTLEFH<br>QNDDAPVGLLEMRF<br>HIPAVESAEEDPVDKF<br>HQNVMSKASVISASG<br>ESIAIFREIQILTPRGRY<br>DIKIFSTFFQLHGKTF<br>DYKIPMDSVLRLFML<br>PHKDSRQMFFVLSLD<br>PPIKQGQTRYHYLVLL<br>FAPDEETTIELPFSEAE<br>LRDKYEGKLEKEISGP<br>VYEVMGKVMKVLIG<br>RKITGPGNFIGHSGT<br>AAVGCSFKAAAGYLY<br>PLERGFIYIHKPPLHIR<br>FEEISSVNFARSGGST<br>RSFDFEVTLKNGTVHI<br>FSSIEKEEYAKLFDYIT<br>QKKLHVSNMGKDKS<br>GYKDVDFGDSDNEN

A0A2R8QK93	DrSSRP1A	*D. rerio*	SSRP1	GSWNDGRLRFSKQTVVYKSHKTGKVDSIPAPDLSEAQWRRVCLGHGLKLATSTGHIYKYDGFKETDYEKISAFFKANYKVELEEKDMCVKGWNWGTAKFAGSLLSFDVSDSPVFEIPLSSVSQCATGKNEVTVEFHQNDDAEVSLMEVRFYVPPNTGDDGSDPVEAFAQNILSKADVIQATGDAVCIFKELQCLTPRGRYDIRIYPTFLHLHGKTFDYKIPYTTVLRLFLLPHKDQRQMFFVISLDPPIKQGQTRYHFLILLFSKDEDISLALNMSEDEVEKRYEGKLSKNMSGPLYEIVSRVMKALVNRKITVPGNFQGHSGSQCITCAYKASSGLLYPLERGFIYVHKPPVHLRFEEISCVNFARGTTTTRSFDFEIETKQNNQFTFSNIEREEYGKLFDFVNAKKLTIKNRGFKEGMKGAEDYSDSD
A0A2R8QBX6	DRSSRP1B	*D. rerio*	SSRP1	GSWNDGRLRFSKQTVVYKNSKTGKVDTIPVPELTQAQWRRVCLGHGIKLWTSTGHIYKYDGFKDADLEKISEYFKDNYKVELTEKDMCVKGWNWGTAKFNGPLLSFDVNDSPTFEIPLSSVSQCTTGKNEVTVEFHQNDDTEVSLMEVRFYVPPTTGDEGSDPVEAFAQNVLSKADVIQATGDAVCIFRELQCLTPRGRYDIRIYPTFLHLHGKTFDYKIPYTTVLRLFLLPHKDQRQMFFVISLDPPIKQGQTRYHFLILLFSKEETISLTLNMNEDEVERRFEGKLNKNMSGSLYEMVSRVMKALVNRKITVPGNFQGHSGAQCITCSYKASSGLLYPLERGFIYVHKPPVHLRFEEIACVNFARGTTTTRSFDFEIETKQGNQYTFSSIEREEYGKLFDFVNAKKLSIKNRGFKEGMKGNDDMY
NP_003137.1	HsSSRP1	*H. sapiens*	SSRP1	GSMNDGRLRLSRQGIIFKNSKTGKVDNIQAGELTEGIWRRVALGHGLKLLTKNGHVYKYDGFRESEFEKLSDFFKTHYRLELMEKDLCVKGWNWGTVKFGGQLLSFDIGDQPVFEIPLSNVSQCTTGKNEVTLEFHQNDDAEVSLMEVRFYVPPTQEDGVDPVEAFAQNVLSKADVIQATGDAICIFRELQCLTPRGRYDIRIYPTFLHLHGKTFDYKIPYTTVLRLFLLPHKDQRQMFFVISLDPPIKQGQTRYHFLILLFSKDEDISLTLNMNEEEVEKRFEGRLTKNMSGSLYEMVSRVMKALVNRKITVPGNFQGHSGAQCITCSYKASSGLLYPLERGFIYVHKPPVHIRFDEISFVNFARGTTTTRSFDFEIETKQGTQYTFSSIEREEYGKLFDFVNAKKLNIKNRGLKEGMNPSYDEYADSDE

NP_001129553.1	MmSSRP1	M. musculus	SSRP1	GSMNDGRLRLSRQGI<br>IFKNSKTGKVDNIQA<br>GELTEGIWRRVALGH<br>GLKLLTKNGHVYKYD<br>GFRESEFEKLSDFFKT<br>HYRLELMEKDLCVKG<br>WNWGTVKFGGQLLS<br>FDIGDQPVFEIPLSNV<br>SQCTTGKNEVTLEFH<br>QNDDAEVSLMEVRF<br>YVPPTQEDGVDPVEA<br>FAQNVLSKADVIQAT<br>GDAICIFRELQCLTPR<br>GRYDIRIYPTFLHLHG<br>KTFDYKIPYTTVLRLFL<br>LPHKDQRQMFFVISL<br>DPPIKQGQTRYHFLIL<br>LFSKDEDISLTLNMNE<br>EEVEKRFEGRLTKNM<br>SGSLYEMVSRVMKAL<br>VNRKITVPGNFQGHS<br>GAQCITCSYKASSGLL<br>YPLERGFIYVHKPPVH<br>IRFDEISFVNFARGTT<br>TTRSFDFEIETKQGTQ<br>YTFSSIEREEYGKLFDF<br>VNAKKLNIKNRGLKE<br>GINPGYDDYADSDED
A0A2K1K1Z7	PpaSSRP1	P. patens	SSRP1	GRGGTNPGQLKINSG<br>GFQWRKAGGGKVVE<br>VSTSDIASLSWTRVPK<br>GFQLGVKLKAGRDVK<br>FNGFRESDMANLGSF<br>FTTNMGITSEEKPLAI<br>SGRNWGDVTLEGSSL<br>AFSVGGKHAFDVSIA<br>DVSQTQMQGKNEV<br>MLEFHVDDTTGASEK<br>DTLMELSFHIPTTNTT<br>YIGDEERPPAQIFREKI<br>LSMGDVGPSGAEAIA<br>LFEEVHILTPRGRYNV<br>ELHASFLRLQGQAND<br>FKIQYSSVVRLFILPKS<br>NQPHTFVVITLDPPIR<br>KGQTFYPHIVLQFPSE<br>EIAECTLSIGEELLNTK<br>YKDRLQPAYKDLSHD<br>VFTQILKGLSGAKVTR<br>PGKFRSAQDGYCVRT<br>SLKAEEGTLYPLEKSFF<br>FLPKPPTLILHDEIEYLE<br>FERHGAAGTSSISSHY<br>FDLLIRLKSEQEHQFR<br>NIQRNEYHNLFNFISG<br>KNLKIMNLGDAQGTS
-	PpPO3	P. polycephal	SSRP1	GRVSSTKGVLKVNST<br>MFLWRTEAGRVISVL<br>PTDLSQAFWTRIYGR<br>VFQLKLQLNIGNSIKF<br>DGFRESDYDVLKTFLS<br>ENFHVELTTVQLATK<br>GLNWGEYELAGPVIT<br>LAVDGKHAFEIPVSEI<br>SGAVMQPTQKNEVA<br>IEFHQDSTLEDEDESL<br>VDIRFFIPNRSTEEGS<br>KTSAQILHKGLLDVIG<br>GGIQGKDRIATLNKV<br>PILTPRGRYDIELYPTF<br>MKFRGKTHDYKVTYE<br>SVSRLFQLPKPDQHHI<br>FFIVTLDPPIRQGTMR<br>YPHLVMHFKMDEKS<br>PPVPITLPADLKEKEQ<br>FKSLPEEFKDADAYEV<br>VNKLFRAFTQRKITTP<br>GSFKSHSGASAVKCA<br>FKANDGFLFLLERSFF<br>FVHKPPLHMRFEEVS<br>SVEFARVASSTNAQA<br>NRTFDLVIRLSDSTVH<br>QFTGIQRNEYGSLFN<br>FIQSKKLRIENPDEAT

NP_013642.1	ScPOB3	S. cerevisiae	SSRP1	SGRFRIADSGLGWKIS<br>TSGGSAANQARKPFL<br>LPATELSTVQWSRGC<br>RGYDLKINTKNQGVI<br>QLDGFSQDDYNLIKN<br>DFHRRFNIQVEQREH<br>SLRGWNWGKTDLAR<br>NEMVFALNGKPTFEI<br>PYARINNTNLTSKNE<br>VGIEFNIQDEEYQPA<br>GDELVEMRFYIPGVI<br>QTNVDENMTKKEESS<br>NEVVPKKEDGAEGED<br>VQMAVEEKSMAEAF<br>YEELKEKADIGEVAGD<br>AIVSFQDVFFTTPRGR<br>YDIDIYKNSIRLRGKTY<br>EYKLQHRQIQRIVSLP<br>KADDIHHLLVLAIEPP<br>LRQGQTTYPFLVLQF<br>QKDEETEVQLNLEDE<br>DYEENYKDKLKKQYD<br>AKTHIVLSHVLKGLTD<br>RRVIVPGEYKSKYDQ<br>CAVSCSFKANEGYLY<br>PLDNAFFFLTKPTLYIP<br>FSDVSMVNISRAGQT<br>STSSRTFDLEVVLRSN
XP_001014803.2	TtSSRP1	T. thermophi	SSRP1	VTFEDDQLVLSHNKR<br>RLCKLSLKKFTNSTVN<br>KTDIVIDLNTVDLQDD<br>EDQLCEMRLFIPQQQ<br>DAQMKPEDDGEGEE<br>KEGEDSVGGYADQL<br>NSEIITKAKIGQYSGQ<br>SIVKFEDISLLVPRGKY<br>QLDMYKKTVRFHGSS<br>FNYIVEYSNVIKGFLLP<br>QPDEVHVAFVLGLD<br>QPLKIGNTVHSYIVM<br>QFKKEQKANIKVNID<br>PEEKKEDKLKDLDEEY<br>DGFLYEIAGQLFKTLC<br>NNVQIIMPAGFQSSD<br>KQNCLKCTLKTHQGL<br>LYPMRKSLIFIYKPVIH<br>IQISDIQKVEFNRVGN<br>ATLNKLFDVKVFTKTT<br>TPQFFGFERKELDVLL<br>EYFKSKNIKITYDDTN<br>QGATFDDEDEEFTDSI<br>SEDEEEGKRAQRKSA<br>KRANKAVKGQSGLPL<br>DDDDEEEDEDFDADE<br>FESEDDEEEEEDEDED<br>EDFEQ
NP_001084164.1	XlSSRP1	X. laevis	SSRP1	GSMNDGRLRLSRAG<br>LMYKNNKTGKVENIS<br>AADIAEVVWRRVAL<br>GHGIKLLTNGGHVYK<br>YDGFRETEYDKLFDYF<br>KSHFSVELVEKDLCVK<br>GWNWGSVRFGGQL<br>LSFDIGDQPAFELPLS<br>NVSQCTTGKNEVTLE<br>FHQNDDSEVSLMEIR<br>FYVPPTQDDGGDSVE<br>AFAQNVLSKADVIQA<br>TGDAVCIFRELQCLTP<br>RGRYDIRIYPTFLHLH<br>GKTFDYKIPYTTVLRLF<br>LLPHKDQRQMFFVIS<br>LDPPIKQGQTRYHFLI<br>LLFSKDEDMTLTLNM<br>SEEEVERRFEGKLKKS<br>MSGCLYEMVSRVMK<br>ALVNRKITVPGNFLG<br>HSGSQCITCSYKASSG<br>LLYPLERGFIYVHKPP<br>VHIRFDEITCVNFARG<br>TTTTRSFDFEIETKQG<br>SQYTFSSIEREEYGKLF<br>DFVNAKKLSIKNRGLK<br>EGMKPAYDDYADSD

Q9LEF5	ZmSSRP1	Z. mays	SSRP1	RGGTNPGQFKVHSG<br>GLAWKRQGGGKTIEI<br>DKADVTAVTWMKVP<br>RAY<br>QLGVRIKAGLFYRFIG<br>FREQDVSNLTNFIQK<br>NMGVTPDEKQLSVS<br>GQNWGGIDIDGNM<br>LT<br>FMVGSKQAFEVSLPD<br>VAQTQMQGKTDVLL<br>ELHVDDTTGANEKDS<br>LMDLSFHVPTSNTQF<br>V<br>GDESRPPAHILWETIL<br>KFADVGSSEEPVVTFE<br>GIAILTPRGRYSVELHL<br>SFLRLQGQAND<br>FKIQYSSIVRLFLLPKS<br>NNPHTFVVITLDPPIR<br>KGQTLYPHIVIQFETE<br>AVVERDLALSK<br>ELLVEKYKDRLEESYK<br>GLIHEVFTKVLRGLSG<br>AKVTRPGSFRSCQDG<br>YAVKSSLKAEDGL<br>LYPLEKGFFFLPKPPTL<br>ILHEEIEFVEFERHGA
NP_015377.1	ScNHP6A	S. cerevisiae	NHP6	MVTPREPKKRTTRKK<br>KDPNAPKRALSAYMF<br>FANENRDIVRSENPDI<br>TFGQVGKKLGEKWK<br>ALTPEEKQPYEAKAQ<br>ADKKRYESEKELYNAT<br>LA
NP_009647.2	ScNHP6B	S. cerevisiae	NHP6	MAATKEAKQPKEPK<br>KRTTRRKKDPNAPKR<br>GLSAYMFFANENRDI<br>VRSENPDVTFGQVG<br>RILGERWKALTAEEK<br>QPYESKAQADKKRYE<br>SEKELYNATRA
-	PpNHP6A	P. polycephal	NHP6	MEGESQAPEDMVNE<br>EEVKDTGKKKGGKKA<br>VKQLVKPKAPAKEDK<br>KKVDAKEDKKKKDAK<br>DDKQKKKEEEAESEE<br>QESDKEEEQEEEDGA<br>ETKAKDKTTAKGKVG<br>AKKATTKESKPKKEKA<br>GKDKKDKKDKTEKKV<br>KDPNAPKRPQTAYLIF<br>ANEQRETIKKDNPSFS<br>PPDILKEAGARWKAL<br>TDKEKRKYQDKNEIEK<br>HRYKKEMEAYNANG<br>GEKDTDKKGAKRKKG<br>EEEEEEDEENAKPNK<br>KAAAADKGKGKPKG<br>LLLAKAGAKKK
-	PpNHP6B	P. polycephal	NHP6	MDTPKECPAKEDAP<br>AEKTEQPAPEKKEAE<br>KEEGKEGKDEEKDVK<br>VSESEEETLKQEPKRA<br>RSAYAIFTAEQVPMV<br>KKEHPDMSRTEITKCI<br>AAKWKAMTAEQKQ<br>PFEEQAVNEKKRIKEA<br>KEAAAKEAKDKEAEK<br>PAESEVRYSLRNSTVI<br>KDGEELVNTKKRALE<br>TEEDSANKKKCVTSEE
-	PpNHP6C	P. polycephal	NHP6	MTTLHECLKTLLADTE<br>NVTLDTEAEILLYNFV<br>SFISTRGTKGNKENTY<br>PTPISSETSSQNEDDS<br>DEEPTRKTKGTPIKRK<br>KDPNAPKRPLTAFML<br>YAHDKRAEIKAQNPD<br>MTFGALGKKLGETW<br>LTFDATQKKVYSDMA<br>DKAKLTYEKELAEYTK<br>NKQT
-	PpNHP6D	P. polycephal	NHP6	MSSDEESASSASEVEE<br>VEEKKKRGPKKGAKK<br>AEKTKKATKKVAKKK<br>KDPNAPKRPLSAFMY<br>YSQKQRPNVKKENPS<br>ATFADVARILGEQWK<br>ALDASLKKEYEKQAA<br>TDKARWEKEKAAYD<br>GGSSKGGKKKAAGS<br>DDEEEESGSDE

-	PpNHP6E	P. polycephal	NHP6	MVRFPSYFGPQDFEF<br>QKGKRARPYFKRKRL<br>LSRCPLFVFQTTRTHS<br>QHSQEHKMVAKKEK<br>KEAVEGERKSSRTSK<br>QVERLSVSPPTILKKK<br>ATKKSTTASKTKSPKK<br>AGAKKGTKKAKGEK<br>GPKRPASAYLLFANE<br>NRAKVKKANPDATF<br>GELGKLLGEAWSNAT<br>AADKKKFTALADKDK<br>ARYEKEKAALA
NP_001031485.1	AtNAP1	A thaliana	NAP family	MSPTSVRSREWEGPS<br>RWTEYLGPEMAASV<br>SSTRSSKQIDGHVGG<br>STKALNIQWVVQMIE<br>VADGLMAKMYRLN<br>QILEYPDPVGHVFSEA<br>FWKAGVFPNHPRICT<br>LLSKKFPEHFSKLQLE<br>RIDKFSLDSLHDGAEL<br>HLQSLEPWIQLLLDL<br>MAFREQALRLILDLSS<br>TVITLLPHQNSLILHAF<br>MDLFCAFVRVNLFAE<br>KIPRKMLLQVYNLLH<br>ALSRNDRDCDFYHRL<br>VQFIDSYDPPLKGLQE<br>DLNFVSPRIGEVLEAV<br>GPSIFLSADTRKLRNE<br>GFLSPYHPRFPDILTN<br>SAHPMRAQDLANVT<br>SYREWVLLGYLVCPD<br>ELLRVTSIDIALVVLKE<br>NLVVTLFRDEVSLYQ<br>MVCEKEFGIGISFASA<br>DSINLTMQYILLHEDY<br>QLYVLPRVLESKKMA<br>KSGRTKQKEADLEYS<br>VAKQVEKMISEVHEQ
NP_194341.1	AtNAP1;1	A thaliana	NAP family	MSNDKDSFNVSDLT<br>AALKDEDRAGLVNAL<br>KNKLQNLAGQRSDVL<br>ENLTPNVRKRVDALR<br>DIQSQHDELEAKFREE<br>RAILEAKYQTLYQPLY<br>VKRYEIVNGTTEVELA<br>PEDDTKVDQGEEKTA<br>EEKGVPSFWLTALKN<br>NDVISEEVTERDEGAL<br>KYLKDIKWCKIEEPKG<br>FKLEFFFDTNPYFKNT<br>VLTKSYHMIDEDEPLL<br>EKAMGTEIDWYPGK<br>CLTQKILKKKPKKGSK<br>NTKPITKLEDCESFFN<br>FFSPPEVPDEDEDIDE<br>ERAEDLQNLMEQDY<br>DIGSTIREKIIPRAVSW<br>FTGEAMEAEDFEIDD<br>DEEDDIDEDEDEEDEE<br>DEEDDDDEDEEESKT<br>KKKPSIGNKKGGRSQI<br>VGEGKQDERPPECK<br>QQ
NP_001319544.1	AtNAP1;4	A thaliana	NAP family	MSNEENIKSDNKSGD<br>SSDLPTIPALDIGAEEC<br>DLLAELKNLTLKRPFD<br>VKKLSPKVTKRVLFLK<br>DIQVTHDELEEKFLAE<br>KSALEATYDNLYKPLF<br>AKRYEIVNGVVEAEA<br>EKEGVPNFWLIAMKT<br>NEMLANEITERDEAA<br>LKYLKDIRSCRVEDTS<br>RNFKLEFLFDSNLYFK<br>NSVLSKTYHVNDEDG<br>PVLEKVIGTDIEWFPG<br>KCLTHKVVVKKKTKK<br>GPKKVNNIPMTKTEN<br>CESFFNFFKPPEIPEID<br>EVDDYDDFDTIMTEE<br>LQNLMDQDYDIAVTI<br>RDKLIPHAVSWFTGE<br>ALVDEDDSDDNDDD<br>DNDEKSD

NP_001077822.1	AtNRP1	A thaliana	NAP family	MVADKSKKSKIEEKG<br>EEENLEQIDAELVLSIE<br>KLQEIQDDLEKINEKA<br>SDEVLEVEQKYNVIRK<br>PVYDKRNEVIQSIPGF<br>WMTAFLSHPALGDLL<br>TEEDQKIFKYLNSLEV<br>EDAKDVKSGYSITFHF<br>TSNPFFEDAKLTKTFT<br>FLEEGTTKITATPIKW<br>KEGKGLPNGVNHDD<br>KKGNKRALPEESFFT<br>WFTDAQHKEDAGDE<br>IHDEVADIIKEDLWSN<br>PLTYFNNDADEEDFD<br>GDDDGDEEGEEDDD<br>DEEEEDGEE
NP_564063.1	AtNRP2	A thaliana	NAP family	MVTDKSKKAKTEEEN<br>VEQIDAELVLSIEKLQE<br>IQDDLEKINEKASDEV<br>LEVEQKYNVIRKPVYD<br>KRNEIIKTIPDFWLTA<br>FLSHPALGELLTEEDQ<br>KIFKYLSSLDVEDAKD<br>VKSGYSITFSFNPNPF<br>FEDGKLTKTFTFLEEG<br>TTKITATPIKWKEGKG<br>LANGVNHEKNGNKR<br>ALPEESFFTWFSDAQ<br>HKEDVEDEMQDEVA<br>DIIKEDLWPNPLTYFN<br>NDADEEDFDGDDDG<br>DEEEKEGDSDEDDDE<br>EDEVGEE
XP_006836211.1	AtrNRP2A	A. trichopoda	NAP family	MVEKGKKSKAQPVE<br>DDVSDHIDGELVLSIE<br>KLQEIQDELEKVNEEA<br>SDKVLEVEQKYNEIRR<br>PVYGRRNEIIQTIPDF<br>WLTAFLSHPALCDLLS<br>EEDQKIFKYLHSLDVE<br>DSKDIKSGYTITFNFN<br>TNPYFEDAKLIKTLTFS<br>DDGSTKVTGTTIKWK<br>EGMDVSNGINHGKE<br>GKKRPFSEGSFFTWF<br>SETDQQKDFTEGLQD<br>EVAEIIKEDLWPNPLK<br>YFNNEAEGDFDEDD<br>DDDENEDDEGESEDS<br>SDEEGDEDDS
XP_006827206.1	AtrNRP2B	A. trichopoda	NAP family	MVLSKGKKAKVSAPE<br>EEEGLEHIDGELVLSIE<br>RLQEIQDELEKVNEEA<br>SDKVLEVEQKYNEIRR<br>PVYNKRKEIIQTIPDF<br>WLTAFQSHPALGELL<br>TEEDEKVFKYLQSLDV<br>QDFKDVKSGYSIAFN<br>FSTNPYFENTKLTKTIS<br>FSDEGTSKITGTTIKW<br>KDGMEMPNGVDHK<br>KKGGKRAFTEESFFS<br>WFSDNRQNGGLSES<br>EDESIVCDEVAEIIKED<br>LWPNPLKYFNNDAD<br>EEEMDEEEEDEEEDD<br>DALGKNENGSDDDD<br>SDDDEQDGDDEDEA
XP_020520079.1	AtrNAP1;4-X4	A. trichopoda	NAP family	MLDLPIVYILFHKSLPR<br>WFMSEYDALEAKFFE<br>ERAALEAKYQKQYEP<br>LYTKRFEIVNGVVEVD<br>SVKNDTTEETAADDN<br>AKGVPDFWLTAMKT<br>NEVLAGEIYERDEGAL<br>KYLKDIKWCKIEDPKG<br>FKLEFFFDTNPFFKNS<br>VLTKTYHVEEDDEPLL<br>EKAIGTEIEWYPGKCL<br>TQKVLKKKPKKGVKN<br>AKPITKTEPCESFFNFF<br>KPPQVPEDDADIDED<br>TAEELQNLMEQDYDI<br>GTTIRDKIIPHAVSWF<br>TGEAIDGEEFEGETG<br>DEEDDDDDDEDGDD<br>DEDEEDDDVEDEDD<br>DDEDEEEEGKSKKSG<br>VVRLKVRLLLSFSLFPP<br>SRMQFTDIRFLFFGV<br>QKAGSRQNGDQGD<br>RPAECKQQ

XP_011620892.1	AtrNAP1;4	A. trichopoda	NAP family	MSKQNDQLSPYNM<br>ADLGSALPGAAALSA<br>EDRAGLVNALKNKLQ<br>HLAGQHSDVLESLST<br>KVKKRVVVLQEIQSQ<br>HDELEAKFFEERAALE<br>AKYQKLYDPLYTKRYE<br>IVNGIVEVDSVENEEA<br>SEKTPRDDTDNNEEK<br>GVPDFWLTAMKTNE<br>VLSGEIYERDEEALKYL<br>RDIKWCRIDNPKGFK<br>LEFFFDPNPYFKNSVL<br>TKTYHMIDDDEPILER<br>AIGTDIEWYPGKSLT<br>QKVLKKKPKKGSKNA<br>KPITKTEDCESFFNFFS<br>PPQVPEDDEDIDEET<br>AEQLQNLMEQDYDI<br>GSTIRDKIIPHAVSWF<br>TGEAVQGDEFEDMD<br>EEDDEDGDEDGDDD<br>EEEEEEDEDDEDDED<br>DEEEERGKSRKKPVA<br>AGHKKIAGGVVGEG<br>QQGERPPECKQQ
NP_501422.1	CeNAP1	C. elegans	NAP family	MADQEHIDAGLLSTN<br>FDMIQALPLNVKQRV<br>CALKNLQMKTIQIES<br>DFYKRVHELEIEFEGK<br>FKSTFDQRKAIVAGE<br>VEPTKEQIDTPILEGLE<br>GDQLAELYKAAEADP<br>SAKGIKDFWLTALRT<br>HDLVAEAIEEHDVPIL<br>SYLTDVTTAASKDPA<br>GFKIEFHFATNPYFKN<br>QVLTKTYLLGFDPDAE<br>APLQFDGPHVIRAVG<br>DTIEWEDGKNVTKKA<br>VKKKQKKGANAGKFL<br>TKTVKADSFFNFFEPP<br>KSKDERNEDEDDEQA<br>EEFLELDYEMGQAIR<br>DTIIPRAVLFYTGELQS<br>DDMFDFPGEDGDDV<br>SDFSDDEA
NP_001021332.1	CeSPR-2A	C. elegans	NAP family	MSEEPAAKRMKNAD<br>EFPNLRSEVAGPVKE<br>KLIELDAIQHQLDVM<br>SENAAEEVLKVEQIFN<br>QKRLPIYEKRKKLTTKI<br>DNFWQTAFLNHHLL<br>STAIPEEQEDLLAALR<br>DLEVQEFEDLRSGFKII<br>MTFDPNEYFTNEVIT<br>KSYHLQSESPSTEITEI<br>EWKENKKPPFPEDG<br>DSAHTFLEWLTYAAL<br>PDSDEIAEVIKDDLYV<br>NPLQYYVMPDMQEV<br>EEDDIEDFLNEERGVD<br>ENGQRIPRRNISDSLK<br>VDQDESADGQEGED<br>EEEEDMGEEEEDGVE<br>EEAEGEEEEEDGAIEE<br>EGGDENVEAHVAVN<br>PENTAE
NP_001021333.1	CeSPR-2B	C. elegans	NAP family	MSEEPAAKRMKNAD<br>EFPNLRSEVAGPVKE<br>KLIELDAIQHQLDVM<br>SENAAEEVLKVEQIFN<br>QKRLPIYEKRKKLTTKI<br>DNFWQTAFLNHHLL<br>STAIPEEQEDLLAALR<br>DLEVQEFEDLRSGFKII<br>MTFDPNEYFTNEVIT<br>KSYHLQSESPSTEITEI<br>EWKENKKPPFKPEDG<br>DSAHTFLEWLTYAAL<br>PDSDEIAEVIKDDLYV<br>NPLQYYVMPDMQEV<br>EEDDIEDFLNEERGVD<br>ENGQRIPRRNISDSLK<br>VDQDESADGQEGED<br>EEEEDMGEEEEDGVE<br>EEAEGEEEEEDGAIEE<br>EGGDENVEAHVAVN<br>PENTAE

NP_958876.1	DrSETB	D. rerio	NAP family	MSASAAKVSRKEQN SNHDGADETSEKEQ QEAIEHIDEVQNEIDR LNEQASEEILKVEQKY NKLRQPFFQKRSELIA KIPNFWVTTFVNHPQ VSALLGEEDEEALHYL TRVEVTEFEDIKSGYRI DFYFDENPYFENKVLS KEFHLNESGDPSSKST EIKWKAGKDLTKRTG QTPNKAGKKRQHEE PESFFTWFTDHSDAG ADELGEVIKDDIWPN PLQYYLVPDMDEEEG EGEEEDDEEEEEGLED IDEEGDEDDGEEDEE EDEGEDGEDDGEDD
NP_958883.1	DrSETA	D. rerio	NAP family	MSASAAKVSKKELNS NHDGGDETSEKEQQ EAIEHIDEVQNEIDRL NEQASEEILKVEQKY NKLRQPFFQKRSELIA KIPNFWVTTFVNHPQ VSALLGEEDEEALHYL TRVEVTEFEDIKSGYRI DFYFDENMYFENKVL SKEIHLNESGDPTSKS TEIKWKPGKDLTSRSS QTQSKAGKKRQHEE PESFFTWFTDHADSG ADELGEVIKDDIWPN PLQYYLVPDMEDEEG EGEDEDDDEEGLDDI DEEGDDDGEEEDDD DGEDDEGDDD
AAH52128.1	DrNAP1L1A	D. rerio	NAP family	HKALASLTGGDADD DCKEMPCSSKKASRE KSRDKSNRPGCSQSD RRTEAAAADSSSSLLL CEDQEDPSFVDDDEE EEEDEDSLPGSSSTAS SSVASDNEDNEDGEC AIVSVKMAPEVRQSV ALLAQVQMRLDALEK KGARLHQRLEMKLSR QRRPHLDQRSAITQA VPGFWVTALLNHPH LSAHIDETDEDALSY MTNLEVESFKNNKLG YRICFHFRRNPFFQN KMIVKELHLGMGGS PVSFSNPILWHRGQN LVGSGEPRRTSQGVY QSFFHWFSDHSNPG RDDIAQILREDLYRNP LRYYLTPLWEPRENG SSAPKPQGSSNRDEC VIISDSDEDEEQNSQS LGQEDDEDDDQGGV SEENERDEGDSSCEE RDEEEVDVEEVEVSH DSGGCEAREQEEEDI DVDEEKS
NP_956349.1	DrTSPY	D. rerio	NAP family	RKRSASPSGECESTQL KQAKVSVEEQKNEP QEDAEPEKVGNGEPK DGAEPSEGAKEKEAKI EDEVAAQESTGQAN VSGGESARQPSDSAA IAAAEALASLTGGDA DDDCKEMPCSSKKAS REKSRDKSNRPGCSQ SDRRTEAAAADSSSSL LLCEDQEDPSFVDDD EEEEEDEDSLPGSSST ASSSVASDNEDNEDG ECAIVSVKMAPEVRQ SVALLAQVQMRLDAL EKKGARLHQRLEMKL SRQRRPHLDQRSAIT QAVPGFWVTALLNH PHLSAHIDETDEDALS YMTNLEIESFKNNKL GYRICFHFRRNPFFQ NKMIVKELHLGMGG SPVSFSNPILWHRGQ NLVGSGEPRRTSQGV YQSFFHWFSDHSNP GRDDIAQILREDLYRN PLRYYLTPLWEPREN GSSAPKPQGSSNRDE

NP_001082816.1	DrNAP1L4a	D. rerio	NAP family	MDKKNEKGEARGKG<br>GTKPDKPEAFLQVER<br>VPYFNLLPKNVKRRV<br>YALKRLQLQSGNIEAK<br>FYEEVHELERKYAGLY<br>QPILDKRREIVTGAVE<br>PTDEECEWHSDKEED<br>ELADDLQKKAALEEK<br>QADSASAVDPKGIPD<br>FWLTIFKHVDMLGE<br>MLQEHDEPILKHLQD<br>VTVKFSEPGQPMSFT<br>LEFHFEPNSYFSNTVL<br>TKVYKMKSEPDAADP<br>FSFEGPEIVDCEGCKI<br>DWHKGKDVTVKIVK<br>KKQKHKGRGTIRTVS<br>KEIPQDSFFNFFNPVK<br>ASPDGMDEDLDFTLA<br>TDFEIGHFFRERVIPR<br>AVLYFTGEALEDDESF<br>EEEDLEEGEEEDLDEE<br>GEEEEEGDFDPTA
NP_001007454.1	DrNAP1L4b	D. rerio	NAP family	MEADNEAKGKVSDQ<br>AMQNPQALAALQDK<br>LDSVSHSASIMDSLPK<br>SVKRRVNALRNLQVD<br>STHIEAKFYKEVHELE<br>RKYSALYQPLFDKRRK<br>VVSGEVEPTDEECEW<br>QSDHEDEAALAEDLK<br>KKAAIEEKTEDANEEK<br>PKGIPEFWLTIFRSVD<br>MLSDMLQEHDEPILK<br>HLHDIKVIFSGPDQP<br>MSFTLEFHFEPNEYFT<br>NTVLTKVYKMKSEPD<br>ADDPFSFEGPEIIDCE<br>GCEIDWQKGKDVTV<br>KTIKKKQKHKGRGTA<br>RVITKQVPNDSFFNFF<br>NPIKVSPDKELDEDSE<br>YTLATDFEIGHFFRERI<br>VPRAVLYFTGEALED<br>DESFEEEELDEGEEED<br>QDDEEEEEEEEEGDS<br>KTGDPQPAECRQQ
XP_005170732.1	DrNAP1L4-X1	D. rerio	NAP family	MDKKNEKGEARGKG<br>GTKPDKPEAFLQVER<br>VPNFNLLPKNVKRRV<br>YALKRLQLQSGNIEAK<br>FYEEVHELERKYAGLY<br>QPILDKRREIVTGAVE<br>PTDEECEWHSDKEED<br>ELADDLQKKAALEEK<br>QADSASAVDPKGIPD<br>FWLTIFKHVDMLGE<br>MLQEHDEPILKHLQD<br>VTVKFSEPGQPMSFT<br>LEFHFEPNSYFSNTVL<br>TKVYKMKSEPDAADP<br>FSFEGPEIVDCEGCKI<br>DWHKGKDVTVKIVK<br>KKQKHKGRGTIRTVS<br>KEIPQDSFFNFFNPVK<br>ASPDGMDEDLDFTLA<br>TDFEIGHFFRERVIPR<br>AVLYFTGEALEDDESF<br>EEEDLEEGEEEDLDEE<br>GEEEEEGDFDPTKDP<br>PPAECKQQ

NP_001294853.1	HsNAP1L1	H. sapiens	NAP family	MADIDNKEQSELDQ<br>DLDDVEEVEEEETGEE<br>TKLKARQLTVQMMQ<br>NPQILAALQERLDGL<br>VETPTGYIESLPRVVK<br>RRVNALKNLQVKCA<br>QIEAKFYEEVHDLERK<br>YAVLYQPLFDKRFEII<br>NAIYEPTEEECEWKP<br>DEEDEISEELKEKAKIE<br>DEKKDEEKEDPKGIPE<br>FWLTVFKNVDLLSDM<br>VQEHDEPILKHLKDIK<br>VKFSDAGQPMSFVLE<br>FHFEPNEYFTNEVLTK<br>TYRMRSEPDDSDPFS<br>FDGPEIMGCTGCQID<br>WKKGKNVTLKTIKKK<br>QKHKGRGTVRTVTKT<br>VSNDSFFNFFAPPEV<br>PESGDLDDDAEAILA<br>ADFEIGHFLRERIIPRS<br>VLYFTGEAIEDDDDDY<br>DEEGEEADEEGEEEG<br>DEENDPDYDPKKDQ<br>NPAECKQQ
NP_068798.1	HsNAP1L2	H. sapiens	NAP family	EEAGNQIMVEGLGE<br>HLERGEDAAAGLGD<br>DGKCGEEAAAGLGEE<br>GENGEDTAAGSGED<br>GKKGGDTDEDSEAD<br>RPKGLIGYVLDTDFVE<br>SLPVKVKYRVLALKKL<br>QTRAANLESKFLREF<br>HDIERKFAEMYQPLL<br>EKRRQIINAIYEPTEEE<br>CEYKSDSEDCDDEEM<br>CHEEMYGNEEGMV<br>HEYVDEDDGYEDYYY<br>DYAVEEEEEEEEEDDI<br>EATGEENKEEEDPKGI<br>PDFWLTVLKNVDTLT<br>PLIKKYDEPILKLLTDIK<br>VKLSDPGEPLSFTLEF<br>HFKPNEYFKNELLTKT<br>YVLKSKLAYYDPHPYR<br>GTAIEYSTGCEIDWN<br>EGKNVTLKTIKKKQK<br>HRIWGTIRTVTEDFPK<br>DSFFNFFSPHGITSNG<br>RDGNDDFLLGHNLRT<br>YIIPRSVLFFSGDALES<br>QQEGVVREVNDAIY<br>DKIIYDNWMAAIEEV
NP_004529.2	HsNAP1L3	H. sapiens	NAP family	HGVAEEEMASSTSDS<br>GEESDSSSSSSSTSDSS<br>SSSSTSGSSSGSGSSSS<br>SSGSTSSRSRLYRKKR<br>VPEPSRRARRAPLGT<br>NFVDRLPQAVRNRV<br>QALRNIQDECDKVDT<br>LFLKAIHDLERKYAEL<br>NKPLYDRRFQIINAEY<br>EPTEEECEWNSEDEE<br>FSSDEEVQDNTPSEM<br>PPLEGEEEENPKENPE<br>VKAEEKEVPKEIPEVK<br>DEEKEVPKEIPEVKAE<br>EKADSKDCMEATPEV<br>KEDPKEVPQVKADDK<br>EQPKATEAKARAAVR<br>ETHKRVPEERLQDSV<br>DLKRARKGKPKREDP<br>KGIPDYWLIVLKNVD<br>KLGPMIQKYDEPILKF<br>LSDVSLKFSKPGQPVS<br>YTFEFHFLPNPYFRNE<br>VLVKTYIIKAKPDHND<br>PFFSWGWEIEDCKG<br>CKIDWRRGKDVTVTT<br>TQSRTTATGEIEIQPR<br>VVPNASFFNFFSPPEI

NP_005960.1	HsNAP1L4	H. sapiens	NAP family	MADHSFSDGVPSDSVEAAKNASNTEKLTDQVMQNPRVLAALQERLDNVPHTPSSYIETLPKAVKRRINALKQLQVRCAHIEAKFYEEVHDLERKYAALYQPLFDKRREFITGDVEPTDAESEWHSENEEEEKLAGDMKSKVVVTEKAAATAEEPDPKGIPEFWFTIFRNVDMLSELVQEYDEPILKHLQDIKVKFSDPGQPMSFVLEFHFEPNDYFTNSVLTKTYKMKSEPDKADPFSFEGPEIVDCDGCTIDWKKGKNVTVKTIKKKQKHKGRGTVRTITKQVPNESFFNFFNPLKASGDGESLDEDSEFTLASDFEIGHFFRERIVPRAVLYFTGEAIEDDDNFEEGEEGEEEELEGDEEGEDEDDAEINPKV
NP_715638.1	HsNAP1L5	H. sapiens	NAP family	MADSENQGPAEPSQAAAAAEAAAEEVMAEGGAQGGDCDSAAGDPDSAAGQMAEEPQTPAENAPKPKNDFIESLPNSVKCRVLALKKLQKRCDKIEAKFDKEFQALEKKYNDIYKPLLAKIQELTGEMEGCAWTLEGEEEEEEEYEDDEEEGEDEEEEEAAAEAAAGAKHDDAHAEMPDDAKK
A6NFF2	HsNAP1L6	H. sapiens	NAP family	MMEGLGEHSTAGEMGPLLGAVAATASPQSLMEYSSDADFIESLPLVVKYRVYTLKKLQAKCAVLEAKYLREFHSVERKFATIYGPLLEKRRQITNALYEPTKEECER
NP_001071165.2	HsTSPY1	H. sapiens	NAP family	MRPEGSLTYRVPERLRQGFCGVGRAAQALVCASAKEGTAFRMEAVQEGAAGVESEQAALGEEAVLLLDDIMAEVEVVAEEEGLVERREEAQRAQQAVPGPGPMTPESAPEELLAVQVELEPVNAQARKAFSRQREKMERRRKPHLDRRGAVIQSVPGFWANVIANHPQMSALITDEDEDMLSYMVSLEVGEEKHPVHLCKIMLFFRSNPYFQNKVITKEYLVNITEYRASHSTPIEWYPDYEVEAYRRRHHNSSLNFFNWFSDHNFAGSNKIAEILCKDLWRNPLQYYKRMKPPEEGTETSGDSQLLS
NP_003300.1	HsTSPYL1	H. sapiens	NAP family	HSIIISDQVPSDQDAHQYLRLRDQSEATQVMAEPGEGGSETVALPPPPPSEEGGVPQDAAGRGGTPQIRVVGGRGHVAIKAGQEEGQPPAEGLAAASVVMAADRSLKKGVQGGEKALEICGAQRSASELTAGAEAEAEEVKTGKCATVSAAVAERESAEVVKEGLAEKEVMEEQMEVEEQPPEGEEIEVAEEDRLEEEAREEEGPWPLHEALRMDPLEAIQLELDTVNAQADRAFQQLEHKFGRMRRHYLERRNYIIQNIPGFWMTAFRNHPQLSAMIRGQDAEMLRYITNLEVKELRHPRTGCKFKFFFRRNPYFRNKLIVKEYEVRSSGRVVSLSTPIIWRRGHEPQSFIRRNQDLICSFFTWFSDHSLPESDKIAEIIKEDLWPNPLQYYLLREGVRRARRRPLREPVEIPRPFGFQSG

NP_071400.1	HsTSPYL2	*H. sapiens*	NAP family	LSSSESPQRDPPPPPP<br>PPPLLRLPLPPPQQRP<br>RLQEETEAAQVLAD<br>MRGVGLGPALPPPP<br>PYVILEEGGIRAYFTLG<br>AECPGWDSTIESGYG<br>EAPPPTESLEALPTPE<br>ASGGSLEIDFQVVQS<br>SSFGGEGALETCSAV<br>GWAPQRLVDPKSKE<br>EAIIIVEDEDEDERES<br>MRSSRRRRRRRRRK<br>QRKVKRESRERNAER<br>MESILQALEDIQLDLE<br>AVNIKAGKAFLRLKRK<br>FIQMRRPFLERRDLII<br>QHIPGFWVKAFLNH<br>PRISILINRRDEDIFRYL<br>TNLQVQDLRHISMGY<br>KMKLYFQTNPYFTN<br>MVIVKEFQRNRSGRL<br>VSHSTPIRWHRGQEP<br>QARRHGNQDASHSF<br>FSWFSNHSLPEADRI<br>AEIIKNDLWVNPLRYY<br>LRERGSRIKRKKQEM<br>KKRKTRGRCEVVIME<br>DAPDYYAVEDIFSEIS
NP_056596.1	MmNAP1L1	*M. musculus*	NAP family	MADIDNKEQSELDQ<br>DLEDVEEVEEEETGEE<br>TKIKARQLTVQMMQ<br>NPQILAALQERLDGL<br>VDTPTGYIESLPKVVK<br>RRVNALKNLQVKCA<br>QIEAKFYEEVHDLERK<br>YAVLYQPLFDKRFEII<br>NAIYEPTEEECEWKP<br>DEEDEVSEELKEKAKI<br>EDEKKDEEKEDPKGIP<br>EFWLTVFKNVDLLSD<br>MVQEHDEPILKHLKD<br>IKVKFSDAGQPMSFV<br>LEFHFEPNDYFTNEVL<br>TKTYRMRSEPDDSDP<br>FSFDGPEIMGCTGCQ<br>IDWKKGKNVTLKTIK<br>KKQKHKGRGTVRTVT<br>KTVSNDSFFNFFAPPE<br>VPENGDLDDDAEAIL<br>AADFEIGHFLRERIIPR<br>SVLYFTGEAIEDDDDD<br>YDEEGEEADEEGEEE<br>GDEENDPDYDPKKD<br>QNPAECKQQ
NP_032697.2	MmNAP1L2	*M. musculus*	NAP family	EELGSQVMAEGPGES<br>QDRSEGVSIEPGDGG<br>QHGEETVAAGVGEE<br>GKGEEAAAGSGEDA<br>GKCGGTDEDSDSDRP<br>KGLIGYLLDTDFVESLP<br>VKVKCRVLALKKLQT<br>RAAHLESKFLREFHDI<br>ERKFAEMYQPLLEKR<br>RQIINAVYEPTEEECE<br>YKSDCEDYFEEEMDE<br>EEETNGNEDGMVHE<br>YVDEDDGYEDCYYDY<br>DDEEEEEEEDDSAGA<br>TGGEEVNEEDPKGIP<br>DFWLTVLKNVEALTP<br>MIKKYDEPILKLLTDIK<br>VKLSDPGEPLSFTLEF<br>HFKPNEYFKNELLTKT<br>YVLKSKLACYDPHPYR<br>GTAIEYATGCDIDWN<br>EGKNVTLRTIKKKQR<br>HRVWGTVRTVTEDF<br>PKDSFFNFFSPHGISL<br>NGGDENDDFLLGHN<br>LRTYIIPRSVLFFSGDA<br>LESQQEGVVREVNDE<br>IYDKIIYDDWMAAIEE

NP_620081.1	MmNAP1L3	M. musculus	NAP family	HGVAEEAMASTACD<br>SGDESDSNSSSSTNSC<br>SSSGSSSSGSSSSSSSS<br>SSSSSSSSSSSSGSSGS<br>SSNGSHLNRKKRVPE<br>PSRRAQRRPSGKLFL<br>DKLPQAVRNRVQAL<br>RNIQNECDKVDTLFL<br>RAIHDLERKYAELNKP<br>LYDKRFQIINAEYEPT<br>EEECEWNSEEEFSGD<br>EEMQDDTPNEMPPL<br>EGEEEEESCNEKAEVK<br>EEGTHVPEEVPEAKV<br>EEEEAPKETPEVKTEE<br>KDIPKEGAEEKAEEQE<br>SSKEIPEVKGEEKADS<br>TDCIDIAPEEKEDVKE<br>VTQANTENKDQPTEE<br>FTPRAPAREAQKRVP<br>ETRPEEGVNIKRARK<br>GKPKKEDPKGIPDYW<br>LTVLKNVDKLGPMIQ<br>KCDEPILKFLSDVSLKF<br>SNPGQPIGYTFEFHFL<br>PNPYFRNELLMKTYII<br>RSKPDHYDPFFAWG<br>WEIEECKGCKIDWRR
NP_001272418.1	MmNAP1L4	M. musculus	NAP family	MAENSLSDGGPADS<br>VEAAKNASNTEKLTD<br>QVMQNPQVLAALQE<br>RLDNVSHTPSSYIETL<br>PKAVKRRINALKQLQ<br>VRCAHIEAKFYEEVH<br>DLERKYAALYQPLFDK<br>RREFITGDVEPTDAES<br>AWHSENEEEDKLAG<br>DMKNKVVIAEKEAAT<br>VEELNPKGIPEFWFTI<br>FRNVDMLSELVQEYD<br>EPILKHLQDIKVKFSD<br>PGQPMSFVLEFHFEP<br>NDYFTNPVLTKTYKM<br>KSEPDKADPFSFEGPE<br>IVDCDGCTIDWKKGK<br>NVTVKTIKKKQKHKG<br>RGTVRTITKQVPNESF<br>FNFFSPLKASGDGESL<br>DEDSEFTLASDFEIGH<br>FFRERIVPRAVLYFTG<br>EAIEDDDNFEEGEEG<br>EEEELEGDEEGEDED<br>DADVNPKV
NP_067407.1	MmNAP1L5	M. musculus	NAP family	MADPEKQGPAESRA<br>EDEVMEGAQGGEDA<br>ATGDSAAAPAAEEP<br>QAPAENAPKPKKDF<br>MESLPNSVKCRVLAL<br>KKLQKRCDKIEAKFDK<br>EFQALEKKYNDIYKPL<br>LAKIQELTGEMEGCA<br>WTLEGEDDEDDEEED<br>DEEEEEEEEAAAGAT<br>GGPNFAKK
NP_033459.1	MmTSYL1	M. musculus	NAP family	MSSPERDEGTPVPDS<br>RGHCDADTVSGTPD<br>RRPLLGEEKAVTGEG<br>RAGIVGSPAPRDVEG<br>LVPQIRVAAARQGES<br>PPSVRGPAAAVFVTP<br>KYVEKAQETRGAESQ<br>ARDVKTEPGTVAAAA<br>EKSEVATPGSEEVME<br>VEQKPAGEEMEMLE<br>ASGGVREAPEEAGP<br>WHLGIDLRRNPLEAI<br>QLELDTVNAQADRAF<br>QHLEQKFGRMRRHY<br>LERRNYIIQNIPGFW<br>MTAFRNHPQLSAMI<br>RGRDAEMLRYVTSLE<br>VKELRHPKTGCKFKFF<br>FRRNPYFRNKLIVKEY<br>EVRSSGRVVSLSTPII<br>WRRGHEPQSFIRRN<br>QDLICSFFTWFSDHSL<br>PESDRIAEIIKEDLWP<br>NPLQYYLCREGIRRPR<br>RRPIREPVEIPRPFGF<br>QSG

NP_084112.1	MmCINAP/M	M. musculus	NAP family	SSSEPRQRDLPPPPPPPLQRLPLPPPQQRPRPQEETEAAQVLADMRGVGPTLPPPLPYVILEEGGIRAYFTLSAESPGWDHAMESGFGEAPSTGIMETLPSSEISGGSLAIDFQVAEPSSLGEKALETCSLGGWGPQMLVGPKRKEEAIIIVEDEDEDDKESVRRRQRRRRRRRKQRKAKESRERSAQRMESILQALESIQMDLEAVNIKAGKAFLRLKRKFIQMRRPFLERRDLIIQHIPGFWVKAFLNHPRISILINQRDRDIFRYLTNLQVQDLRHISMGYKMKLYFQTNPYFTNMVIVKEFQRNRSGRLVSHSTPIRWHRGQEPQAYNRRSHDTRESFFNWFSNHSLPEADRIAEIIKNDLWVNPVRYYMRRGGYRSSRKKQHGKERAKNQYEMVIMEDAHDHYAIEDILSDISEIDEITDNET
NP_941019.2	MmTSPYL3	M. musculus	NAP family	MAWEGIGVLEATMCLPPIQERDAHPARPRVPGAEPLRPCNPAASPATAPPSSENTGENSASRVLNLDGCEKGSGNGVGTKGKTEEVTTEEDSVAAEEPAEVGEKLEWVAEAQESLRPLDLRALIVDPLEAIQWELEAMSAQADGAHLQLVRRFGRMRRLHLARRSFIIQNIPGFWVTAFLNHPQLSAMISPRDEDMLGYLMNLEVRELRHSRTGCKFKFLFESNPYFRNEVIVKEYECRASGGVVSIATRILWHRGQEPPALVHRNRDAVRSFFSWFSQHSLLEADRVAQIIKDDLWPNPLQYYLLGQRPYRARRSLARWSAEARPRPYGFQSG
NP_084479.1	MmTSYL4	M. musculus	NAP family	MNGVEGNNELSLANTTTPSHASEDLDLKQDQGLQEETDTVREMEAAGEAGADGGASPDSEHCGPELCFRVAENSCAAAARGLEDAPSPSKGGDAPSAPVAADDSSKNGCQLEGPHSPAKPKALEACGAVGLGSQQMPGPKKTKEMTTTKCAISVATGKEGEAGAAMQEKKGLQKEKKVAGGGKEETRPRAPKINCMDSLEAIDQELSNVNAQADRAFLQLERKFGRMRRLHMQRRSFIIQNIPGFWVTAFRNHPQLSPMISGQDEDMMRYMINLEVEELKQPRVGCKFKFIFQSNPYFRNEGLVKEYERRSSGRVVSLSTPIRWHRGQEPQAHIHRNREGNTIPSFFNWFSDHSLLEFDRIAEIIKGELWSNPLQYYLMGDGPRRGVRVPPRQPVESPRSFRFQSG

NP_001078890.1	MmTSPYL5	M. musculus	NAP family	MSGRSRGRKSSRAK<br>GRGKGRARARVRAA<br>AEDAWHDEKPPQSP<br>RLGEDSAAAQVQAG<br>AAQGGAEPAELREEA<br>ACRLPLDCGLALRAR<br>AADERGLAAPDPDLE<br>RARSLAERLTSDTSFV<br>GTVGALAKLRRGSRI<br>GNRRVPGRKAPDTRS<br>ATGRGPQATVSGKPK<br>MASAGLCAAAPVGE<br>EKKMTEKHAGAGSP<br>ATVGSMDTLETVQLK<br>LETMNAQADRAYLRL<br>SRKFGQLRLHHLERR<br>NLLIQSIPGFWGQAF<br>QNHPQLSAFLNTKDK<br>EVLSYLNRLEVEELGL<br>ARLGYKIKFYFGRNPY<br>FQNKVLIKEYGCGPS<br>GQVVSRSAPIQWLP<br>GHDLQSLSKENPENN<br>GSFFGWFSNHSSIES<br>DKIVEIINEDLWPNPL<br>QYYLISEEARGEKGKE<br>ERPGPAKLSPAPAVR<br>QPN
NP_012974.1	ScNAP1	S. cerevisiae	NAP family	MSDPIRTKPKSSMQI<br>DNAPTPHNTPASVLN<br>PSYLKNGNPVRAQA<br>QEQDDKIGTINEEDIL<br>ANQPLLLQSIQDRLG<br>SLVGQDSGYVGGLPK<br>NVKEKLLSLKTLQSELF<br>EVEKEFQVEMFELEN<br>KFLQKYKPIWEQRSRI<br>ISGQEQPKPEQIAKG<br>QEIVESLNETELLVDE<br>EEKAQNDSEEEQVKG<br>IPSFWLTALENLPIVC<br>DTITDRDAEVLEYLQD<br>IGLEYLTDGRPGFKLLF<br>RFDSSANPFFTNDILC<br>KTYFYQKELGYSGDFI<br>YDHAEGCEISWKDN<br>AHNVTVDLEMRKQR<br>NKTTKQVRTIEKITPIE<br>SFFNFFDPPKIQNED<br>QDEELEEDLEERLALD<br>YSIGEQLKDKLIPRAV<br>DWFTGAALEFEFEED<br>EEEADEDEDEEEDDD<br>HGLEDDDGESAEEQ<br>DDFAGRPEQAPECK<br>QS
NP_014153.1	ScVPS75	S. cerevisiae	NAP family	MMSDQENENEHAK<br>AFLGLAKCEEEVDAIE<br>REVELYRLNKMKPVY<br>EKRDAYIDEIAEFWKI<br>VLSQHVSFANYIRAS<br>DFKYIDTIDKIKVEWL<br>ALESEMYDTRDFSITF<br>HFHGIEGDFKEQQVT<br>KVFQIKKGKDDQEDG<br>ILTSEPVPIEWPQSYD<br>SINPDLIKDKRSPEGK<br>KKYRQGMKTIFGWF<br>RWTGLKPGKEFPHG<br>DSLASLFSEEIYPFCVK<br>YYAEAQRDLEDEEGE<br>SGLSADGDSEDDDGS<br>LGEVDLPLSDEEPSSK<br>KRKV

-	PpNAP1L1	P. polycephal	NAP family	MGKGSTFGGPPSAN<br>AAAEHDEEELDLAEL<br>QDALGSPLHELPPVV<br>RRRVDALSNLHKQYL<br>TLQKQYHAEVRELER<br>KYQKLYDPVFDKRAN<br>IVSGSYEPTDEEAKVE<br>KAEEEEGVKKREAQP<br>GDADIKGVPEFWLTV<br>LQNNGFVAQSIFEED<br>EEALSFLRDIRYSELEG<br>ENEGFTLTFHFAENPF<br>FSNTTLSKTYHMEQD<br>ELMGELQYDHATGTK<br>IEWKAGKNLTVKQTK<br>KKTKAKGKKPARVVT<br>VEEPCDSFFSFFSPVQ<br>LPASEEEELSEEDAQN<br>MEMDFEIGCIIKDKLI<br>PFSVLWFTGEAAEYE<br>DDDDDMFGDEEGD<br>DDEEDDEEDDDEEEE<br>EEAPKRGGKRQGGG<br>PGHSFAPPPGAKPGA<br>GAPGQPQQPECKQQ
-	PpSET	P. polycephal	NAP family	MAAPKTKRSKTEPED<br>TVDDEDIQVLEGIQD<br>QIEELNDKATEEILEVE<br>RKYNRLRKPMYDKRA<br>EAIRKLPEFWFKVFTS<br>QDMLAALLVGEDLA<br>AFKYMKELNVEDHD<br>DVKSGFKISLKFDPNP<br>FFKNELLSKEFRYDKE<br>GNLTVIPTKIEWKDG<br>KDLTKKSQSKDTKGK<br>RSRDEESESFFTWFN<br>PDDQDLELAEIIKEEL<br>WPNPAKYYLGLVEDE<br>AEEEGDDDDEGEDD<br>GEDGEGEGEGEGEAE<br>EDQ
NP_001116293.1	HsSET	H. sapiens	NAP family	MAPKRQSPLPPQKK<br>KPRPPPALGPEETSAS<br>AGLPKKGEKEQQEAI<br>EHIDEVQNEIDRLNE<br>QASEEILKVEQKYNKL<br>RQPFFQKRSELIAKIP<br>NFWVTTFVNHPQVS<br>ALLGEEDEEALHYLTR<br>VEVTEFEDIKSGYRIDF<br>YFDENPYFENKVLSKE<br>FHLNESGDPSSKSTEI<br>KWKSGKDLTKRSSQT<br>QNKASRKRQHEEPES<br>FFTWFTDHSDAGAD<br>ELGEVIKDDIWPNPL<br>QYYLVPDMDDEEGE<br>GEEDDDDDEEEEGLE<br>DIDEEGDEDEGEEDE<br>DDDEGEEGEEDEGED<br>D
XP_645850.1	DdNAP1	D. discoideur	NAP family	MSENNEIEMELPFDS<br>SAITSTEVLDRVNALL<br>TLQDTQNELTEQMEK<br>EILEIEKKYLKKFQPLA<br>EKRFEIVSGKVEPTKE<br>DQQCKAPIQVENLKS<br>VPTDKGIPKFWLHVL<br>QNTEVKDIIEECDIEAL<br>EYLVDIKIVQVGDAQ<br>DYSLDFHFSENPFFTN<br>TVISKTVKLEEDNELN<br>EIVSTPINWKDGKNF<br>TVQSKKKTVKSKPTK<br>GKAATTTSTTVQEVV<br>PCFFSTFVSPNQDPTS<br>DEEADEIMYIQYQIIA<br>KLKDIVIPEAVNFFLG<br>RASDAEENDYDFGED<br>FEDEEGEDDDEEDDE<br>EEQTIKKPSGKGKAQ<br>PQQPQDCKQQ

XP_642786.1	DdNAP2	D. discoideur	NAP family	MSKHQKKEVEVEFEK<br>DDPKAVYLELFDKDTE<br>LKSLSDEIEKLTGEKDL<br>ELMKCDKDIYKSAEPL<br>YRERRALLLEIPDFWA<br>TIFTNLFLKLGFVDELT<br>MCVDDFFVEDTETEF<br>RLFIDFRENDIISNKQ<br>VIITVTTPTTSEELSQE<br>VKISTTPIELKQNKTET<br>EKKNKDSKKSTDDED<br>EDEDDDEISGFLKFLL<br>NPTKDIATFIVGSVW<br>SNPIDSFCNDDDEDY<br>DGLIASDSEGEEDK
NP_650438.2	DmSET	D. melanogas	NAP family	MSSVPKRAKLDGAP<br>ADGNTSAAAGNNEE<br>ESEALEQIDACQNEID<br>ALNEKASEEILKVEQK<br>YNKLRKPCYEKRSELV<br>KRIPNFWVTSFINHP<br>QVSGILDEEEEECLHA<br>LNKLEVEEFEDIKSGY<br>RINFHFDENPYFENK<br>VLTKEFHLNSAAASE<br>NGDWPASTSTPIKW<br>KEGKNLLKLLLTKPYG<br>NKKKRNSEYKTFFDW<br>FSDNTDPVNDEIAELI<br>KDDLWPNPLQYYLVP<br>DIEVEPEDEEDNEDN<br>DEEAFDDEDGEDGE<br>GEEEEEDEDDK
NP_001246485.1	DmNAP1	D. melanogas	NAP family	MDAPAEGHVDPESC<br>NEIEDEKSGSDCQSM<br>PAYMNSVMRRQYLQ<br>QMVKMLPAPVQNRI<br>VFLKNLQLQHLNIEA<br>QFFEDVYKLEQKYQV<br>QYQPLFDKRREIIEGK<br>VDPAEEKPQWKEPES<br>STDNEADAEHFREAL<br>SSLKSIPKDAKGIPGF<br>WLTVFRNTAIMSEM<br>VQPHDEPAIRKLIDISI<br>KYDNGHSYTLEFHFD<br>KNEYFSNSVLTKQYVL<br>KSTVDPNDPFAFEGP<br>EIYKCTGCTINWEKK<br>MNLTVKTIRKKQKHK<br>ERGAVRTIVKQVPTD<br>SFFNFFSPPEVPSDQE<br>EVDDDSQQILATDFEI<br>GHFLRARIIPKAVLYY<br>TGDIVDDEDDEDEEE<br>YDENEEDEYDDDDAP<br>PPKGPKSAGIKKQSP<br>NDCPNQ
NP_058661.1	MmNCKP1	M. musculus	NAP family	KLTILNDRGVGMLTR<br>LYNIKKACGDPKAKPS<br>YLIDKNLESAVKFIVRK<br>FPAVETRNNNQQLA<br>QLQKEKSEILKNLALY<br>YFTFVDVMEFKDHVC<br>DLLNTIDVCQVFFDIT<br>VNFDLTKNYLDLTVTY<br>TTLMILLSRIEERKAIIG<br>LYNYAHEMTHGASD<br>REYPRLGQMIVDYEN<br>PLKKMMEEFVPHSKS<br>LSDALISLQMVYPRR<br>NLSADQWRNAQLLS<br>LISAPSTMLNPAQSD<br>TMPCEYLSLDAMEK<br>WIIFGFILCHGMLNTE<br>ATALNLWKLALQSSS<br>CLSLFRDEVFHIHKAA<br>EDLFVNIRGYNKRIND<br>IRECKEAAVSHAGSM<br>HRERRKFLRSALKELA<br>TVLSDQPGLLGPKALF<br>VFMALSFARDEIIWLL<br>RHADNMPKKSADDF<br>IDKHIAELIFYMEELRA<br>HVRKYGPVMQRYYV<br>QYLSGFDAVVLNELV

XP_024361543.1	PpaNAP1	P. patens	NAP family	LKRNNDASPIRRGPT<br>EEAWNADDTGTRST<br>DSPMWRRVSMTSN<br>RSQPNGKNVQKTRQ<br>IEWVIHLQNVAQGL<br>MTKLHKLHQILGPPE<br>PGSNMQYPESFWKT<br>GIIPDMPKLCQHVAR<br>KFPEHPAKIQLDKVD<br>KAGVDLLHEHAGRYV<br>ATLEPWIMVLEDLMT<br>FREQSLRVILDLSSTVV<br>TLMPNQNPLLLQVF<br>MDLFCCFVRVNLLAD<br>KVPRKMLLQMHNLV<br>HTIMKSGRDYGAYHR<br>MVQFVEAYDPPLKGL<br>HDDLNFVSPRIGEILD<br>AVGPTVLLGSDFQRL<br>RSEGYLSPFHPRYPEK<br>LTNSAHPARAQDLV<br>NIEAYREWALIGYLVC<br>PTELLRPGAVDIALVL<br>LKESLLLPLFRDEYVLL<br>HEEYQQYVLPRIAESK<br>KLAKAGRAKGGDAD<br>VEYNLAKQVEKQICD<br>AQDTAINNADVAHR
XP_001022132.2	TtNAP1L1	T. thermophi	NAP family	MSSETAAKHDHQNC<br>EDHDHDHEEIDIDQQ<br>IEESIKELALGEKIRAV<br>AINHHLAEKKALDEEL<br>QKKIQALTFEYEQKS<br>MPIYAKTQEIVQGRV<br>PTEEELKDLEKYLKEEE<br>KTQVEGAKKEEPITEY<br>WLKAMKSNDVLAM<br>EIKEQDETALKTLTKIE<br>YVLEDTKKFHIVFTFG<br>PNDYFTNTELKKTVEL<br>DENEEPVKTTGTPIE<br>WKEGKNTTVKITKKT<br>QKNKKTGVKRVVERE<br>TKIESFFNFFSDSAPT<br>DAPEEEDEEKDEQA<br>MDGDRINIDFDIARSL<br>IDEVIPYSLEYFLGIKT<br>GGEDHDEEIDMDDL<br>DEEEIAQLQKEYAAKT<br>GKKGPAAGGANDKK<br>DCKQQ
XP_001024488.1	TtNAP1L2	T. thermophi	NAP family	MRSKKNIVEEDYISFH<br>KDLSNKMSEKILHFEP<br>KFRIKLCAMLYHHKQ<br>KQNLDKDLHNELLKL<br>KRNYEKKTYEIIKQM<br>NSLIKEEGTIILNDFSDI<br>SDHFTMPGDPNYLEP<br>LAYPKIQKYWSSCIQ<br>NCTILQHQFTKKDQD<br>IAQCLETIEISREETKTT<br>KSFTLSFQFSQDQKYF<br>TNSFVQVTFYLIDSVN<br>AYRQESNAEFKIKTG<br>NKETATTGIFLQLFQN<br>VNLEEGLNKISDAHD<br>LCTKLGRSYEIALIFKD<br>ELIPYSLEYYLNARTPN<br>LKQEMLEKQLMEQY<br>QEKKKIKSKFVQQQQ<br>EEADEELADEQYLED<br>NQCKQQ

XP_012655909.1	TtNAP1L3	T. thermophi	NAP family	MDEELKAVITIKDEYQ<br>LVKQKMEQQIQQKL<br>QNEGYYQLIHLYQLK<br>DDLEQENKIYQSKVK<br>EILEKYKTLGQQIFQS<br>VNQIFLKNKNYFKFIN<br>AIQQQDIISGRRKEFQ<br>FSKEETADLDRYFNIT<br>NHNDQRAKNERFAS<br>HEVLPISNYWFMAFK<br>NFDLVKPYVKEKDDQ<br>ILKHLHNIFVNIGDKS<br>SIELKFEFKENEFFQN<br>QYLVKVFFLNDNMEP<br>ERSEGTQIEWYEGKD<br>VCRKTVKKSQKNRKT<br>GAIRVITHEVEDESFF<br>NFFNSLNLEEIKLMG<br>QKKENSLIQQNKYID<br>RMNIDYDIARAIIDEL<br>LPYSLEYYLDVRRLKSK<br>QAEVQDLNFLKEINE<br>SVSDSSDSDGGDNQ<br>KNDNSPTKQSQNNK<br>GKKDIKDTSKDKSGV<br>QDNTNECKQY
XP_012655909.1	XlNCKP1	X. laevis	NAP family	KLTILNDRGVGMLTR<br>LYNIKKACGDPKAKPS<br>YLIDKNLESAVKFIVRK<br>FPAVETRNNNQQLA<br>QLQKEKSEILKNLALY<br>YFTFVDVMEFKDHVC<br>ELLNTIDVCQVFLDIT<br>VNFDLTKNYLDLIVTY<br>TTLMIMLSRIEERKAII<br>GLYNYSHEMTHGAS<br>DREYPRLGQMIVDYE<br>NPLKKMMEEFVPHS<br>KSLSDALISLQMVYPR<br>RNLSAEQWRNAQLL<br>SLISAPSTMLNPAQS<br>DTMPCEYLSLDTMEK<br>WIILGFILCHGILNTD<br>ATSLNLWKLALQSSS<br>CICLYRDEVLHFHKAA<br>EDLFVNIRGYNKRIND<br>IRECKENAVSHAGAT<br>HRERRKFLRSALKELA<br>TVLSDQPGLLGPKALF<br>VFMALSFARDEIIWLL<br>RHADNIPKKIADDFM<br>DKHIAELIFYMEELRA<br>HVRKYGPVMQRYYV<br>QYLSGFDAVVLNELV
NP_001087969.1	XlNAP1L1A	X. laevis	NAP family	MCTDRSKFYEEVHEL<br>ERKYAALYQPLFDKRS<br>DIINATYEPTEEECEW<br>KVEEEDISGDLKEKAK<br>LEEEKKDEEKEDPKGI<br>PEFWLTVFKNVDLLS<br>DMLQEHDEPILKHLK<br>DIKVKFSDAGQPMSF<br>TLEFYFEPNEFFTNEV<br>LTKTYKMRSEPDESD<br>PFSFDGPEIMGCTGC<br>LIDWKKGKNVTLKTIK<br>KKQKHKGRGTVRTVT<br>KTVPNDSFFNFFTPPE<br>VPENGELDDDAEAIL<br>TADFEIGHFLRERIIPR<br>SVLYFTGEAIEDDDDD<br>YDEEGEEADDEEGEE<br>EADEDNDPDYEPKKG<br>QNPAECKQQ

NP_001080547.1	XlNAP1L1B	X. laevis	NAP family	MANIDNKEQTELDQQDMEDVEDVEEETGEEANSKARQLTAQMMQNPQVLAALQERLDDLVGTPTGYIESLPKVVKRRVNALKNLQVKCAQIEAKFYEEVHELERKYAALYQPFFEKRSDIINASYEPTEEECEWKVDEEEDIAEDLKEKAKLEEEKKDEEKEDPKGIPEFWLTVFKNVDLLSDMVQEHDEPILKHLKDIKVKFSEAGQPMNFMLEFHFEPNEFFTNELLTKTYKMRSEPDESDPFSFDGPEIMGCTGCLIDWKKGKNVTLKTIKKKQKHKGRGTVRTVTKTVPNDSFFNFFSPPEVPENGELDDDAEAILTADFEIGHFLRERIIPRSVLYFTGEAIEDDDDDYDEEGEEADDEEGEEEADEDHDPDFDPKKAQNPAECKQQ
NP_001001249.1	XlNAP1L1	X. laevis	NAP family	MANIDKTKKKNAALSKEQTELDQQDMEDVEDIEEEEAGEDANSKARQLTAQMMQNPQVLAALQERLDDLVGTPTGYIESLPKVVKRRVNALKNLQVKCAQIEAKFYEEVHELERKYAALYQPLFDKRSDIINATYEPTEEECEWKVDEEEDISGDLKDKAKLEEEKKDEEKEDPKGIPEFWLTVFKNVDLLSDMVQEHDEPILKHLKDIKVKFSEAGQPMSFTLEFHFEPNDFFTNEVLTKTYKMRSEPDESDPFSFDGPEIMGCTGCLIDWKKGKNVTLKTIKKKQKHKGRGTVRTVTKTVPNDSFFNFFSPPEVPENGELDDDAEAILTADFEIGHFLRERIIPRSVLYFTGEAIEDDDDDYDEEGEEADDEEGEEEADEDNDPDYEPKKDQNPAECKQQ
NP_001335312.1	ZmNAP1	Z. mays	NAP family	MSRWSEYLSVEEPIPSAMATWRNMSADAPQGSSAGSHKHLQMEPVVQLSKVAEGLLAKMYRLNSILDYPDPNTHTFSDAFWKAGVFPNFPKICITMSKKFPEHPNKLQLERVDKFGLDALNENAEGYMHNLEQWILLLLDLLAFREQALRLILDLSSTVITLLPHQNSLILHAFMDLFCSFVRVNLFSDKMPRKMILQVYNILHIMLKGGRDCEFYHRLVQFVDSYDPPIKGLHEDLNFVSPRIGEVLEAVGPIIFLSTDTKKLRNEGFLSPFHPRYPDILTNSAHPMRAQDLANVTSYREWVLLGYLVCPDELLRVTSIDVAMVVLKENLILPLFRDEYILLHENYQLYVLPKVLESKRMAKSGRTKQKEADLEYNVAKQVERMLTEVHEQALVSCDAMHRERRILLKQEIGRMVLFFTDQPSLLAP

NP_001321759.1	AtSPT6L	A thaliana	SPT6	EDDDGEPVHGDPAE<br>HDENDDEEDDDDVG<br>NEYENDGFIVNDEDE<br>EEEEEEDEERKDSDEE<br>RQKKKKKRKKKDEGL<br>DEDDYLLLQDNNVKF<br>KKRQYKRLKKAQREQ<br>GNGQGESSDDEFDS<br>RGGTRRSAEDKIKDR<br>LFDDVDVDDPPDDV<br>GDEEDLVVEEDVVGS<br>EDEMADFIVDEDDEH<br>GPPKRGNSKKKKYRQ<br>GSDITAMRDANEIFG<br>DVDELLTIRKKGLASN<br>QRMERRLEDEFEPTV<br>LSEKYMTGNDDEIRQ<br>LDIPERMQISEESTGS<br>PPVDEISIEEESNWIY<br>AQLASQLRESDGTFD<br>GRGFSVNKDDIAKFL<br>ELHHVQKLEIPFIAMY<br>RKEQCRSLLDTGDFD<br>GANQGKKPETKWHK<br>VFWMIHDLDKKWLL<br>LRKRKMALHGYYTKR<br>YEEESRRVYDETRLNL<br>NQYLFESVIKSLKVAE
XP_020528358.1	AtrSPT6	A. trichopoda	SPT6	EEVDRIVQDNEATNV<br>DGGDDDEEEEEEEGP<br>DEYEKDGFIVDDVED<br>EQEEEEEKERDHSDE<br>DLKAKKKKRRKRETEE<br>NYELDEDDYELLQEA<br>NVTGFRRPKPGSKKF<br>KRLKKAGRDTDQEER<br>TRFSDEEEIEEGGHSG<br>RTAEEKLKRSLFGDDE<br>GVPPEDISEDEQQEG<br>EDDEDIGDEDEMRDF<br>IVDEEEVDENGLPVR<br>RRKPKKKVLRQAPGV<br>SSSALQEAHEIFGDVD<br>ELLRFRKLGHGKAGD<br>GFDGVDDSVDGKPK<br>RLEDEFEPSILEEKYM<br>TEKDDRIREVDRPERI<br>QLSEEITGPVPTDEESI<br>TKEAEWIYSQLVGSA<br>TGPSPVGFQHIMRLD<br>KDHITAEIANVIGML<br>HVQKFDIPFIAMYRK<br>ELCLDLLRDPEEEVLS<br>NEERDKSDKAPPLRW<br>HKVLWAIHNFDRKW<br>LLLQRRKKALAVSYSK
NP_497969.1	CeSPT6	C. elegans	SPT6	GHSDDEEPQSKKMK<br>MAKEKSKRKKKMVA<br>SSDEDEDDDDDEEEN<br>RKEMQGFIADDDDE<br>EEDAKSEKSEKSRHSG<br>EDELDDEDLDLINENY<br>DIRETKKQNRVQLGD<br>SSDEDEPIRRPNHED<br>DDLLSERGSDDGDRR<br>KDRGRGDRGGYGSE<br>SERSEDDFIEDDGDA<br>PRRHRKRHRGDENLP<br>EGAEDDARDVFGVE<br>DFNLDEFYDDDDGED<br>GLEDEEEEIIEDDGEG<br>GEIKIRRKKDTTKKSTL<br>LESIEPSEIDRGFLLPG<br>DKKIAKEDLPERFQLR<br>RTPVTEADDDELESE<br>ALWIIKYAFEEGTVTN<br>QADLDQDDKLDCIM<br>NLDPSVYEDRKKAVIK<br>SIKKVLQFIRVRSNSFE<br>PTFIGFYRKEDIDNLLT<br>MNNLWRVYDFDEK<br>WCHLSEKKNKIYDLM<br>RRMREYQELSDDLTA<br>KRRPISDADLMDTKY

XP_645136.1	DdSPT6	D. discoideur	SPT6	NIHPKKKFKNLNKNK<br>DDESSELEEYHDSSEE<br>DENEPNQYQYDDFV<br>VGGDDDERRKRKKKE<br>KRKKSSSNSDNEDDN<br>EEEEEEDRGGGGGSG<br>SGGSGGSGGRNSSSP<br>KRTNNHLDEDIDGDE<br>DGDDGSDGSESESES<br>EEDEEVAQARRERRR<br>KRREERRKREKGGLQ<br>RLKKLRASNKDLSDKE<br>DDVEDGYNGEQEDD<br>GFVVDSHGKPVKRSH<br>ERERMSYESSNRSRI<br>MSDIFDDDEEEGYGS<br>GEEEEDSGINKTEAD<br>RAKDRLAAIREQYEPS<br>LLEEKHFTDADEEIRN<br>KNVPERLQSRKGTQY<br>AGEMACEEEAEWIYE<br>VAFEQRDFQQQQQR<br>NPNTEPVRDAKAITAI<br>FKILQFIQRDLLEIPFIY<br>TYEKDIYEPYFTLQEL<br>WNIFDLDEKWAHM<br>KVNKRNLEQIGSNNQ<br>QLEPYKSVLLESRSEES
NP_001284936.1	DmSPT6	D. melanogas	SPT6	ELDVNERKRLKKLKA<br>AVSDSSEEEEDDEERL<br>REELKDLIDDNPIEED<br>DGSGYDSDGVGSGK<br>KRKKHEDDDLDDRLE<br>DDDYDLIEENLGVKV<br>ERRKRFKRLRRIHDNE<br>SDGEEQHVDEGLVRE<br>QIAEQLFDENDESIGH<br>RSERSHREADDYDDV<br>DTESDADDFIVDDNG<br>RPIAEKKKKRRPIFTD<br>ASLQEGQDIFGVDFD<br>YDDFSKYEEDDYEDD<br>SEGDEYDEDLGVGDD<br>TRVKKKKALKKKVVK<br>KTIFDIYEPSELKRGHF<br>TDMDNEIRKTDIPER<br>MQLREVPVTPVPEGS<br>DELDLEAEWIYKYAFC<br>KHTVSEQEKPESREK<br>MRKPPTTVNKIKQTL<br>EFIRNQQLEVPFIAFY<br>RKEYVKPELNIDDLW<br>KVYYYDGIWCQLNER<br>KRKLKVLFEKMRQFQ<br>LDTLCADTDQPVPDD<br>VRLILDSDFERLADVQ
NP_660094.1	DrSPT6	D. rerio	SPT6	EEKDLKPKKTQRFME<br>EDEEEEEENTEDQDE<br>HGNLRGLIDDDDVEE<br>EEEEERGEPPAGEDS<br>DSGEEVRHRRRKRSF<br>DDYLDDDDLDLIEENL<br>GVKVKRRKKKYSRVK<br>TMDDEGDDDDEKDL<br>IADEIFTGDGDGEGE<br>VEDGEAVDTLHPRDD<br>EEEEDDEESDIDDFIV<br>DDDGQPITKKKGKKF<br>SGYTDAALQEAQEIF<br>GGDFDFAEFDTEAYD<br>HAEEEEEDQDDESW<br>DRPKKQTKRRVSRRSI<br>FEIYEPSELESSHMTD<br>QDNEIRSTDMPERFQ<br>LRAIPVKPAEDDELEE<br>EAEWIYRNAFSTPTIS<br>MQESTDYLDRGTTTN<br>FSRKGPSTIAKIKEALN<br>FMRNQHFEVPFIAFY<br>RKEYVEPELNINDLW<br>KVWQWDEKWTQLK<br>TRKQNLTRLFQRMQ<br>SYQFEQISADPDKPLA<br>DSTRPLDTADMERLK

NP_001307684.1	HsSPT6	H. sapiens	SPT6	NDEGEVVPRVTKKFV<br>EEEDDDEEEEEENLD<br>DQDEQGNLKGFIND<br>DDDEDEGEEDEGSDS<br>GDSEDDVGHKKRKRT<br>SFDDRLEDDDFDLIEE<br>NLGVKVKRGQKYRR<br>VKKMSDDEDDDEEEY<br>GKEEHEKEAIAEEIFQ<br>DGEGEEGQEAMEAP<br>MAPPEEEEEDDEESD<br>IDDFIVDDDGQPLKK<br>PKWRKKLPGYTDAAL<br>QEAQEIFGVDFDYDE<br>FEKYNEYDEELEEEYE<br>YEDDEAEGEIRVRPKK<br>TTKKRVSRRSIFEMYE<br>PSELESSHLTDQDNEI<br>RATDLPERFQLRSIPV<br>KGAEDDELEEEADWI<br>YRNAFATPTISLQESC<br>DYLDRGQPASSFSRK<br>GPSTIQKIKEALGFMR<br>NQHFEVPFIAFYRKEY<br>VEPELHINDLWRVW<br>QWDEKWTQLRIRKE<br>NLTRLFEKMQAYQYE<br>QISADPDKPLADGIR
NP_033323.2	MmSPT6	M. musculus	SPT6	NHEGEVVPRVTKKFV<br>EEEDDDEEEEEENLD<br>DQDERGNLKDFINDD<br>DDEEEGEEDEGSDSG<br>DSEDDVGHKKRKRPS<br>FDDRLEDDDFDLIEEN<br>LGVKVKRGQKYRRVK<br>KMSDDDEDDEEEYG<br>KEEHEKEAIAGEIFQD<br>EEGEEGQEAVEAPM<br>APPDEEEEDDEESDID<br>DFIVDDDGQPLKKPK<br>WRKKLPGYTDAALQE<br>AQEIFGVDFDYDEFEK<br>YNEYDEELEEDYEYED<br>DETEGEIRVRPKKTTK<br>KRVSRRSIFEMYEPSE<br>LESSHLTDQDNEIRAT<br>DLPERFQLRSIPVKAA<br>EDDELEEEADWIYRN<br>AFATPTISLQDSCDYL<br>DRGQPTSSFSRKGPS<br>TVQKIKEALGFMRNQ<br>HFEVPFIAFYRKEYVE<br>PELHINDLWRVWQ<br>WDEKWTQLRIRKEN<br>LTRLFEKMQAYQYEQ<br>ISADPDKPLADGIRAL
XP_024376317.1	PpaSPT6	P. patens	SPT6	GQDEYEKDGFIVDEE<br>EEEEEEPEEEPQESSD<br>EDSKAKKKKRKKRGE<br>ETYELDEDDYDLLQEA<br>NVTGFHRPPKADGKT<br>KFKRLKKGGIGGEKG<br>SGGIGLSDDDEEEDG<br>GGRRGRTAEEELKRS<br>LFGEDDGAAAEDVVE<br>EEELDDEEEVDEDEM<br>ADFIVDEEEEEVDEH<br>GEPSKHRKKKKKVKR<br>KDFRQAPGINSIALQE<br>AQEIFGDVTSLLEERR<br>RRERDTLAGVDDEDR<br>VDDDDDDGYGRSRK<br>PPSQKLLEKQFEPSLL<br>EEKFLTERDDRLRETD<br>VPERLQILEEAVGALP<br>TSDSAIRQAAEWIYD<br>RAFGHLAVPVRPEFR<br>HLSRDRDLISRQIANV<br>LHLIHDDKFEIPFIAM<br>YRREECFDLLKEASDD<br>YEDEERPLVRTYAAL<br>WAVQQWDKKWLLL<br>QRRKTALQAAYEKRI<br>PSDIRDDPEKDELVDK

XP_024375737.1	PpaSPT6-X4	P. patens	SPT6	VDEENELEDETGVDE<br>DRSFFKYGHLQKRRA<br>RKVQSKSTVFQKGAQ<br>WIYKGSRPSVGSRRK<br>RDRNSSAVINEESDD<br>SDENDGNDQETLAR<br>KFLEKQFEPSPLEAHN<br>VIARDDRLRSTDIPERI<br>QVLEEVAGPLPRSIVE<br>LRRASEWIFDRAFGH<br>LCLYSRSEFQHLLNM<br>DKYEVVKQIANVLHL<br>VHNEKLEIPFIAMYRR<br>EECLDLLKELTDEVSH<br>EDRRTIYRFEALWAV<br>HEWNKKWLILQRRK<br>SDLQDSYEMRIPIGIIA<br>IPEKDNLFKRILRSLAD<br>AESEQAVGDCEAQF<br>NLHFPTVEVDMVDR<br>GFKRPKCRSSYSCAH<br>DAGLGSITKYFGLTSE<br>AFGDNLKAGHKMNE<br>ILDEAFLPDDIASKFC<br>NESGPTTQFRDPASIL<br>RGARHMLAVEISTEP<br>SVRECVRMFYSKNAT<br>VNTRPTPAGMEIIDA
XP_024375742.1	PpaSPT6-X6	P. patens	SPT6	EVSHEDRRTIYRFEAL<br>WAVHEWNKKWLIL<br>QRRKSDLQDSYEMRI<br>PIGIIAIPEKDNLFKRIL<br>RSLADAESEQAVGDC<br>EAQFNLHFPTVEVD<br>MVDRGFKRPKCRSSY<br>SCAHDAGLGSITKYF<br>GLTSEAFGDNLKAGH<br>KMNEILDEAFLPDDIA<br>SKFCNESGPTTQFRD<br>PASILRGARHMLAVEI<br>STEPSVRECVRMFYS<br>KNATVNTRPTPAGM<br>EIIDAFHQFEGVKLIK<br>DKQISAFDDEQWLLI<br>HKAEAEKLIDVTVGVS<br>KESIDTFLSECEPLFSS<br>DGSTCTSQHWNEQR<br>KQILQEATVTILLSKM<br>EKEARMNLVTNAKR<br>VVVAQCVLQLWNKV<br>SIGPYDPAKGSGRKC<br>RNEEGSALRVLACCW<br>GPASKGTTFVMLDA<br>DGEILNILHTGYLSTK<br>VFTPEQAQRKENDQ<br>SRLLQVLIDFKPHIVVL
-	PpSPT6	P. polycephal	SPT6	VPEGEAAAPEGAEEG<br>YQEGQAEGQEETQE<br>GEVAEQEEGAEEEGE<br>GESREKKKKKKKKKRS<br>AFIEDEAILSGSEVDSS<br>EEEDGDSQNEYEYDD<br>FTVPTGMEEEGREGG<br>GGGERRRRRKKKRR<br>HEETLEEDDYDLVRE<br>NTGQNIARRKQDSA<br>GAGGGNKNSKKLKRL<br>KQMGNRTDLYSNIQS<br>RLFGDDDDEAGPSEA<br>ITHEEPRSDDELDDED<br>EDDFIVGADGKPIKRS<br>KQRRYRGGEDRGAL<br>QDAEDIFGSVDFFKQ<br>ASGRRSRPRGDEFDD<br>DGDYVDGTQGLSEDE<br>LDAEIEEGDEKQKDPL<br>ADIYEHYEPSLLEQKH<br>FTSEDEKIRQLDVPER<br>LQMRKGTAYDEKLVP<br>EEAEWIFTRAFARTH<br>ATDKAIIIQRIIHVLRFL<br>QEQLLEVPFISTYRKD<br>YYQPLKISELWDIYD<br>WDEKWAHLQARKN

NP_011631.1	ScSPT6	S. cerevisiae	SPT6	EIVNDNDETKAPSEEE<br>EGEDVFDSSEEDEDID<br>EDEDEARKVQEGFIV<br>NDDDENEDPGTSISK<br>KRRKHKRREREEDDR<br>LSEDDLDLLMENAGV<br>ERTKASSSSGKFKRLK<br>RVGDEGNAAESESD<br>NVAASRQDSTSKLED<br>FFSEDEEEEESGLRNG<br>RNNEYGRDEEDHEN<br>RNRTADKGGILDELD<br>DFIEDDEFSDEDDETR<br>QRRIQEKKLLREQSIK<br>QPTQITGLSSDKIDE<br>MYDIFGDGHDYDWA<br>LEIENEELENGNDNN<br>EAEEEEIDEETGAIKST<br>KKKISLQDIYDLEDLKK<br>NLMTEGDMKIRKTDI<br>PERYQELRAGITDYG<br>NMSSEDQELERNWI<br>AEKISVDKNFDANYD<br>LTEFKEAIGNAIKFITK<br>ENLEVPFIYAYRRNYIS<br>SREKDGFLLTEDDLW<br>DIVSLDIEFHSLVNKK<br>DYVQRFYAELHIDDPI
XP_001030352.2	TtSPT6	T. thermophi	SPT6	RNIGDEEEEDYQGQV<br>EEEEDGNNQVEEDDE<br>EEEDDDEQDDYQQD<br>GFVVGDSEEEEIEEEE<br>DEDAEERRRRKKEKK<br>RQKKLEKQNQHRRIL<br>KKGPKKRELSEDEME<br>NNEIQDLEEYHEDSEE<br>GIPDRKRNQQSRTG<br>NDGHQGDKMDLED<br>QGNEDYLEDVGDYG<br>AKYGKTAFEELFMDD<br>ESGEEEQNENEDDEN<br>REVDDYIDVQQLFEP<br>DDLKKRFERDDDKKIK<br>EEDIPERLQIRMQGR<br>KAPDPEELMEETKWI<br>SEKIKTIKDYKGSKKLN<br>DINFHSKIFSFLQSLHL<br>DKEEVMYIYTYKKNEF<br>HPQFDLEDLWRLYDL<br>DGEWAQFNRQKNRI<br>LLQIQQLRRELENTPL<br>LIQNNIIDFEQVRKVE<br>EFFHKAIDTQSLKFIKE<br>YFDYLFIKIYPQKEHQ<br>RLKIRKDRKTRMINTY<br>IQCKVHKLVSTLTLSP
XP_041439251.1	XlSPT6	X. laevis	SPT6	WAIAAAGSDVTPACS<br>AFYPPASALDLLLHF<br>WTKLFGKEYGFRRRQ<br>LGSALAFTSERDSPRQ<br>SRERLCSLIAMDFVES<br>EAEESEGEYEDGNTA<br>ERSNKKFVEDDDEEE<br>EEENPEDQDELGNLK<br>GFINDDDDDEDEEEE<br>DEGGSGGSDEDVGH<br>KKRKHSFDDRLEDDD<br>FDLIEENLGVKVKRQK<br>FRRVRKMSDDEDEEE<br>EDFGKEEHEKEAIAEE<br>IFQDGEDESRPDHVD<br>QPVAGPEEEEEEEDE<br>ESDIDDFIVDDDGQP<br>LKKPKWRKKLPGYTD<br>AALQEAQEIFGVDFD<br>YDEFEKYDEDDEEME<br>EYDYEDDEGDAETRV<br>RPKKTAKKRVSRRSIF<br>EMYEPSELESSHLTDQ<br>DNEIRTTDMPERFQL<br>RTISVKSAEDDELEEE<br>AEWIYRNAFATPTISL<br>QETNDYLERGQVGS<br>NFNRKGPSTIQKIREA

PWZ19394.1	ZmSPT6	Z. mays	SPT6	ADEEDPRPSRRNRDD<br>MDERDDDDDDDED<br>GQDEFEKDDFIVDDE<br>EEEVEGEEEEQKSDD<br>ERRRKKKKKKRESDD<br>FMLDEDDYMLLQDN<br>NITGISRPKPGNKFKR<br>LKKAGRESEMDEHSG<br>FSDGDGTGKKRSGKE<br>RVEYSLFGDHQDAAP<br>FEEDFEDDQQAGEGE<br>EEVGDDEDEMADFIV<br>EEDEIDGNGQVVRRK<br>KFKKKVPRQAAGVSS<br>SALQEAQDIFGDVDE<br>LLALRKQELEREALNS<br>GELRGARLEDEFEPFI<br>LAEKYMTSKDEQIKE<br>NDVPERMQLSEELTG<br>YPPTDKTTIEEESSWI<br>HSHLTGDGFLSFFGN<br>EHINKDIDQKDIVNV<br>VTMLHANKFEIPFIA<br>MYRKESCPTLLSFDSD<br>EDNEDIESDARKLRW<br>HKLLWAVQALDRKW<br>LLLQKRKVALQLYYEK<br>RFDDEKRRIDDVTRQ
NA		A thaliana	APLF	
NA		A. trichopoda	APLF	
NA		C. elegans	APLF	
NA		D. discoideum	APLF	
NA		D. melanogas	APLF	
NP_001093485.1	DrAPLF	D. rerio	APLF	VKVDASPGSEIVLINS<br>ECTVGRKKDCDLSFP<br>ANKLVSGNHCKITHD<br>QNSGKVWLEDMSTN<br>GTVINMSKVVKKQT<br>HLLQNGDVIYFVYRK<br>NEPEQNIAYVYQSITP<br>QESASHDVEDAGREE<br>DSDLTETESEPAPVEP<br>VIVKPLPQSGHEDPQ<br>PSTSSSSLHFYNMPLS<br>TCSDVSARKNPVSSS<br>AVCKGDSTSSGSPAQ<br>TRLKWTCWTDGEPE<br>EEMQRKRRKTDRDD<br>PGFGSAHSDASADIP<br>LRGASGKEKTEGATT<br>DKMEESLTCIICQDLL<br>YDCISVQPCMHTFCA<br>ACYSGWMERSSFCP<br>TCRCPVERIRKNHILN<br>NLVEAYLLQHPEKCR<br>TEDDLRSMDARNKIT<br>QDMLQPKVERSFSDE<br>EASSDYLFELSDNDSD<br>ISDMSQPYMMCRQC<br>PGYRKELSSALWICES<br>AQSESLAKTAGDGPS
NP_775816.1	HsAPLF	H. sapiens	APLF	RVALAPGETVIGRGP<br>LLGITDKRVSRRHAILE<br>VAGGQLRIKPIHTNP<br>CFYQSSEKSQLLPLKP<br>NLWCYLNPGDSFSLL<br>VDKYIFRILSIPSEVEM<br>QCTLRNSQVLDEDNI<br>LNETPKSPVINLPHET<br>TGASQLEGSTEIAKTQ<br>MTPTNSVSFLGENRD<br>CNKQQPILAERKRILP<br>TWMLAEHLSDQNLS<br>VPAISGGNVIQGSGK<br>EEICKDKSQLNTTQQ<br>GRRQLISSGSSENTSA<br>EQDTGEECKNTDQEE<br>STISSKEMPQSFSAITL<br>SNTEMNNIKTNAQR<br>NKLPIEELGKVSKHKI<br>ATKRTPHKEDEAMSC<br>SENCSSAQGDSLQDE<br>SQGSHSESSSNPSNP<br>ETLHAKATDSVLQGS<br>EGNKVKRTSCMYGA<br>NCYRKNPVHFQHFS<br>HPGDSDYGGVQIVG<br>QDETDDRPECPYGPS<br>CYRKNPQHKIEYRHN

NP_001163960.1	MmAPLF	M. musculus	APLF	VPVGPGQTVIGRGPL<br>LGITDKRVSRRHAILE<br>VVDSQLRIKPIHRNPC<br>FYQSSEKSQHSPMET<br>QVWSQLHPGDSFSLL<br>LDKYAFRVFSAESEVE<br>MECTLRNSQMLDED<br>DILSEMQKSPVVNLP<br>DKTTGASQLQGSPEIT<br>KTKCPTIDPMSSSGEC<br>RAFSEHQPRPTQRKR<br>ILPAWMLAESLSDQS<br>LSTPAEGGDKDVIQR<br>SGKAGTCEDRTPGNT<br>SWHGKKRLSPSGNSK<br>SVSAEQDPGKKCRKA<br>DQEGPGVSSENVPES<br>SSSNIVKDPDVDIVKT<br>NKQKDGILIEELGEVS<br>KHKAATKPTTNEEGE<br>SCARVQSKSPPEKSQ<br>GCHPESSSAPSSPDAL<br>HTDTADPVLGCSEES<br>KVRRTACMYGANCY<br>RRNPLHFQHFSHPG<br>DSDYGEVHGTDEGVI<br>GDRPECPYGASCYRK<br>NPQHKMEYRHSALP
NA		P. patens	APLF	
-	PpAPLF	P. polycephal	APLF	AQNQEKKEKEKEKKK<br>GGSVGEGGARWVLE<br>CSEEGDIKVELMGGP<br>TELGRGLVNNKKISRK<br>QCVVETVDDGVELTP<br>LGVNPMTMSRAGSE<br>DVEILSRNEKYTLHEG<br>DSFTLCGEQYRFVLQ<br>RPTVTQTTTSQTTSQ<br>KDGEKSAKPASRQAS<br>KIEGAEGEGEKTKRD<br>GEADEINEGDKGEEE<br>GREKQRGEKGEEGEK<br>GEEGSPEKKGVKRKA<br>SDIATTTTTTSSAKKTP<br>QGSAESDSATVPFSA<br>GYDSPEESKAKSKKKP<br>DVKTEEADDVIDDEEL<br>ARKLQEELNKEDEAP<br>KPKTPKSKNSTPEKD<br>AEIARKLQEKYNKESG<br>GGGKKKRKQEDDED<br>DGEVNWAAKVKKQK<br>KGKKGGSDDEDEDW<br>NDVGGGGDEDSDED<br>YQEEESDSEKNKKNK<br>KKGASPKKPPCKYGK<br>SCYRTNPDHLRDFSH
NA		S. cerevisiae	APLF	
XP_001009833.2	TtAPLF	T. thermophi	APLF	KGGLVQETFVLNQKE<br>HILGRRGDLKVDNPK<br>VSGQHCVLKYDYAQK<br>KAYIIDVSSNGTSLFN<br>KKLEKNKEVELENGDL<br>VNLLQDKSQWIGYIF<br>KLVDNVDSFNKDQQ<br>DTATKNNTDQKQLE<br>QSLEQYKQNEKEQQE<br>QNEQIKKMQNELEER<br>LKKVKEDDEHFEKDQ<br>TCVVCIDLLYNPYLMT<br>PCLHNYCCDCMCELL<br>KNKDIACPQCREKPIS<br>VQKNYQLNNLIEAFIK<br>RNPDKKWQEDVIKK<br>KNESNLLNKDFLDQI<br>NEKLSNKTKKVGIYKR<br>QRSYSDSESSDRNNN<br>RNYSEEDEEEFDEDEE<br>EEEYQYNNNQPFQQ<br>FGYNYNFIMPAHLNR<br>CVECQNARQDDNFK<br>CSPYQQHIKCINCDK<br>MMPQRNDNNLYPQ<br>NCTICERSFCNLYFKQ<br>DCSSKYNSSISHHLRL<br>FQDIPIPQVLNNNVLS

NP_001089240.1	XlAPLF	X. laevis	APLF	RLLGAETDSSSELFLYK<br>KEWTIGRKKACDLSF<br>PGNKLVSGEHCKITV<br>NEESGEVSLEDTSTN<br>GTVINKLKVIRKQTYP<br>LKNGDVIYVVYRKNE<br>PEQNVAYLYKSLNQG<br>QDSLHDPADTSGSEE<br>AETQTLSSQDDQLSY<br>EEPQPSTSTSSLFSTPT<br>TSAIPGVQLESAEKSG<br>ESLGGHSSTSDASPAI<br>RASIPKSNLSTQEQGS<br>LGPPKKRIRTEDHWT<br>TNKNFVPASCPIGAS<br>DESKTPSMKPDKMEE<br>TLTCIICQELLHDCVSL<br>QPCMHTFCAACYSG<br>WMERSSLCPTCRCPV<br>ERICKNHILNNLVEAY<br>LIQHPEKCRSEEDRCS<br>MDARNKITQDMLQP<br>KVRRSFSDEEGSSEDL<br>LELSDVDSESSDISQP<br>YTVCRQCPGFVRHS<br>MQPPPYPPPSDTETS<br>RTQGDAPSTSTNFPT<br>ATQEYVCPSHGSHVI
NA		Z. mays	APLF	
-	PpXRCC1	P. polycephal	XRCC1	DAEQKASNIAVQDLY<br>ATWQTEPGEEKASILL<br>ELPAETTLHTIEIVNA<br>GSTNVEIYVTNSENQ<br>QDEEILLPTAIMMTL<br>GEVNSNTNTNRLRFF<br>GPDKLTKGISDKAWK<br>YIKIVCISFDTSRKPFG<br>LARIRLFSSAPSDEYIA<br>SSIADNRSSMVSVPSS<br>QPTTSTYVPQKYASR<br>SKSFAGPPPDTTIAHD<br>TNNKKTPNHTNTAN<br>KSNNNNRTNDTNNS<br>PAKSAAKRKRVIMND<br>DYDDTEGDSAKDQN<br>PRSLTSLHISPHVKKS<br>VPVANRSKSLVFTSFN<br>KILSGVSFVVSGIANP<br>ERQELRDKAIEMGAQ<br>YYADWIESATHLICPF<br>TNTPKFILAQKAGGTI<br>VTPAWILDCYKNKVR<br>LPETEYLLAEVKKIDD<br>AGMKSTMGNMRD<br>WLATFRPKFDKPAN<br>NSTNTTQSQPQPKLT<br>KSLTFVNNARITKDSQ
NP_006288.2	HsXRCC1	H. sapiens	XRCC1	DSTHCAENLLKADTY<br>RKWRAAKAGEKTISV<br>VLQLEKEEQIHSVDIG<br>NDGSAFVEVLVGSSA<br>GGAGEQDYEVLLVTS<br>SFMSPSESRSGSNPN<br>RVRMFGPDKLVRAA<br>AEKRWDRVKIVCSQP<br>YSKDSPFGLSFVRFHS<br>PPDKDEAEAPSQKVT<br>VTKLGQFRVKEEDES<br>ANSLRPGALFFSRINK<br>TSPVTASDPAGPSYA<br>AATLQASSAASSASP<br>VSRAIGSTSKPQESPK<br>GKRKLDLNQEEKKTP<br>SKPPAQLSPSVPKRPK<br>LPAPTRTPATAPVPA<br>RAQGAVTGKPRGEG<br>TEPRRPRAGPEELGKI<br>LQGVVVVLSGFQNPF<br>RSELRDKALELGAKYR<br>PDWTRDSTHLICAFA<br>NTPKYSQVLGLGGRI<br>VRKEWVLDCHRMRR<br>RLPSQRYLMAGPGSS<br>SEEDEASHSGGSGDE<br>APKLPQKQPQTKTKP

XP_041427601.1	XlXRCC1	X. laevis	XRCC1	DTTHTAENLLKADTY<br>RKWKAARPGEKQISV<br>ILQFEKEEQIHSIDIGN<br>EGSAFVEVLVGRSTSI<br>SEQEYEVLLGMSSFM<br>SPSESKNESNLNRTR<br>MFGPDKLVKGAAEK<br>KWDRVKIMCTQPYT<br>KNLAYGISFIRLNSPPE<br>DGSPTAPSPKVTKLG<br>QFMVKEEENSSPSM<br>RPGSLFFNRTSKPQV<br>TPPKTPPATQSYAAA<br>ALQGTAETSSSTEKQI<br>KTPPSNNITKEPSSSK<br>RKFEFNKEPSSHSAVK<br>KPDVKESPSSTKAKTS<br>SQPAPKKPKVETPPT<br>KKPSPSEKPNQKKSP<br>PSSQPMDLGRILQGT<br>VFVLSGFQNPFRSDL<br>RDKALEMGAKYRPD<br>WTPDSTHLICAFANT<br>PKFSQVKAAGGIIVRK<br>EWVLDCYKKRQRLP<br>QKQYLLGTAESSSDEE<br>DDSEEDEPPKAPQHK<br>PHPDPSRTNHKKPSP
NP_001003988.2	DrXRCC1	D. rerio	XRCC1	DNTHKADNLLSSDTY<br>RKWKAARPGEKQVS<br>VILQLEKEEQIHSIDIG<br>NEGSAFIEVLVGHSTS<br>VKDQDFEVLLVTSSF<br>MSPTESRNGTNTNR<br>VRFFGPGQLVKAQS<br>QEKWDRVKIVCSQPY<br>SKTIAYGISFIKLHSPP<br>DGNEALVSTPPKLTKL<br>GQFRVKDESPSAGSN<br>VQPGALFFSRDTSAK<br>SSTGLKVSPQNERLSY<br>AAAALKTEGSSTHTP<br>ASDTQQAPVKRKFEF<br>SKERLEASGPPAKKPS<br>SSASAETTPKAKARSS<br>MGTSPSSRASPAQKS<br>SDKRESPKTKPESKPK<br>PKPKPKSSESVPFNRI<br>LEGVVFVLSGFQNPF<br>RADLRDKALALGARY<br>RPDWTPDATHLICAF<br>ANTPKYSQVKAAGGI<br>IVRKEWVMDCHKNK<br>QKISCKRYLMDGAES<br>SSESEAEQDESEEETP<br>KAKTPEKKPVTPKKQ
NA		T. thermophi	XRCC1	
NP_001326839.1	AtPIE1	A thaliana	SWR1	ASKSGKSKPDNESRA<br>KRQKTLEAPKEPRRP<br>KTHWDHVLEEMAW<br>LSKDFESERKWKLAQ<br>AKKVALRASKGMLD<br>QASREERKLKEEEQRL<br>RKVALNISKDMKKF<br>WMKVLYKHQLVRNE<br>KKKKAMDKQLEFLLG<br>QTERYSTMLAENLVE<br>PYKQGQNTPSKPLLTI<br>ESKSDEERAEQIPPEI<br>NSSAGLESGSPELDED<br>YDLKSEDETEDDEDTI<br>EEDEKHFTKRERQEEL<br>EALQNEVDLPVEELLR<br>RYTSGRVSRETSPVKD<br>ENEDNLTSVSRVTSP<br>VKDENQDNLASVGQ<br>DHGEDKNNLAASEET<br>EGNPSVRRSNDSYGH<br>LAISETHSHDLEPGM<br>TTASVKSRKEDHTYDF<br>NDEQEDVDFVLANG<br>EEKDDEATLAVEEELA<br>KADNEDHVEEIALLQ<br>KESEMPIEVLLARYKE<br>DFGGKDISEDESESSF

XP_020520860.1	AtrPIE1	A. trichopoda	SWR1	KDDETTLAEEEEFAKS<br>EANDPSDEIKLLQQES<br>EMPLEELLAMYKMD<br>TCKDEDDLSASECAST<br>SSEEQVDHSGNKEVK<br>GEDSGPDEDRDGLTV<br>LPSDSTEIESFSPLKHS<br>VMQKLKGKLENRTEE<br>GRESENIIADAAAAA<br>RSAQPTGNTFLTTKV<br>RTKFPFLLKHPLREYQ<br>HIGLDWLVTMYEKRL<br>NGILADEMGLGKTIM<br>TISLLAHLACEKGIWG<br>PHLIVVPTSVMLNWE<br>TEFLKWCPAFKILTYF<br>GTAKERKNKRQGWL<br>KPNSFHVCITTYRLVI<br>QDAKVFKRKKWKYLI<br>LDEAHLIKNWKSQR<br>WQTLLNFNSKRRILLT<br>GTPLQNDLMELWSL<br>MHFLMPHVFQSHQE<br>FKDWFSNPISGMVE<br>GQDRVNKEVVDRLH<br>NVLRPFILRRLKRDVE<br>KQLPKKYEHVIYCRLS<br>RRQRNLYEDFIASSET
NP_001255180.1	CeSSL	C. elegans	SWR1	RTSSRSVADDQPSTSS<br>AVAPPPSPIAIETDED<br>AVVEEEKKKKKTSDDL<br>EIITPRTPVDRRIPYICS<br>ILLTENRSIRDKLVLSS<br>GPVRQEDHEEQIARA<br>QRIQPVVDQIQRVEQ<br>IILNGSVEDILKDPRFA<br>VMADLTKEPPPTPAP<br>PPPIQKTMQPIEVKIE<br>DSEGSNTAQPSVLPS<br>CGGGETNVERAAKRE<br>AHVLARIAELRKNGL<br>WSNSRLPKCVEPERN<br>KTHWDYLLEEVKWM<br>AVDFRTETNTKRKIAK<br>VIAHAIAKQHRDKQI<br>EIERAAEREIKEKRKM<br>CAGIAKMVRDFWSS<br>TDKVVDIRAKEVLESR<br>LRKARNKHLMFVIGQ<br>VDEMSNIVQEGLVSS<br>SKSPSIASDRDDKDEE<br>FKAPGSDSESDDEQTI<br>ANAEKSQKKEDVRQE<br>VDALQNEATVDMDD<br>FLYTLPPEYLKAYGLT<br>QEDLEEMKREKLEEQ
XP_647182.1	DdSWR1	D. discoideun	SWR1	TDESMNIDDNGGDQ<br>IHNALSPTLSEDTVTS<br>NTNPTTTTTPRQTRS<br>SLSLSSPSIDNTIALAT<br>ATNSSKKRSRSNSTSK<br>NKSNTGSSSSTNPSP<br>MGSPTLSTSTINLLNT<br>TTTTTTTSTIKQIELNL<br>QKNKDEFIDINNKRNI<br>LIKELLILQQQDNNN<br>NNNNNNNNNNNN<br>NNNNNNNNNNND<br>DIKQQQQLSLLNLDQ<br>DKLQLINGKVEEFCNS<br>NGFSFYSQIFIDNLSSL<br>TSKSLQPTTTSSKIPTI<br>PVEKPIIEKPTLPERPK<br>SERLKRQQSLLNSTDS<br>KKLSTTSTTTTTTTTPT<br>TTTATTPPPPPSSSSTS<br>TSTSTSGTPIGGINTTP<br>TNTTPTSTNNNNNNK<br>GICNIKDTTPIPFSLAE<br>ETLTDKSTPIKTSIQQP<br>QKKQRRESSSTQLNK<br>QITEQIQQQQQPIEEI<br>TIPIEQQQMQLLAQV<br>NPQDFKPIPLPPPSAL

NP_524833.2	DmDomino	D. melanogas	SWR1	SPAPPAVPDRVTPHS<br>TEISVAPANSTSTTVR<br>AAGSVGAALPATRH<br>HQHIATQVKGIASSSS<br>KQQKQLASAQLPVPL<br>SPLPQQQQQTAEAT<br>AAAAAPAHSNVSVSS<br>STIEASVLPPQAKRQR<br>LDDNEDRTSAASIVG<br>PAESSNIVSSLLPASV<br>ASSSEVGGLSSTALQD<br>LNALKKRILQQKLQIL<br>RNLKERHLENVSEYFY<br>LQNGGSMMDYPAW<br>RKKTPTPQFISYSNAN<br>RIDQLIHEDKPSTSAA<br>AAAAQNQKYTTQQT<br>DSVESSLVSGIGTGAT<br>KGAPLDGNISNSTVK<br>TNTQSQVPSKIGSFTE<br>STPAATESNSSTTVPG<br>TATSGAATSTSATSAE<br>ASGNVLAVEAEIKIPA<br>VGATPVAISTKLPAAV<br>VQLTQQGGTPLLPCN<br>TSAGSTALRRPQGQN<br>NASSGSAAASGGGG<br>SLTPTPLYTGNGPAAL
XP_005156368.1	DrSRCAP	D. rerio	SWR1	QHHGDTGGVSAPFQ<br>TAKEPLPPETIKLDYK<br>NVSGKATSGESPSAP<br>KTEVTPSVKGAVKST<br>DKDMEHPKPTPLFET<br>NQTAASSVLQKQSTT<br>NSATQLCKFDSKETM<br>EDVTRDLLKTSKEDDI<br>SETLSAGGTKQGISG<br>NSICSGYSSKQGGQH<br>ITPNRGNNTPNDSDK<br>GSCYLTENCSQTQTS<br>TQKTSTLPKTPGIPTL<br>VENIQCVQELSSQYL<br>HINPKMVTLSKETSTR<br>GDLYCNVAAKEVLCN<br>VSVDEPKLSGHGTCT<br>DTAHVSIMSERSQV<br>MGCGSSDVILKQNDS<br>ANQIEISMSSNHQQ<br>DGSILLTHAENCVTSE<br>GLESDQPNLSDCLVQ<br>QLQESIPTDHKLSQIQ<br>ICLNNTPDIVNEEERQ<br>EMNAEALSAVEVKDL<br>IDVGILMQPDMEDT<br>WLVVSEIDGQPEHAE<br>MEVSDESLVQCPESQ
XP_009302483.1	Dr-p400	D. rerio	SWR1	LQRSKSFTGSEAEDQ<br>QQQQSQQPQQQQ<br>QQPVPVTQQQSANA<br>NLPQSPVTSFAPSASP<br>SAPQSPNYQIIMSRS<br>PVTGQNMNITLQNV<br>GPMVTGNQQITLTSL<br>PLQSPASPGFQHQP<br>QQWRFEPGSSSYIQV<br>TSPLPQTMQPQSPT<br>QHSPVPLQAVPRPN<br>APGSALGVCGQSPTR<br>FVEANIMVRQINLSS<br>PPGSGHFVYQDGAG<br>LATGTVHLTSPGTPG<br>AVRERRLSQPHSQPG<br>SSTVHHKALPSQVPT<br>LGSPGPITTTNLPPQI<br>SSIIQGQLARPTMFEK<br>TSQSVVGVANSAAAT<br>FGVPSTIPPSSPSRSN<br>SSQGLSSQSLTPTSM<br>KKMQPKKLEEIAPSN<br>PEVAQLRKQCLEHHG<br>KKMEGLKEVFKEYLIE<br>LFFLQHLQGNMMDY<br>IAFKKKPCVPLFTYLR<br>QNDLDLEDEEEEEEQ

NP_006653.2	HsSRCAP	H. sapiens	SWR1	QTQMVSDGMTGSN<br>PVSPASSSSPASSGAG<br>GISPQHIAQDSSLDG<br>PPGPPDGATVPLEGF<br>SLSQAADLANKGPK<br>WEKSHAEIAEQAKHE<br>AEIETRIAELRKEGFW<br>SLKRLPKVPEPPRPKG<br>HWDYLCEEMQWLS<br>ADFAQERRWKRGVA<br>RKVVRMVIRHHEEQ<br>RQKEERARREEQAKL<br>RRIASTMAKDVRQF<br>WSNVEKVVQFKQQS<br>RLEEKRKKALDLHLDF<br>IVGQTEKYSDLLSQSL<br>NQPLTSSKAGSSPCL<br>GSSSAASSPPPPASRL<br>DDEDGDFQPQEDEE<br>EDDEETIEVEEQQEG<br>NDAEAQRREIELLRRE<br>GELPLEELLRSLPPQLL<br>EGPSSPSQTPSSHDS<br>DTRDGPEEGAEEEPP<br>QVLEIKPPPSAVTQR<br>NKQPWHPDEDDEEF<br>TANEEEAEDEEDTIAA<br>EEQLEGEVDHAMELS
NP_056224.3	Hs-p400	H. sapiens	SWR1	LQRSRACPGSEGEEQ<br>PAHPNPPPSPAAPFA<br>PSASPSAPQSPSYQIQ<br>QLMNRSPATGQNVN<br>ITLQSVGPVVGGNQ<br>QITLAPLPLPSPTSPGF<br>QFSAQPRRFEHGSPS<br>YIQVTSPLSQQVQTQ<br>SPTQPSPGPGQALQ<br>NVRAGAPGPGLGLCS<br>SSPTGGFVDASVLVR<br>QISLSPSSGGHFVFQD<br>GSGLTQIAQGAQVQL<br>QHPGTPITVRERRPS<br>QPHTQSGGTIHHLGP<br>QSPAAAGGAGLQPL<br>ASPSHITTANLPPQIS<br>SIIQGQLVQQQQVLQ<br>GPPLPRPLGFERTPG<br>VLLPGAGGAAGFGM<br>TSPPPPTSPSRTAVPP<br>GLSSLPLTSVGNTGM<br>KKVPKKLEEIPPASPE<br>MAQMRKQCLDYHY<br>QEMQALKEVFKEYLIE<br>LFFLQHFQGNMMDF<br>LAFKKKHYAPLQAYLR<br>QNDLDIEEEEEEEEEE
NP_001291195.1	MmSRCAP	M. musculus	SWR1	QTQMVSDGMTGSN<br>PVSPASSSSPDSSGA<br>GGISPQHIAQDSSLD<br>GPPGPQDGTTVPLEG<br>FSLSHAADLVNRGQK<br>WEKSHAEIAEQAKHE<br>AEIETRIAELRKEGFW<br>SLKRLPKVPEPPRPKG<br>HWDYLCEEMQWLS<br>ADFAQERRWKRGVA<br>RKVVRMVIRHHEEQ<br>RQKEERARREEQAKL<br>RRIASTMAKDVRQF<br>WSNVEKVVQFKQQS<br>RLEEKRKKALDLHLDF<br>IVGQTEKYSDLLSQSL<br>NQPPASSKAGSSPCL<br>GSSSAASSPPPPVSRL<br>DDEDGDFQPQEEEEE<br>DDEETIEVEEQQEGN<br>DAETQRREIELLRHEG<br>ELPLEELLRSLPPQLLG<br>GPFSPSQTPSHDSDT<br>QDGPEENIEEEPSQD<br>LEVHPPSSAVTQCNK<br>QRWHPDEDDEEFTA<br>NEDEAEDEEDTIAAEE<br>QLEGEVDHAMELSEL

NP_083613.2	Mm-p400	M. musculus	SWR1	LQRSRSFTGSEEEQPA<br>HPNLPPSPAAPFAPS<br>ASPSAPQSPGYQIQQ<br>LMSRSPVAGQNVNIT<br>LQNVGPVVGGNQQI<br>TLAPLPLPNPTSPGFQ<br>FGAQQRRFEHGSPSY<br>IQVTSPMSQQVQTQ<br>SPTQPSPGPGQTLQN<br>VRAGAPGPGLGICSN<br>SPTGGFVDASVLVRQ<br>ISLSPSSGGHFVFQEA<br>PGLTQMAQGAQVQ<br>LQHSGAPITVRERRLS<br>QPHAQSGGTIHHLG<br>PQSPAAAGGTGLQPL<br>ASPNHITTASLPPQIS<br>SIIQGQLIQQQQQVL<br>QGQPMNRSLGFERT<br>PGVLLPGVGGPSAFG<br>MTSPPPPTSPSRTTM<br>PPGLSSVPLTSMGSS<br>GMKKVPKKLEEIPPA<br>SQEMAQMRKQCLDY<br>HYKEMEALKEVFKEYL<br>IELFFLQHLQGNMM<br>DFLAFKKKHYAPLQA<br>YLRQNDLDIEEEEEEE
XP_024369996.1	PpaPIE1	P. patens	SWR1	GRRNRAKPEPSSVES<br>GPPLVKDVRGLTPVK<br>KVSPTPTSDQAVDVF<br>VAQVKKPHAGRGRP<br>RKVVGVPKQGVKSLS<br>DVVTKKMPATRSSA<br>GKPVAFVASDGREVA<br>KDSISIEETVPEGSSLK<br>TLVRSQDKVPVRVRI<br>VGPFSNGGSKHGRD<br>ESFGSLSSGSQKSRG<br>QIVRQEAQRAADDET<br>SDGLQPNQRKSDGL<br>AARDEKISCRREIERSS<br>IHEKKVAEEQVIDKRE<br>EGQTVPEKKRTAHFH<br>YGRKDAVEQVIDKRN<br>QWLPVTERKMGGKT<br>HHDIHETGEQVIHKR<br>GEGRYIFEMSAGGKS<br>QRERIDGEHSTRGKH<br>PSASEGTLASRRRFM<br>ALRRRQREDLLLSRA<br>DEILLKQEFLEANKDK<br>KVQPKSKKPARPKTH<br>WDFVIEEMTWLAKD<br>FERERKWKLTQAKKI<br>VLRVNRSKLDVVARE
-	PpSWR1	P. polycephal	SWR1	GNKRQKGVAEPPIHD<br>TRNANTKRPSRTSEQ<br>ASPIPKKRVRGSGVPE<br>SPSVEAIDVSQESEVA<br>PKAGVETDINIPAIRE<br>YEVEKRLELIKQLTEQ<br>KENELKELAYLEAGN<br>NLMDYEAGFTKGKI<br>MTAKEEPGENVAVG<br>AEVPNKKGPGRPAK<br>DPNSQSRRRSTVGQS<br>PVQGESLQAQSPIPQ<br>TPSAPQTPVQPSQPT<br>PQTPTQPTEQITAQE<br>AKPAVVFHQPLQVPL<br>PSIFTVPPPTSEQDRE<br>RALKDAAIIRRVGELQ<br>RTGMWSLKRIPKAVE<br>PPRAKVHWDHVLAE<br>MGWLANDFKQERK<br>WKMALAKKVSKQV<br>MKYHQMQETREQR<br>KRKEEEHQIRKIASGI<br>AREVKKFWQKIEKVV<br>MFKEQTKLDEKKKEV<br>LDKHLDFLVGQTEKYF<br>EMIANDLKQPEGGA<br>QKDAPTTEQSTEEKA

NP_010621.1	ScSWR1	S. cerevisiae	SWR1	GGEQDLADLKFRYDL<br>LTNELFHLREFVSLVD<br>YDPTHFNDSESFQKFL<br>RETHLSLEERGEKFTD<br>DVAKKGTNGDLTRRR<br>RNLRTSTVVSSETTNE<br>KKGDIELKLESIAPLVR<br>NKCEELKYKLSDHSN<br>RKSIVPQKRPIQHLKK<br>REAAKSLKFKSERKEN<br>PLPLHEHIAEERYDHI<br>AKVEEPSEAFTIKCPS<br>DDSSFENTSEHYSDN<br>FYFTTSSEEEDIKKKRG<br>RKKKKPRIKLVVHPPK<br>QTITNPLHVVKPGYES<br>LHEYIASFKSLEDDLTL<br>EEYNKYIDEQRRLLSR<br>LKKGIENGALKYDKET<br>DSLQPITSKEIKTIITYK<br>PDPISYFYKQQDLQIH<br>TDHLINQGIHMSKLF<br>RSSTKARIARAKKVSQ<br>MIEQHFKHVAGAEE<br>RKAKEEERHKKSLARF<br>AVQAVKKRWNMAE<br>KAYRILRKDEEEQLKRI<br>EGKQHLSKMLEKSTQ
XP_001018403.2	TtSWR1	T. thermophi	SWR1	YFFDCLFVSFRKAWS<br>CVNFIYICFYQIYYGAE<br>GNLFLSYFTKIFRRKK<br>QIGSQLKKIQEQVQK<br>QMNSTEHDGQGLNS<br>SIRNYKNNSDISYEQN<br>STNLSKNQDSYKDSIK<br>KILETMKTESEKQNKK<br>YDENLRKIDQWRQE<br>MKINFEISDDEMNDE<br>DSYNFDFDPENQLRQ<br>SDLANQKYNQLIEEQI<br>DKLLQSSIIDKELDIVK<br>QRIHNAHSSSTFLESK<br>KAFLKNKRKNLFKNY<br>VKTVNQNNQNAEKI<br>QNWQNGNNNLSTG<br>VSSTSSQVSSSSNVQT<br>LEQIIKESMKLQSSKN<br>SKLIEHQLMRLTQAIE<br>SPRHDDFIENHIFKKF<br>KVTKDKLQTRTQQVI<br>GYKPSDFRKIKRYRFN<br>PQNMPFCGSNFDEA<br>KKALVPQYHAVYND<br>QSDPFNRLLSKPIIPRI<br>GVNKQQLQMKPKK<br>NVNEGNQPEPTTSPL
XP_018092196.1	XlSRCAP	X. laevis	SWR1	KMTGSNPVSPGSSAE<br>LSPFDLSGEMGDTAC<br>GSPTYDGMDGSQQD<br>NSGGQKWDGQSEIA<br>EQAKHEAEIENRIAEL<br>RKEGFWTPRRLSKVP<br>EPTRPKVLWDYLCEE<br>MQWLSADFAQERR<br>WKRGVARKLVRMVV<br>RHHEELKQKEERARR<br>EEQAKLRRIATTIAKE<br>VRQFWSNVEKVVQF<br>KQQSRLEEKRKKALD<br>LQLDFIVGQTEKYSDL<br>LSQSLNETLLPVSKSSS<br>SCIGSSQGGSLRTSPT<br>PSVHHNYDGDFLPH<br>DEEDDEETIEIEERQD<br>GNDAESQRLEIELLKK<br>ESELPLEELLESLGPNF<br>LQDVDPDQDMQESD<br>SPLLPQVFEEEDGEFT<br>VNEEEGEDEEETIEAE<br>EALEGQQDHAEELSE<br>LAKEGELSLEELLQKYS<br>MVKTDEEIFDPSEES<br>QQSDQLSDSSHCENS<br>DSDSEGVEFLVKPED

XP_018117870.1	XL-p400	X. laevis	SWR1	PSSGNDGDQQRTQK<br>TTPPSPAQSFTPTVSS<br>PAPQSPSYQIQHLIM<br>SRNSMPGQNVNITL<br>QNVGPVVPGSQQITL<br>TSLPITSPASPGFQFN<br>TQPRRFEHGSPSYIQ<br>VTSPVSQQVQTQNS<br>TQPNTVPIQALQGVR<br>TGTSSASLGMCSQSP<br>TRGFVDAGLLVRQLS<br>LGPSNTGTFVIQEGS<br>GITQIAQSAQVHLSP<br>GTPTVPAHNLLHSSS<br>PTVGPVHQFGSQNS<br>VTGSVNVQSPSTPSQ<br>LTTNLSQQLSSIIQGQ<br>LIQQQQQQQVLHGQ<br>QVSRTFSFDGTSSGM<br>IAGVTGTSAFGITSAA<br>TPTSPSRVTGPQARS<br>SLPLTTTLGTTLKRQA<br>RKLEEIPPATQEDALL<br>RKLCFDYHHQQMQA<br>LKETYTEYLIELFFLQH<br>CQGNMMDFLPFKKK<br>RNIYQNFLRQNDLD<br>MEVDEEEADKNSSRA
AQK73705.1	ZmPIE1	Z. mays	SWR1	RARRQKALEAPREPR<br>RPKVHWDHVLAEML<br>WLAKEFDSERKWKLS<br>MAKKIAQRANKSIVD<br>QAAKGERKQKEEEHR<br>MRKVAVNISKDVKKF<br>WIKIEKLASSFLSSNVI<br>VNCDTTLDISFLKKTEL<br>YFATSFLSSNVMGKQ<br>VVYKHQLELEERKKK<br>ALDKQLDFLLGQTER<br>YSTMLAENLVDMPYS<br>QNLETETSQINQPSH<br>QEVVAEENLNATIPD<br>DLDNMEVDDDYGSS<br>LDEEPEDDEHTIDEDE<br>AQITEAERNEELAALE<br>AEADLPLDVILKMYTK<br>TKVTPNQANGCDHE<br>LAHSSSDEGNSSEEV<br>DDGHSYAEFVKKNH<br>VHLLLSYMWVKVMEI<br>FLLSMIRHVLVVYVVY<br>GFIYSLSHEDEDYIAAE<br>EVKDDEATLSEEEQLA<br>KKEDPDHLEEIKLLQK<br>ESEIPLEELLARYREDG<br>CADHETAELENSPHF
NP_181212.2	AtSWC2	A thaliana	SWC2	MEIDEEEPMVFLDRT<br>TRATRGKRMTKLLDD<br>EVEEDEQFWNQEAL<br>KEEEHDDEYEAEREV<br>ADEFDSDFNDDEPEP<br>DAVAVNEKELRDLPK<br>KRLIYPGKTASKKKKK<br>KTKVVSQLEYIPGDEK<br>PGEELGNKEQEEKEE<br>NEAQEDMEGEKVIRK<br>STRTSVVVRQAERDA<br>LRAAIQATTKPIQRKK<br>VGEEKRMTQEEMLLE<br>AAQTEIMNLRNLERV<br>LAREEEVKKKAIVHKA<br>VYKGPQIRYHSKDGC<br>NYLEFCNGASFNSELS<br>TKSVPYPEKAVCVITG<br>LPAKYRDPKTGLPYAT<br>RDAFKAIRERFMDEH<br>DGLRKKMEMGDLFD<br>TLVAKGFATKQKRTKI<br>PKSNKSFSLRSSARFL<br>SSESEEESEEDSD

XP_011620654.1	AtrSWC2	A. trichopoda	SWC2	MDPMASSAEQEVM<br>LLPRSARKTRGRRMS<br>KLLDEEIEEDELFWNQ<br>DALKEEELDAEYEEEA<br>EVADEFDSDFDEDEP<br>EPDDEEAKNDGDERE<br>RVKKKLIFPGKKAVKK<br>KSKKKVLAAIPKAENS<br>SETPEPMQLEHQDIP<br>DDLEGERAVRKSTRT<br>SVIVRQAERDAIKAAL<br>QATMRPVKRKKEGE<br>EKRMTQEEMLLEAA<br>QTEIVNLRNLERVLAR<br>EEEVKKRAVVHKAVY<br>NGPQIRFLSRNGKTFL<br>EFNNGATFESAIATTP<br>ASYAEPAVCAITSMP<br>AKYRDPKTGLLYATKE<br>AFKIIRERFYKEVSERE<br>KERPDMGSLFALISGE<br>GFSMKRKRSCMENR<br>NQHLDYRRQYIF
NP_492505.1	CeVPS72	C. elegans	SWC2	MAPRRRSSRAVKNK<br>KESREGSSNDEHSNE<br>SSTRSSKSRDGSHHN<br>EEEGETSKASDPQINE<br>ESEDETSNASDEQEE<br>NDDTSSDEDAEPIVL<br>NVTERARRATAGNK<br>MAALLASADHEDEFY<br>KTAYGGFEENNEVDK<br>EFKSPVHSDDDEVDS<br>DFDKPEEEDEPASGG<br>EEDGRPRRKKRKFNE<br>PKRGMTADDILAKNK<br>KWAMARLAGNIVAA<br>NSVDDKTQAAMLKE<br>AEKTEKMNIESLKKYE<br>AFELERKRKREKNTVR<br>VFPPGPREQIKMTES<br>GTTITLSEIKTFKCERP<br>RERNLCAVTGRPARY<br>LDPVTRLPYSTAYAFK<br>VIRDRYHKHLRSIRGN<br>DEVTTYLKSLKALPSP<br>QISPRVPLLTTGPITSG<br>PLVASSAGLTVMATS<br>TSSAPKTTTH
XP_629104.1	DdYL1	D. discoideum	SWC2	LSTADEELWQQLLKE<br>SDNEKEDPDYIVNSD<br>EEEEEEDIVDSDAASD<br>EEEEEGGGDDETETK<br>STKKKSSKSYKDPKSK<br>KSISSRRKKSSSDTDSK<br>STMETELEKHLDNNT<br>STTTTNDNTPNKRKR<br>KEPSPPKTPNSEPTSP<br>STRRSSQRSSAIEQLQ<br>KSQPQTLTKNKKLKV<br>DDDDEEDESTSTTTTT<br>TTATTRSRRSTRNSES<br>IKTTETTTTRKGGKNS<br>KSDKNDEISSTPSTPSI<br>DGEGLSKLDTISTYGL<br>TQEELLEECKETEIYNT<br>ESLNHLLQQEEDKKK<br>VFHPKKAILTGPRIIYR<br>STPEQTTITFTDSILPY<br>CLTNQPQINNITSSG<br>NEKKKKVRASRKSLD<br>PPPIVDITSTTTTTTTA<br>ATTATTNPTTTATVTT<br>ETNPTTTSTQEPIVEQ<br>KDKGDTNRTENSENE<br>IKKNIELCVITGLPAKY<br>IDPESKKPFANLEAYKI

NP_001285823.1	DmYL1	D. melanogas	SWC2	MAASRSRRNNAGNK<br>IAHLLNEEEEDDFYKT<br>SYGGFQEDEEDKEYE<br>QKDEEEDVVDSDFSI<br>DENDEPVSDQEEAPE<br>KKRKRGVVNTKAYKE<br>TKPAVKKETKATPAL<br>HKKRPGGGVTKRRPR<br>PRFTVLDSGRKSIRTS<br>TAIKTQATKIRLKELD<br>DARKRKKKKVRVEDY<br>MPTQEELLEEAKITEE<br>ENTKSLEKFQKMELE<br>KKKSRPTKRTFSGPTI<br>RYHSLTMPAMRKPT<br>RGANPAVDSKDLAG<br>KCERTFVTIENDFNDK<br>VFQSLFRHKAPPKAS<br>NGICPITRLPARYFDPI<br>TQQPYYSIQAFKILRE<br>AYYMQLEQQGGGSE<br>QPELAKWLEWRKLV<br>KENRLKASAAASKNG<br>DN
NP_001076413.1	DrVps72	D. rerio	SWC2	MSLANSREQRSTAG<br>NRMSKLLDAEEEDEF<br>YKTTYGGFNDESGDD<br>EYHGDHSDTEDEVDS<br>DFDIDEGDEPDSGQE<br>EDAPKRKSRVVTKAY<br>KEPLKVSKPKVKRVSE<br>ELKPERPRVERRTVRD<br>ELQDLGDIRKSVRKST<br>SEHTRKTNERLQERQ<br>QEAPRKRKGAQSERV<br>LTQDELLDEAKLTAES<br>NLRSLENYERLEADKK<br>KQVHKKRRFEGPMIR<br>YHSVLMPLLPDTHLK<br>EENVDVEGLDQDTP<br>QATPTSSSSTQGAGS<br>LCSRTYITFSDDEAFSS<br>AFPSAARCTPTHPVQ<br>EVCPVTHKPALYRDP<br>VTDIPYANARAFRIIR<br>EAYQKYIAAHGFPNA<br>SGSFSANTDASDSPA<br>NLKSARPKAVLKQSA<br>AAT
NP_001258016.1	HsYL1	H. sapiens	SWC2	MSLAGGRAPRKTAG<br>NRLSGLLEAEEEDEFY<br>QTTYGGFTEESGDDE<br>YQGDQSDTEDEVDS<br>DFDIDEGDEPSSDGE<br>AEEPRRKRRVVTKAY<br>KEPLKSLRPRKVNTPA<br>GSSQKAREEKALLPLE<br>LQDDGSDSRKSMRQ<br>STAEHTRQTFLRVQE<br>RQGQSRRRKGPHCE<br>RPLTQEELLREAKITEE<br>LNLRSLETYERLEADK<br>KKQVHKKRKCPGPIIT<br>YHSVTVPLVGEPGPK<br>EENVDIEGLDPAPSVS<br>ALTPHAGTGPVNPPA<br>RCSRTFITFSDDATFE<br>EWFPQGRPPKVPVR<br>EVCPVTHRPALYRDP<br>VTDIPYATARAFKIIRE<br>AYKKYITAHGLPPTAS<br>ALGPGPPPPEPLPGS<br>GPRALRQKIVIK
NP_033362.2	MmYL1	M. musculus	SWC2	MSLAGGRAPRKTAG<br>NRLSGLLEAEEEDEFY<br>QTTYGGFTEESGDDE<br>YQGDQSDTEDEVDS<br>DFDIDEGDEPSSDGE<br>AEEPRRKRRVVTKAY<br>KEPLKSLRPRKVSTPA<br>SSSQKAREEKTLLPLEL<br>QDDGSDSRKSMRQS<br>TAEHTRQTFLRVQER<br>QGQSRRRKGPHCER<br>PLTQEELLREAKITEEL<br>NLRSLETYERLEADKK<br>KQVHKKRKCPGPIITY<br>HSVTVPLVGEPGPKE<br>ENVDVEGLDPAPTAS<br>ALAPHAGTGTGAAA<br>ATPPAHCSRTFITFSD<br>DATFEEWFPQGRPP<br>KVPVREVCPVTHRPA<br>LYRDPVTDIPYATARA<br>FKIIREAYKKYITAHGL<br>PPTASALGPGPPPPE<br>PLPGSGPRALRQKIVI<br>K

XP_024394407.1	PpaSWC2	P. patens	SWC2	RLSKLLDEEIEADEEF<br>WNQDAFKEDVADDE<br>YEEEQELADEFDSDF<br>NDDEDESGDEEVEEK<br>ERRPKKKQILPPGGKK<br>PLKKGPSGSTKKKGV<br>SFLEEAAAAADAGET<br>PLPTLKTPRQATRPSP<br>EKEDGETEEGEKLLRK<br>STRTSVIVRQAEREA<br>QRAIQQALPKIVKKK<br>REGEDRKMTQEEML<br>LEAAQTEIQNRQSLET<br>LLAREEEVKRKAIINKE<br>VYSGPLIRFYSKEGIN<br>VLEFVKMPEVPEVIN<br>AKAPPCTFTFRNPKQ<br>PVCVVTGLLAKYRDP<br>KSGFPYATKEAFAIIRE<br>RQRKGEDISRKDSLEK<br>VQRKRTKVEREKFAP<br>GTRTGAVKIKYKKPD<br>KPVETKFQNSPKPQS<br>QPSVENRASGGAGP<br>SGATSSGLGTLDKPE<br>NSNKVGEFEDKLDHT<br>STPAPAPAPLPTPMV<br>EANEADFMLEDLGVS
-	PpSWC2	P. polycephal	SWC2	MSTRSLPSRSTRGKR<br>MNALVGDDALVDEE<br>FWKMDMFKEDEED<br>DEYKSEAEEEDIIDAD<br>FDNPEEAESEEEVEVA<br>KEKKEKKRMAYVDPK<br>AKKGPKRRKIQDKAK<br>ALDAELLKGPESDVEV<br>IMDEEPNEEKGEAPK<br>AEPKKGRGGRRKKTP<br>KALEIPEGEEGADAAS<br>PPSTPGSVPNTPKSTR<br>KSTRAVAIQRAEQRE<br>KEREETEKKRSQRSRK<br>RSPVVVRRITQEELLE<br>EAKMTEIYNTESLKYIL<br>SMEEEKKKIKEAPTIV<br>GPAIKYYSKDGKTCVC<br>FDELPDVLASAPPTPP<br>SREICPITGLPAKYKDP<br>KTGICYANLDAFKTLR<br>TKLLQQEEAETNLKIA<br>QLTALLQESKRKKDQ<br>LQNTPTGVLV
NP_010773.4	ScVps72	S. cerevisiae	SWC2	FIIQTRSRRSNAGNKL<br>QKLLEQELRDIESTKR<br>QISSYKNGNDDEEDEI<br>GLLFQEDEDDEDFEM<br>MAKDDDDEGEEKED<br>ETQSIRKEPSQASSEQ<br>AADDLMFSSSESEDS<br>SNENDEDAEEKEIRR<br>QELLSRKKRNKRLQK<br>GPVVIKKQKPKPKSG<br>EAIPRSHHTHEQLNA<br>ETLLLNTRRTSKRSSV<br>MENTMKVYEKLSKAE<br>KKRKIIQERIRKHKEQ<br>ESQHMLTQEERLRIA<br>KETEKLNILSLDKFKEQ<br>EVWKKENRLALQKR<br>QKQKFQPNETILQFLS<br>TAWLMTPAMELEDR<br>KYWQEQLNKRDKKK<br>KKYPRKPKKNLNLGK<br>QDASDDKKRESEESIK<br>NDGDVNSLGENSSSV<br>HNQKRIEETSTNDTV<br>EGESSPDAAVSRVNS<br>DELKPTALPDVTLDAI<br>ANKQSTVDEAPNSQ<br>PQKNIITNEQKITNVG

XP_001019354.1	TtYL1	T. thermophi	SWC2	KNEEIYNSDQDDQD<br>QTPQNKSHSNNSNQ<br>VGAQSKKGAVQDKS<br>AKNSKVTASKGNQKK<br>KSQEVDSKAPKKGKA<br>ISKGKTNKKGKDKGG<br>AASKKGGKKKVQKG<br>KKGEKAPVKGSATKK<br>KGGAQDKKKADNSA<br>LKKTKKISQGKAEIIED<br>EKEEVFNDESNATPP<br>QYSQTNATSYQSDNI<br>SKLKKKPAAKASNKK<br>TQQTLAASKLKTQKE<br>EQGEKSSSLKAFSSIIK<br>DKKNGKNVPFQPTE<br>NKESEEIEIEPETDEQE<br>SDPNQLKKKLGKSDL<br>NTEHQNGGNQVKD<br>DKKGQSESSLKNRSK<br>VLVDSKKPNSKLYKN<br>DNGHEQSTTSKGSKV<br>SAIKNIRKDSLDMELD<br>NTLENEEDEEDILSGS<br>EDSENSQNSDNIKTN<br>KKQLDKKKSDSLSSKL<br>KNKADESEDDGEGED<br>NEEDDEVDDDEEDEE
NP_001085907.1	XlVps72	X. laevis	SWC2	MNLADGRAPRKTAG<br>NRMSGLLQAEEEDDF<br>YKTTYGGFNEESGDE<br>EYNEDRSASEDEVDS<br>DFDIDEGDEPTSDHE<br>EDEPKKKRRVVTKAY<br>KEPIQLLKPKPKKPEA<br>PPNTAAKSRPEKPQE<br>PPDDTVDSRKQMRQ<br>STTEHTRQTFLRVKER<br>QIQSKKKKGPHLDRP<br>LTQEELLEEAKITEEINI<br>RSLENYERLEADRKK<br>QVHKKRRCAGPTIRY<br>HSMVMPLITELRMKE<br>ENVDVEGLDHEQTD<br>RTHAGKCSRSFITFSD<br>DETFERFFPRSKRGKF<br>SVRDVCPVTHKPALY<br>RDPITDIPYYNSKAFKI<br>IRDAYKKYITTHGLPN<br>AAMATTMGPSADA<br>AQRNTRQKIIIKQSVP<br>SA
ACG47775.1	ZmVps72	Z. mays	SWC2	MDAGDDEPPVLLDR<br>AARASRGKRITKLLED<br>EVEQDEAFWNQEAL<br>KDEENDDNYVEEPDA<br>GDEFDSDFGEDESET<br>DDEPEKEVRERLPIKK<br>RLMFPGKTLRKTNVK<br>KKKATPKPEDGTKAD<br>KSADKPSSSTQADLP<br>DELEAEKAIRKSSRTS<br>VIVRQAEREAIRAEKE<br>ATAKPIKRKKEGEEKR<br>MTQEEMLLEAAETEI<br>MNMRNLERVLAREE<br>EVKKKAVVHKDTYEG<br>PTIRFFSRDGESRLEFI<br>NGASFGSELCTTSSPY<br>PEKPVCVVTGLPAKY<br>RDPKTGLPYATMEAF<br>KIIRESFLKEEADRKRP<br>NMSNMGELFESITG<br>GHLMPKKRRVEIRSP<br>NMLGGSRHGGRTW<br>RIPAFDMVDED
XP_006855071.1	AtrPolE3	A. trichopoda	PolE3	MEVDELPRSIVKRMV<br>KEKLSYLSKVDDEPSK<br>DVNISNEALSAFSESA<br>RIFIHYLSATANDICQ<br>ESKRQTINAEDVFKAL<br>EEIDFPEFTEPLRVSLE<br>DFRKKKAFKKSKETN<br>KKRKSEEEPESNVIGS<br>EGKGEETEEGGHTEK<br>EDMDEQEEDCGDEG<br>KEDEEE
NP_498421.1	CePolE3	C. elegans	PolE3	MSSNDVDQNDDRIA<br>QMMMPAAIVTRLM<br>KEDNISASKDARDVIA<br>RAAAVFLINLSDVSA<br>QSARDQKHKTISGDD<br>VVKGLRELENTSIHN<br>HCKSANDKWKVISQ<br>QKALARKNPVESSSV<br>NAEMDDDIIDDTPVD<br>DSLMN

XP_645298.1	DdPolE3	D. discoideun	PolE3	MSESQDLPGAIVNRIIKASLPEGVLCAKESRLAIAKAAKVWIHYLTAASIDFSSHSGRSTISPKDVFQAIEEIDFENFKPQLEEYLAALKSEKEKEKEKEKEKEKEKEKDSNKDDTKSKDENKSSKKSSSSSKDEK
NP_476646.2	DmChrac-14	D. melanogas	PolE3	MVERIEDLNLPNAVIGRLIKEALPESASVSKEARAAIARAASVFAIFVTSSSTALAHKQNHKTITAKDILQTLTELDFESFVPSLTQDLEVYRKVVKEKKESKASKKDSNTAENANASATATAEEAPE
NP_957095.1	DrPolE3	D. rerio	PolE3	MAERPEDLNLPNAVITRIIKEALPEGVNVSKEARRAISQAASVFVLYATSCANSFAMKAKRKTLNAGDVMSAMEEMEFERFLQPLREALEAYKKGQKGKKEASEQKRKDKEKKNGTDENDKSRDEEEDEHMDDEQDGENEAEEEDVEN
NP_001265184.1	HsPolE3	H. sapiens	PolE3	MAERPEDLNLPNAVITRIIKEALPDGVNISKEARSAISRAASVFVLYATSCANNFAMKGKRKTLNASDVLSAMEEMEFQRFVTPLKEALEAYRREQKGKKEASEQKKKDKDKKTDSEEQDKSRDEDNDEDEERLEEEEQNEEEEVDN
NP_067473.2	MmPolE3	M. musculus	PolE3	MAERPEDLNLPNAVITRIIKEALPDGVNISKEARSAISRAASVFVLYATSCANNFAMKGKRKTLNASDVLSAMEEMEFQRFITPLKEALEAYRRDEKGKKEASEQKKKDKDKKDSEEQDKSREVEEEDEERLDEDDQNEEEEIDN
-	PpPolE3	P. polycephal	PolE3	MSGDKTEPKEEKEKSGEDLELPRAVVQRLIKYSLPDNVHVQQEAKLAIAHAGKVFINYLTACANDYCIQGTRSTISAKDVLNACDELEMPEITKKLKEVLEAYKKEQSEKKEKGKDKEKDEGKSED
NP_010406.3	ScDPB4	S. cerevisiae	PolE3	MPPKGWRKDAQGNYPTTSYIKEQENITIQDLLFPKSTIVNLAREVPQQSGKKLLINKDASLALQRGATVFVNHLLLFAREIAKSQDKKSCSVDDVLSALDHIGHSALKGPVRDKLDEYQAAVEQRKKEKLDSGEVDADGDIDMGEDKENVPVEKVKEHDEIEEQGDALQDVEESSEKKQKTESQDVETRVQNLEQT
XP_018084282.1	XlCHRAC17	X. laevis	PolE3	MAERPEDLNLPNAVVTRIIKEALPEGVNISKEARSAISRAASVFVLYATSCANNFAMKGKRKTLNASDVLAAMEEMEFQRFLTPLKESLEVYRQDQKGKKEATEQKKKDKEKKADSEDQDKSREEENEEEDEKMEEDEVVEEEEVEN

OAO93127.1	AtHSP90.1	A thaliana	HSP90	FQAEINQLLSLIINTFY<br>SNKEIFLRELISNSSDA<br>LDKIRFESLTDKSKLD<br>GQPELFIRLVPDKAN<br>KTLSIIDSGIGMTKAD<br>LVNNLGTIARSGTKEF<br>MEALQAGADVSMIG<br>QFGVGFYSAYLVAEK<br>VVVTTKHNDDEQYV<br>WESQAGGSFTVTRD<br>VDGEPLGRGTKISLFL<br>KDDQLEYLEERRLKDL<br>VKKHSEFISYPIYLWTE<br>KTTEKEISDDEDEDEP<br>KKENEGEVEEVDEEK<br>EKDGKKKKKIKEVSHE<br>WELINKQKPIWLRKP<br>EEITKEEYAAFYKSLTN<br>DWEDHLAVKHFSVE<br>GQLEFKAILFVPKRAP<br>FDLFDTRKKLNNIKLY<br>VRRVFIMDNCEELIPE<br>YLSFVKGVVDSDDLPL<br>NISRETLQQNKILKVI<br>RKNLVKKCIEMFNEIA<br>ENKEDYTKFYEAFSKN<br>LKLGIHEDSQNRGKIA<br>DLLRYHSTKSGDEMT
OAO94685.1	AtHSP90.2	A thaliana	HSP90	QLLSLIINTFYSNKEIFL<br>RELISNSSDALDKIRFE<br>SLTDKSKLDGQPELFI<br>HIIPDKTNNTLTIIDSG<br>IGMTKADLVNNLGTI<br>ARSGTKEFMEALAAG<br>ADVSMIGQFGVGFYS<br>AYLVADKVVVTTKHN<br>DDEQYVWESQAGGS<br>FTVTRDTSGETLGRGT<br>KMVLYLKEDQMEYLE<br>ERRLKDLVKKHSEFISY<br>PISLWIEKTIEKEISDD<br>EEEEEKKDEEGKVEEV<br>DEEKEKEEKKKKKIKE<br>VSHEWDLVNKQKPI<br>WMRKPEEINKEEYAA<br>FYKSLSNDWEEHLAV<br>KHFSVEGQLEFKAILF<br>VPKRAPFDLFDTKKK<br>PNNIKLYVRRVFIMD<br>NCEDIIPEYLGFVKGIV<br>DSEDLPLNISRETLQQ<br>NKILKVIRKNLVKKCL<br>ELFFEIAENKEDYNKF<br>YEAFSKNLKLGIHEDS<br>QNRTKIAELLRYHSTK<br>SGDELTSLKDYVTRM
XP_006828794.1	AtrHSP83	A. trichopoda	HSP90	FAFQAEINQLLSLIINT<br>FYSNKEIFLRELISNSS<br>DALDKIRFESLTDKSKL<br>DAQPELFIRLVPDKV<br>NKTLSIIDSGIGMTKA<br>DLVNNLGTIARSGTK<br>EFMEALQAGADVSM<br>IGQFGVGFYSAYLVAE<br>KVIVTTKHNDDEQYV<br>WESQAGGSFTVTRD<br>VNGEPLGRGTKITLFL<br>KEDQQDYLEERRLKD<br>LVKKHSEFISYPIYLWT<br>EKTTEKEVSDDEDEEV<br>DKEKKEGDVEEVEDD<br>EGKDKKKKKKMKEVS<br>HEWTLVNKQKPIWM<br>RKPEEITKEEYASFYKS<br>LTNDWEDHLALKHFS<br>VEGQLEFKAILFLPKR<br>APFDLFDTRKKMNNI<br>KLYVRRVFIMDNCEE<br>LIPEYLGFVKGVVDSD<br>DLPLNISREMLQQNK<br>ILKVIRKNLVKKCIEMF<br>FEIAENKEDYNKFYEA<br>FSKNIKLGIHEDSQNR<br>AKLADLLRYHSTKSGD

NP_506626.1	CeHSP90	C. elegans	HSP90	QLMSLIINTFYSNKEIY<br>LRELISNASDALDKIRY<br>QALTEPSELDTGKELFI<br>KITPNKEEKTLTIMDT<br>GIGMTKADLVNNLG<br>TIAKSGTKAFMEALQ<br>AGADISMIGQFGVGF<br>YSAFLVADKVVVTSK<br>NNDDDSYQWESSAG<br>GSFVVRPFNDPEVTR<br>GTKIVMHIKEDQIDFL<br>EERKIKEIVKKHSQFIG<br>YPIKLVVEKEREKEVE<br>DEEAVEAKDEEKKEG<br>EVENVADDADKKKTK<br>KIKEKYFEDEELNKTK<br>PIWTRNPDDISNEEY<br>AEFYKSLSNDWEDHL<br>AVKHFSVEGQLEFRA<br>LLFVPQRAPFDLFENK<br>KSKNSIKLYVRRVFIM<br>ENCEELMPEYLNFIKG<br>VVDSEDLPLNISREML<br>QQSKILKVIRKNLVKK<br>CMELIDEVAEDKDNF<br>KKFYEQFGKNLKLGIH<br>EDSTNRKKLSDFLRYS<br>TSAGDEPTSLKEYVSR
XP_647482.1	DdHSP90	D. discoideur	HSP90	NQLMSLIINTFYSNKE<br>VFLRELISNASDALDKI<br>RYQSLTDASVLESKTE<br>LEIKIIPDKTAKTLTLID<br>SGIGMTKTDMVKNL<br>GTIARSGTKNFMEQL<br>QSGAADISMIGQFGV<br>GFYSAYLVADTVIVHS<br>KNNDDEQYVWESSA<br>GGEFTIALDHTEPLGR<br>GTKIVLHMKEDQLDY<br>LDETKIKNLVKKHSEFI<br>QYPISLLTIKEKEVDEE<br>TTAKEGEEESTDAKIE<br>EIEEEKEKKKVKVQEK<br>EWDVLNKTKPLWTR<br>NPSDVTKEEYNSFYKS<br>ISNDWEEPLAVKHFS<br>VEGQLEFKAILFVPKK<br>APFDLFESKKKANNIK<br>LYVKRVFIMDNCADII<br>PEYLNFVRGIVDSEDL<br>PLNISRETLQQNKILT<br>VIRKNLVKKCIELFNEI<br>AENSEDYKKFYEAFSK<br>NLKLGVHEDSQNREK<br>FADLLRYQTSKSGDEL<br>VTLKEYVGRMKEGQ
NP_001261362.1	DmHSP83B	D. melanogas	HSP90	QLMSLIINTFYSNKEIF<br>LRELISNASDALDKIRY<br>ESLTDPSKLDSGKELYI<br>KLIPNKTAGTLTIIDTG<br>IGMTKSDLVNNLGTI<br>AKSGTKAFMEALQA<br>GADISMIGQFGVGFY<br>SAYLVADKVTVTSKN<br>NDDEQYVWESSAGG<br>SFTVRADNSEPLGRG<br>TKIVLYIKEDQTDYLEE<br>SKIKEIVNKHSQFIGYP<br>IKLLVEKEREKEVSDD<br>EADDEKKEGDEKKE<br>METDEPKIEDVGEDE<br>DADKKDKDAKKKKTI<br>KEKYTEDEELNKTKPI<br>WTRNPDDISQEEYGE<br>FYKSLTNDWEDHLAV<br>KHFSVEGQLEFRALLF<br>IPRRTPFDLFENQKKR<br>NNIKLYVRRVFIMDN<br>CEDLIPEYLNFMKGV<br>VDSEDLPLNISREML<br>QQNKVLKVIRKNLVK<br>KTMELIEELTEDKENY<br>KKFYDQFSKNLKLGV<br>HEDSNNRAKLADFLR

NP_571403.1	DrHSP90A	*D. rerio*	HSP90	EVETFAFQAEIAQLM<br>SLIINTFYSNKEIFLREL<br>ISNSSDALDKIRYESLT<br>DPSKLDSCKDLKIELIP<br>DQKERTLTIIDTGIGM<br>TKADLINNLGTIAKSG<br>TKAFMEALQAGADIS<br>MIGQFGVGFYSAYLV<br>AEKVTVITKHNDDEQ<br>YIWESAAGGSFTVKP<br>DFGESIGRGTKVILHL<br>KEDQSEYVEEKRIKEV<br>VKKHSQFIGYPITLYIE<br>KQREKEVDLEEGEKQ<br>EEEEVAAGEDKDKPKI<br>EDLGADEDEDSKDGK<br>NKRKKKVKEKYIDAQ<br>ELNKTKPIWTRNPDD<br>ITNEEYGEFYKSLSND<br>WEDHLAVKHFSVEG<br>QLEFRALLFVPRRAAF<br>DLFENKKKRNNIKLYV<br>RRVFIMDNCEELIPEY<br>LNFIKGVVDSEDLPLN<br>ISREMLQQSKILKVIR<br>KNLVKKCLDLFTELAE<br>DKDNYKKYYEQFSKN<br>IKLGIHEDSQNRKKLS
NP_571385.2	DrHSP90B	*D. rerio*	HSP90	AFQAEIAQLMSLIINT<br>FYSNKEIFLRELVSNAS<br>DALDKIRYESLTDPTK<br>LDSGKDLKIDIIPNVQ<br>ERTLTLIDTGIGMTKA<br>DLINNLGTIAKSGTKA<br>FMEALQAGADISMIG<br>QFGVGFYSAYLVAEK<br>VTVITKHNDDEQYA<br>WESSAGGSFTVKVD<br>HGEPIGRGTKVILHLK<br>EDQTEYIEEKRVKEVV<br>KKHSQFIGYPITLYVEK<br>ERDKEISDDEAEEEKA<br>EKEEKEEEGEDKPKIE<br>DVGSDDEEDTKDKDK<br>KKKKKIKEKYIDQEEL<br>NKTKPIWTRNPDDIS<br>NEEYGEFYKSLTNDW<br>EDHLAVKHFSVEGQL<br>EFRALLFIPRRAPFDLF<br>ENKKKKNNIKLYVRR<br>VFIMDNCEELIPEYLN<br>FIRGVVDSEDLPLNIS<br>REMLQQSKILKVIRKN<br>IVKKCLELFAELAEDK<br>DNYKKFYDAFSKNLKL<br>GIHEDSQNRKKLSELL
NP_001017963.2	HsHSP90A	*H. sapiens*	HSP90	EEVETFAFQAEIAQL<br>MSLIINTFYSNKEIFLR<br>ELISNSSDALDKIRYES<br>LTDPSKLDSGKELHIN<br>LIPNKQDRTLTIVDTG<br>IGMTKADLINNLGTIA<br>KSGTKAFMEALQAG<br>ADISMIGQFGVGFYS<br>AYLVAEKVTVITKHN<br>DDEQYAWESSAGGS<br>FTVRTDTGEPMGRG<br>TKVILHLKEDQTEYLE<br>ERRIKEIVKKHSQFIGY<br>PITLFVEKERDKEVSD<br>DEAEEKEDKEEEKEKE<br>EKESEDKPEIEDVGSD<br>EEEEKKDGDKKKKKKI<br>KEKYIDQEELNKTKPI<br>WTRNPDDITNEEYGE<br>FYKSLTNDWEDHLAV<br>KHFSVEGQLEFRALLF<br>VPRRAPFDLFENRKK<br>KNNIKLYVRRVFIMD<br>NCEELIPEYLNFIRGV<br>VDSEDLPLNISREML<br>QQSKILKVIRKNLVKK<br>CLELFTELAEDKENYK<br>KFYEQFSKNIKLGIHE

NP_001258898.1	HsHSP90B	H. sapiens	HSP90	AFQAEIAQLMSLIINT<br>FYSNKEIFLRELISNAS<br>DALDKIRYESLTDPSK<br>LDSGKELKIDIIPNPQE<br>RTLTLVDTGIGMTKA<br>DLINNLGTIAKSGTKA<br>FMEALQAGADISMIG<br>QFGVGFYSAYLVAEK<br>VVVITKHNDDEQYA<br>WESSAGGSFTVRAD<br>HGEPIGRGTKVILHLK<br>EDQTEYLEERRVKEVV<br>KKHSQFIGYPITLYLEK<br>EREKEISDDEAEEEKG<br>EKEEEDKDDEEKPKIE<br>DVGSDEEDDSGKDKK<br>KKTKKIKEKYIDQEEL<br>NKTKPIWTRNPDDIT<br>QEEYGEFYKSLTNDW<br>EDHLAVKHFSVEGQL<br>EFRALLFIPRRAPFDLF<br>ENKKKKNNIKLYVRR<br>VFIMDSCDELIPEYLN<br>FIRGVVDSEDLPLNIS<br>REMLQQSKILKVIRKN<br>IVKKCLELFSELAEDKE<br>NYKKFYEAFSKNLKLG<br>IHEDSTNRRRLSELLR
NP_034610.1	MmHSP90A	M. musculus	HSP90	EEVETFAFQAEIAQL<br>MSLIINTFYSNKEIFLR<br>ELISNSSDALDKIRYES<br>LTDPSKLDSGKELHIN<br>LIPSKQDRTLTIVDTGI<br>GMTKADLINNLGTIA<br>KSGTKAFMEALQAG<br>ADISMIGQFGVGFYS<br>AYLVAEKVTVITKHN<br>DDEQYAWESSAGGS<br>FTVRTDTGEPMGRG<br>TKVILHLKEDQTEYLE<br>ERRIKEIVKKHSQFIGY<br>PITLFVEKERDKEVSD<br>DEAEEKEEKEEEKEKE<br>EKESDDKPEIEDVGSD<br>EEEEEKKDGDKKKKK<br>KIKEKYIDQEELNKTK<br>PIWTRNPDDITNEEY<br>GEFYKSLTNDWEEHL<br>AVKHFSVEGQLEFRA<br>LLFVPRRAPFDLFENR<br>KKKNNIKLYVRRVFIM<br>DNCEELIPEYLNFIRG<br>VVDSEDLPLNISREML<br>QQSKILKVIRKNLVKK<br>CLELFTELAEDKENYK<br>KFYEQFSKNIKLGIHE
NP_032328.2	MmHSP90B	M. musculus	HSP90	AFQAEIAQLMSLIINT<br>FYSNKEIFLRELISNAS<br>DALDKIRYESLTDPSK<br>LDSGKELKIDIIPNPQE<br>RTLTLVDTGIGMTKA<br>DLINNLGTIAKSGTKA<br>FMEALQAGADISMIG<br>QFGVGFYSAYLVAEK<br>VVVITKHNDDEQYA<br>WESSAGGSFTVRAD<br>HGEPIGRGTKVILHLK<br>EDQTEYLEERRVKEVV<br>KKHSQFIGYPITLYLEK<br>EREKEISDDEAEEEKG<br>EKEEEDKEDEEKPKIE<br>DVGSDEEDDSGKDKK<br>KKTKKIKEKYIDQEEL<br>NKTKPIWTRNPDDIT<br>QEEYGEFYKSLTNDW<br>EDHLAVKHFSVEGQL<br>EFRALLFIPRRAPFDLF<br>ENKKKKNNIKLYVRR<br>VFIMDSCDELIPEYLN<br>FIRGVVDSEDLPLNIS<br>REMLQQSKILKVIRKN<br>IVKKCLELFSELAEDKE<br>NYKKFYEAFSKNLKLG<br>IHEDSTNRRRLSELLR

XP_024395876.1	PpaHSP80	P. patens	HSP90	AFQAEINQLLSLIINTF<br>YSNKEIFLRELISNSSD<br>ALDKIRFESLTDKSKLD<br>GQPELFIHIVPDKAN<br>NTLSIIDSGIGMTKAD<br>MVNNLGTIARSGTKE<br>FMEALSAGADVSMI<br>GQFGVGFYSAYLVAE<br>KVVVTSKHNDDEQYI<br>WESQAGGSFTITRDT<br>SGEPLGRGTHIKLYLK<br>EDQLEYLEERRLKDLV<br>KKHSEFISYPISLWTEK<br>TTEKEVSDDEDEDDK<br>KDEEGKIEEVDEEKEK<br>DKKKKKVKEISREWTL<br>INKQKPIWMRKPED<br>VTKEEYAAFYKSLTND<br>WEEHLAVKHFSVEG<br>QLEFKSVLFVPKRAPF<br>DLFDSRKKQNNIKLYV<br>RRVFIMDNCEELIPEY<br>LGFVKGVVDSEDLPL<br>NISRETLQQSKILKVIR<br>KNLVKKCMEMFSEIA<br>ENKEDYQKFYEAFSK<br>NLKLGIHEDSQNRSKL<br>ADLLRYHSTKSGDEM
-	PpHSP90	P. polycephal	HSP90	LMSLIINTFYSNKEIFL<br>RELISNASDALDKIRYE<br>SLTDKSKLEGAPELFIH<br>IVPDKENKTLTLIDTGI<br>GMTKADLINNLGTIA<br>RSGTKNFMEHLQSGS<br>ADISMIGQFGVGFYS<br>AYLVADKVTVTSKHN<br>DDEQYIWESSAGGEF<br>FIRRDTGEQLGRGTKI<br>ILHLKDDQLEYLEEKRI<br>KDLVKKHSEFIQYPISL<br>WETKETEKEVSDDEE<br>EVKEGEKPTIEEVDED<br>KEADKKKKKKVKEVT<br>HEWVLLNKQKPIWT<br>RNPADITKEEYAAFYK<br>SISNDWEEHLAVKHF<br>SVEGQLEFRAILFVPK<br>RAPFDMFESRKKLNN<br>IKLYVRRVFIMDDCKD<br>LIPEWLNFIKGIVDSE<br>DLPLNISRETLQQNKI<br>LKVIKKNLVKKCIELFF<br>EIAENAEDFKKFYEAF<br>GKNLKLGIHEDSQNR<br>AKLAELLRYNSTKSGD<br>EPTSLKDYITRMPESQ
NP_015084.1	ScHSP82	S. cerevisiae	HSP90	MSLIINTVYSNKEIFLR<br>ELISNASDALDKIRYKS<br>LSDPKQLETEPDLFIRI<br>TPKPEQKVLEIRDSGI<br>GMTKAELINNLGTIA<br>KSGTKAFMEALSAGA<br>DVSMIGQFGVGFYSL<br>FLVADRVQVISKSND<br>DEQYIWESNAGGSFT<br>VTLDEVNERIGRGTIL<br>RLFLKDDQLEYLEEKRI<br>KEVIKRHSEFVAYPIQ<br>LVVTKEVEKEVPIPEE<br>EKKDEEKKDEEKKDE<br>DDKKPKLEEVDEEEEK<br>KPKTKKVKEEVQEIEE<br>LNKTKPLWTRNPSDI<br>TQEEYNAFYKSISND<br>WEDPLYVKHFSVEG<br>QLEFRAILFIPKRAPFD<br>LFESKKKKNNIKLYVR<br>RVFITDEAEDLIPEWL<br>SFVKGVVDSEDLPLNL<br>SREMLQQNKIMKVIR<br>KNIVKKLIEAFNEIAED<br>SEQFEKFYSAFSKNIKL<br>GVHEDTQNRAALAK<br>LLRYNSTKSVDELTSLT

AF151114_1	TtHSP82	T. thermophi	HSP90	INTFYSNKEIFLRELIS<br>NASDALDKIRYISITDS<br>EKAKLEVEPNFRIRIIP<br>DKANNTLTLWDTGIG<br>MTKKELINNLGTIAKS<br>GTKAFMEALSSGADI<br>SMIGQFGVGFYSAYL<br>VAEKVEVISKSNDDES<br>QWRWESSAGGTFTV<br>VNDDENPEKLTRGTK<br>IILHMKNDNLEFLEER<br>RIKDLIKKHSEFIAFPIE<br>LQVEKTEEKEETDEED<br>EEKEKEDKEKTDEPEI<br>KEETEKKDKKKKKVKV<br>VHTEFEEQNKNKPL<br>WMRKPEEITKEEYVN<br>FYKSLTNDWEEHQA<br>VKQFSVEGQLEFRAIL<br>FIPKRAPFDLFETKKK<br>KNNIKLYVRRVFIMD<br>DCEELIPEYLNFIKGVV<br>DSEDLPLNISREFLQH<br>NKILKVIKKNIVKKCLD<br>LIQEVADNEEDFKKFY<br>EQFGKNLKLGIHEDS<br>ANREKLSSFLRYHSSK<br>SGEELTTLKDYVSRM
XP_018086052.1	XlHSP90A	X. laevis	HSP90	QDQQMEEDVETFAF<br>QAEIAQLMSLIINTFY<br>SNKEIFLRELISNSSDA<br>LDKIRYESLTDPSKLDS<br>GKELKIELIPNKQDRS<br>LTIIDTGIGMTKADLI<br>NNLGTIAKSGTKAFM<br>EALQAGADISMIGQF<br>GVGFYSAYLVAEKVT<br>VITKHIDDEQYAWES<br>SAGGSFTVRVDNSEP<br>LGRGTKVILHLKEDQS<br>EYFEEKRIKEIVKKHSQ<br>FIGYPITLFVEKERDKE<br>ISDDEAEEEKEEKKDE<br>PKDEEKPEIEDVGSDD<br>EEDKKEGDKKKKKKIK<br>EKYIDQEELNKTKPIW<br>TRNPDDITNEEYGEFY<br>KSLTNDWEDHLAVK<br>HFSVEGQLEFRALLFV<br>PRRAPFDLFENRKKK<br>NNIKLYVRRVFIMDN<br>CDELIPEYLNFMRGV<br>VDSEDLPLNISREML<br>QQSKILKVIRKNLVKK<br>CLELFTELSEDKENYK<br>MFYEHFSKNIKLGIHE
XP_041418547.1	XlHSP90B	X. laevis	HSP90	AFQAEIAQLMSLIINT<br>FYSNKEIFLRELISNAS<br>DALDKIRYESLTDPSK<br>LDSGKDLKIDIIPNRQ<br>ERTLTVIDTGIGMTKA<br>DLINNLGTIAKSGTKA<br>FMEALQAGADISMIG<br>QFGVGFYSAYLVAEK<br>VVVITKHNDDEQYV<br>WESSAGGSFTVKIDH<br>GEPIGRGTKVILHLKE<br>DQTEYLEEKRVKETVK<br>KHSQFIGYPITLYLEKE<br>REKEISDDEAEEEEKE<br>EEKKEEEGENEKPKIE<br>DVGSDDEEEGKDKKK<br>KTKKIKEKYIDQEELN<br>KTKPIWTRNPDDITQ<br>EEYGEFYKSLTNDWE<br>DHLAVKHFSVEGQLE<br>FRALLFIPRRAPFDLFE<br>NKKKKNNIKLYVRRV<br>FIMDSCDELIPEYLNFI<br>RGVVDSEDLPLNISRE<br>MLQQSKILKVIRKNIV<br>KKCLELFSELAEDKEN<br>YKKFYEAFSKNLKLGI<br>HEDSTNRKRLSELLRY

NP_001135416.3	ZmHSP82	Z. mays	HSP90	TETFAFQAEINQLLSLI<br>INTFYSNKEIFLRELIS<br>NASDALDKIRFESLTD<br>KSKLDAQPELFIRLVP<br>DKASKTLSIIDSGVGM<br>TKSDLVNNLGTIARSG<br>TKEFMEALAAGATDV<br>SMIGQFGVGFYSAYL<br>VADRVMVTTKHNDD<br>EQYVWESQAGGSFT<br>VTHDTTGERLGRGTK<br>ITLFLKDDQLEYLEERR<br>LKDLVKKHSEFISYPIY<br>LWTEKTTEKEISDDEE<br>EDNKKEEEGDVEEVD<br>DEDKDAKDKSKKKKK<br>VKEVSHEWVQINKQ<br>KPIWLRKPEEITREEY<br>ASFYKSLTNDWEDHL<br>AVKHFSVEGQLEFKAI<br>LFVPRRAPFDLFDTRK<br>KLNNIKLYVRRVFIMD<br>NCEELIPEWLGFVKG<br>VVDSDDLPLNISRETL<br>QQNKILKVIRKNLVKK<br>CIEMFFEIAENKDDYA<br>KFYDAFSKNIKLGIHE<br>DSQNRAKLADLLRYH
NP_195870.1	AtHSP70-1	A thaliana	HSC70	TTYSCVGVWQHDRV<br>EIIANDQGNRTTPSYV<br>AFTDSERLIGDAAKN<br>QVAMNPVNTVFDAK<br>RLIGRRFSDSSVQSD<br>MKLWPFKIQAGPAD<br>KPMIYVEYKGEEKEFA<br>AEEISSMVLIKMREIA<br>EAYLGVTIKNAVVTVP<br>AYFNDSQRQATKDA<br>GVIAGLNVMRIINEPT<br>AAAIAYGLDKKATSV<br>GEKNVLIFDLGGGTF<br>DVSLLTIEEGIFEVKAT<br>AGDTHLGGEDFDNR<br>MVNHFVQEFKRKSK<br>KDITGNPRALRRLRTS<br>CERAKRTLSSTAQTTI<br>EIDSLYEGIDFYSTITR<br>ARFEELNMDLFRKC<br>MEPVEKCLRDAKMD<br>KSTVHDVVLVGGSTR<br>IPKVQQLLQDFFNGK<br>ELCKSINPDEAVAYG<br>AAVQGAILSGEGNEK<br>VQDLLLLDVTPLSLGL<br>ETAGGVMTTLIPRNT<br>TIPTKKEQVFSTYSDN
XP_006833219.1	AtrHSC70	A. trichopoda	HSC70	TTYSCVGVWQHDRV<br>EIIANDQGNRTTPSYV<br>AFTDTERLIGDAAKN<br>QVAMNPVNTVFDAK<br>RLIGRRFSDASVQSDI<br>KLWPFKVIPGPGDKP<br>MIVVQYKGEDKQFA<br>AEEISSMVLMKMREI<br>AEAYLGSAVKNAVVT<br>VPAYFNDSQRQATK<br>DAGVIAGLNVMRIIN<br>EPTAAAIAYGLDKKAT<br>SVGEKNVLIFDLGGG<br>TFDVSLLTIEEGIFEVK<br>STAGDTHLGGEDFDN<br>RMVNHFVQEFKRKH<br>KKDISGNPRALRRLRT<br>SCERAKRTLSSTAQTT<br>IEIDSLYEGIDFYSTITR<br>ARFEELNMDLFRKC<br>MEPVEKCLRDAKMD<br>KNTIHDVVLVGGSTRI<br>PRVQQLLQDFFNGKE<br>LCKSINPDEAVAYGA<br>AVQAAILSGEGNEKV<br>QDLLLLDVTPLSLGLE<br>TAGGVMTVLIPRNTT<br>IPTKKEQVFSTYSDNQ

NP_503068.1	CeHSP-1	C. elegans	HSC70	SCVGVFMHGKVEIIA<br>NDQGNRTTPSYVAFT<br>DTERLIGDAAKNQVA<br>MNPHNTVFDAKRLI<br>GRKFDDPAVQSDMK<br>HWPFKVISAEGAKPK<br>VQVEYKGENKIFTPEE<br>ISSMVLLKMKETAEA<br>FLGTTVKDAVVTVPA<br>YFNDSQRQATKDAG<br>AIAGLNVLRIINEPTA<br>AAIAYGLDKKGHGER<br>NVLIFDLGGGTFDVSI<br>LTIEDGIFEVKSTAGD<br>THLGGEDFDNRMVN<br>HFCAEFKRKHKKDLA<br>SNPRALRRLRTACER<br>AKRTLSSSSQASIEIDS<br>LFEGIDFYTNITRARFE<br>ELCADLFRSTMDPVE<br>KSLRDAKMDKSQVH<br>DIVLVGGSTRIPKVQK<br>LLSDLFSGKELNKSINP<br>DEAVAYGAAVQAAIL<br>SGDKSEAVQDLLLLD<br>VAPLSLGIETAGGVM<br>TALIKRNTTIPTKTAQ<br>TFTTYSDNQPGVLIQ
XP_646617.1	DdHSC70	D. discoideur	HSC70	VWQNDRVEIIANDQ<br>GNRTTPSYVAFTDTE<br>RLIGDAAKNQVAMN<br>PTNTVFDAKRLIGRKF<br>SDKEVQSDMKHWPF<br>KVIPKDGDKPHIQVEF<br>KGETKVFSPEEISSMV<br>LLKMKETAEAYLGKTI<br>NNAVITVPAYFNDSQ<br>RQATKDAGTISKLNV<br>QRIINEPTAAAIAYGL<br>EKKGSGEKNILIFDLG<br>GGTFDVSLLTIEDGVF<br>EVKATAGDTHLGGED<br>FDNRLVSHFVDEFKR<br>KHKKDIMGNQRAVR<br>RLRTACERAKRTLSSS<br>AQASIEIDSLFEGIDFY<br>TSITRARFEELCADLFR<br>GCLDPVEKVLKDSKL<br>DKKSIHEIVLVGGSTRI<br>PKVQQLLQEFFNGKE<br>LNKSINPDEAVAYGA<br>AVQAAILSNEGGAKV<br>ADLLLLDVAPLSMGL<br>ETAGGVMTTLIPRNT<br>TIPCKKTQTFSTYSDN<br>QPGVLIQVYEGERAM
AAG26909.1	DmHSP70Bb	D. melanogas	HSC70	GVYQHGKVEIIANDQ<br>GNRTTPSYVAFTDSE<br>RLIGDPAKNQVAMN<br>PRNTVFDAKRLIGRKY<br>DDPKIAEDMKHWPF<br>KVVSDGGKPKIGVEY<br>KGESKRFAPEEISSMV<br>LTKMKETAEAYLGESI<br>TDAVITVPAYFNDSQ<br>RQATKDAGHIAGLN<br>VLRIINEPTAAALAYG<br>LDKNLNGERNVLIFDL<br>GGGTFDVSILTIDEGS<br>LFEVRSTAGDTHLGG<br>EDFDNRLVTHLAEEF<br>KRKYKKDLRSNPRAL<br>RRLRTAAERAKRTLSS<br>STEATIEIDALFEGQD<br>FYTKVSRARFEELCAD<br>LFRNTLQPVEKALND<br>AKMDKGQIHDIVLVG<br>GSTRIPKVQSLLQEFF<br>HGKNLNLSINPDEAV<br>AYGAAVQAAILSGDQ<br>SGKIQDVLLVDVAPLS<br>LGIETAGGVMTKLIER<br>NCRIPCKQTKTFSTYS<br>DNQPGVSIQVYEGER

NP_001103873.1	DrHSPA8	D. rerio	HSC70	SCVGVFQHGKVEIIA<br>NDQGNRTTPSYVAFT<br>DTERLIGDAAKNQVA<br>MNPTNTVFDAKRLIG<br>RRFDDGVVQSDMKH<br>WPFNVINDNSRPKV<br>QVEYKGESKSFYPEEI<br>SSMVLTKMKEIAEAY<br>LGKTVSNAVITVPAYF<br>NDSQRQATKDAGTIS<br>GLNVLRIINEPTAAAI<br>AYGLDKKVGAERNVL<br>IFDLGGGTFDVSILTIE<br>DGIFEVKSTAGDTHL<br>GGEDFDNRMVNHFI<br>TEFKRKHKKDISDNKR<br>AVRRLRTACERAKRT<br>LSSSTQASIEIDSLYEGI<br>DFYTSITRARFEELNA<br>DLFRGTLDPVEKALR<br>DAKMDKAQIHDIVLV<br>GGSTRIPKIQKLLQDY<br>FNGKELNKSINPDEA<br>VAYGAAVQAAILSGD<br>KSENVQDLLLLDVTPL<br>SLGIETAGGVMTVLIK<br>RNTTIPTKQTQTFTTY<br>SDNQPGVLIQVYEGE
NP_006588.1	HsHSC70	H. sapiens	HSC70	SCVGVFQHGKVEIIA<br>NDQGNRTTPSYVAFT<br>DTERLIGDAAKNQVA<br>MNPTNTVFDAKRLIG<br>RRFDDAVVQSDMKH<br>WPFMVVNDAGRPK<br>VQVEYKGETKSFYPEE<br>VSSMVLTKMKEIAEA<br>YLGKTVTNAVVTVPA<br>YFNDSQRQATKDAG<br>TIAGLNVLRIINEPTA<br>AAIAYGLDKKVGAER<br>NVLIFDLGGGTFDVSI<br>LTIEDGIFEVKSTAGD<br>THLGGEDFDNRMVN<br>HFIAEFKRKHKKDISE<br>NKRAVRRLRTACERA<br>KRTLSSSTQASIEIDSL<br>YEGIDFYTSITRARFEE<br>LNADLFRGTLDPVEK<br>ALRDAKLDKSQIHDIV<br>LVGGSTRIPKIQKLLQ<br>DFFNGKELNKSINPD<br>EAVAYGAAVQAAILS<br>GDKSENVQDLLLLDV<br>TPLSLGIETAGGVMT<br>VLIKRNTTIPTKQTQT<br>FTTYSDNQPGVLIQV
NP_112442.2	MmHSC70	M. musculus	HSC70	SCVGVFQHGKVEIIA<br>NDQGNRTTPSYVAFT<br>DTERLIGDAAKNQVA<br>MNPTNTVFDAKRLIG<br>RRFDDAVVQSDMKH<br>WPFMVVNDAGRPK<br>VQVEYKGETKSFYPEE<br>VSSMVLTKMKEIAEA<br>YLGKTVTNAVVTVPA<br>YFNDSQRQATKDAG<br>TIAGLNVLRIINEPTA<br>AAIAYGLDKKVGAER<br>NVLIFDLGGGTFDVSI<br>LTIEDGIFEVKSTAGD<br>THLGGEDFDNRMVN<br>HFIAEFKRKHKKDISE<br>NKRAVRRLRTACERA<br>KRTLSSSTQASIEIDSL<br>YEGIDFYTSITRARFEE<br>LNADLFRGTLDPVEK<br>ALRDAKLDKSQIHDIV<br>LVGGSTRIPKIQKLLQ<br>DFFNGKELNKSINPD<br>EAVAYGAAVQAAILS<br>GDKSENVQDLLLLDV<br>TPLSLGIETAGGVMT<br>VLIKRNTTIPTKQTQT<br>FTTYSDNQPGVLIQV

XP_024379975.1	PpaHSC70	P. patens	HSC70	TTYSCVGVWQHDRV<br>EIIANDQGNRTTPSYV<br>AFTDTERLIGDAAKN<br>QVAMNPTNTVFDAK<br>RLIGRRFSDASVQSD<br>MKLWPFKITPGPGEK<br>PMISVQYKGEEKTFA<br>AEEVSSMVLIKMKEIA<br>EAYLGSTVKNAVVTV<br>PAYFNDSQRQATKD<br>AGVIAGLNVLRIINEP<br>TAAAIAYGLDKKATSV<br>GEKNVLIFDLGGGTF<br>DVSILTIEEGIFEVKAT<br>AGDTHLGGEDFDNR<br>MVNHFVQEFKRKYK<br>KDISSNPRALRRLRTA<br>AERAKRTLSSTAQTTI<br>EIDSLYEGVDFYSTITR<br>ARFEELNMDMFRKC<br>MEPVEKSLRDAKMD<br>KSSIHDVVLVGGSTRI<br>PKVQQLLQDFFNGKE<br>LCKSINPDEAVAYGA<br>AVQAAILSGEGNEKV<br>QDLLLLDVTPLSLGLE<br>TAGGVMTVLIARNTT<br>IPTKKEQVFSTYSDNQ
-	PpHSC70	P. polycephal	HSC70	SCVGVWQNDRVEIIA<br>NDQGNRTTPSYVAFT<br>DQERLIGDAAKNQV<br>ALNPENTVFDAKRLI<br>GRRFSDPVCQDDMK<br>HWPFKVIAKEGDKPH<br>IRVTFKGEQKDFSPEE<br>ISAMVLIKMKETAEAF<br>LGATVTNAVITVPAYF<br>NDSQRQATKDAGTIS<br>GLNVLRIINEPTAAAI<br>AYGLDKTAEAEKNVLI<br>FDLGGGTFDVSLLTIE<br>SGVFEVKSTGGNTHL<br>GGEDFDNRLVNHFM<br>EEFKRKFKKDITGNQR<br>AIRRLRTACERAKRTL<br>SSSTQASIEIDSLHEGV<br>DFYTNITRARFEELCID<br>LFRGCLEPVEKVLRDA<br>KLSKSQVHEIVLVGGS<br>TRIPKVQQLLQDFFN<br>GKELNKSINPDEAVA<br>YGAAVQAAILTGEGG<br>KKVQDLLLLDVTPLTL<br>GIETAGGVMTSLISR<br>NSTIPCKKAQVFSTYS<br>DNQPGVLIQVYEGER
NP_009396.2	ScSSA1	S. cerevisiae	HSC70	VAHFANDRVDIIAND<br>QGNRTTPSFVAFTDT<br>ERLIGDAAKNQAAM<br>NPSNTVFDAKRLIGR<br>NFNDPEVQADMKHF<br>PFKLIDVDGKPQIQVE<br>FKGETKNFTPEQISS<br>MVLGKMKETAESYLG<br>AKVNDAVVTVPAYF<br>NDSQRQATKDAGTIA<br>GLNVLRIINEPTAAAI<br>AYGLDKKGKEEHVLIF<br>DLGGGTFDVSLLSIED<br>GIFEVKATAGDTHLG<br>GEDFDNRLVNHFIQE<br>FKRKNKKDLSTNQRA<br>LRRLRTACERAKRTLS<br>SSAQTSVEIDSLFEGID<br>FYTSITRARFEELCADL<br>FRSTLDPVEKVLRDAK<br>LDKSQVDEIVLVGGST<br>RIPKVQKLVTDYFNG<br>KEPNRSINPDEAVAY<br>GAAVQAAILTGDESS<br>KTQDLLLLDVAPLSLG<br>IETAGGVMTKLIPRNS<br>TIPTKKSEIFSTYADN<br>QPGVLIQVFEGERAK

NP_013076.1	ScSSA2	S. cerevisiae	HSC70	VAHFSNDRVDIIAND<br>QGNRTTPSFVGFTDT<br>ERLIGDAAKNQAAM<br>NPANTVFDAKRLIGR<br>NFNDPEVQGDMKHF<br>PFKLIDVDGKPQIQVE<br>FKGETKNFTPEQISS<br>MVLGKMKETAESYLG<br>AKVNDAVVTVPAYF<br>NDSQRQATKDAGTIA<br>GLNVLRIINEPTAAAI<br>AYGLDKKGKEEHVLIF<br>DLGGGTFDVSLLSIED<br>GIFEVKATAGDTHLG<br>GEDFDNRLVNHFIQE<br>FKRKNKKDLSTNQRA<br>LRRLRTACERAKRTLS<br>SSAQTSVEIDSLFEGID<br>FYTSITRARFEELCADL<br>FRSTLDPVEKVLRDAK<br>LDKSQVDEIVLVGGST<br>RIPKVQKLVTDYFNG<br>KEPNRSINPDEAVAY<br>GAAVQAAILTGDESS<br>KTQDLLLLDVAPLSLG<br>IETAGGVMTKLIPRNS<br>TIPTKKSEVFSTYADN<br>QPGVLIQVFEGERAK
NP_009478.1	ScSSA3	S. cerevisiae	HSC70	VAHFSNDRVEIIAND<br>QGNRTTPSYVAFTDT<br>ERLIGDAAKNQAAIN<br>PHNTVFDAKRLIGRK<br>FDDPEVTTDAKHFPF<br>KVISRDGKPVVQVEY<br>KGETKTFTPEEISSMV<br>LSKMKETAENYLGTT<br>VNDAVVTVPAYFNDS<br>QRQATKDAGTIAGM<br>NVLRIINEPTAAAIAY<br>GLDKKGRAEHNVLIF<br>DLGGGTFDVSLLSIDE<br>GVFEVKATAGDTHLG<br>GEDFDNRLVNHLATE<br>FKRKTKKDISNNQRSL<br>RRLRTAAERAKRALSS<br>SSQTSIEIDSLFEGMD<br>FYTSLTRARFEELCAD<br>LFRSTLEPVEKVLKDS<br>KLDKSQIDEIVLVGGS<br>TRIPKIQKLVSDFFNG<br>KEPNRSINPDEAVAY<br>GAAVQAAILTGDQST<br>KTQDLLLLDVAPLSLG<br>IETAGGIMTKLIPRNS<br>TIPTKKSETFSTYADN<br>QPGVLIQVFEGERTR
NP_011029.3	ScSSA4	S. cerevisiae	HSC70	VAHFANDRVEIIAND<br>QGNRTTPSYVAFTDT<br>ERLIGDAAKNQAAM<br>NPHNTVFDAKRLIGR<br>KFDDPEVTNDAKHYP<br>FKVIDKGGKPVVQVE<br>YKGETKTFTPEEISSMI<br>LTKMKETAENFLGTE<br>VKDAVVTVPAYFNDS<br>QRQATKDAGTIAGLN<br>VLRIINEPTAAAIAYGL<br>DKKSQKEHNVLIFDL<br>GGGTFDVSLLSIDEGV<br>FEVKATAGDTHLGGE<br>DFDSRLVNFLAEEFKR<br>KNKKDLTTNQRSLRR<br>LRTAAERAKRTLSSSA<br>QTSIEIDSLFEGIDFYT<br>SITRARFEELCADLFRS<br>TLEPVEKVLADSKLDK<br>SQIDEIVLVGGSTRIPK<br>VQKLVSDFFNGKEPN<br>RSINPDEAVAYGAAV<br>QAAILTGDQSSTTQD<br>LLLLDVAPLSLGIETAG<br>GIMTKLIPRNSTIPTK<br>KSEVFSTYADNQPGV<br>LIQVFEGERTRTKDN

XP_001012263.1	TtHSC70	T. thermophi	HSC70	LGTTYSCVGVFINDRV<br>EIIANDQGNRTTPSYV<br>AFTETERLIGDAAKN<br>QVARNPTNTVFDAK<br>RLIGRKFNETVVQKDI<br>KLWPFKVEAGPDDK<br>PKIVVKHKGEVKKFH<br>AEEISSMVLVKMREIA<br>EAFLTKQIKNAVITVP<br>AYFNDSQRQATKDA<br>GAIAGLNVLRIINEPT<br>AAAIAYGLDKKGQGE<br>KNVLIFDLGGGTFDVS<br>LLTLDDGIFEVKATAG<br>DTHLGGEDFDNKLVE<br>FCAADFLKKKNIDIRE<br>NPRAMRRLRTQCER<br>AKRILSSSAQATIEVD<br>ALADSEDFMMVISRP<br>KFEELCLSMFKECIPP<br>VEKVLKDSGMAKNQ<br>VHEVVLVGGSTRIPK<br>VIQLITEFFNGKEPNR<br>SINPDEAVAYGAAIQ<br>AAILTGSGSEHIQDVL<br>LLDVTPLSMGIETAG<br>QVMTVLIPRNTTIPTK<br>KSQVFTTYADNQPGV
NP_001079632.1	XLHSAP8.L	X. laevis	HSC70	SCVGVFQHGKVEIIA<br>NDQGNRTTPSYVAFT<br>DTERLIGDAAKNQVA<br>MNPTNTVFDAKRLIG<br>RRFEDAVVQSDMKH<br>WPFTVVSDGGRPKV<br>QVEYKAETKSFYPEEV<br>SSMVLTKMKEIAEAY<br>LGKTVTNAVVTVPAY<br>FNDSQRQATKDAGTI<br>SGLNVLRIINEPTAAAI<br>AYGLDKKVGVERNVL<br>IFDLGGGTFDVSILTIE<br>DGIFEVKSTAGDTHL<br>GGEDFDNRMVNHFV<br>AEFKRKHKKDIIDNKR<br>AVRRLRTACERAKRT<br>LSSSTQASIEIDSLYEGI<br>DFYTSITRARFEELNA<br>DLFRGTLDPVEKSLRD<br>AKLDKSQIHDIVLVGG<br>STRIPKIQKLLQDFFN<br>GKELNKSINPDEAVA<br>YGAAVQAAILSGDKS<br>ENVQDLLLLDVTPLSL<br>GIETAGGVMTVLIKR<br>NTTIPTKQTQTFTTYS<br>DNQPGVLIQVYEGER
NP_001148198.1	ZmHSC70	Z. mays	HSC70	GTTYSCVGVWQHDR<br>VEIIANDQGNRTTPSY<br>VAFTDSERLVGDAAK<br>NQVAMNPINTVFDA<br>KRLIGRRFSDASVQSD<br>AKLWPFKVIPGSGDK<br>PMIGVQFRGEEKQFS<br>AEEISSMILNKMKETA<br>EAYLGTTIKNAVVTVP<br>AYFNDSQRQATKDA<br>GVISGLNVMRIINEPT<br>AAAIAYGLDKKSSSD<br>GEKNVLIFDLGGGTF<br>DVSLLTIEEGIFEVKAT<br>AGDTHLGGEDFDNR<br>LVNHFVQEFKRKNKK<br>DITGNPRALRRLRTAC<br>ERAKRTLSSTAQTTIEI<br>DSLYEGIDFYTTITRAR<br>FEELNMDLFRKCMEP<br>VEKCLRDAKMDKSSV<br>HDVVLVGGSTRIPRV<br>QQLLQDFFNGKELCK<br>SINPDEAVAYGAAVQ<br>AAILTGEGNEKVQDL<br>LLLDVSPLSLGLETAG<br>GVMTVLIPRNTTIPTK<br>KEQVFSTYSDNQPGV

NP_176725.1	AtFAS1	A thaliana	CAF1A	TMIEPKKLNKRKREPT<br>AIENLTSEEKESQISSL<br>NLEMKGLFDYFREV<br>MDKSKRTDLFSGFSE<br>CSSLNSMVALLMEE<br>MSLPLSKLVDEIYLKLK<br>EKTESVTMVAVKSAV<br>VSVGQRVSYGVLNV<br>DADVLEDDSESCLWC<br>WETRDLKIMPSSVRG<br>VLKLRRTCRKKIHERIT<br>AVSAMLAALQREETE<br>KLWRSDLSKAAEKLG<br>KILSEVDIRSFMDNM<br>MQKNSSEMAEKDSK<br>REEKLLLKQLEKNRCE<br>AEKEKKRMERQVLKE<br>KLQQEKEQKLLQKAI<br>VDENNKEKEETESRK<br>RIKKQQDESEKEQKR<br>REKEQAELKKQLQVQ<br>KQASIMERFLKKSKDS<br>SLTQPKLPSSEVTAQE<br>LSCTKHENEIGKVVQ<br>AIDNAFSTTCEATVD<br>DIRREHFASWRQLGH<br>LLSSSKKHWGMRRQ<br>PKSELFPKLKLSTNSG
XP_006858774.1	AtrFAS1	A. trichopoda	CAF1A	RSPKTRMIEHENPRV<br>SPVAVMAIESHGQP<br>MEKSGVSCDMDCRG<br>LVSPIQSQDLPKRKRK<br>RRSLEGNMSQEEKEL<br>KIAAFREEIQGLLRYY<br>DEFMSEKTDLGVDLN<br>GSMDSVIACLIEESEL<br>PLKKLVDSVLKSLKRK<br>GGEGANITLLSVQNSI<br>HRVGIRNKYGIENPN<br>ADLMEDESKPCLWC<br>WETRELKLLPKAHHG<br>ILKIRRTGRRRIRERIA<br>AVSEMISSLSAQENE<br>NDYKAKIMEASGKLR<br>KILNLERIRDLVSNLLQ<br>KIEAGREVIGEQEQKL<br>LDRELKNMGKELKRQ<br>MEEAEREERQREKEL<br>KRQQDEADKEQRRR<br>EREEAELKKQLKRQQ<br>EDAEKEQRRHEKEEA<br>ESKKQLKKQQEEAEK<br>DKRRREKEEAERKKQ<br>LSVQKQAAMMKRFF<br>KSNSNVNISPTKVSSP<br>RSPHKNEQISNAVILA
NP_492440.1	CeChaf-1	C. elegans	CAF1A	LIETNENSCDPNQKG<br>VKRVAQTPPEKDAKK<br>VKLSRDSSNQSVIELS<br>DTSPQKETSQQSPKA<br>PKTPKTPKTPKVSREE<br>REKQKREKMEEREKQ<br>RIERERILEEKRLEKDK<br>LAEEKRLDKEKKEKER<br>LDKKLEEDKKKEEKRK<br>EAEEKKKKDEEEKMK<br>KDEERNKRKKEEEEKK<br>EAKRREDEEKKEAKR<br>KEEEAIEERKRRQSAL<br>FAKFFSKVEKKKVAEP<br>QKESSNWYLPFERKD<br>GMSLAKIISRDPLPDD<br>ADIFTQHEEINSIASFI<br>ESAAKIVPIESSKARN<br>MKAKLFQFHGNRRP<br>QYYGTWRKKSKIVSG<br>SCPLAEEIGIDYNVVS<br>DDEWEDEPSDGEEC<br>NSDDDAEKDNDDDD<br>GGEEDDGFFVPPCYL<br>SDGEGDEDSTSDNDI<br>AGDKKKEKQPKRITID<br>SDDDENSTDAAERKA<br>RLAQRAEDWAKRTG

XP_637076.1	DdCAF1A	D. discoideur	CAF1A	IKDTEMKEETPNELPK PIPTSTTTTTTTTTTTP TSTTTTTTSTPKRKRLT EEEKQKREDEKKKREE DRIKREEDKKKKDEEK KKLTEEREIEKKKRDEE KKKLLEEKEINRKKREE EAEKKRIEKKKKEEEIE KKKEEIERKKGEAERL KEEEQKKQKKLTSFFS PIEAPIKPQKSNSFIQP IELPSNTQLYQYQLPT YSNSYEVFKESISSSN DTIKLETNQKKRDYFS KLNRGTQHGELGQR NHRFKLKIDSLPGAIM HHDLVSRLSALKLLKF HDSFRPSYYGTFSKTS KQITAKNPFKKDLTID YDYDSSDEWEEEKDD DQAEKITSADEKEED QDADEEENEEDRAW IDDIGLNDDDDTQVD NSTNISVNERVFSRK NKKKRFEEKKPIILEPY FNQYPTIINLNVHNK NNNNNNYNNNNNNN NNNNNNNNNNNNN
NP_572495.1	Dm-p180	D. melanogas	CAF1A	RSQLDGLFRHRDEEA DDEVEEEEQNIVRRK PPNRANLYLSELSSGR RKPLKMQRDVKLQR RTKDEEDDDDVQVID YLSPAGLPIEVEQPKQ LTRMKAKYLHFADNR RPPYYGTWRKKSSSIS ARRPLAQDKVLFDYE VDSDCEWEEEEPGES LSASEDEKEKESEEESE EEYNEWYVPHGHLS DEELQNDGDGMEDG HTREAQKAKLQVLQ QEFAQEMKKQTKKIK ARLLGPVWLDENGN KSELFPAIFAHTIDMY ACWQVEPLSLEPPPE PERQDQTPEQPVPLQ LDDRLMQQLVRLTH GNRNSKTFLINEYLEY LKTQATAEANQTTLP SKAVLREKFDELASW KTVELSTPEAAAASA NSAKKGRKPKKRLC WVVASDMLEKFQLP DLQLQNQWNYTLTP KVSDGPDAAQQEQS
NP_001038478.2	DrChaf1-A	D. rerio	CAF1A	TPRRDMDCVGKANT NKKLVQARLPFKRLN PEPKECNEPKRTKGP VAPKCSEPSDQENDQ DSSSISHHGPALVNG RGPLDCFMSRRKRSP LRSAPEATIDLTEDSN DSAKQQPAPPIAATC PLSEEKTKTSEGTTEPT IPLTEEETEKDEAEDV DALPLLDITQDSDTEE EEEEEEEEEEQQQEAE VSHGNESVLSTGSTSS ASVIASSPEPSKSAPT TPASTSRINAANKVK RRSLKSVQEQEEKQR QRDEKERLKQEAKAA KEKKKEEARKMKEEK EREKKEKKEKDEKERR EKKERDEKEKADKLK AKEEQRQMKIEAKLE EKRKKEEEKRLKEEKD RIKAEKAEITRFLQKP KTQLAPKTLASACGK FAPFEIKAHMSLAPLT RVQCEDSVLEDLDRY LAQPDSTLNGLKDWT GHKPRSSGPTRPRHS

NP_005474.2	Hs-p150	H. sapiens	CAF1A	AATAMDCKDRPAFP<br>VKKLIQARLPFKRLNL<br>VPKGKADDMSDDQ<br>GTSVQSKSPDLEASLD<br>TLENNCHVGSDIDFR<br>PKLVNGKGPLDNFLR<br>NRIETSIGQSTVIIDLT<br>EDSNEQPDSLVDHNK<br>LNSEASPSREAINGQ<br>REDTGDQQGLLKAIQ<br>NDKLAFPGETLSDIPC<br>KTEEEGVGCGGAGR<br>RGDSQECSPRSCPELT<br>SGPRMCPRKEQDSW<br>SEAGGILFKGKVPMV<br>VLQDILAVRPPQIKSL<br>PATPQGKNMTPESE<br>VLESFPEEDSVLSHSSL<br>SSPSSTSSPEGPPAPP<br>KQHSSTSPFPTSTPLR<br>RITKKFVKGSTEKNKL<br>RLQRDQERLGKQLKL<br>RAEREEKEKLKEEAKR<br>AKEEAKKKKEEEKELK<br>EKERREKREKDEKEKA<br>EKQRLKEERRKERQE<br>ALEAKLEEKRKKEEEK<br>RLREEEKRIKAEKAEIT
NP_038761.1	Mm-p150	M. musculus	CAF1A	DCKDRPGFPVKRLIQ<br>ARLPFKRLNLVPKEKV<br>EEDTSPKAAVESKVP<br>DLQLSLGTFESQCHT<br>GSHVGLSTKLVGGQG<br>PIDSFLRATIKPVPSVV<br>IIDLTENCSDIPDSPEG<br>HSELSPDTAGVVTTV<br>EGAAKQQEHSAAELC<br>LLETPSDITCHMEEEP<br>GSPGDPKRTGDCQA<br>GSLQSCPELTPGSRTC<br>PTKELSSWSKAGDLLF<br>IEKVPVVVLEDILATKP<br>SIASLPMMSLDRSVT<br>SESEILESCPEDDSILS<br>HSSTNSSSPTSSPEGP<br>STPPEHRGGRSSPSTP<br>ACRVAKNFVKGSTEK<br>GRSKLHRDREQQREE<br>KEKLREEIRRAKEEAR<br>KKKEEEKELKEKERRE<br>KREKDEKEKAEKQRL<br>KEEKRKERQEALEAKL<br>EEKRKKEEEKRLREEE<br>KRLREEEKRIKAEKAEI<br>TRFFQKPKTPQAPKT<br>LAGSCGKFAPFEIKEH
XP_024369555.1	PpaFAS1	P. patens	CAF1A	PAEREKLKREKAERKA<br>EKLRIRAEKEAARAER<br>ELKKQQEEAEKEQRR<br>KDRELRKKNEEEKKQ<br>LDDVEKEQRRKEKEAI<br>EAEKELKRKERELRRQ<br>QEEVEKEQKRKEKEV<br>AEKKRQLLLQKQATI<br>MDRLFRRKESITPVA<br>HEKQSPAQGVSSPSP<br>AHQTPEGISTSRTEPS<br>TVEMVMRLDEGLKV<br>PCTLTEEEILRGHVSS<br>WQEKWKRRSLEPFR<br>RWGVRMAPKVTVYR<br>ELRLQGASASFPRRVE<br>SAGNAVSSSTLEAQA<br>PSLKRSREDFESIRDC<br>KVEDDCILLNEDPIDS<br>PPRKKQCNTRWKLL<br>QFDKSHRPAYYGTFS<br>KLSSTVGPRHPLRKDP<br>SLDYEVDSDEEWEEE<br>DPGESLSDCEDKEED<br>VEKVDSELDEDAADG<br>FVVPDGYLSENEGVH<br>LEETDVEDTEKAAEVK<br>APCESGTGAECMVS

-	PpCAF1A	P. polycephal	CAF1A	VFMDAWLKTKRVNT<br>PQNDVNNAPAPITD<br>NMDIDTTPKQEDTKI<br>KSPSKTKSPSKPPPPD<br>LQNCFVLLSPCPTFPP<br>TQNTPPPHPNIPNG<br>NTLQDKPILVDPSVN<br>QSHAPNPAVDAAAT<br>DQPAKPSPKKRKLSP<br>EEKQAREDAKKAKEE<br>ERKLQAKQREDAKNK<br>KEEEKRLLALAREEEK<br>KKRDEEKRVKDQAKE<br>EARLKKEAEKKARDD<br>AKLQKDLEKKAKEEA<br>RLQKLQEKQKQEEEE<br>KRKKERQQAVLANFI<br>KVAPVEAPRKPQKVD<br>PDLLIQPFVCGSEFVF<br>PPSYLRTRQPSPGFD<br>DAIAHKDGQITIEKLQ<br>VEFREHMHGKRNRK<br>RDKKRRAEKLQGLPP<br>ALSNMKLLRFHDNVR<br>PAYYGTFSKRSAVLTG<br>RRPLRTDDAVFDYEV<br>DSDAEWEDEGEGEEL<br>SESEGEKDKEEEESEE
NP_015343.1	ScCac1	S. cerevisiae	CAF1A	KKGILSFFQNTTTVKS<br>NKFLTKEKDVITLDDP<br>KEDVSGPMIETVKQE<br>TMKSINKECADEMKT<br>TPKKANAEDKLLCYK<br>NSPIQSTKYDRNTNK<br>QVPNGNIIAIETKSRS<br>SSPCSKRELSSSKKEEA<br>KREKELKKQQRAEEK<br>HRKELLRQEEKKKKEL<br>KVEEERQRRAELKKQ<br>KEEEKRRKEEARLEAK<br>RRKEEERLKKEEEIRLK<br>EEAKERAQSRIGNFFK<br>KLSDSNTPVVEKSDYE<br>KFFLPFYAKDGVRVS<br>NKWKLTKVELEGSKR<br>KIDDELLNSKDKTSSD<br>DLLNWLQSRRLPRGH<br>KIKRKAVDVLQQMPL<br>KEKTDDELQSLLAQV<br>PHKYIKFYENVRPPFI<br>GTYSMDFTLPPNDPF<br>STKGTGFNYDYDSDV<br>EWVNEEEEGEVDNLE<br>SGEEEEEEEDDEDVPS<br>EGEFDGFLDSEENSDL<br>DGLPCAKRKFVGPLIP
XP_001021065.2	TtCAF1A	T. thermophi	CAF1A	NSRQMNIECQGDSP<br>AQTLNQSQNNVQQS<br>AVEVNSQNVQENIE<br>QAKVLNNQQNGQS<br>NISEQYWQSKKNGFI<br>QELNKLLIQGIDLSEQI<br>MFAMPQEILSQKIQD<br>YFSAINDQSSTEQIAD<br>LQRKLIKESCLWIVEG<br>KNCNDFDLIKLCTVLQ<br>EYTKQCLAQISDISQK<br>DFDFYRDIIGEWAEK<br>KNYGLKLTKDIQTLLL<br>KQQNEGNLSDQQVS<br>ALKIGLHEIWEVKEIQ<br>CLSNNTKIEELDYIRKK<br>RRQHQALLRGLEKLIK<br>AIEKGKGEDEAFSAY<br>QKTVMNPPKMKVQ<br>TKINGKSNSQKQNQ<br>VDKSIEDNLKKMKIDS<br>GKVTPIDVGEMQNE<br>TNQQENSQLIQQEAK<br>PKKKTAKKSNKKAAE<br>GSDQQIEQNEDTQK<br>QSICESSVNNNSITQE<br>QSKQISSKKNQNESK<br>KSGNQQQQQSASNT

NP_001082096.1	Xl-p150	X. laevis	CAF1A	STKSNTKKMVQARLP<br>FKRLNPVPKDEGCLEE<br>KKVRITKNVSPQKML<br>HSLNSSMEDMENDC<br>DMETETVPIPKAVNG<br>KGPLDNYIRKAPKVS<br>HAPSITTIDLTEESNISI<br>SNDCPLNGESETHLA<br>NGTLALEESTPNLPLS<br>AKEECTVSLENKTVEN<br>THFSELKSDQLHQAA<br>ATSTSASNFSPERVVK<br>EDCNSSADDDSASVS<br>SSSSPVSLSSPDAQTG<br>SQFRNRSSPSTSTTPT<br>GKVTANKTSADKNKT<br>KDKDKQRQAEKEERE<br>RAKKEARSAKKKKRQ<br>GLLKNLQRKRGKTSES<br>SGKEYKKEKKEREDKE<br>KAEKMKLKEEKKREK<br>LEALEAKQEEKRKKDE<br>EKRQKEEEKRQKEEE<br>KRLKEEEKRVKAEKAE<br>ITRFFQKPKTPQAPKT<br>FSRSCGKFAPFEIKKG<br>MALAPLCRIDFEPEAS<br>EELDRFLQEQNSKIYF
XP_008656983.1	ZmFAS1A-like	Z. mays	CAF1A	EAPDRNDAEPRRDM<br>GQSQMQVGGPVVL<br>NRSAELGSGDFMAID<br>DAPVQAPSGQPAAA<br>KHQSPAALTDTIMEV<br>QKQLKRKRASNNPVI<br>AAADKDALAAGCRQ<br>ELEGLFQFYKEVSDRK<br>VQLDGGNLSVNGMV<br>GCLLEESSLGLTKLVD<br>GIYEKLKGLDGVSVAS<br>VRGSVLLVGQRMMY<br>GKSSLDADVLEDDTE<br>SALWCWETRDLKLM<br>PVKARSVLSTRRSVRK<br>KIHERITAIYSTLSVLE<br>NPGVYVQVNDLKKA<br>SLRLNKSLNLEGIRSM<br>VERVAQKNLERGVKV<br>AGSAIKELKQETEKND<br>QNLRRLEDTSVSELQ<br>NGNSPDNEKEVQKA<br>QKQVEKEIKRQEKEE<br>ARMKKLQKKQQEDV<br>LREQKRREKEEAEAK<br>KQQKKQEEEALKEQK<br>RREKEEAEMKKQHKK<br>QQEEAEKEQKRLEKE
Q9SXY1	AtFAS2	A thaliana	CAF1B	PVLTVDFHPISGLLAT<br>AGADYDIKLWLINSG<br>QAEKKVPSVSYQSSLT<br>YHGCAVNTIRFSPSG<br>ELLASGADGGELFIW<br>KLHPSETNQSWKVH<br>KSLSFHRKDVLDLQW<br>SPDDAYLISGSVDNSC<br>IIWDVNKGSVHQILD<br>AHCHYVQGVAWDPL<br>AKYVASLSSDRTCRIY<br>ANKPQTKSKGVEKM<br>NYVCQHVIMKADQQ<br>RGDETKTIKTHLFHDE<br>TLPSFFRRLSWSPDGS<br>FLLIPAGSFKVSPTSEA<br>VNATYVFSRKDLSRP<br>ALQLPGASKPVVVVR<br>FCPVAFKLRGSSSEEG<br>FFKLPYRLVFAIATLNS<br>VYIYDTECVAPIAVLA<br>GLHYAAITDITWSPN<br>ASYLALSSQDGYCTLV<br>EFEDKELGEAVSISVG<br>KKPVDGEEKKHDLEK<br>GDELMTETTPDESKK<br>QAELEQNEESKQPLP<br>SKITTDGKEKEHIMQ

XP_020531827.1	AtrFAS2	A. trichopoda	CAF1B	QPVLTLDFHSLTNLLA<br>TGGADKDIKLWALV<br>QSEAQKKAPTACYQT<br>SLPPHHSTAVNVLRFS<br>PSGEQLASGADGGEL<br>AIWKMQSDDRHTW<br>KVLKTLSFHRKDVLDL<br>QWSTDGAFLLSGSVD<br>NSCIIWDVNKGAVH<br>QILDAHLHYVQGVA<br>WDPSGQYLASLSSDR<br>TCRIYSRKPQKNKAK<br>GQENLNYVCQHVVS<br>KGEFQNLGDTKLSSA<br>RHHLFHDETLPSFFRR<br>LAWSPDGSFLLAPAG<br>IDKLSSASPTCNTAYV<br>FSRNDLARPALQLPG<br>ASKPIVAVRFCPISFTL<br>RGSNSAGFFKLPYRIIF<br>AVATLNSLYIYDTESV<br>APVAILAGLHYAAITD<br>IAWSSDAKYLAVSSQ<br>DGYCTILEFENDELGS<br>PLSLTAATEAKIVAKT<br>SEESPVPKTEHMQVD<br>ENRVTIAVEEKARIQI<br>SKVEDNEPKEKNVEIK
NP_490902.2	CeChaf-2	C. elegans	CAF1B	GLLSVDLHHETRNGK<br>YRLATASVQKEVRVW<br>EFEFEMGLDPKTQEN<br>KPQLTVGFLANLVFH<br>NHAINQVKFSPSKEH<br>ELLASGDCEGRITIWK<br>LSDQPVPPPQDEMP<br>SNKENWIRYKVLNH<br>NSDVNALCWDPSGT<br>QLASVSNDHTLAVHD<br>ALTGKRLFVASNFRSP<br>NGVCWDPSGKYIAT<br>MSPDRKMDLIDAVR<br>GARLKHFSSALLPSM<br>TIPSANGDLHLETKIH<br>KLFHDDQLFSFQRAL<br>GFSPNGEFIAAPCAH<br>LELGSSDLYGTYFFRR<br>EDLGVKEAPYTFYPAP<br>RPTFLVKFSPVTFSLLP<br>STKENHLGLPYRLLWI<br>ALNKDAIYFYDSQHSY<br>PVAVVDNIHLNALTD<br>ASFSHDGRVLVVSSLE<br>GFCSFVKINLTQWGE<br>VMTEIVPVCGSPNLIE<br>EKKQKKRKSTVAVVIE<br>KEKEKEEDAAKSPMR
XP_646299.1	DdCAF1B	D. discoideum	CAF1B	PIYSADFDPTSNKFCT<br>TGFDNEIKIWGYTKN<br>KDGHLSVEFLSSLAKH<br>VKPVNIARFSPGGNL<br>LASGSDDGSIVIWRL<br>NQNAIPPSDSSMKEI<br>WSIVTVLRVTTDVYD<br>LSWSCDGQYLSTVST<br>DNSVSIWSPLSKTHH<br>QLITEHSHYVQGVSW<br>DPLNDFMITQSSDGT<br>CRIYRNEKKKKKIITQP<br>SSCPSSSTPVSEKDKD<br>SEDTDDNDNSNSND<br>SNNNNTNSNSTGNN<br>VVNSATIEDVNTNSR<br>KKLNLIISNVLSRRSYN<br>NTSIPNEDIAEIEELQ<br>QQQQQLDEAKIKEES<br>LVSHRMFYDERASTF<br>FRRPSWSPDGSIFITP<br>TGKFRDSPTSKYQSTS<br>YIFSRHIRDRPLIHLPS<br>NNPTVVVKFNPIIFKL<br>RPDTNNNNNNNNN<br>DDDSNNNDDNNNN<br>NNNNNNINNNNNT<br>NNDNDNDNDTDSDS

NP_610589.2	Dm-p105	D. melanogas	CAF1B	VLSVDIQQNGLGLRS<br>PTICRLASGGSDAHVL<br>IWYVNRSDDAEGVD<br>VELAADLSRHQRAVN<br>AVRWSPNGELLASG<br>DDESVVFIWKQKAD<br>HEVVNIVDADGCSEQ<br>DKEVWLTLKVLRGHR<br>EDIYDLSWAPNSQFL<br>VSGSVDNTAMLWDV<br>HSGKSLAILDDHKGY<br>VQGVAWDPCNQYIA<br>TMSTDRQMRIFDAN<br>TKRVLHRVSKCVLPV<br>KEDHEMHGKSMRLY<br>QDGTLQTFFRRLCFT<br>PDGKLLLTPSGITDYD<br>GVVKPINTSYGFSRH<br>DLSKPAFVLPFPKEYA<br>VAVRCSPVLYRLRPY<br>NAEKNPPIISLPYRMI<br>YAVATKNAVFFYDTQ<br>QPVPFAIVSNIHYSRL<br>TDLAWSSDGTVLIVSS<br>TDGYCSLITFEPTELG<br>DCYEDMETVLSVVLK<br>SSENATVLKKKRQKLR<br>KVSLDEPRKPLQEKSK
NP_001315058.1	DrChaf1B	D. rerio	CAF1B	PVYSLDFQQSGDGKT<br>QRLATAGVDTTVRM<br>WRVDKGPDGKAVVE<br>FLSNLARHTKAVNVV<br>RFSPTAEVLASGGDD<br>AAILLWKLNDNKEPE<br>QTPTFQEEEDAQLNK<br>ESWSVVKTLRGHIED<br>VYDISWTSDGNFMA<br>SGSVDNTAIMWDVN<br>KGQKMCIFNDHKSY<br>VQGVAWDPLGQYIS<br>TLSCDRVMRVYSAH<br>NRKKAYSISKMTSSAT<br>ADGEVKNYRMFHDD<br>SMRSFFRRLTFTPDGS<br>FLLAPAGCVEAGENV<br>TNTTYVFSRKSFKRPI<br>AHLPCPSKATLAVRC<br>SPVYFELRTKRAEDDS<br>LKPVANTFNLPYRLVF<br>AVASEDSIFFYDTQQT<br>LPFGYVSNIHYHTLSD<br>LSWSRDGSFLAVSST<br>DGYCSFISFDEGELGT<br>PLKEKPPLEMVTPSST<br>NEKKGKRALANGRTA<br>SPVPRQANTPAAQD
NP_005432.1	Hs-p60	H. sapiens	CAF1B	VYSLDFQHGTAGRIH<br>RLASAGVDTNVRIWK<br>VEKGPDGKAIVEFLSN<br>LARHTKAVNVVRFSP<br>TGEILASGGDDAVILL<br>WKVNDNKEPEQIAF<br>QDEDEAQLNKENWT<br>VVKTLRGHLEDVYDIC<br>WATDGNLMASASV<br>DNTAIIWDVSKGQKI<br>SIFNEHKSYVQGVTW<br>DPLGQYVATLSCDRV<br>LRVYSIQKKRVAFNVS<br>KMLSGIGAEGEARSY<br>RMFHDDSMKSFFRR<br>LSFTPDGSLLLTPAGC<br>VESGENVMNTTYVFS<br>RKNLKRPIAHLPCPGK<br>ATLAVRCCPVYFELRP<br>VVETGVELMSLPYRL<br>VFAVASEDSVLLYDT<br>QQSFPFGYVSNIHYH<br>TLSDISWSSDGAFLAI<br>SSTDGYCSFVTFEKDE<br>LGIPLKEKPVLNMRTP<br>DTAKKTKSQTHRGSS<br>PGPRPVEGTPASRTQ<br>DPSSPGTTPPQARQA

NP_082359.1	Mm-p60	M. musculus	CAF1B	VYSLDFQHGATWKIH<br>RLASAGVDTAVRIWK<br>LERGPDGKAIVEFLSN<br>LARHTKAVNVVRFSP<br>TGEILASGGDDAVILL<br>WKMNDSKEPEQIAF<br>QDEEEAQLNKENWT<br>VVKTLRGHLEDVYDIC<br>WATDGNLMTSASVD<br>NTVIIWDVSKGQKISI<br>FNEHKSYVQGVTWD<br>PLGQYIATLSCDRVLR<br>IYNTQKKRVAFNISK<br>MLSGQGPEGEARSFR<br>MFHDDSMKSFFRRLS<br>FTPDGSLLLTPAGCM<br>ESGENVTNTTYVFSR<br>KHLKRPIAHLPCPGK<br>ATLAVRCCPVYFELRP<br>VAETEKASEEPSPELV<br>NLPYRMVFAVASEDS<br>VLLYDTQQSFPFGYVS<br>NIHYHTLSDISWSSD<br>GAFLAISSTDGYCTFV<br>TFEKGELGIPLKEKPVL<br>SIRTPDTAKKAKNQT<br>HQGSSPGSRSVEGTP<br>SNRTQDPSSPCTTPS
XP_024390997.1	PpaFAS2-like	P. patens	CAF1B	PVLSLDFHPHSGLLAT<br>AGADHDIKIWSVHQ<br>KESGSPTVEFEADLCY<br>HSKAVNVLRFSPSGQ<br>LLASGGDGGEILIWKP<br>IERADKKPSWKVLKTL<br>QLHVRDVLDLAWSP<br>DSLSLMSGSVDNQC<br>MIWDVVSGKVLQVL<br>NDHQHFVQGVAWD<br>PAGEFLTSISSDRTCRI<br>YSRQSVPKSKKRKLAV<br>VQNMFTCKQVLAKS<br>DALPSKTNITIDADGR<br>VAKASQLFHDETLPSF<br>FRRLAWSPDSSFLVV<br>PSGLHRIAHEAPSCN<br>VSFIFSRSDLSRPCIHL<br>PAPTKPVVAVRFCPVI<br>FSLRENETEKGSSSGF<br>ALPYRLVFAVATLDSL<br>YVYDTQRSEPIVIFAGI<br>HYAAITDIAWSADAQ<br>YVAVSSQDGYCSLLIF<br>HGNELGTKLSSGDVP<br>ANIAKFLPENLLGKPQ<br>ESLPNSTELSQVSEPV<br>GLMNEEQSSPIVICPR
-	PpCAF1B	P. polycephal	CAF1B	PVFSVDFHSSGRLASS<br>GADNDIKIWKIGKRA<br>DGSVSLDFLSSLTRHT<br>KAVNVVRFSPDGNFL<br>ATGSDDGYIYIWKLN<br>TSVPVLPDSIDKEVW<br>SVAYTLRGKAQDVYD<br>LAWSPDSSQIIVGSTD<br>NSASVWSVKGAQCK<br>HHFEHHNHYVQGVA<br>WDPLGIYLLTFSSDRT<br>LRAYTRKLPKNKKSHK<br>ALPPPTPSSSASASTS<br>SSSSDPLLPVPDPLSS<br>PSSPVPAPAQNPPAA<br>TPSKKRVKEKWIVAQ<br>LVNKREFPGTQGLLE<br>KEKEKEKEESGKGKEK<br>EKEKDCLQHKLFLDES<br>VNTFFRRLTWSPEGS<br>LLFLPTGIHKASPASP<br>PTNVCYAFSRNNLNR<br>PLFYIPSGKPVIAVRCS<br>PILYKLWNGKHPAQT<br>KPLSTSPTPSTPSSAPS<br>PTSSSTSPTPASSTPVS<br>TDAPSAPTSTPPATIP<br>DTPMSDSPTNTAPTP

NP_013605.1	ScCac2	S. cerevisiae	CAF1B	PVYSLTFQKNSANDK<br>LFTAGGDNKVRIWKL<br>NRDENGQNGGVRKI<br>ESLDFLGSLTHHEQAI<br>NVIRFNSKGDVLASA<br>GDDGQVLLWKQEDP<br>NTQQESVVRPFGMD<br>AETSEADENKEKWVV<br>WKRLRGGSGATAAA<br>EIYDLAWSPDNRNIV<br>VACMDNSIRLFDVGA<br>GMLVCGQSDHGHYV<br>QGVAWDPLNQFILS<br>QSADRSLHVYGVILSS<br>AGVVTGLKLRSKIAKA<br>ELPCPGDVLRTNYLFH<br>NETLPSFFRRCSISPC<br>GGLVVIPSGVYKVAG<br>DEVANCVYVYTRSGIL<br>NSAGGVKNRPAIRIP<br>SLKKPALMAAFSPVF<br>YETCQKSVLKLPYKLV<br>FAIATTNEVLVYDTDV<br>LEPLCVVGNIHYSPIT<br>DLAWSEDGSTLLISST<br>DGFCSYVSIDTETQFG<br>SRIEPPAMHAEPLDT<br>DESAVAAKNQREAG
XP_001020614.1	TtFAS2	T. thermophi	CAF1B	QRIVTLDVHPFINLLV<br>TGGSDEEVYEGEDLEF<br>EEEIGYIKLWQINENY<br>TPGQGGDAKPVTFIR<br>ALKKHSNPVNCVRFS<br>PNGQYLASASDDHKI<br>VIWHVDKGFQNISTN<br>QKIFNLVPKNILEGHN<br>REVCDLRWFNDSTHL<br>ISGGMDYRAYIWNV<br>KEGVIKQTIVGAHKSY<br>VQGVAVDPKMKFCL<br>TLGNDRTVKVWRKLK<br>SNNKKKNIFEYIPSNT<br>MKRLPFADVNIEEEM<br>LSDDEDKSSITGNQM<br>NGANGAVNQAMTY<br>GMFLSERQLNTFVKR<br>PDWSPDGSFFLLPAA<br>IYQEKRDSKIEMCVYL<br>YRRNVLNKPSLIINTN<br>NKPAICTRFCQKLFKK<br>KEENQFSMVDIPYVII<br>FAISTIDNVMIYSTASL<br>SPLAVVGNIHFALIND<br>LTFFSNQSLIICSSDG<br>MCSFVFFEENDLGKP<br>LNSEEIQDEEIRNIVN
NP_001090195.1	Xl-p60	X. laevis	CAF1B	VYSLDFQHGNDKFNR<br>LASAGVDTAVRLWKI<br>ERSPEGKAVVEFLASL<br>ARHTKAVNVVRFSPN<br>GEILASGGDDAAILL<br>WKLNETKECEPAPFQ<br>EQDEFELNKENWTVF<br>KTLRGHLEDVYDICW<br>TQDSNFMVSASVDN<br>TAITWDVTKGQKLSIF<br>NEHKSYVQGVTWDP<br>LGQYIATLSCDRIMRV<br>YKTETKRVAYNVSKM<br>TSSPGPDGEAKSFRM<br>YHDDSMKSFFRRLTF<br>TPDGSLLLTPAGCVEA<br>GETVINTTYVFGRKNL<br>RRPIAHLPCPTKATLA<br>VRCCPVYFELRPTMK<br>DDSGESRPQGLITQP<br>YRMVFAVASEDAVLF<br>YDTQQLFPFGYVSNV<br>HYHTLSDISWSSDGN<br>FLAISSTDGYCSFVTFE<br>EGELGVPLKEKPVALI<br>PKTPATERKIKKAQSK<br>KVSSPGSRQELTPSNK<br>SMEHSAPSTPMNSR

NP_001132835.1	ZmFAS2	Z. mays	CAF1B	QPVLSLDFHPVSRRL<br>ATAGADHDVKIWEV<br>ASDGKLPTATFKTGLV<br>PNTTAHSSAVNVLRF<br>SPSGEYLASGADGGG<br>ITLWKLHPADDGEA<br>WKIHKTLLFHHKDVL<br>DLQWSHDSTFLVSAS<br>VDNTCIIWEASKGTV<br>QQKLEGHLHYVQGV<br>AWDPLGQYIASLSSD<br>RTCKIYAKKPQGKSK<br>NAERLNFVCQHTLVK<br>VEYQNHDESKPPTKS<br>HLFHDETLPSFFRRLA<br>WSPDGSFLVLPAGLS<br>KHSSEVINTAYVMSR<br>RDLSRPSIQLPGASKA<br>IVAVRFCPVLFKPRGS<br>NSDGFFKLPYRVVFA<br>VATLNSLYVYDTESVP<br>PILVHAGLHYAAITDI<br>AWSSDAKYLAVSSRD<br>GYCTIIEFENEELGQP<br>HILPGSKEVANGNLT<br>PETKKPVCADSMKVD<br>NSANKLKMEASPVA<br>VGVRAPLLPTENIKRT
NP_200631.1	AtMSI1	A thaliana	CAF1C	LINEEYKIWKKNTPFL<br>YDLVITHALEWPSLTV<br>EWLPDREEPSGKDYS<br>VQKMILGTHTSESEP<br>NYLMLAQVQLPLDDT<br>ESEARQYDDDRSEFG<br>GFGCATGKVQIIQQI<br>NHDGEVNRARYMP<br>QNPFIIATKTVNAEVY<br>VFDYSKHPSKPPLDG<br>ACNPDLKLRGHSSEG<br>YGLSWSKFKQGHLLS<br>GSDDAQICLWDINAT<br>PKNKSLDAQQIFKAH<br>EGVVEDVAWHLRHE<br>YLFGSVGDDQYLLIW<br>DLRSPSASKPVQSVV<br>AHSMEVNCLAFNPF<br>NEWVVATGSTDKTV<br>KLFDLRKLSTALHTFD<br>SHKEEVFQVGWNPK<br>NETILASCCLGRRLM<br>VWDLSRIDEEQTVED<br>AEDGPPELLFIHGGH<br>TSKISDFSWNPCEDW<br>VISSVAEDNILQIWQ<br>MAENIYHDEDDAPG<br>EEPSKAS
XP_006826731.1	AtrMSI4	A. trichopoda	CAF1C	RYTQWKSLVPVLYD<br>WLANHNLVWPSLSC<br>RWGPQLEQATYKNR<br>QRLYLSEQTDGSVPN<br>TLVIANCEVVKPRVA<br>AAEHISQFNEEARSPF<br>VKKFKTIIHPGEVNRI<br>RELPQNSKIVATHTDS<br>PDVLIWDVEAQPNR<br>HAILGASDSRPDLILT<br>GHKDNAEFALAMCP<br>TDPYVLSGGKDKSVV<br>LWSIEDHISAAAAEA<br>PSTKSPGVPSSSGSKS<br>NTKTVGPSDKILDSPA<br>IGPRGIYKGHSDTVED<br>VQFCPSSAQEFCSVG<br>DDSCLILWDARIGVD<br>PAIKVEKAHNADLHC<br>VDWNPHDENLILTGS<br>ADNSVRMFDRRNLT<br>SNGIGSPVYKFEGHK<br>AAVLCVQWSPDKAS<br>VFGSSAEDGYLNVW<br>DHEKVSKKKENVGNK<br>MPNSPAGLFFQHAG<br>HRDKVVDFHWNVSD<br>PWTIVSVSDDGESTG

NP_492552.1	CeLIN-53	C. elegans	CAF1C	MATLEDGTSEDRVA<br>NDEYKIWKKNTPFLY<br>DLVMTHALEWPSLSV<br>QWLPDVAKDNSDHT<br>IHRLILGTHTSDEQNH<br>LLISKICMPTDDAQFD<br>ASRYDTERSEYGGFG<br>AVNGKVEPDIRINHE<br>GEVNRARYMPQKSN<br>IIATKSPHADVYIFDYL<br>KHSAVPRDNTFNPLI<br>RLKGHTKEGYGLSW<br>NPNKEGLILSASDDQ<br>TVCHWDINANQNVA<br>GELQAKDVFKGHESV<br>VEDVAWHVLHDGVF<br>GSVGDDKKLLIWDVR<br>TSTPGHCIDAHSAEV<br>NCLAFNPYSEFILATG<br>SADKTVALWDLRNLR<br>MKLHSFESHRDEIFQ<br>VQWSPHNETILASSG<br>TDKRLHVWDLSKIGE<br>DQSAEDAEDGPPELL<br>FIHGGHTAKISDFSW<br>NPNEPWVVCSVSED<br>NILQVWQMADNIYN<br>EVDEETPADVVERQQ
XP_640105.1	DdCAF1C	D. discoideur	CAF1C	EEYKIWKRHTPFLYD<br>MVITHALEWPSLTVA<br>WLPVKTSQPNKPYSI<br>EKVILGTHTSDEEQNY<br>LMVAKVHLPVDEASI<br>ESLKYDDTKGEVGGI<br>GNVSEKIEIIQKINHE<br>GEVNRARVMPQNHS<br>IIATKTVSSEVYIFDTT<br>KHPLEPTPDGKCSPN<br>LKLTGHKKEGYGISW<br>NPRKEGHLLSCSDDQ<br>SICMWDISAASKSDS<br>TLDALNIYNGHTSIVE<br>DVAWHYIHDTFFGSV<br>GDDKKLMIWDTRTG<br>TKPIHVVEAHNSEVN<br>CLSFNPFCEFLVATGS<br>TDKTVALWDMRNLG<br>NRLHSLISHTDEVFQV<br>QFSPHNETVLASCGS<br>DRRVNVWDLSRIGEE<br>QNNEDAADGPPELLF<br>IHGGHTSKISDFSWN<br>PNDPWSIASVAEDNI<br>LQIWQMAENIYNDR<br>EDDLENSKVTNAQIG<br>E
NP_524354.1	Dm-p55	D. melanogas	CAF1C	AVEERVINEEYKIWKK<br>NTPFLYDLVMTHALE<br>WPSLTAQWLPDVTK<br>QDGKDYSVHRLILGT<br>HTSDEQNHLLIASVQ<br>LPSEDAQFDGSHYDN<br>EKGEFGGFGSVCGKI<br>EIEIKINHEGEVNRAR<br>YMPQNACVIATKTPS<br>SDVLVFDYTKHPSKPE<br>PSGECQPDLRLRGHQ<br>KEGYGLSWNPNLNG<br>YLLSASDDHTICLWDI<br>NATPKEHRVIDAKNIF<br>TGHTAVVEDVAWHL<br>LHESLFGSVADDQKL<br>MIWDTRNNNTSKPS<br>HTVDAHTAEVNCLSF<br>NPYSEFILATGSADKT<br>VALWDLRNLKLKLHS<br>FESHKDEIFQVQWSP<br>HNETILASSGTDRRLH<br>VWDLSKIGEEQSTED<br>AEDGPPELLFIHGGH<br>TAKISDFSWNPNEP<br>WIICSVSEDNIMQV<br>WQMAENVYNDEEP<br>EIPASELETNTA

NP_997760.1	DrRBBP4	D. rerio	CAF1C	RVINEEYKIWKKNTPF<br>LYDLVMTHALEWPSL<br>TAQWLPDVTRPEGK<br>DFSVHRLVLGTHTSD<br>EQNHLVIASVQLPND<br>DAQFDASHYDSEKGE<br>FGGFGSVSGKIEIEIKI<br>NHEGEVNRARYMPQ<br>NPCIIATKTPTSDVLV<br>FDYTKHPSKPDPSGE<br>CTPDLRLRGHQKEGY<br>GLSWNPNLRSCLLSA<br>SDDHTICLWDISTVPK<br>EGKIVDAKTIFTGHTA<br>VVEDVSWHLLHESLF<br>GSVADDQKLMIWDT<br>RSNNTSKPSHAVDAH<br>TAEVNCLSFNPYSEFIL<br>ATGSADKTVALWDL<br>RNLKLKLHSFESHKDE<br>IFQVQWSPHNETILA<br>SSGTDRRLNVWDLSK<br>IGEEQSPEDAEDGPP<br>ELLFIHGGHTAKISDF<br>SWNPNEPWVICSVS<br>EDNIMQVWQMAEN<br>IYNDEDPEGAADTEV<br>QG
NP_001128727.1	Hs-p48	H. sapiens	CAF1C	RVINEEYKIWKKNTPF<br>LYDLVMTHALEWPSL<br>TAQWLPDVTRPEGK<br>DFSIHRLVLGTHTSDE<br>QNHLVIASVQLPNDD<br>AQFDASHYDSEKGEF<br>GGFGSVSGKIEIEIKIN<br>HEGEVNRARYMPQN<br>PCIIATKTPSSDVLVFD<br>YTKHPSKPDPSGECN<br>PDLRLRGHQKEGYGL<br>SWNPNLSGHLLSASD<br>DHTICLWDISAVPKE<br>GKVVDAKTIFTGHTA<br>VVEDVSWHLLHESLF<br>GSVADDQKLMIWDT<br>RSNNTSKPSHSVDAH<br>TAEVNCLSFNPYSEFIL<br>ATGSADKTVALWDL<br>RNLKLKLHSFESHKDE<br>IFQVQWSPHNETILA<br>SSGTDRRLNVWDLSK<br>IGEEQSPEDAEDGPP<br>ELLFIHGGHTAKISDF<br>SWNPNEPWVICSVS<br>EDNIMQVWQMAEN<br>IYNDEDPEGSVDPEG<br>QGS
NP_033056.2	Mm-p48	M. musculus	CAF1C	RVINEEYKIWKKNTPF<br>LYDLVMTHALEWPSL<br>TAQWLPDVTRPEGK<br>DFSIHRLVLGTHTSDE<br>QNHLVIASVQLPNDD<br>AQFDASHYDSEKGEF<br>GGFGSVSGKIEIEIKIN<br>HEGEVNRARYMPQN<br>PCIIATKTPSSDVLVFD<br>YTKHPSKPDPSGECN<br>PDLRLRGHQKEGYGL<br>SWNPNLSGHLLSASD<br>DHTICLWDISAVPKE<br>GKVVDAKTIFTGHTA<br>VVEDVSWHLLHESLF<br>GSVADDQKLMIWDT<br>RSNNTSKPSHSVDAH<br>TAEVNCLSFNPYSEFIL<br>ATGSADKTVALWDL<br>RNLKLKLHSFESHKDE<br>IFQVQWSPHNETILA<br>SSGTDRRLNVWDLSK<br>IGEEQSPEDAEDGPP<br>ELLFIHGGHTAKISDF<br>SWNPNEPWVICSVS<br>EDNIMQVWQMAEN<br>IYNDEDPEGSVDPEG<br>QGS

XP_001776478.1	Ppa-p48	P. patens	CAF1C	LVPALYDWLTNHHL<br>MWPSLSCRWGPQLE<br>AGSSKTRQLLFYSERT<br>DGECPNTIVVAQCDI<br>MKPRTAAAEQISQAF<br>IPPCCMSQRPEFKEG<br>GKSPHLKKLKTIIHPG<br>EVNRIREIPQNSNILA<br>THTDSPKVLIWNTKT<br>QPNRATTSAASESKP<br>DLVLIGHTDNAEFAL<br>NVSRTAPYVISGGKTP<br>KSITPTAAGSKQSGTA<br>GGAADTTNVYTRGIF<br>KGHTDTVEDVQFRPS<br>SMNEFCSVGDDSCLL<br>LWDARTGYQPISKVV<br>KAHNADLHCVDWN<br>AHDENLILTGSADNS<br>VRLFDHRKILARGQAI<br>PVEQFEGHSAAVLCV<br>QWCPDRASVFGSCA<br>EDGLLNVWDYEKVG<br>KALDTTNLKQPVKVP<br>PGLFFQHTGHRDKVV<br>DFHWDSRDPWTIVS<br>VSEDANTPGGGGTL<br>QIWRMIDFIYRPEDE
-	PpCAF1C	P. polycephal	CAF1C	NEEYKIWKKNAPFLY<br>DLVMTHALEWPSLTV<br>HWLPDKKIQSNKEFS<br>VQRLVLGTHTSDAEQ<br>NYLMIAKVHLPLDDA<br>NIDPRKYDDQKGEVG<br>GFGAVSEKIEIEQRIN<br>HSGEVNRARAMPQ<br>NPTIIATKTVTSDVFLF<br>DYTRHPSKPSADGKC<br>NPNAILKGHTKEGYGI<br>DWNPSREGHLLSASD<br>DKTICLWDIHDAKAN<br>TTIEAKQIFRGHKEVV<br>EDVAWHCSHADYFA<br>SAGDDKKLMIWDVR<br>TNADPLQVEAHAGE<br>VNCVAFNPFCEFLLA<br>TGSADKTVALWDMR<br>NLDAKLHSFVSHTDE<br>VFQVQWSPHHETVL<br>ASCGSDRRVNVWDL<br>SRIGDEQTAEDAEDG<br>PPELLFIHGGHTSKIS<br>DFSWNYNEPWVLAS<br>VAEDNIIQVWQMAE<br>NIYNDDDTDEAQPSN<br>EGDLE
NP_009754.1	ScCac3	S. cerevisiae	CAF1C	MNQCAKDITHEASSI<br>PIDLQERYSHWKKNT<br>KLLYDYLNTNSTKWP<br>SLTCQFFPDLDTTSDE<br>HRILLSSFTSSQKPEDE<br>TIYISKISTLGHIKWSSL<br>NNFDMDEMEFKPEN<br>STRFPSKHLVNDISIFF<br>PNGECNRARYLPQN<br>PDIIAGASSDGAIYIFD<br>RTKHGSTRIRQSKISH<br>PFETKLFGSHGVIQDV<br>EAMDTSSADINEATS<br>LAWNLQQEALLLSSH<br>SNGQVQVWDIKQYS<br>HENPIIDLPLVSINSD<br>GTAVNDVTWMPTH<br>DSLFAACTEGNAVSLL<br>DLRTKKEKLQSNREK<br>HDGGVNSCRFNYKN<br>SLILASADSNGRLNL<br>WDIRNMNKSPIATM<br>EHGTSVSTLEWSPNF<br>DTVLATAGQEDGLVK<br>LWDTSCEETIFTHGG<br>HMLGVNDISWDAH<br>DPWLMCSVANDNS<br>VHIWKPAGNLVGHS

XP_001026968.2	TtCAF1C	T. thermophi	CAF1C	ENQAINDEYKIWKKN<br>APFLYDIAITHELEWP<br>SLSVQWLPTKDIPQE<br>SDYAIHKLILGTHTSG<br>QDKDYLLIAKVRLPLE<br>ETATDISEYQNQAKE<br>VGQTGLSAGENRIEIE<br>TKILHDGEINRARYM<br>PQKYNVIATKVTNGEI<br>HVFDYTQHPTTPQN<br>DQVRPQLRLVGHSAE<br>GYGISWNPKKQGYIV<br>SGGYDKKICIWNVEA<br>ASQLNSSISPLHDIEF<br>HKSCVEDVAWHQIN<br>PDIFGSVSDDRTVAI<br>WDMRQKSNAGLINP<br>THCTQAHTGDIYCLD<br>FNPFNEYLFITGSEDK<br>NIGFWDMRNTSKRL<br>HTFVGHTDQVLRCE<br>WSPFNVGVFSSCSAD<br>RRVIVWDISKCGQE<br>MKNEDLVDGPPELLF<br>MHGGHRAKVNDIS<br>WNQKENLILASVEEN<br>NILQVWQMARNIYD<br>DTDDEVMKD
NP_001083811.1	Xl-p48	X. laevis	CAF1C	RVINEEYKIWKKNTPF<br>LYDLVMTHALEWPSL<br>TAQWLSDVTRPDGK<br>DFSIHRLVLGTHTSDE<br>QNHLVIASVQLPNDD<br>AQFDASHYDSEKGEF<br>GGFGSVSGKIEIEIKIT<br>HDGEVNRARYMPQ<br>NPCIIATKTPTSDVLV<br>FDYTKHPSKPDPSGE<br>CNPNLRLRGHQKEGY<br>GLSWNPNLSGNLLSA<br>SDDHTICLWDISAVP<br>KEGKVVDAKTIFTGH<br>TAVVEDVSWHLLHES<br>LFGSVADDQKLMIW<br>DTRSNNTSKPSHSVD<br>AHTAEVNCLSFNPYS<br>EFILATGSADKTVAL<br>WDLRNLKLKLHSFES<br>HKDEIFQVQWSPHN<br>ETILASSGTDRRLNV<br>WDLSKIGEEQSPEDA<br>EDGPPELLFIHGGHT<br>AKISDFSWNPNEPW<br>VICSVSEDNIMQVW<br>QMAENIYNDEDTEG<br>GVDPEGQGS
NP_001105556.1	ZmMSI1	Z. mays	CAF1C	RAEVEERLINEEYKIW<br>KKNTPFLYDLVITHAL<br>EWPSLTVQWLPDRT<br>EPPGKDHSVQKMILG<br>THTSDNEPNYLMLA<br>QVQLPLDDAEADAR<br>HYDDDHADIGGFGA<br>ASGKVQIVQQINHD<br>GEVNRARYMPQNSFI<br>IATKTVSAEVYVFDYS<br>KHPSKPPLDGACNPD<br>LRLKGHNSEGYGLSW<br>SIFKEGHLLSGSDDAQ<br>ICLWDIKANSRNKSL<br>DALQIFKHHDGVVED<br>VAWHLRHEYLFGSV<br>GDDYHLLIWDLRSPA<br>PTKPVQSVVAHQGE<br>VNCLAFNPFNEWVV<br>ATGSTDKTVKLFDLRK<br>IDTSLHTFDCHKEEVF<br>QVGWSPKNETVLAS<br>CCLGRRLMVWDLSRI<br>DQEQTPEDAEDGPPE<br>LMFIHGGHTSKISDFS<br>WNPCEDWVVASVA<br>EDNILQIWQMAENIY<br>HDEDDLPISDEPAKTS

NP_001319681.1	AtHIRA	A thaliana	HIRA	QIFSIDVQPNGERFAT<br>GGGDHKVRIWNMK<br>SVDKDLQNIDTKERLL<br>ATLRDHFGSVNCVR<br>WAKNSRYVASGSDD<br>QVIQIHERKPGSGTTE<br>FGSGEAPDVENWKA<br>VMTLRGHTADVVDL<br>NWSPDDSMLASGSL<br>DNTVHIWNMRTGM<br>CTTVLRGHLSLVKGVT<br>WDPIGSFIASQSDDK<br>TVIIWRTSDWGMAH<br>RTDGHWAKSLGSTFF<br>RRLGWSPCGHFLTTT<br>HGFQKPKHSAPVLER<br>GEWSVAYDFLGHSA<br>PIIVVRFNHSMFKRIP<br>SSTHETKQVGWSNG<br>TSKSGEKDLQSYNVIA<br>MGSQDRTITVWTTG<br>SARPLFVAKHFFGQS<br>VVDLSWSPDGYSLFA<br>CSLDGTVAMIHFDPK<br>ELGVRLTDTELDELKK<br>SRYGDVRGRQANLV<br>ESPAQLLLETASTKQA<br>GSKRAASDVQQNQV
XP_006851504.1	AtrHIRA	A. trichopoda	HIRA	QIFSIDIQPGGLRFAT<br>AGGDHKVRIWNMKS<br>VGRETEYDQTNQTLL<br>ATLRDHFGSVNCVR<br>WAKHGRYVASGSDD<br>QVILVHERKPGSGTTE<br>FGSGEPPDVENWKV<br>VMTLRGHTADVVDL<br>NWSPDDLILASCSLD<br>NTVHIWNMSNGICT<br>AVLRGHSSLVKGIAW<br>DPIGSFIASQSDDKTV<br>IIWRTNDWSQEHRTE<br>GHWGKSVGSTFFRRL<br>GWSPCGHFITTTHGF<br>QKPRHSAPVLERGD<br>WSATFDFLGHNAPV<br>VAVKFNHSMFRKNL<br>ANGADLKEFQQVET<br>VGWSNGATKSVPRE<br>SMPYNVIAIGSQDCTI<br>TVWTTASPRPLFVAK<br>HFFTQSVVDLSWSPD<br>GYALFACSLDGTVAT<br>FYFEVKELGHRISDAE<br>LDELKRSRYGDVRGR<br>QANLAESPAQLLLEV<br>ASAKQLPTKRGLSNLS
NP_498101.2	CeHIRA	C. elegans	HIRA	DTGSILAIDCHPSGKK<br>FITCGQKARTSNGLV<br>VVWNAEPVLDKKKA<br>SNENVPKLLFQVESQ<br>SQSNSCRWSPDGKR<br>FAFGSDDSSVSVWEY<br>VGLINSMGSITGGAQ<br>NVERYKECCVLRGHS<br>MEVLTVEWSPNGKY<br>LASGSIDYRIIIYNARK<br>LPDRITVLNDIQLPVK<br>GLSWDPIGKYLASLE<br>GDKKLRFWATDSWQ<br>CVKSVTEPFESNIEET<br>MLTRLDWSPDGKYL<br>MTPAAVRSGKPLIKLI<br>QRQTWKSDQFLAGH<br>HKGTTCVRAMPRLIE<br>ANLKNGKRMQLTCA<br>AVGSRDKSISIWVFP<br>GTLKPLFVINNIFNHT<br>VMDFAWCGRNLLAC<br>SQDGTVKVIHLSESVI<br>GEMISNEAMSDLCY<br>QIYSIRPPRYELTDKEE<br>DESQDSFNLSDLSSSA<br>NNASFVTCPEDVLIKR<br>KKLVAAQQSSDIQLT

XP_643117.1	DdHIRA	D. discoideur	HIRA	SIYSIDIHPDGTRVAT<br>GGGDAKIKIWSMAPI<br>SLLEAEEDAGIPKLLCS<br>IENAHFHSVNSVKWS<br>KDGKYLASGSDDKLC<br>MIWGLSNNNSLLKN<br>TTENWVCVATLRGH<br>ASDISEVSWSPDNKYI<br>ATCSFDKSIIIWETNK<br>FQMVSKLEEHKGFVK<br>GLTWDPLGRYLASQS<br>EDKSLIIWRTSDWVV<br>ETIVTEPFKHSGNSFF<br>LRPSWTPDGQFIVAT<br>HGINNATHTGVLVSR<br>TDWDIGLDLVGHRK<br>AVVVSRCSSKIYKDFK<br>SRDQKFCLILLGGQDS<br>TLSLWSSSSPRSLMVT<br>RSLFDQCIQDISWCS<br>DGYSFVACSTDGTVG<br>YISLDPEEIGGSPIGPE<br>EKQIFFKNYYGDAVTI<br>DKEGNVIYGAAGGNL<br>SSAANSGSHAILPENP<br>DQLAMEESNANGVG<br>SGVSGGGVSGGGGS<br>GSGINNQNGGENHS
NP_572401.2	DmHIRA	D. melanogas	HIRA	QIFSVDIHKDCTKFAT<br>GGQGSDCGRVVIWN<br>LLPVLSDKAEFDADVP<br>KMLCQMDQHLACV<br>NCVRWSQNGQNLA<br>SGSDDKLIMIWRKSA<br>GSSGVFGTGGMQKN<br>HESWKCFYTLRGHD<br>GDVLDLAWSPNDVY<br>LASCSIDNTVIIWDAQ<br>AFPHSVATLKGHTGL<br>VKGVSWDPLGRFLAS<br>QSDDRSIKIWNTMN<br>WSLSHTITEPFEECGG<br>TTHILRLSWSPDGQYL<br>VSAHAMNGGGPTA<br>QIIEREGWKCDKDFV<br>GHRKAVTCVRFHNSI<br>LSRQENDGSPSKPLQ<br>YCCLAVGSRDRSLSV<br>WMTALQRPMVVIHE<br>LFNASILDLTWGPQE<br>CLLMACSVDGSIACL<br>KFTEEELGKAISEEEQ<br>NAIIRKMYGKNYVNG<br>LGKSAPVLEHPQRLLL<br>PQGDKPTKFPLSNNN<br>EANQRPISKQTETRTK
XP_696478.2	DrHIRA	D. rerio	HIRA	PIFSVDIHPDGTKFAT<br>GGQGEDSGKVVIWN<br>MAPVLREEDEKNENI<br>PKLLCQMDNHLACV<br>NCVRWSNNGLYLAS<br>GGDDKLVMVWKRA<br>AFIGPSTVFGSSSKLA<br>NVEQWRCVTILRNH<br>TGDVMDVAWSPHD<br>VWLASCSVDNTIVIW<br>NARKFPEIVMTLKGH<br>TGLVKGLTWDPVGKY<br>IASQADDHSLKVWRT<br>MDWQLETNITKPFSE<br>CGGTTHVLRLSWSPD<br>GQYLVSAHAMNNSG<br>PTAQIIERDGWKTN<br>MDFVGHRKAVTVVK<br>FNPKIFKKKQKNGST<br>PKPSCPYCCCAVGSK<br>DRSLSVWLTSLKRPLV<br>VIHDLFDKSIMDITWT<br>LNGLGLLVCSMDGTV<br>AFLDFSQDELGDPLN<br>EEEKNAIHQNIYGKSL<br>AITMESQLSTTIIENPE<br>MLKYQQERQGNQN<br>ANVAQGSGPENQTP

NP_003316.3	HsHIRA	H. sapiens	HIRA	PIFSVDIHPDGTKFAT<br>GGQGQDSGKVVIW<br>NMSPVLQEDDEKDE<br>NIPKMLCQMDNHLA<br>CVNCVRWSNSGMYL<br>ASGGDDKLIMVWKR<br>ATYIGPSTVFGSSGKL<br>ANVEQWRCVSILRN<br>HSGDVMDVAWSPH<br>DAWLASCSVDNTVVI<br>WNAVKFPEILATLRG<br>HSGLVKGLTWDPVG<br>KYIASQADDRSLKVW<br>RTLDWQLETSITKPFD<br>ECGGTTHVLRLSWSP<br>DGHYLVSAHAMNNS<br>GPTAQIIEREGWKTN<br>MDFVGHRKAVTVVK<br>FNPKIFKKKQKNGSS<br>AKPSCPYCCCAVGSK<br>DRSLSVWLTCLKRPLV<br>VIHELFDKSIMDISWT<br>LNGLGILVCSMDGSV<br>AFLDFSQDELGDPLSE<br>EEKSRIHQSTYGKSLAI<br>MTEAQLSTAVIENPE<br>MLKYQRRQQQQL<br>DQKSAATREMGSAT
NP_034565.2	MmHIRA	M. musculus	HIRA	PIFSVDIHPDGTKFAT<br>GGQGQDSGKVVIW<br>NMSPVLQEDDEKDE<br>NIPKMLCQMDNHLA<br>CVNCVRWSNSGMYL<br>ASGGDDKLIMVWKR<br>ATYIGPSTVFGSSGKL<br>ANVEQWRCVSILRSH<br>SGDVMDVAWSPHD<br>AWLASCSVDNTVVI<br>WNAVKFPEILATLRG<br>HSGLVKGLTWDPVG<br>KYIASQADDRSLKVW<br>RTLDWQLETSITKPFD<br>ECGGTTHVLRLSWSP<br>DGHYLVSAHAMNNS<br>GPTAQIIEREGWKTN<br>MDFVGHRKAVTVVK<br>FNPKIFKKKQKNGSST<br>KPSCPYCCCAVGSKD<br>RSLSVWLTCLKRPLVV<br>IHELFDKSIMDISWTL<br>NGLGILVCSMDGSVA<br>FLDFSQDELGDPLSEE<br>EKSRIHQSTYGKSLAI<br>MTEAQLSTAVIENPE<br>MLKYQRRQQQQL<br>DQKNATTRETSSASS
XP_001762381.1	PpaHIRA	P. patens	HIRA	QIFSVDTQPNGLRFA<br>TAGGDHKVRIWNM<br>KPLAEKETDSDTKLLA<br>TLRDHFGSVNCVRW<br>AKCGQKIASGSDDQV<br>VYIHEKRPGSGTTEFG<br>SGEPPDVENWKVLLT<br>LRGHSADVVDLGWS<br>PDDTQLASCSLDNSIR<br>IWQTSTGALLSVLTG<br>HQSLVKGLTWDPIGS<br>FLATQSDDKSVIIWRT<br>SNWSMVKKVEGPW<br>EKTVGSTFFRRLGWS<br>PCGHFIATTHGFQNP<br>SHTAPVLERGEWMA<br>SFDFVGHNAPVVAV<br>RFNHSMFRKVKTIAL<br>SGNGAILGAANGTSS<br>KTKEPPPYNVIAIGSQ<br>DCCISVWTTGSPRPV<br>FIGKHFFQQSVVDLS<br>WSPDGYTLFCCSLDG<br>SVASFQFERKELGEKI<br>SDAEMEEFKKSRYGD<br>LRVRQATVAESPAQL<br>MLEAAAAKQWGSG<br>NASNANTIDSTKAVS

-	PpHIRA	P. polycephal	HIRA	ACAKFEDQQNTSYNE<br>QMRILKPDWVTHTG<br>YPIFSVDIHPDGSRFA<br>TGGGDNKVIIWASAP<br>VQYEEQEANNSCPRK<br>LAELTYHMSPISCVR<br>WSHNGQFLASGSDD<br>ALVMLWKLSSGPASK<br>SFGSDELTQENWRCT<br>ATLRGHSKDITDVAW<br>SPDDSQLASCSVDNEI<br>LVWDTAKNSVVAKLS<br>GHTGFVKGVAWDP<br>MGRYIASQSEDKTMI<br>VWRCSDWKIETKIEE<br>PFVRSAATTFYRRPS<br>WSPDGQFISSAHAFS<br>GSNHVAMITARDTW<br>RSVCDFVGHKLPIVVT<br>RFNPVIFADTAGNNY<br>GVCLIGGQDNTISVW<br>TTISSRPLLALNSCFKQ<br>SVLDASWAKDGFSF<br>MCCSTDGTVVWVEF<br>AKGELGTALSPKEMD<br>KVLRSIYGDHILSRPKS<br>TVTVAEDPSQLYLPKT<br>TAPMKPVAAQPPPV
NP_009545.2	ScHIR1	S. cerevisiae	HIRA	SRKYEIYTVDVSHDGK<br>RLATGGLDGKIRIWSI<br>DSILRCMELESLTPEIP<br>LPQDLQMPLCSMSR<br>HTGSITCVKFSPDGKY<br>LASGSDDRILLIWALD<br>EEQSSQPAFGSEHER<br>EHWTVRKRLVAHDN<br>DIQDICWAPDSSILVT<br>VGLDRSVIVWNGSTF<br>EKLKRFDVHQSLVKG<br>VVFDPANKYFATTSD<br>DRTMKIFRYHKTGDIS<br>FTIEHIITEPFKESPLTT<br>YFRRPSWSPDGQHIA<br>VPNATNGPVSSVAIV<br>NRGTWDTNVSLIGH<br>DAPTEVARFNPRLFE<br>RNAGVKQKKDDDPE<br>NALVGQNDDKVHHF<br>DKNIDSVVATAGQDK<br>SLAVWSTSRPRPILVA<br>FDIANKSITDMSWNP<br>DGSLLFVASLDSSITLF<br>KFENNELGKPIPLEKN<br>MEQLYRYGVDKDSLD<br>FPESINQLLLEDQTKS<br>FKHTKISTSKLGENHP
NP_014681.1	ScHIR2	S. cerevisiae	HIRA	NALAALGPYIILAGSG<br>GHVMAWRQQQLVD<br>TAFDRVMIKDLKPEV<br>SFQVDQDTTGDIFFIT<br>GDLETLYIGSEHRLW<br>GYSGWLCRDTNNINS<br>VEKMNSKLLFECKSPS<br>TITDVKYDINLGILFVL<br>LSNENKILLFRHKTFD<br>KLSEITIDKASKPITGII<br>DPTGQTFTVMTSDRS<br>ILVYQINKTGTHKLIN<br>KLTQHVQMYPLHYRI<br>SMSPQADILPVINSV<br>KGVPNNATSCTALLD<br>RNNNYKVTKTLVTPS<br>SNGCRVLVYSPAFYEK<br>PNLKKGTSTRYNLIAT<br>SGSTDGTILVWNTKR<br>MKPLFNALQVSSTAI<br>NDMSWSQDGFTLFA<br>ISNDATLYTFAFQEKD<br>LGVALPQTEIKSLQEV<br>NKKLPKLEEPLAEQIP<br>KSFPENIKLEESASAA<br>PIPNDIGRSAVGKKPT<br>KKKTANNQTNGIKTI<br>QSTSMEFNTPSYTVP

XP_001014644.1	TtHIRA	T. thermophi	HIRA	EEIIKSINFQNQGNVL<br>VFCSGQDNDQLKIVK<br>TTINQDYMNQNVTN<br>KVDFQPLNIRSVQSS<br>NLIKWSPNDQFLAAA<br>FEKLGIWGYANGDIY<br>KLKDFKDHELEIAAIT<br>WAPDSSRIASGGLDG<br>KIVVRSVDIAKNYEKI<br>KELSHDNKILGLVWD<br>TYDKYLAALYSDNNVI<br>IWKCNSWEKSFIVSLT<br>CPISSDISKITSKRDDR<br>KIDWSPDYRYVLVPSL<br>DDKIVPFVCALDRQK<br>DFKVCKTFMGPFSSI<br>NCVKFNPNLYENKES<br>VINVFALGDNDGNISI<br>WGIGDGYQAKKPFFL<br>FKSHPNGNELIEDIC<br>WNAEGNMLIATTLK<br>KYVFLALFGETVFGSK<br>LSPQQKQDFLEGKYG<br>TLRTVAATNMKFKVF<br>KATQQEEEEEQKNKE<br>KQLFEAKGDNKQIDSI<br>LGRGNNNGKTATQF<br>GSSNSTNQNADQNQ
Q8QFR2	XlHIRA	X. laevis	HIRA	PIFSVDIHPDGTKFAT<br>GGQGQDSGKVVIW<br>NMPPMLKEEDEKNE<br>NIPKMLCQMDNHLA<br>CVNCVRWSNNGAYL<br>ASGGDDKLIMVWKR<br>SGYIGPSTVFGSSSKL<br>ANVEQWRCLSILRSH<br>SGDVMDVAWSPHD<br>AWLASCSVDNTVVI<br>WNALKFPEIISTLRGH<br>SGLVKGLTWDPVGKY<br>IASQADDHSIKVWRT<br>MDWQLETSITKPFDE<br>CGGTTHVLRLSWSPD<br>GHYLVSAHAMNNSG<br>PTAQIIERDGWKTN<br>MDFVGHRKAVTVVK<br>FNPKIFKKKQKNGSST<br>KTSCPYCCCAVGSKD<br>RSLSVWLTCLKRPLVV<br>IHELFDKSIMDISWTL<br>NGLGILVCSMDGSVA<br>YLDFSQDELGDPLSEE<br>EKNNIHQSTYGKSLAI<br>TTEPQLPNTVIENPE<br>MLKFQQRQQLQQD<br>GEHMVQAQMEAPI
A0A3L6FHG3	ZmHIRA	Z. mays	HIRA	FSIDIQTGGLRFATGG<br>GDQKIWASVLPAGR<br>PVPKKERVSGGMKH<br>GGEKQVCGAQLGFR<br>PCFMAHLAPPPQLLA<br>PSSPQPLPALASAAAS<br>NAASVRLAKAARLVR<br>LAKAARLVRLAKAAR<br>PLQLCLHRRPRLTAGS<br>PPTRTMGASCSFTITT<br>PLPPACTSAVSPPLPL<br>LFYRQTPATKIILSLEH<br>PATTRPPVPSLLQAPK<br>RLQKKALDSQSEGML<br>ACCVISLLRKMVRIW<br>SMESVHKDNANNDS<br>NQRLLATLRDHFGSV<br>NCVRWAKHGRYLAS<br>GSDDQVILIHERKAGS<br>GTSEFGSGEPPDAEN<br>WKVIMTLRGHTADV<br>VDLSWSPDDSTLASG<br>SLDNTIHIWNMNNGI<br>CTAVLRGHTSLVKGV<br>TWDPIGSFIASQSDD<br>KTVMIWRTSDWSLA<br>HKTEGHWTKSLGSTF<br>FRRLAWSPCGHFITT

NP_173580.2	AtUBN1	A thaliana	UBN	ELLRASPKVLTAGDRK<br>LLKVELRPGDTTYVS<br>WKKLMRDAGKVNGL<br>SASVPDPPPNANPNL<br>EFRIAPGHPVEIETNE<br>QPHSNRFNAVIEKIER<br>LYKGNDSSDGEELDG<br>APDDDEYDTEDSFID<br>DAELDEYFEVDNSTV<br>KHDGFYVNRGKLER<br>MEPSTTSNQQPKKR<br>RRKDSAKPCRDAVDV<br>SDKHTKLSITARKKDQ<br>STAPGSWKTQESPLP<br>SGAQDANTSVPLDD<br>VKHSDRANHQSRND<br>TSHKSRETGSSSALHQ<br>KYSNKSLHQQSTSLLG<br>KSPPNVFAEVTVVRQ<br>KENNGMHQLANVT<br>GSRQSSQASKKDGSN<br>VKSKTSILEKAIRELEK<br>VVVESRPPAITENQE<br>ADTSSQAVKRRLPRD<br>VKLKLAKVARIAQAS<br>QGKHSTELINRLMSIV<br>GHLIQLRSLKRNLKIM<br>IDMGDSATREKDTRF
NP_177855.4	AtUBN2	A thaliana	UBN	NESCKISSEILTAGDRK<br>LLKVELLKEETTLVSW<br>KKLMDEASKENGGLF<br>VSAPERLLNANPNLE<br>FRLAPGAQTENEMV<br>NQPHPNRLNSVIAKI<br>ERLYMGKDGSDGEEL<br>DGAPDDDDYDTEDSF<br>IDDAELDEYFEVDNSP<br>IKHDGFFVNRGKLERI<br>EPSATSNQQQPKKRR<br>RKESAKPCGDVVDVS<br>RKRAKMAKTAGGKD<br>QSASPGPSSKKISNDS<br>KTVQDSFSPLKAQNG<br>NDSLVLENVKHTDKA<br>NHQPMNATSPKSKA<br>AGSSGPLHPKCSSKS<br>VHEQSNSPPGKSRPN<br>VSAKSAVVRQQVNN<br>GMPDLDIATESKTSIQ<br>ISKKSGSNGRPKYSTL<br>EKAIRNLEKLVAESRP<br>PAATENQDADISSQA<br>VKRGLPGDVKLHLAK<br>VARIAYASQGEISGELI<br>NRLMGIVGHLIQIRSL<br>KRNLKIMIDSIVTANR
XP_006847173.1	AtrUBN	A. trichopoda	UBN	SVSPSGAVASSSLYDP<br>KGAISQRQSFSVDLR<br>PGETTIVSWKRLVKD<br>SNKAIRNSASVEPPV<br>GAHPALESRIAPEGH<br>ARHELEDVVPPSNRF<br>SAVIQKIERLYKGRESS<br>DEEDLDDIPDDDQYD<br>TDDSFIDDAELDEYFQ<br>VDKSETKHNGFFVNR<br>GKLEKTNDPISSPVHA<br>PKKRRRRDLNNAISE<br>NAAENLPKRHLNVG<br>GVRMKAAARNAPLV<br>GNKLSVPSQTAKTSV<br>GGELGHDIKSSQNQL<br>IACVETSKQKPLDSS<br>MKQENSISTKLPNKDI<br>SMEDKDGSKQKPGLL<br>PSGEGASKLTVNKEH<br>AHPIDRDLLDKGNPV<br>QVESQHKKALKDAK<br>GLLPSSKIRPKEGVGS<br>SDMRDTHILASKSPL<br>QKMKLPSLASKEGAA<br>VRPKGTMLERAIRELE<br>NKVAELRPPAMDLQ<br>EVDASAQGIKRRLPQ

NP_001255177.1	CePQN-80	C. elegans	UBN	SAKNEVIQIRISVAKP<br>GSKKYTHVDWEELLK<br>NNGKERDEDDLRRFY<br>DEDTLFMAKKLGETR<br>SKSGKKLRVNLDQLQ<br>HFNRKCGYDMDDDF<br>IDDTEIVDDEPTTSKK<br>GGFYVGKGDVKDSN<br>DEDEEEEEETDVEESI<br>QEVPKKVKIRKIKEKS<br>GEKSSKSSDSSSSSSS<br>DSEDAAGPPRMTGA<br>PPSIRMVSSSSSLKKL<br>PAPRKPSPPPAVEPT<br>RSVEIIEKPEKTEKPAE<br>KRKTAATTSALEPAEK<br>KKKPDSSSSSEEIILIDD<br>NAPAAKKQPESAQK<br>GSTTVTMSAKKICKQ<br>QEEIAKQKPSSSSVTP<br>SKPAAPTASTPKKEEI<br>GTLETFLKNIFANLDA<br>KGKLYQEKKLKTFDTE<br>TLKEITTFIDVMKHHK<br>TEKVNMEPLIYSLAKS<br>FGMTTQEVMKQVE<br>QEISSKKATTPKPSTN<br>AVLSTISSQKSLEKNA
XP_645639.1	DdUBN	D. discoideur	UBN	NKISNGNSFFSPNLFT<br>FSSSKTLTSRILNFKNN<br>NNNNNNNNNNNN<br>NNNNNNNNNNNN<br>NNNNNNNNNNNN<br>NNNNNDYNDNSSNE<br>EENEYSIIKRKKKIKIAP<br>TISKLLSIKKSEFNSDD<br>NSDEESYEDDFVDYE<br>NENENENENENEYK<br>NENENEDNKNEDNQ<br>DNDLAPSSSKLFRFLC<br>TPKIQTNIKINNNKNK<br>NKNKGNNDSTKKVD<br>SEINEKEEDSNAELKS<br>KPKAIRIEVDLKSNGIII<br>NFQQLVSNESNPLIS<br>NNNNNNNKSLISQT<br>NNFVKSFKNENISNIT<br>FNERKNVLGMVLIKI<br>GAGAKNNSSTRVEQ<br>KVEKEEERGEEEDFHY<br>YLDSDGDSTISEYDIE<br>DPFVDDSLLLIKDHAH<br>SDSNGIDDDSDDINN<br>LETNGFYVIQNGIDLK<br>FNNNNNNNNNKNN<br>NNNSGGANNNNNN
NP_476893.1	DmYem	D. melanogas	UBN	QGDAGFSRFGSNILE<br>PDAASAAPDNSSKPT<br>TKTAKCIRIKLDLFETD<br>SNKYPEFNYSRLLYLE<br>KKKTKKLKQVSTTNG<br>SASTDPFADNDDDV<br>ARIVKELEAKYGNSYA<br>TGRGKSKKDDYRDIG<br>MGYDESDSFIDNTEA<br>YDEIIPEEAETLEGGFY<br>INCGALEFKNLTKKSY<br>TTRTDAIIKMPERSRK<br>RMVSSSSESSSSSSGD<br>DDENDDGNNEEDDE<br>SDSEDDSEENDESDS<br>EDDSESESLEDEDSAA<br>TAKSSSKYKDNHQAK<br>RAKVIVTGKSKPSSSS<br>LTSGKKPPTKPITTSSS<br>SNSPRPSTVEISDTED<br>GQDPIQTQPSSQLQS<br>LPQSQAQAQALKKV<br>VKTTTVKDMLKAKRD<br>SFLKSQSGTAAVKGV<br>GNGELKCVSTDVSSS<br>DSSDMESEHGRADR<br>QAGQHGKDGQENLR<br>TADTLLPTTLDADIVT

A0A0J9YIW0	DrUBN1	D. rerio	UBN	EAYDELVPACLTTRYGGFYINSGTLQFRPASDEGENENDFEDSGLKPKKRKLKQGKDKKIMKKKRDEYGLAKKDEEEFTKSTPPDKSSAKKKKNKKPLSIDKMLKKFHKEKLQQMKEFNSREKELHSLNAIHAGPAIQAEIQEAHVSSDPLFSLIGSASADDLLQAVKAAEQDFDLDDLLGEPQNICPPGLEEVNGEALVVSVAEKPPALLPDGLPPMLEQHIKDISQAAKNIEGQNKMEILSSELNSVLLDVEVNSKQLGGKVRSRIFSYLASQLSCSKGTLVKRAKKLHSQQQDEQMQELLKKLENAVARSMPEQITRFQNHCQAHSEARAAKLEAEKERAIDGSDEEEEERSGKRVFGPRKRFRWNEEIRELLCEIVNVKMMIYDSESPSCSSLEEYLKAFLEADVKSLWPKGWMQSRILLIETRKAHGHIT
NP_001073638.1	DrUBN2	D. rerio	UBN	AGGPGADNRRRRLEEDGAAEMSFDREGRMKMMGTGVTTAGDRGGLIGKRDSEEPKGKTVRLNLCLSEPSEQCSAEFNYSELIQSQQPKKNPPCSKSSLDPNDPFNDDEKERLQVEALAKKFESKYGNAGKKRRKDRMQDLIDIGFGYDESDPFIDNSEAYDELVPASLTTKLGGFYINTGTLQFRAASESEGEDKVKDEKPRIRDGEEPLMKRRKKKDVIGIEEKKPRKNKVAKQGVAGLSLHRPEKKKRKKLMKDSLCLAAMLRRFTREKEEIRKQDISSSHLGLTSAPNSNNLLQNSHLHIGHANHTDLSLADLTADPAMMSLLSSANESELQDIMRDLDFSSLDAAPQTPPSCRENGQVGLGSSLGNKIGVGGLNRGQAGVVSFPTLPNGLPAPLIKRIEDLRAASRQFDQEGRKKFFT
NP_001072982.1	HsUBN1	H. sapiens	UBN	LNPAFLKKSRKEEAGAGEQHQDCEPAAAAVRITLTLFEPDHKRCPEFFYPELVKNIRGKVKGLQPGDKKKDLSDPFNDEEKERHKVEALARKFEEKYGGKKRRKDRIQDLIDMGYGYDESDSFIDNSEAYDELVPASLTTKYGGFYINSGTLQFRQASESEDDFIKEKKKKSPKKRKLKEGGEKIKKKKKDDTYDKEKKSKKSKFSKAGFTALNASKEKKKKKYSGALSVKEMLKKFQKEKEAQKKREEEHKPVAVPSAEAQGLRELEGASDPLLSLFGSTSDNDLLQAATAMDSLTDLDLEHLLSESPEGSPFRDMDDGSDSLGVGLDQEFRQPSSLPEGLPAPLEKRVKELAQAARAAEGESRQKFFTQDINGILLDIEAQTRELSSQVRSGVYAYLASFLPCSKDALLKRARKLHLYEQGGRLKEPL

NP_775840.3	HsUBN2	H. sapiens	UBN	RREAEYPGPEREPEYP<br>REPPRLEPQPYREPA<br>RAEPPAPREPAPRSD<br>AQPPSREKPLPQREV<br>SRAEPPMSLQREPPR<br>PEPPPPFPPLPLQPPP<br>PRESASRAEQPPRPP<br>RETVRLELVLKDPTDE<br>SCVEFSYPELLLCGEQ<br>RKKLIHTEDPFNDEH<br>QERQEVEMLAKKFE<br>MKYGGKPRKHRKDR<br>LQDLIDIGFGYDETDP<br>FIDNSEAYDELVPASL<br>TTKYGGFYINTGTLQF<br>RQASDTEEDDITDNQ<br>KHKPPKVPKIKEDDIE<br>MKKRKRKEEGEKEKK<br>PRKKVPKQLGVVALN<br>SHKSEKKKKRYKDSLS<br>LAAMIRKFQKEKDAL<br>KKESNPKVPVTLSTPS<br>LNKPPCAAAALGND<br>VPDLNLSSGDPDLPIF<br>VSTNEHELFQEAENA<br>LEMLDDFDFDRLLDA<br>ASDGSPLSESGGENG<br>TTTQPTYTSQVMPKV
NP_080942.1	MmUBN1	M. musculus	UBN	LNPAFLKKSRKEEVG<br>GTEQHQDCEPAAAA<br>VRITLTLFEPDHKRCP<br>EFFYPELVKNIRGKVK<br>GLHPGDKKKDVLDPF<br>NDEEKERHKVEALAR<br>KFEEKYGGKKRRKDRI<br>QDLIDMGYGYDESDS<br>FIDNSEAYDELVPASL<br>TTKYGGFYINSGTLQF<br>RQASESEDDFIKEKKK<br>KSPKKRKLKEGGEKIK<br>KKKKDDTYDKEKKSK<br>KSKFSKAGFTALNASK<br>EKKKKKYSGSLSVRE<br>MLKKFQKEKEAQKKR<br>EEEHKPVAVSSIEAQ<br>GLRELEGTSDPLLSLF<br>GSTSDNDLLQAATA<br>MDSLTDLDLEQLLSES<br>PEGSPFRDMDDGSD<br>SLGVGLDQEFRQPSS<br>FPEGLPIPLEKRVKELA<br>QAARAAEGESKQKFF<br>TQDINGILLDIEVQTR<br>ELTSQIRSGVFAYLAS<br>FLPCSKDALVKRARKL<br>HLYEQGGRLKEPLQK
NP_796159.3	MmUBN2	M. musculus	UBN	RREADFAGAEREPPR<br>LEPQPYREPARAEPA<br>PRADAQPPARDKPLP<br>QREVSRAEPPMALQ<br>REPPRPEPPPPPLPLQ<br>TPPPRESASRAEPPPR<br>PPKETVRLELVLKDPT<br>DESCVEFSYPELLLCG<br>EQRKKLVHTEDPFTD<br>EHKERQEVEMLAKKF<br>EMKYGGKARKHRKD<br>RLQDLIDIGFGYDETD<br>PFIDNSEAYDELVPAS<br>LTTKYGGFYINTGTLQ<br>FRQASDTEEDDFTDN<br>QKHKPPKVPKIKEDDI<br>EVKKRKRKEEGEKEKK<br>PRKKVPKQLGVVALN<br>SHKSEKKKKRYKDSLS<br>LAAMIRKFQKEKDAL<br>KKESTPKVPVTPSSSS<br>LPKPPCVTTALGDDIP<br>DLGLNSADPDLPIFVS<br>TNEHELFQEAENALE<br>MLDDFDFDRLLDATS<br>DGSPLSESGGENGNT<br>THPTFPSQVVPKVVP<br>TLPEGLPVLLEKRIEDL

XP_024392537.1	PpaUBN	P. patens	UBN	TTVVSWKKLVSDAEG<br>KLVSARVSDAPAGAN<br>PALEARIAPEKGAGS<br>VTGNDDPLPQVPNR<br>FSSVIERIERLYKGGDS<br>DDDGAEDTPDEDQY<br>DTDDPFIDDEELNEYF<br>MVEEAKTKHTGFFIN<br>RGALEKISVPSPSPAV<br>QPKKRRRRESKKDKK<br>DEKEVKRRMKGRIM<br>QGSRQFPSHSHNFA<br>GGETQVSNEHTLNKA<br>QSSDPEEISSDSDAFR<br>QGSAGPSRVSYKQEK<br>LDFADVDNNGGVVE<br>RLLETTAMLNTESVF<br>GGGRNIQIKSEYRYGS<br>SFEEKKWKQAAREPK<br>GVMKVSESLKMKMI<br>QQSGKEDGAKDSDG<br>GAGHAVAGYTKSLSP<br>GPKEPSPGRRGWPK<br>GTVMERAIQDLIKMV<br>ALLCPPSPEGEEQEAL<br>SQPGAKAKRLPREVK<br>TRLAKVARLAQAKQG<br>KVPDELVERLHGILGH
-	PpUBN	P. polycephal	UBN	CYRFSISLSETKGVVN<br>YADLMSQERKKRHLL<br>SNPPSDPSTNPTTTTT<br>TSEDTPKTPPLNGSV<br>DEASIDTAGDESVEA<br>ADESVEVDEGGNAS<br>ASKDVKKSSPKRSMP<br>VLPKKKTTNRLVQIID<br>KIEKMYTTGGDSFMN<br>GYDTSDSFIDDSDMV<br>EKPTEEVENEYGGFFV<br>HSGELKTRKKKRVSDF<br>QEDSPDKNKSKRTKK<br>LKIEDEKGSPKEKDKK<br>NDENGKKSKDKKPKD<br>KKKEETPKDKEKKKED<br>STKDKEKKKEDPGKE<br>KEKKKEEKEETKDSEE<br>EKAALRQKLLQTEEKI<br>EQAIKELETEAKKKIVP<br>GQTKRIPAELAPQLS<br>NLYEALQQTANGFIT<br>DRLLDRLVEFLPFKKS<br>TIKMHLKKSKHKAAV<br>DTMEVELKRLEDAYD<br>TEYNLLRAAVFNTMA<br>EEQKKQQQNPPPTD<br>PPADPSNPAEPKKYA
NP_009774.4	ScHpc2	S. cerevisiae	UBN	KQTKSSGKMQTQTD<br>TNAEVLNTDNSIKKET<br>GSDSEDLFNKFSNKK<br>TNRKIPNIAEELAKNR<br>NYVKGASPSPIIISGSS<br>STSPSGPSSSSTNPM<br>GIPTNRFNKNTVELY<br>QHSPSPVMTTNKTD<br>TEEKRQNNRNMDNK<br>NTPERGSSSFAAKQL<br>KISSLLTISSNEDSKTL<br>HINDTNGNKNSNAA<br>SNNIPSAYAELHTEG<br>NSIESLIKPPSSPRNKS<br>LTPKVILPTQNMDGTI<br>AKDPHLGDNTPGILIA<br>KTSSPVNLDVESTAQ<br>SLGKFNKSTNSLKAAL<br>TKAPAEKVSLKRSISS<br>VTNSDSNISSSKKPTS<br>EKAKKSSSASAILPKPT<br>TTKTSKKAASNSSDST<br>RKKNASNKTTSAIKKE<br>SNAGSKLNTVKKENS<br>SLSSIKATEKEKDKGG<br>NSTEAKNSTSNVRKE<br>PTAKSPKRLVAAPTVS<br>PPKILQTAETKAKEPSI

XP_001017549.4	TtHpc2-1	T. thermophi	UBN	PNFGFCQDKQIFQH<br>DLNENNINQQHQKIL<br>SKIEEKPNIKQNKHILE<br>RMHDEQSHDEEHEL<br>RGYEWIQSTNEEDFQ<br>IMKNVCYLEQFLDMY<br>DDLEEEDFLHLDIPQF<br>MQYLENSESESESIQS<br>DEEEMEDQFEDNDLI<br>QNVNTHRYSESRLLN<br>TNLKNEQGVKKTDEF<br>QKFDQPANNAESTM<br>NAEAVVKNNKIITNM<br>KNKGKNQMSQNSID<br>SQREDDIYSQINQKG<br>KAKRYQSSLALLAQE<br>QFIRISKYGDIALTSNI<br>ARKNPKSDDFYDQED<br>SFIDDDQVDTELKQL<br>AICEADYDDFFMVEG<br>GLQDLVKSSEFRERLK<br>DIQEYRDTKKIENHLK<br>TDKKEKQPNPKIKISS<br>ENSKNLSFNEKKQQG<br>LNLLMDAEDEEDDK<br>QIKKKKKNAAKNKKQ<br>FDLLHLKQPTFQEEM<br>LQNAQNTKIQPAKT
XP_001010706.2	TtHpc2-2	T. thermophi	UBN	GEDVADSNSSDIMEE<br>EENEVDKPSIAKFSNL<br>NNNNNNKLDQVNG<br>NKNSVNGSADKKKK<br>KQRSTNKVSIEELTKQ<br>IKQNQSKRVNGQED<br>GVVIKANFDWVNYN<br>TTEDKTLNYLSDFLKL<br>NGENNLDYFKLDLVE<br>FEDVNIVQHHSQVSS<br>ITDDLYKRILRYGQIEN<br>IQRIAKKSGAISKGEG<br>TNVNFRDELYYDQND<br>PFIDDDEYNNTSVKV<br>SHRMQLAEVFIDDFR<br>TFDGDLKDFIKSEIYK<br>NRVEIMHNLEKKNEI<br>EEETENFNKKKKKPN<br>PPSNPAISSSNNLSSN<br>LANNSLTAALNSATA<br>TLNNKIQKKATADVE<br>KDLNQVESVKTQEKK<br>KFKKDNVNNTTSLSS<br>GQQLQNAQNILQAA<br>GLNQNLQSVSGSVN<br>AQSLPQAVQASNLLL<br>GADLSTSQLSQLLSQ<br>AQSSNNNNLLQNAA
XP_018092158.1	XlUBN1L	X. laevis	UBN	AVKRQRSEEQESETS<br>VAATVRISLTLFEPDQ<br>KRCPEFCYPELLRNLR<br>GDGKKGLHGDKSQK<br>KVVSVISEEEAEKKEV<br>ANIAKRFEEKYGNKK<br>RRRDRKQDLIDMGY<br>GYDESDSFIDNSEAYD<br>ELVPASLTTKHGGFYI<br>NSGTLQFRQASESED<br>EVVKEKKKKKSPKKM<br>KDRGEKVKKKKRAEE<br>KKCKKNKHPKPGFTA<br>LNGTKDKKKKKHSAIF<br>KMLARFEQEKNAQK<br>NIITPPSPTPTLKPSSIS<br>QQPILSQPTPQEAEP<br>APDAAVSTHVSELLK<br>AVSAIDSVREKQMEN<br>IHNGPPEKKHGVQG<br>GSTSEEAIKTPASLPD<br>GLPHALEKRIKELTKA<br>VKASEGEKKGLLFSQE<br>MNSALLDIYILSRELSS<br>SLRLAVFTHLASVLPC<br>GKDTLVKWATRLYLQ<br>KQGGRLREPLRKLKE<br>AVARTMPEQITKYHE

XP_018116614.1	XlUBN2S	X. laevis	UBN	KPRETGPVLSVEQQN<br>QQTPPPLQPPAQRP<br>PQPRAAHQVMFQPR<br>PPPQALGHRLQREPS<br>SPALEAGICHVLDKP<br>QGDDTHQLAGTVRL<br>QLALTDPTEEGCAEFS<br>YPDLLQKEQKAGRALI<br>PVTRKETAGLSKPPAS<br>NDPFNDDERERQEVE<br>NLAKKFEAKYGGKSH<br>HHRKDRVQDLIDIGY<br>GYDETDPFIDNSEAY<br>DELVPASLNTKYGGF<br>YINTGTLQFRQASDSE<br>NEDFVENKKHKTSKV<br>SKIKDGERSLKKRKRK<br>EENTEKEKPKKMKAA<br>KQIGVMAFNSHKPD<br>KKKKKLYKDSVALAA<br>MLRKFQKEKDAIHKK<br>VNKPNPPTVTVVNTA<br>PKLVTAASNDLSDLGL<br>GADPVLAIFGSEREL<br>MQEAESALEMLSDF<br>DFDKLLDSASNDSPA<br>GSGENSRVGQSLNPS<br>EVQLPKQVPPLPEGL
XP_020405992.1	ZmUBN	Z. mays	UBN	AAAAAARVPGAVAA<br>APGVTSAPSALAPTQ<br>PPATAAAVSEAATPC<br>QRQLFTVELRPGETTI<br>VSWKKLLKEAGHTAA<br>APPVAVEPAFAAHA<br>GPSGPAHPAENDPK<br>DPTQPNRFNAVIEKIE<br>RLYMGKHSSDEEDLN<br>DVPDDDQYDTEDSFI<br>DDAELDEYFEVDNLK<br>TKHDGYFVNKGKLEQ<br>IEPGTAANVAPKKRR<br>RKDASSSYLESNHLGP<br>IDYFDIGDVPEKSSAR<br>GTVQTGKQLVSSNVS<br>SYGQYHEDNRVVKN<br>KISGPGGAPKRKSSEF<br>SGDAAARAKIIKDVS<br>HAPLELRDMEKHKA<br>AALPVDYAHKSKISET<br>FDYAYPAYRDKGTSG<br>QLDFQQRKISRENQG<br>PSNRTYRKEKHGTNE<br>YPGIAMATAVYSTQT<br>MHPVVGREGSGTKP<br>KGTRLERAIRDLQKIV<br>AEYRPPTIDINEVDPN
NP_195008.6	AtCABIN1	A thaliana	CABIN1	WEPLAPSKEAQEFHL<br>SQTYHDGLLKLQAKD<br>YDKARELLESILKDPIIT<br>NSKVETIANDNHLHH<br>LRFLALKNLATVFLEL<br>GSSHYENALNCYLQA<br>IDLDAKDSVLWNHLG<br>TLSCSMGLLSISRWAF<br>EQGLLCSPNNWNC<br>MEKLLEVLIAVGDEVS<br>CLSVANLILRHWPSH<br>SRALHVKHCIEDTDS<br>APFAPKGIDKLEPQH<br>VRLKFLGKRKVSDMN<br>QDMDATSKKLRKRV<br>QFKLPEASWVALLNIL<br>IGIVHPSRETVGISADI<br>PITIELSLSTEAVMQG<br>MKKKDHCVDSDSSN<br>VSVKDCNIERESGGS<br>VKEKEPVFSEEHPQE<br>RRSTRLERLRNQKPEK<br>EGLEFDNSKDPSSDIL<br>QYLEKFVLKRGFDRES<br>AGSFCNEESDPISEHA<br>VVSNFVKENLENYGA<br>YHMGHLLLEYIANKC<br>EHVLSRETALKILELEK

W1P7Q2	AtrCABIN1	A. trichopoda	CABIN1	QWETLAPTKEAQEFH<br>LTQEYHEGLLQLQAK<br>EYSKAQRNFEAVLRD<br>PLMSNAQVDGNTRD<br>GHLLQLRFLALKNLAT<br>VFLEQGSMHYESAIN<br>CYLQAVEIDTKDSVV<br>WNQLGTLSCYIGLLSI<br>ARWAFEQGLSCSPN<br>NWNCMEKLLEVLIAI<br>GDEISCLSVANLILRN<br>WPSHARALLVKTVIE<br>ESERIPFAPRGIDRLEP<br>KHVRLKFPNKRKSIDE<br>PYDGADIKKKRNQTV<br>ELLLPEASLSSLVDAIL<br>KILRPLSAKGSENSPIN<br>EKQSRSAEFPSRNNE<br>LHGKGETDDLNMKG<br>RYTDMRMRINVSSSS<br>ENVGDPFIGKGMGM<br>LLAGNQSSTAEKNPE<br>KIFHGKEKEGSTDEEV<br>HPQERRSTRLERLRSR<br>KPEKEELDYPLSKNRA<br>EAAFQILKPFIVVGLE<br>KKDYNYNQTNTVLL<br>MDTLEFGVNDISRFF
NP_001299995.1	CePICD-1	C. elegans	CABIN1	LNEDDESSDGENSVII<br>LDSDTEPEDGAGGEE<br>DEDNGEDSEEEDEED<br>SDEEESEDDSDSSVDT<br>ESVNSVWSNVDEEN<br>GEEITTEAAEAKIIKKF<br>EKAIFSIADNDSETGR<br>RILEKLLNDAIIATYNQ<br>ENPDFELLDEPPRLAK<br>MLQVFIASHRNLAKL<br>CEKEVENADEKEKEQ<br>LKSEEIQHLCQVLAYE<br>PNSSDIWLDVALKSIE<br>FGDLNFSKYAFKRCET<br>LKESLESHATLLYLTCD<br>YNESLMIMKQFQEQ<br>NDVLNDKLKYLKHKIR<br>STNQYYKLSCDRIFEE<br>DEVYADVQSVEKRKI<br>QDFDERIEALRSRVV<br>GKIEQRDVDFRDEEE<br>KCSVIPIRIEADQDLQ<br>TVCTIFCDLFDRIHAY<br>SHCRHQLIEFTEWES<br>RRDHLDVLDTVEHLV<br>DVVECVEDISQKVEEL<br>ENLKRGAKGRRKKQ<br>QQKDEKKEYLASWR
XP_647263.1	DdCABIN1	D. discoideum	CABIN1	YSEYESEDSEYDDTDG<br>DDDNNINNINNNNI<br>NNNNNNDKFDNMSI<br>EAQETYNMKLYSNAL<br>KHQQNNDYLQAQQL<br>YQELLQSPMIERINPQ<br>SKKSILSSQMATLKYS<br>TLKNMANINEAIDNK<br>ELSLKLSTQAIDLDGR<br>DVSLWYSIGKISMELE<br>KWNISRLALERAIQIS<br>PTHWLSLEKLIELLYVI<br>GDDNSCKNIIEQRILQ<br>EQPSNLKAIIIKLLIQR<br>SNQSNYNEINEILNNI<br>LISNQNSKSYIQSLIKK<br>RDRIQQQQQQQQQ<br>QQQQQQQQQQQQ<br>QQQQQQQMIIGKQ<br>PQLTNELLDKPVEYIL<br>ENKNWSSLLDLLNSIY<br>NEKKESTSSLSIFSPIEI<br>KLHPSLLGIQILVLDG<br>NEDDVDVEMEEEEE<br>VDITPINTNVDNKKQ<br>PIAIEITDTSITTATVTA<br>TTSSTNLESSIRPATPV<br>TPTTPTINTSNTTTTTT
NA		D. melanogas	CABIN1	

E0YA53	DrCABIN1	*D. rerio*	CABIN1	EHEDAFRSTKPGQTK<br>EAQEAEAFALYHKAL<br>DLQKHDKFDESAKAY<br>HELLKTPLLKEAVASE<br>DEKVGLKHPGLMLKY<br>STYKNLASLAVLKDDL<br>DTAMDFYVQAVMLD<br>STDVNMWYKMGKL<br>ALRKVSMPLARHAFE<br>VGLHCNPEHWPCLD<br>SLITVLYALSDYSCCLY<br>YICKALEKDSGYSKGR<br>VLKEKIFQEQPCLRRD<br>SMKMFSKLDISLQYV<br>DVDEEEAHSIVEEALE<br>LRRQRQAKLTRHPVA<br>DLQLVQPIKYFTWKS<br>VGESFLAMYKHQNA<br>CLVPRPDFGRRIDLS<br>MYRDPDCLLQMPISA<br>PVPAAAAAPSDPPQ<br>PTVPISDPPAPVVLPP<br>APSSPSASLQLYDTVI<br>WYSRVNGALDDKVK<br>KGTKRKRVIEDTVETA<br>KRRSARVRNTKCKKE<br>EKIDFQELLLKFLPSRL<br>KKFEDDEDEESLSNM
NP_001186210.1	HsCABIN1	*H. sapiens*	CABIN1	HEGSFKSHKTQTKEA<br>QEAEAFALYHKALDL<br>QKHDRFEESAKAYHE<br>LLEASLLREAVSSGDE<br>KEGLKHPGLILKYSTY<br>KNLAQLAAQREDLET<br>AMEFYLEAVMLDSTD<br>VNLWYKIGHVALRLI<br>RIPLARHAFEEGLRCN<br>PDHWPCLDNLITVLY<br>TLSDYTTCLYFICKALE<br>KDCRYSKGLVLKEKIF<br>EEQPCLRKDSLRMFL<br>KCDMSIHDVSVSAAE<br>TQAIVDEALGLRKKR<br>QALIVREKEPDLKLVQ<br>PIPFFTWKCLGESLLA<br>MYNHLTTCEPPRPSL<br>GKRIDLSDYQDPSQP<br>LESSMVVTPVNVIQP<br>STVSTNPAVAVAEPV<br>VSYTSVATTSFPLHSP<br>GLLETGAPVGDISGG<br>DKSKKGVKRKKISEES<br>GETAKRRSARVRNTK<br>CKKEEKVDFQELLMK<br>FLPSRLRKLDPEEEDD<br>SFNNYEVQSEAKLESF
NP_766137.2	MmCABIN1	*M. musculus*	CABIN1	HEGSFKSHKIQTKEA<br>QEAEAFALYHKALDL<br>QKHDRFEESAKAYHE<br>LLEARLLREAVSSGDE<br>KEGLKHPGLILKYSTY<br>KNLAQLAAQREDLET<br>AMEFYLEAVMLDSTD<br>VNLWYKIGHVALRLI<br>RLPLARHAFEEGLRC<br>NPDHWPCLDNLITVL<br>YTLSDYTTCLYFICKAL<br>EKDCRYSKGLVLKEKI<br>FEEQPCLRKDSLRMF<br>LKCDMSIHDVSVSAA<br>ETQAIVDEALGLRKKR<br>QALIVREKEPDLKLVQ<br>PIPFFTWKCLGESLLA<br>MYSHLTTCEPPRPSL<br>GKRIDLSDYQDPSQLL<br>ASSVVVTPVSVVQPS<br>PICTNPTVAVAEPVLS<br>YTSVTAASFPLHSPSL<br>LDTGTPMGDVSGGD<br>KSKKGVKRKKTVEES<br>GETAKRRSARVRNTK<br>CKKEEKVDFQGLLVK<br>FLPSRLRKLDPEEEDD<br>PFNNYEVQAEAKLES

XP_024373289.1	PpaCABIN1	P. patens	CABIN1	FSEPLAPTKEAQESRLTQMYGEALSHLQHGQIEKAQSLFQSILQDPISIKAQVEPVSSANTMLQLRYSALKNLAETFSMQGSGHHVEAVDCFLQAAAIDGKDVVLWNRLGTLSCALGNLNVARRAFEEGLRCSPRHWSCMEKLVEILIAVGDESACLSVAKRLLKLSPSHPRALQIQHAIEQGVSMRPFDGPKSGHETSSVLKIGPRGFDLLQPEYFGLSFDNKRKLENAYETVQHQKKRKVHSIDVHLPEASWFALVNAVTDVLKGGRFWKDDEPSLNTQIVVSPGGNQSEQQKAASAGISAGLVNATVKFSVHKSHPIIVDLNSEYKEQVAGEEAELISEDCLNSSSTKTSEEVSREGLGVAPVLAEPRVESASENRAEGHKDVENASVHQKQQGTGEKQEASKLPDLMKPSDPTTEW
-	PpCABIN1	P. polycephal	CABIN1	KKHKTPTKGKDALEAENFALYEKALRHQYAGEYQEAEKLYKSILASPIVNEFVEVSKRDDLYYTLPVVTLRYLAYKNFASLCETTSRKTEAMEYYAKALDVDGRDVVVWYHMALVCMSVERLQLARFALEQGLKASPDHWLSIDKLAEVLFILGDYGACMNVVDRILELEPGFSKAILLKTKMMSENPDNKFKVEAFLWEAGDLGDCNIDDLVSARSKRQKVADFDHDTVEEPPREFVLKEKTWSSFADLLYDIYKEMTSEKGKSKFLNDPIYITDPAPLSPPGSPAASALNDSFPSLNFTTPSSGTDLSNSGSGTASSGTNQPGLNESATNLNTSATNLNTSATNLNTSTSNLNTSATGVNSGETEAPNVESGRKSTRGRKPAADSATRQSARVKEIKHDKISSDLDEYLNKLLNTQEESDETTASS
NP_012674.1	ScHir3	S. cerevisiae	CABIN1	QYEAEEHSRELQIEQSFNILQDALIDLKNKDFEKSDSKFQELFQIDVVKPDRWGMYRNSSPTLDNLRYLCYRNRGMYYHLYLENNYERLNSQELVNCILKAVENLVESIQHSDADFAVTDLLARIFKSFNSVKLERLISEYEFTKQENLSLLLGRHRKFLLNDLTLMMNNYVELTNKLLVPNLSDNTIFERYHLEKYKDIKPEPLAFGPILSRISEMKKQDEEIMKKLDVFNVTLNEESWDEVAKALKNLLPSVKTSSLIGRNMDPYNEIEEPIEAVKFELSEAINNTPSLDRESERQEEEQDNESVRADDKSGNLAPSDIQTNEEARPNKRTDEHIDSTKPLQRSSKRFKEREQENSKELVMDVHKRFFGEFNTLLSYIHILPFCDFDTFASKFIIGSSDKQPEKFIPYTDLYECLKSWSSRYTDIFNQNDYLSSGSNE
NA		T. thermophi	CABIN1	

XP_018117354.1	XlCABIN1	X. laevis	CABIN1	EHEETFKNHKTQTKE<br>AQEAEAFALYHKALD<br>LQKLDHFDESSQAYH<br>QLLETRLLQEAIPAND<br>EKEGLKHPGLMLKYS<br>TYKNLAQLASQRDDL<br>ETAMEFYIEAVMLDS<br>TDVNLWYKIGCLALR<br>LVRIPLARHAFEEGLQ<br>CNPDHWPCLDNLITV<br>LYTISDYTTCLYFICKAL<br>EKDCRYSKGLVLKEKI<br>FEEQPCLRRDSFRMF<br>LKCNMSIHEVDVSDE<br>EARTIVEEAMDLRRR<br>RQALTVRKAEPDLKL<br>VQPIPYFTWKCLGESL<br>LNMYRHLISCEPPRPS<br>LGKRIDLTEYKDPAVL<br>FHPPPVSSPTDIIVDP<br>LMISTPPTPVSVIEMP<br>SVLPTATPPASSSNVI<br>DTSAVQADSSTGDKT<br>KKGVKRKKISEDAGET<br>AKRRSARVRNTRCKK<br>EERVDFQELLLKFLPS<br>RLRKSDSEEEDDPFCS<br>FETQSEMKQEHYAN
A0A317YDT9	ZmCABIN1	Z. mays	CABIN1	WEPLAPTKEAQEFAL<br>SQKYHEGLLKLQQKD<br>YAKARELLEDVLKDPL<br>ISNVQADNIGSDQHL<br>LQLRFLTLKNLASVFL<br>QQGFEFYDNALHCYL<br>QAVELDSNDSVVWN<br>HLGTLSCSMGLLSVSR<br>WAFEQGLVCSPNN<br>WNCMEKLLEVLISIRD<br>EVACLSVANLILNSW<br>PSHHRALHVKKTIEC<br>AEPVPFAPRGIDILEP<br>KHAKLIFSNKRKSVDD<br>EICQEIGTKKSKQSAT<br>LQLNEAKWLALLDGI<br>LSLISANTEKAGEYNC<br>SNTVDRCSISEGSAKG<br>LAYNIIDVVISTDTLKS<br>VETAGGNGNDSHHG<br>EILPYDCKATVKEKDG<br>NSDREHPHERRSTRL<br>ERLRSRKSGKDENGS<br>DGKDISHAMTQFLES<br>FILKGPSPVEKAYCSG<br>NADASNPDTHNYTS<br>NEEANDLKQFLCKISK<br>NFGPHHIGYMLLEE
NP_974699.1	AtNASP	A thaliana	NASP	MVEESASASEASVIQ<br>TLTEPATEIAQTLEPN<br>LASIEATVESVVQGGT<br>ESTCNNDANNNNAA<br>DSAATEVCDEEREKTL<br>EFAEELTEKGSVFLKE<br>NDFAEAVDCFSRALEI<br>RVAHYGELDAECINA<br>YYRYGLALLAKAQAE<br>ADPLGNMPKKEGEV<br>QQESSNGESLAPSVV<br>SGDPERQGSSSGQEG<br>SGGKDQGEDGEDCQ<br>DDDLSDADGDADED<br>ESDLDMAWKMLDIA<br>RVITDKQSTETMEKV<br>DILCSLAEVSLEREDIE<br>SSLSDYKNALSILERLV<br>EPDSRRTAELNFRICI<br>CLETGCQPKEAIPYCQ<br>KALLICKARMERLSNE<br>IKGASGSATSSTVSEI<br>DEGIQQSSNVPYIDKS<br>ASDKEVEIGDLAGLAE<br>DLEKKAT

XP_006856185.1	AtrNASP	A. trichopoda	NASP	AVVVEMAAEPSASV<br>DGALSKGMDLGSQV<br>GEASGSQGIEGSSEK<br>ALEFADELMEKGREA<br>IRSDDFVEAVDCLSRA<br>LEIRVQHYGELAPKCA<br>MSYYHYGRALLYKAQ<br>EESDPLNVSVTMAKK<br>AASSSGPGKMDIDLS<br>KGKGVANGEASTAS<br>DGLKDSSNKGKESEG<br>SNGNEENDDESSEDD<br>GDGVEDDQGVDGTE<br>EEEESDLDLSWKMLD<br>IARVIIEKNPVDTLEKV<br>DVISTLGDVSLEREDF<br>ETSLGDYFKALSILESL<br>VEKDNRQLAELNFKI<br>CLAMQLADRPADAV<br>KYCEKSMSVCEARLQ<br>RLTSEVSESTAKTTSV<br>DNESFSVALEEDITKS<br>SEGNDSNTSSVQEKN<br>SEIETLKMLLVDLKEKL<br>EDLQQMMSSLTNPLI<br>QATEKLISTIAEKAKE<br>DSSMSSRMGTETCG<br>FDSPKLTTITTTTKSTE
NP_496380.1	CeNASP-1	C. elegans	NASP	MDTENIADASDIRVK<br>DASGDSDEKGNGTTT<br>EEETVEQKEKRLAELL<br>AAGRRALKVNDIDKA<br>SDSLSEATELSSEIYGE<br>NHENTFDSLYYYGMA<br>TLELAKEESQLLKGPG<br>EKESGDEEQAGNSDD<br>KTDEENGETEKEDGE<br>ESGEEEDDDDDTMKL<br>SWEILETARCIAAAKI<br>EALEAEQSGISAIEEW<br>NLKLADVLVLLGEHGI<br>SDGKYTQAFEDLDRA<br>LNIQRNVLPPSSRKIA<br>QTYILIGNACASDANY<br>DETVQYFGKTKDVLIA<br>RQTELKHELERGVDD<br>KEKKSEFENELKELEE<br>MMPGVEEMIADAV<br>HSAAQVEETKKAIKA<br>QFEGFTQVLAKLPQE<br>AGDQKEANDISSLVR<br>RPAKRAVDAPTDNQ<br>AVKKEKEEEGTTSI
NP_506298.1	CeNASP-2	C. elegans	NASP	ETIAKNVDSTGEEKVA<br>MTNVAEETTPSTDSA<br>TTNEKTASENSPTEA<br>AASDVKDVAEASTGA<br>QETNTSSETPAVVED<br>EKASMEVSIGTSIEDS<br>ETSQKEIEGELVVPEN<br>PVEEISPEQKRKSLSE<br>NIIAGRRFMRTDVFD<br>KAVDVLSVAASIAAE<br>VFGDAHEDTFEANFL<br>YGKALLEVGKLEDRVL<br>ANALTDVPKMAEGE<br>EEVQDGIVENPEDVP<br>QDERAEIKQKVEEAL<br>GVASEEPETVADEAV<br>KTEQKEAEEDSVEKD<br>VENSDEQNQEEEEVV<br>ENEATVDPTEDVEME<br>GVEEVNDEKEVGQT<br>AEVDGEAEEDDEAED<br>DEADSMKLAWELLET<br>SRCIADKKAASLAAES<br>TVDGEAIKMWKLNL<br>ADVLTSLGEHGIADSK<br>YEQAQKDLTEAISIQT<br>VHLPATSRVIANTTHL<br>LAKAFSSDSLFEQAAA

XP_629898.1	DdNASP	D. discoideur	NASP	ATTTTVTKKDEKSKVE<br>IGEGEAGITFEDYEEE<br>GEEEGEEEGEEEEEEE<br>EEEKDKETIIKSAEGN<br>EKAGDLLMKEKDYSS<br>ACGLYCVALKNLSSIY<br>GALSPKVAHLYVKYG<br>NSLILTYKQEESENLM<br>NLLGSKIKEIAEKGED<br>STTSSTTPPQTTTTTTT<br>TTTTTTTTTTTPTEDA<br>PDKNASDKKNSKKEE<br>EESEDISTEESSKPVNK<br>LNTKENEDDEATPIQI<br>AWEVLETARVIYEKEK<br>DKQIELSDVHVSLGDL<br>FIDSDQIENAIEEYDK<br>ALRIRKDIRDNDLRSI<br>AEIHYAMGLAFHVD<br>NKAPIAEKHYRTAIDL<br>LGDQLNKIQQSLNGE<br>TDTDKLKSLADEIQEL<br>KNIIVDLQGRLDEVVP<br>IDTSNEPVPKELQQD<br>EQQPVFSTSASQNPI<br>KPGTPVNTFGVVGKS<br>ARRSITPQTNNSNSSS<br>TPSTPNKFTPIETTTTT
NP_001246993.1	DmNASP	D. melanogas	NASP	VSSPSKTVAVEPVAA<br>DTTPDNAPAVSTEGS<br>GKAEQERAEKILKGKE<br>LFSQGSRNFLVKSYDE<br>AADELSQVCQLYEEV<br>YGELADELGQPLLLYA<br>KALIAMALDENKVID<br>VPDEAADDDDEDVD<br>DDEEESAEDGAAKKE<br>EKKDTKEAANGASSS<br>NGKELDTIKEGSDEA<br>DSTGEAEQAQSDEKP<br>SKKVPTGVDEVSSSN<br>GGGGAAVNDDERPS<br>TSNGEVTASCSNGAA<br>PAVEEEPEEEEGVSGS<br>LQLAWEILEAAAQIFS<br>RQGLSGLPYLAEVQT<br>ELANIEFENGILEAAR<br>EDYEKALKIHGELPTR<br>NRRALAELHYKIGLTY<br>LMQQLNKEGATALR<br>QSSVLIEEEIAEIKGKD<br>EPSERDRNNMLDLEE<br>TKQEILAKIQEIEEMQ<br>AQTIAEVRAALDSYIK<br>PMSSGDAAAASSSSS<br>SSANGAASSSSSSSKG
E9QE69	DrNASP	D. rerio	NASP	MPEETGATSTAERM<br>EEKPCSSSTGDSSVDV<br>AEEAKKLIGTGSRHLV<br>MGDVVSAVSVFQEA<br>CAMLAEKYGDTADE<br>CGEAFFFCGKALLELA<br>RMENTVLGNALEGV<br>PEESSEEGEKQDDSKI<br>ESADNLDGDDDGDE<br>DEDDDEDAEGDAKD<br>KESEEDEVGNLQLAW<br>EMLEVAKVIYKRKDD<br>KEDQLMAAQIHLKLA<br>EVGVESGNYSQALED<br>FNECLTLQLKHLPSHS<br>RLLAETHYQLGTTYSY<br>TTQYNQAIEHFSNSIK<br>VIESRLAMLQEVIDKA<br>EGEDSAKEEKGEFEEL<br>KQLLPEIKEKIEDAKES<br>QRTAAAASEAIHQTL<br>AGASTSSAFPTENGG<br>PSSSTASQ

NP_001182122.1	HsNASP	H. sapiens	NASP	LVSADKIEDVPAPSTS<br>ADKVESLDVDSEAKK<br>LLGLGQKHLVMGDIP<br>AAVNAFQEAASLLGK<br>KYGETANECGEAFFF<br>YGKSLLELARMENGV<br>LGNALEGVHVEEEEG<br>EKTEDESLVENNDNI<br>DEEAREELREQVYDA<br>MGEKEEAKKTEDKSL<br>AKPETDKEQDSEMEK<br>GGREDMDISKSAEEP<br>QEKVDLTLDWLTETS<br>EEAKGGAAPEGPNE<br>AEVTSGKPEQEVPDA<br>EEEKSVSGTDVQEEC<br>REKGGQEKQGEVIVS<br>IEEKPKEVSEEQPVVT<br>LEKQGTAVEVEAESL<br>DPTVKPVDVGGDEPE<br>EKVVTSENEAGKAVL<br>EQLVGQEVPPAEESP<br>EVTTEAAEASAVEAG<br>SEVSEKPGQEAPVLP<br>KDGAVNGPSVVGDQ<br>TPIEPQTSIERLTETKD<br>GSGLEEKVRAKLVPS<br>QEETKLSVEESEAAG
NP_001074944.1	MmNASP	M. musculus	NASP	VSADKIEDAPAPSTSA<br>DKMESLDVDSEAKKL<br>LGLGQKHLVMGDIPA<br>AVNAFQEAASLLGKK<br>YGETANECGEAFFFY<br>GKSLLELARMENGVL<br>GNALEGVHVEEEEGE<br>KTEDESLVENNDNVD<br>EEAREELREQVYDAM<br>GEKEAKKAEGKSLTKP<br>ETDKEQESEVEKGGR<br>EDMDISEPEEKLQETV<br>EPTSKQLTESSEEAKE<br>AAIPGLNEDEVASGK<br>TEQESLCTEKGKSISG<br>AYVQNKEFRETVEEG<br>EEIISLEKKPKETSEDQ<br>PIRAAEKQGTLMKVV<br>EIEAEIDPQVKSADVG<br>GEEPKDQVATSESEL<br>GKAVLMELSGQDVE<br>ASPVVAAEAGAEVSE<br>KPGQEITVIPNNGPV<br>VGQSTVGDQTPSEP<br>QTSAERLTETKDGSSV<br>EEVKAELVPEQEEAM<br>LPVEESEAAGDGVET<br>KVAQKATEKAPEDKF
A0A2K1IFP3	PpaNASP	P. patens	NASP	TQGSAAVDLEEAQRL<br>FDDGCEFMHNEAFE<br>DAAECFSKALEIRVRH<br>FGELAPECATTYFKYG<br>CALFDKLQEEADPLG<br>ETVNPNPALPSSHPE<br>GSSGKEPEVTGKDPV<br>EEAGGDDDGDEDDV<br>MGEGEEGKEEEEGDL<br>EDAWKMLEFARVIHE<br>KLDSRTIEAVDIITKLA<br>DVNCFKENYEACYED<br>YAKALEMLKQIVEPD<br>SRILAELYFKIAIAKQL<br>DLKPKEALIYCSNAVA<br>VCEARVQRLKHEINN<br>VHTEGVDALGTSEAA<br>SVVDVEHSEPQTNGS<br>MKATEHDNDEVINP<br>PTEEKSAAESKVNEIK<br>DIEDLLVDLKDKEQEL<br>KEMASAPTLVEQLQA<br>ANPEAAASMKQLFSL<br>AAGSQASDSGGSSSS<br>TADGIKTSNGFDQPT<br>LANFTSSTAVVTDLG<br>VVGRGIKRAEPVAVV<br>PAGIVNSSQPSKKRSF

-	PpNASP	P. polycephal	NASP	NTTTTTTTTSTTTSPQ<br>ETAVEAGASSSEPKEL<br>TPEEAAKKYEEAKALF<br>NEDKYAEASDLFADV<br>LTHKVAVHGELGLEC<br>AECYFLYGKCLFLQAK<br>AESSVFGPTAQQKIN<br>EDDEDSSDEEDVPPV<br>GEGGEKTEGKELEEK<br>PEDVENTQVAWETLE<br>VARLIYEKHPEKAAEL<br>SDTRYALAEIFLETDN<br>CNDAIEEFKKCVSLRE<br>SIFVPNDRRIAEAHYH<br>LGLAYFSTDRPADAH<br>FHYSKAADVLDSKLKS<br>LPPGSAEIEDIKSILED<br>LAAKMEEVDPSLLAK<br>EKEATENAINGTTNG<br>KGKAPVSSSSSSAPPS<br>SFPKEETGFAAPIFDK<br>TQPVTRFGVVGSSVK<br>RATPVTASSSSSSSSSS<br>SSSSTPSAFQIASASSS<br>SSSSSSSTTPSPAPRP<br>ALSSSGEKRKLEDIMG<br>GEEGFEKRTKTQDTV<br>CETRT
NP_013078.1	ScHif1	S. cerevisiae	NASP	MKLRAEDVLANGTSR<br>HKVQIDMERQVQIA<br>KDLLAQKKFLEAAKR<br>CQQTLDSLPKDGLLP<br>DPELFTIFAQAVYNM<br>EVQNSGNLFGDALLA<br>GDDGSGSESESEPES<br>DVSNGEEGNENGQT<br>EIPNSRMFQFDQEEE<br>DLTGDVDSGDSEDSG<br>EGSEEEEENVEKEEER<br>LALHELANFSPANEH<br>DDEIEDVSQLRKSGF<br>HIYFENDLYENALDLL<br>AQALMLLGRPTADG<br>QSLTENSRLRIGDVYIL<br>MGDIEREAEMFSRAI<br>HHYLKALGYYKTLKPA<br>EQVTEKVIQAEFLVCD<br>ALRWVDQVPAKDKL<br>KRFKHAKALLEKHMT<br>TRPKDSELQQARLAQ<br>IQDDIDEVQENQQH<br>GSKRPLSQPTTSIGFP<br>ALEKPLGDFNDLSQL<br>VKKKPRRH
XP_001030823.3	TtNASP	T. thermophi	NASP	EITKEQKILDKEENGD<br>QNNLNDEGSRNQEE<br>AEEITKILKESSSKVSE<br>KYLESRLTEAVECYKN<br>KNFDQAENIFSDLMI<br>YAVKFYKSETHVKMS<br>KYFFFYGDMLLSKLEN<br>NPDVFGDKAQKKLD<br>NQTTEEVEKNPNQM<br>ASEEASEQKMVAQK<br>EQKEEDEEENEESQS<br>QGEEEIVQLDTVIFKD<br>ENVEIRDETNAPQNN<br>EENQTEENNLPIGKV<br>TMEKSDPIDDLQLA<br>WENLELSRKIYNQELL<br>QLQDSQDEDRKEKQI<br>QVKKQIVEVTLRIADL<br>EQWRDNFSDALKEYT<br>MALQLSKEIEDREYSR<br>TLSSIFFQLGNAVLYE<br>NKEYCEEEGLKNFIDS<br>AQILENCLSKKIAIPPQ<br>ELPSFKILKKDDIKVD<br>HLKHSFLDDDESKEIK<br>DTLKVLYEKIEDTLIQR<br>DERDKYKAYQELMKK<br>QSQNQNQFAQPSQ

NP_001089907.1	XlNASPL	X. laevis	NASP	DTATAPSTSADKADG<br>MDIDTEAKRLMGAG<br>QRHLVMKDVRSSVTS<br>FQEASSLLAKKYGETA<br>DECAEAFYSYGTSLLE<br>LARMENGVLGNALE<br>GMPEDEEEGEKEEDA<br>NIPSADNLDEKEREQL<br>RDQVYDAMAEEERA<br>PKETSESEALGKPKDE<br>SKPMDTDEQNKPVE<br>DKMKNGNMETGKV<br>TDGLKLESVNRDVSM<br>DESEKGEVPESKNAP<br>TPGEHTEQKEKSEPKE<br>SKAPEKKDTKDTANQ<br>SPDSTEVAEERAHTD<br>TAEKMDSEASAKTEE<br>GASKTPTGNEASKSE<br>DPEKMKGEEEEGEDS<br>EESEENESTEEKETED<br>EDVGNLQLAWEMLD<br>LSKMIFKRQKSREAQ<br>LKAAQAHLKLGEVST<br>ESENYAQAVEDFLAC<br>LDIQKEHLEEHDRLLA<br>ETHYHLGLAYQYSSK<br>HEEAISHFTQSVGVIE
A0A804MWY6	ZmNASP	Z. mays	NASP	PQAPPPPDTEPTEAA<br>PGEAEDEPQTLERAQ<br>ELFDRGSKAIEDEDFV<br>DAVDCLSRALEIRASH<br>YGELAPECASTYFKYG<br>CALLYKAQEESDVLG<br>NVPKSLPNEDSVKST<br>ATKDDSGTSKVSGTD<br>AEDAASSEKVDAEEG<br>HNSNGKNQENGNG<br>EVEKDDDDVDDDEK<br>MGDEEDNDLDLSWK<br>MLDIARAIVEKSQDN<br>TMEKVKIYSALAEVAT<br>EREDMDNSLSDYMK<br>ALSMLEHLVEPDHRR<br>VVELNFRICLVYELVS<br>KIGEAISYCAKAISLCK<br>SRIQSLKSSKDALLAGI<br>DGDASAAEAEGGSEK<br>SAIEKELEQLTSILPDL<br>EKKLEDLSEANPSAD<br>MDEMVKAIVSRVTD<br>VMPKAASFTSSQMA<br>TSSNGFDSSVMSTAE<br>ASNELASSRSLLNLLLR<br>NLHLIHHLNKVTAAS<br>TLRLFLQRRLETSQYLS

Table S3: Pairwise comparison of protein sequence identity between *Physarum* histone chaperones and those from the other studied organisms. In red, are indicated the percentage of the two most related proteins to the Physarum chaperone.

Pp Chaperone	*Homo sapiens*	*Mus musculus*	*Danio rerio*	*Drosophila melanogaster*	*C.elegans*	*Xenopus laevis*	*Arabidopsis thaliana*	*Zea mays*	*Amborella trichopoda*	*Physcomitrella patens*	*Tetrahymena*	*Saccharomyces cerevisae*	*Dictyostelium*
PpCAF1A	17,50 %	18,00 %	19,40 %	15,40 %	18,60 %	18,40 %	17,90 %	14,60 %	17,60 %	**19,80 %**	12,90 %	19,60 %	**25,20 %**
PpCAF1B	26,30 %	28 %	27,30 %	20,10 %	20,80 %	**28,40 %**	28,40 %	28,10 %	27,10 %	27,70 %	22,90 %	26,60 %	**30,70 %**
PpCAF1C	67,30 %	67,30 %	67 %	65 %	62,20 %	67,30 %	62,30 %	62,80 %	30,80 %	30,00 %	48,70 %	27,20 %	68,20 %
PpHIRA	31,10 %	31,10 %	**31,90 %**	23,40 %	21,30 %	**32,30 %**	27,90 %	24,10 %	27,90 %	27,50 %	18,40 %	hir1 = 19.9%; hir2 = 18.9%	29,60 %
PpUBN	**HSUBN1= [illegible]**; HsUBN2= [illegible]	**MmUBN1 = [illegible]**; MmUBN2= [illegible]	DrUBN1= [illegible]; **DrUBN2= [illegible]**	16,40 %	12,10 %	XlUBN1=19 [illegible]; XlUBN2= [illegible]	AtUBN1 = [illegible]; AtUBN2= [illegible]	16,20 %	16,80 %	14,80 %	13,70 %	11,70 %	5,80 %
PpCABIN1	17,50 %	16,60 %	16,50 %		15 %	16,40 %	**19,20 %**	19 %	5,50 %	18,80 %		18,10 %	**20,90 %**
PpNASP	13,20 %	13,30 %	19,10 %	21,20 %	NASP1=18. [illegible]; NASP2 = [illegible]	15,40 %	17,20 %	21,10 %	**21,80 %**	21,20 %	18,80 %	15,60 %	**28,00 %**
PpASF1	**HsASF1A= 49.8%**; HsASF1B= 42.8%	**MmASF1A= 49.8%**; MmASF12= 43.1%	DrASF1A = 44.2%; DrASF1B=43.9%	47.5%	35.0%	**XlASF1A-A= 50.0%**; XlASF1A-B= 43.6%	AtASF1A = 42.9%; AtASF1B=44.2%	ZmASF1A= 46.5%; ZmASF1B= 42.2%	46.7%	42.1%	31.0%	35.2%	43.6%
PpMCM2	47.0%	46.5%	**48.2%**	45.9%	42.1%	**48.2%**	45.9%	42.8%	46.6%	**47.8%**	41.7%	42.0%	45.7%
PpIPO4	27.3%	27.7%	24.3%	DmArtemi [illegible]; DmApollo [illegible]	20.7 %	24.6%	31.7%	28.2%	**30.4%**	**30.5%**		23.8%	28.2%
PpSPT16	38.6%	38.5%	38.4%	37.1%	36.1%	39.1%	38.8%	**40.1%**	34.6%	39.9%	34.6%	33.3%	**42.2%**
PpSSRP1	27.2%	27.5%	DrSSRP1A= [illegible]; DrSSRP1B= [illegible]	27.8%	CeSSRP- [illegible]; CeSSRP1- [illegible]	28.5%	30.3%	30.6%	**31.5%**	**33.0%**	21.1%	ScPOB3=30.3%; ScNHP6A [illegible]; ScNHP6B [illegible]	41.2%
PpSPT6	28.8%	28.7%	28.1%	27.9%	24.1%	27.3%	27.6%	26.9%	29.7%	PpaSPT6 = [illegible]; PpaSPT6- [illegible]; PpaSPT6- [illegible]	21.4%	21.2%	31.3%
PpAPLF	17.4%	18.6%	13.3%	9.8%	19.3%	13.8%					17.3%		
PpSWR1	HsSRCAP = 24.3%; Hs-p400= 21.4%	MmSRCAP= 23.8%; Mm-p400= 22.5%	DrSRCAP=21.5%; Dr-p400=21.8 [illegible]	**28.5%**	27.4%	XlSRCAP= 20.9%; XL-p400= 21.3%	**28.1%**	26.0%	26.5%	27.1%	15.9%	21.1%	25.2%
PpSWC2	24.8%	23.1%	24.8%	**27.9%**	18.7%	26.5%	31.2%	26.5%	**28.3%**	21.0%	10.6%	10.6%	25.8%
PpPolE3	**33.5%**	33.3%	**34.0%**	31.4%	23.8%	32.3%			25.4%			15.7%	33.6%
PpHSP90	HsHSP90A= 68.5%; HsHSP90B=68.6%	MmHSP90A=68.4%; MmHSP90B=68.7%	DrHSP90A=67.3%; DrHSP90B=68.2%	66.9%	68.6%	XlHSP90A= 67.5%; XlHSP90B= 68.7%	AtHSP90.1 = 75.3%; **AtHSP90.2= 77.9%**	75.3%	75.5%	**77.7%**	68.9%	67.2%	74.5%
PpHSC70	**76.2%**	**76.2%**	**75.9%**	68.4%	74.7%	**75.9%**	74.0%	74.1%	75.1%	73.5%	67.4%	ScSSA1=7 [illegible]; ScSSA2=7 [illegible]; ScSSA3=7 [illegible]; ScSSA4=7 [illegible]	75.8%

	Homo sapiens									
PpNAP1L1	HsNAP1L [illegible]	HsNAP1L1 [illegible]	HsNAP1L2 [illegible]	HsNAP1L4 [illegible]	HsNAP1L5 [illegible]	HsNAP1L6 [illegible]	HsTSPY1 [illegible]	HsTSPYL [illegible]	HsTSPYL [illegible]	HsSET=2 [illegible]
PpSET	HsNAP1L [illegible]	HsNAP1L1 [illegible]	HsNAP1L2 [illegible]	HsNAP1L4 [illegible]	HsNAP1L5 [illegible]	HsNAP1L6 [illegible]	HsTSPY1 [illegible]	HsTSPYL [illegible]	HsTSPYL [illegible]	HsSET=3 [illegible]

	Mus musculus										
PpNAP1L1	MmNAP1L [illegible]	MmNAP1L [illegible]	MmNAP1L [illegible]	MmNAP1L [illegible]	MmNAP1L [illegible]	MmTSYL1 [illegible]	MmCINAP [illegible]	MmTSPYL [illegible]	MmTSYL4 [illegible]	MmTSPYL [illegible]	MmNCKP1= [illegible]
PpSET	MmNAP1L [illegible]	MmNAP1L [illegible]	MmNAP1L [illegible]	MmNAP1L [illegible]	MmNAP1L [illegible]	MmTSYL1 [illegible]	MmCINAP [illegible]	MmTSPYL [illegible]	MmTSYL4 [illegible]	MmTSPYL [illegible]	MmNCKP1= [illegible]

	Danio rerio							*Drosophila melanogaster*		*C.elegans*		
PpNAP1L1	DrSETB=2 [illegible]	DrSETA=2 [illegible]	DrNAP1L1 [illegible]	DrTSPY=1 [illegible]	DrNAP1L4 [illegible]	DrNAP1L4 [illegible]	DrNAP1L4 [illegible]	DmSET=2 [illegible]	DmNAP1= [illegible]	CeNAP1= [illegible]	CeSPR-2A [illegible]	CeSPR-2B [illegible]
PpSET	DrSETB=4 [illegible]	DrSETA=4 [illegible]	DrNAP1L1 [illegible]	DrTSPY=1 [illegible]	DrNAP1L4 [illegible]	DrNAP1L4 [illegible]	DrNAP1L4 [illegible]	DmSET=3 [illegible]	DmNAP1= [illegible]	CeNAP1=1 [illegible]	CeSPR-2A [illegible]	CeSPR-2B [illegible]

	Xenopus laevis				*Arabidopsis thaliana*					*Zea mays*
PpNAP1L1	XlNCPKP1= [illegible]	XlNAP1L1A [illegible]	XlNAP1L1B [illegible]	XlNAP1L1 [illegible]	AtNAP1=4 [illegible]	AtNAP1-1= [illegible]	AtNAP1-4= [illegible]	AtNRP1=2 [illegible]	AtNRP2=1 [illegible]	ZmNAP1= 3.6%
PpSET	XlNCPKP1= [illegible]	XlNAP1L1A [illegible]	XlNAP1L1B [illegible]	XlNAP1L1 [illegible]	AtNAP1=0 [illegible]	AtNAP1-1= [illegible]	AtNAP1-4= [illegible]	AtNRP1=4 [illegible]	AtNRP2=4 [illegible]	ZmNAP1=0.1%

	Amborella trichopoda				*Physcomitrella patens*	*Tetrahymna thermophila*			*Saccharomyces cerevisae*		*Dictyostelium discoideum*	
PpNAP1L1	AtrNRP2A [illegible]	AtrNRP2B [illegible]	AtrNAP1-4 [illegible]	AtrNAP1-4 [illegible]	PpaNAP1= 1.7%	TtNAP1L1=3 [illegible]	TtNAP1L2 [illegible]	TtNAP1L3 [illegible]	ScNAP1=30 [illegible]	ScVPS75=37.8%	DdNAP1=3 [illegible]	DdNAP2=17,3%
PpSET	AtrNRP2A [illegible]	AtrNRP2B [illegible]	AtrNAP1-4 [illegible]	AtrNAP1-4 [illegible]	PpaNAP1= 4.0%	TtNAP1L1=3 [illegible]	TtNAP1L2 [illegible]	TtNAP1L3 [illegible]	ScNAP1=17 [illegible]	ScVPS75=20.5%	DdNAP1=2 [illegible]	DdNAP2=22,7%

Table S4: List of primers

Primer ID	Sequence	Gene
HIRA_F	GTGAATGGATTGCAGGTGCC	HIRA
HIRA_R	GTGGGAGGGATGAACACAGG	HIRA
NASP_F	GTACCACCAGTTGGTGAGGG	NASP
NASP_R	CCGCTTTTTCAGGGTGCTTC	NASP
CAF1C_F2	ATTTTGCTTCTGCTGGCGAC	CAF1C
CAF1C_R2	AAACGGGTTAAAAGCCACGC	CAF1C
19S_QF	CAACGGGTACAGAGGATAAGGG	19S
19S_QR	ATTCATATGTCCCCTAACGCCC	19S

Table S5: **Prediction of NLS signals in chaperone proteins.**

Physarum Histone chaperone	Probability	Start	End	Signal Sequence
PpASF1	This protein has no NLS predicted by NLStradamus			
PpMCM2	0.792	115	125	KKRNRKVQKAK
	0.744	148	154	KPRRKRP
	1.000	378	399	KKKKKKKKKKKKKKKKKKKRSK
PpHSP90	0.879	244	251	KKKKKKVK
PpHSC70	This protein has no NLS predicted by NLStradamus			
PpIPO4	This protein has no NLS predicted by NLStradamus			
PpSPT6	0.998	63	74	REKKKKKKKKKR
	0.996	122	136	GGGGERRRRRKKKRR
	0.706	171	179	NKNSKKLKR
	0.643	240	245	KRSKQR
PpAPLF	0.837	25	35	KKEKEKEKKKG
	0.773	205	228	RGEKGEEGEKGEEGSPEKKGVKRK
	0.911	309	347	KPKTPKSKNSTPEKDAEIARKLQEKYNKESGGGGKKKRK
	0.889	362	372	KVKKQKKGKKG
	0.961	405	419	KNKKNKKKGASPKKP
PpPolE3	0.743	114	124	EKKEKGKDKEK
PpSWR1	0.808	1567	1575	REKRERKKH
	0.817	1590	1618	MPTKGGRGRKANGGLFPSRIVPKTARDGK
	0.779	1980	1992	KPSPSKRSKKNAE
PpSWC2	0.992	79	107	KEKKEKKRMAYVDPKAKKGPKRRKIQDKA
	0.990	137	155	APKAEPKKGRGGRRKKTPK
	0.893	200	219	REKEREETEKKRSQRSRKRS
PpSPT16	0.744	494	509	QKGKSKGSEDKIKKHQ
PpPob3	0.996	503	528	KPKKDKHEGKRKKSDGEEKPKKKKKA
PpSET	This protein has no NLS predicted by NLStradamus			
PpNAP1L1	0.964	231	245	KQTKKKTKAKGKKPA
	0.685	345	353	KRGGKRQGG
PpCAF1A	0.994	143	264	KPSPKKRKLSPEEKQAREDAKKAKEEERKLQAKQREDAKNKKEEEKRLLALAREEEKKKRDEEKRVKDQAKEEARLKKEAEKKARDDAKLQKDLEKKAKEEARLQKLQEKQKQEEEEKRKKE
	0.982	345	359	GKRNRKRDKKRRAEK
	0.850	499	513	HMERDKTKREKREKK
PpCAF1B	0.677	194	201	LPKNKKSH
	0.704	283	296	EKEKEESGKGKEKE
PpCAF1C	This protein has no NLS predicted by NLStradamus			
PpHIRA	0.999	599	669	TKKHKKDEESDKEKGKEAEKGDKNKDIEADHTKEKEREKERDKDREKERRRREKEKEKEKVRKEKEKEREA
PpUBN	0.717	190	194	RKKKR
	0.999	204	283	DKNKSKRTKKLKIEDEKGSPKEKDKKNDENGKKSKDKKPKDKKKEETPKDKEKKKEDSTKDKEKKKEDPGKEKEKKKEEK
	0.605	525	525	K
	0.981	615	644	KIKKEKAKNGEKKAAGPGGAKPAKVYSRKK
PpCABIN1	This protein has no NLS predicted by NLStradamus			
PpNASP	This protein has no NLS predicted by NLStradamus			

Table S6: Prediction of NES signals in chaperone proteins.

Physarum Histone chaperone	Score	Start	End	Signal Sequence	number of matches for the NES
PpCabin1	100.0	766	778	QT-L-GVS-L-ST-L-N-L-Q	1
PpIPO4	75.0	566	581	DA-M-SLA-L-AG-L-E-L-GFSE	1
PpSPT6	73.6	1216	1230	EE-L-KK-L-NKF-L-V-I-ELE	4
PpCAF1A	70.2	522	534	SS-A-KAI-L-TN-L-S-I-Q	2
PpSWR1	64.9	1764	1779	DV-L-AL-I-SQR-V-V-L-TMPQ	1
PpSPT6	61.1	634	647	Q-M-ED-F-QML-L-V-L-KAE	1
PpSPT6	61.1	959	972	S-L-QY-V-SG-L-G-V-RKAE	1
PpSPT16	56.2	839	854	SL-I-EPP-F-FV-L-T-L-KEIE	2
PpSPT16	56.2	106	121	EN-A-PYV-L-CF-L-Q-L-SKAE	1
PpMCM2	56.2	557	568	S-A-VG-L-TAA-V-R-I-D	1
PpSPT6	56.2	1308	1319	S-A-RS-L-GS-L-F-I-GE	1
PpCABIN1	51.8	157	170	S-I-DK-L-AEV-L-F-I-LGD	1
PpPob3	51.8	84	97	DV-L-KTF-L-SEN-F-H-V-E	1
PpNASP	51.8	204	217	DA-I-EE-F-KKC-V-S-L-RE	1
PpMCM2	49.2	262	274	Q-I-FDE-V-AMK-L-V-L-E	1
PpSPT6	48.7	474	489	ED-L-SD-L-QDY-F-Q-M-FYAD	2
PpPob3	46.4	114	125	EL-A-GP-V-IT-L-A-V-D	1
PpMCM2	43.9	878	891	QL-L-FH-I-LT-M-E-V-REQ	2
PpSPT16	41.2	324	336	S-I-KPG-V-KIS-V-I-M-D	1
PpMCM2	39.8	656	670	DP-I-LSR-F-DI-M-C-V-VRD	2
PpCAF1A	20.3	6	18	S-I-YLC-L-VWA-V-F-M-D	1
PpASF1	NA				
PpSET	NA				
PpNAP1L1	NA				
PpSWC2	NA				
PpPolE3	NA				
PpHSP90	NA				
PpHSC70	NA				
PpCAF1B	NA				
PpCAF1C	NA				
PpHIRA	NA				
PpUBN	NA				

Table S7: RNA-Seq data for histone chaperones during the cell cycle. Normalized read count are presented in TPM.

Physarum Encoded Protein	Physarum Transcript ID	Average early S-phase	SEM early S-phase	Average mid S-phase	SEM mid S-phase	Average late S-phase	SEM late S-phase	Average early G2-phase	SEM early G2 phase	Average late G2-phase	SEM late G2-phase
**PpASF1**	c_ppoly_21148	1685.64018535042	382.571302686029	1059.9304195573	492.595273451016	362.802070527273	108.002266976866	524.77419380426	121.605051084244	1935.94237626234	220.851430113619
**PpMCM2**	c_ppoly_24879	348.316651403373	107.612867406748	30.736854935386	6.7519544495917	18.0262054632708	9.33210563139798	19.6441524563809	8.78750018426791	320.298470991307	83.2392746707242
**PpIPO4**	c_ppoly_21477	500.07573411145	155.059505888504	285.169396424205	125.57755857642	300.045891422379	44.5007710355702	440.72913492516	226.920583929418	320.56752056917	99.5996768964357
**PpSPT6**	c_ppoly_23850	946.221323399772	267.782426259667	801.75039129217	196.592902112129	796.128154000321	225.869751198728	883.769437899125	418.910375801823	535.910510796423	108.739600667991
**PpPolE3**	c_ppoly_20662	498.946034384142	84.3251390005563	369.130590586745	20.427037370197	38.8726987573958	19.8434389071508	60.3746550698003	16.3747911981762	730.295813248133	202.149890161534
**PpHSP90**	c_ppoly_11600	20007.6443158073	6426.45272292963	9734.37029857941	5156.02179431029	15770.1416854257	265.79873656357	8178.16622784559	1159.11881355825	15918.9721285622	4950.54779397259
**PpHSC70**	c_ppoly_14645	23225.7456694507	3247.06191059978	9646.97444768135	3701.48661572195	24989.3498155015	9841.83672566814	17255.8499772107	5068.55676580514	18211.4644589064	3051.81467142549
**PpNASP**	c_ppoly_25875	1629.35403343165	620.414116060328	363.899319613087	209.842458800485	100.358580632022	49.2779416872384	135.875297632927	63.9403874024608	922.890744802152	440.673737748532
**PpCAF1A**	c_ppoly_04687	597.254804471717	120.092250699239	62.1585607690401	23.4137215227569	26.8581363237992	13.1076862635872	28.3681013175196	14.1973006626964	434.341742433927	99.4410586715044
**PpCAF1B**	c_ppoly_07527	750.710058213592	233.916619770675	183.331866595398	79.089799099446	14.2463273177883	7.91558909639024	6.39873554427367	2.74463936330242	493.114229648283	97.6520207782227
**PpCAF1C**	c_ppoly_21398	2501.2459346085	578.127883384376	3371.9451619237	764.048482179825	1943.08803111066	509.35511092029	2459.11637656382	271.929299332971	2379.00764440829	288.673270974413
**PpHIRA**	c_ppoly_12971	989.598820352507	140.79766480616	1160.8239286344	197.737924512506	1212.72394731905	452.371827102978	1134.73843959412	334.63691041208	760.968307356968	70.766415233273
**PpUBN**	c_ppoly_06650	378.743171268866	88.0311047036758	688.542276271353	26.190024394418	682.568343394393	262.45494899595	509.773405608509	70.615779913131	539.487296205266	90.4127318057242
**PpCABIN1**	c_ppoly_25516	305.317386411327	108.982428985004	123.793553723011	34.3114249875473	87.3060935803543	30.8516180968159	113.957483964267	47.5692809749332	182.024985513339	30.267088246206
**PpAPLF**	c_ppoly_14659	602.253242194539	117.392752510644	696.635847592961	119.153243588837	567.470471090983	141.273674480576	594.889695389578	192.973403903031	463.49685999437	45.6443812599786
**PpSWR1**	c_ppoly_23444	646.256991190687	44.8386018649976	524.264004191157	114.675703587593	750.759297095279	200.595591582832	809.632893598996	283.378733605977	445.385395046608	51.3320997499832
**PpSWC2**	c_ppoly_14213	76.3530595685763	12.1138778936789	287.056516156046	60.1224577135304	192.637043757318	24.4457821070988	121.082884673759	7.89472148236264	104.872094197058	8.93252644801636
**PpSPT16**	c_ppoly_05072	1636.36073180243	303.322016727763	1199.89815004542	384.411533528417	599.805837571488	39.3030725590596	882.275887448549	206.48198026475	1607.57888712835	223.137689211474
**PpPob3**	c_ppoly_22049	2229.8086235541	570.071074636041	834.499671535581	296.684402950269	485.228384979042	144.516443107806	908.926892518441	279.746447641578	2779.57096039765	400.016961261955
**PpNAP1L1**	c_ppoly_06693	13756.1314103008	3384.84587293737	15303.6934508884	3841.67850719629	13997.2210675457	2383.26933024534	13826.715003288	3013.02502051686	10769.7059192618	2239.71095674852
**PpSET**	c_ppoly_06196	3864.36061664834	508.320802019727	7191.29184354547	2647.07569765998	4959.35118588884	732.62386714741	4791.89302691023	777.030852241146	4052.06069090031	209.469028677767
**PpHT01**	c_ppoly_11752	224.696253946371	64.0543175847619	82.1480278004567	36.0232191739293	56.8407614086324	17.1144801265771	31.3177687480039	14.1154140043007	201.232068992229	82.272118714642
**PpHTA2**	c_ppoly_09597	915.261558275856	157.750403876033	597.704488201613	74.6491583767923	540.232443693337	71.0450192193106	478.278797518563	94.3203004094633	919.533399670732	293.589766273955
**PpHTA3**	c_ppoly_08402	12768.2104766242	1764.19512439209	19699.028596941	8527.599947596	6298.59967750296	1310.25871828244	5418.08853376133	756.639194212458	13212.4754814818	1176.4678460523
**PpHTB1**	c_ppoly_07519	516.711905099623	73.1012567019167	286.04909909138	59.1602429755268	311.385778068313	141.223766772719	247.940835274094	72.7312655722548	437.695433961764	144.971408575862
**PpHTB2**	c_ppoly_07520	19591.0487732844	3852.84466629448	19477.6081457671	7330.41880876511	5953.18640798085	1305.43618961695	6995.68294236743	947.448208448614	29290.2210913313	9024.79107813862
**PpHTT1**	c_ppoly_23074	362.936493621585	30.7899100607491	184.837175371919	54.7652150451114	175.238136352701	72.839740023364	78.117185948318	8.82195351238481	343.537584809706	114.774352425865
**PpHTT2**	c_ppoly_07595	245.457643975657	71.5039049378998	228.797500426871	64.5931817164329	170.908285886344	40.1595818055351	211.854606562614	54.5582604859831	165.719077762714	24.6101279199501
**PpHTT3**	c_ppoly_07042	813.285604922061	93.9522087593013	354.217927444129	51.6391828541076	311.668949854076	106.117662637738	509.168664755438	289.786224425149	552.733119862111	246.6473716141
**PpHTT4**	c_ppoly_00698	9895.6108639726	724.633057838997	19329.1179871322	5883.7362724994	12788.8950614719	1092.30683795137	11143.0920774536	466.57600829916	9981.80779712691	319.802030472577
**PpHTT5**	c_ppoly_10421	29.3192111194444	8.99358097544886	176.432671524867	37.6843272724607	84.3202148809206	9.232765909262	34.7932332606828	9.39613097875041	41.1153001275873	7.74354993753454
**PpHTF1**	c_ppoly_07791	3084.03601807698	576.640520811421	3191.90476212654	212.242124852856	3038.563595261	1064.27838964211	2143.55897320622	553.386093720452	4777.70786964036	1177.45821282105
**PpHTF2**	c_ppoly_15298	771.495274942455	189.771049071911	574.531486893002	50.5536608956485	548.001158296472	39.0745859034198	389.985749592906	47.1831650477286	844.051363434518	374.735789162973

Table S8: RNA-Seq file details.

Sample Name	cell cycle phase	Sequence i7	Sequence i5	R1 file Name	R2 file Name
S4	early S-phase	TCCGGAGA	TATAGCCT	S4_TCCGGAGA-AGGCTATA-AHFMH7DRXY_L001_R1.fastq	S4_TCCGGAGA-AGGCTATA-AHFMH7DRXY_L001_R2.fastq
S13	early S-phase	TCCGGAGA	ATAGAGGC	S13_TCCGGAGA-GCCTCTAT-AHFMH7DRXY_L001_R1.fastq	S13_TCCGGAGA-GCCTCTAT-AHFMH7DRXY_L001_R2.fastq
S24	early S-phase	TCCGGAGA	CCTATCCT	S24_TCCGGAGA-AGGATAGG-AHFMH7DRXY_L001_R1.fastq	S24_TCCGGAGA-AGGATAGG-AHFMH7DRXY_L001_R2.fastq
S6	mid S-phase	CGCTCATT	TATAGCCT	S6_CGCTCATT-AGGCTATA-AHFMH7DRXY_L001_R1.fastq	S6_CGCTCATT-AGGCTATA-AHFMH7DRXY_L001_R2.fastq
S16	mid S-phase	CGCTCATT	ATAGAGGC	S16_CGCTCATT-GCCTCTAT-AHFMH7DRXY_L001_R1.fastq	S16_CGCTCATT-GCCTCTAT-AHFMH7DRXY_L001_R2.fastq
S7	late S-phase	GAGATTCC	TATAGCCT	S7_GAGATTCC-AGGCTATA-AHFMH7DRXY_L001_R1.fastq	S7_GAGATTCC-AGGCTATA-AHFMH7DRXY_L001_R2.fastq
S18	late S-phase	GAGATTCC	ATAGAGGC	S18_GAGATTCC-GCCTCTAT-AHFMH7DRXY_L001_R1.fastq	S18_GAGATTCC-GCCTCTAT-AHFMH7DRXY_L001_R2.fastq
S28	late S-phase	GAGATTCC	CCTATCCT	S28_GAGATTCC-AGGATAGG-AHFMH7DRXY_L001_R1.fastq	S28_GAGATTCC-AGGATAGG-AHFMH7DRXY_L001_R2.fastq
S10	early G2 phase	ATTCAGAA	TATAGCCT	S10_ATTCAGAA-AGGCTATA-AHFMH7DRXY_L001_R1.fastq	S10_ATTCAGAA-AGGCTATA-AHFMH7DRXY_L001_R2.fastq
S20	early G2 phase	ATTCAGAA	ATAGAGGC	S20_ATTCAGAA-GCCTCTAT-AHFMH7DRXY_L001_R1.fastq	S20_ATTCAGAA-GCCTCTAT-AHFMH7DRXY_L001_R2.fastq
S29	early G2 phase	ATTCAGAA	CCTATCCT	S29_ATTCAGAA-AGGATAGG-AHFMH7DRXY_L001_R1.fastq	S29_ATTCAGAA-AGGATAGG-AHFMH7DRXY_L001_R2.fastq
S2	late phase	ATTACTCG	TATAGCCT	S2_ATTACTCG-AGGCTATA-AHFMH7DRXY_L001_R1.fastq	S2_ATTACTCG-AGGCTATA-AHFMH7DRXY_L001_R2.fastq
S12	late phase	ATTACTCG	ATAGAGGC	S12_ATTACTCG-GCCTCTAT-AHFMH7DRXY_L001_R1.fastq	S12_ATTACTCG-GCCTCTAT-AHFMH7DRXY_L001_R2.fastq
S22	late phase	ATTACTCG	CCTATCCT	S22_ATTACTCG-AGGATAGG-AHFMH7DRXY_L001_R1.fastq	S22_ATTACTCG-AGGATAGG-AHFMH7DRXY_L001_R2.fastq
